# The second survey of the Saudi Acute Myocardial Infarction Registry Program: Main results and temporal changes in care (STARS-2 program)

**DOI:** 10.1371/journal.pone.0331215

**Published:** 2025-09-02

**Authors:** Ayman AlSaleh, Shukri M. AlSaif, Osama Alhadramy, Mohammed Alshehri, Khalid Al Faraidy, Fawaz Almutairi, Abdulhalim J. Kinsara, Mushabab Al-Murayeh, Abdullah E. Ghabashi, Mirvat Alasnag, Gamal Abdin Hussein, Tamer M. Askar, Kamel H. Haider, Ibrahim A. Alharbi, Abdulaziz Almokhlef, Belal A. Sayed, Adel Almasswary, Zia- ul-Sabah, Hameedullah Kazim, Hazem A. Albareda, Mubarak A. Aldossari, Rasha Albawardi, Rudaynah Alali, Ibrahim A. M. Abdulhabeeb, Saifeldin Mohamed Ibrahim, Sami Alasmari, Abdulrahman M. Almoghairi, Abdullateef Y. Khoja, Naveed Hussain, Balarabe S. Aminu, Abdulhalim Serafi, Abdalla Osman Eltayeb, Ali Husain BuSaleh, Basel Alsabatien, Mirghani K. Hamza, Rasha Alsharkawy, Awatif A. Awwad, Maha A. Mohamed, Mohammed A. Al Habeeb, Syed Shujauddin, Jamila Ado Ya’u, Nashwa M. Attia, Naji Kholaif, Nazeeh M. Bin Ghouth, M.Kheir I. Youssef, Mohammed A. Qutub, Samih R. Lawand, Raed Alkutshan, Emadaldein Ahmed, Ayman Basardah, Wasem I. Alhaj, Hani Altaradi, Muhammad Ali, Wael Alqarawi, Khalid F. Alhabib, on behalf of the STARS-2 Investigators

**Affiliations:** 1 Department of Cardiac Sciences, King Fahad Cardiac Center, College of Medicine, King Saud University, Riyadh, Saudi Arabia; 2 Saud AlBabtain Cardiac Center (SBCC), Dammam, Eastern Province, Saudi Arabia; 3 Saudi German Hospital, Al Madinah, Saudi Arabia; 4 Department of Medicine, College of Medicine, Taibah University, Medina, Saudi Arabia; 5 Military Hospital Cardiac Center, Khamis Mushait, Aseer, Saudi Arabia; 6 King Fahd Military Medical Complex Cardiac Center, Dhahran, Eastern Province, Saudi Arabia; 7 National Guard Riyadh, Riyadh, Saudi Arabia; 8 Ministry of National Guard Health Affair, King Saud bin Abdulaziz University for Health Sciences, COM-WR. King Abdullah International Medical Research Center, Jeddah, Makkah, Saudi Arabia; 9 Prince Faisal Bin Khalid Cardiac Center, Abha, Saudi Arabia; 10 King Abdullah Medical City, Makkah, Saudi Arabia; 11 King Fahad Armed Forces Hospital, Ministry of Defense Health Services, Jeddah, Saudi Arabia; 12 King Salman Armed Forces Hospital (KSAFH) in Northwest Region, Tabuk, Saudi Arabia; 13 King Khalid General Hospital, Alkharj, Riyadh, Saudi Arabia; 14 Prince Mohammed Bin Nasser Hospital, Jizan, Saudi Arabia; 15 Prince Sultan Cardiac Center, Qassim, Saudi Arabia; 16 Baish General Hospital, Jizan, Saudi Arabia; 17 Aseer Central Hospital, Abha, Aseer, Saudi Arabia; 18 Prince Faisal Bin Khalid Cardiac Center, King Khalid University, PFKCC, Abha, Aseer, Saudi Arabia; 19 College of Medicine, King Khalid University, Prince Faisal Bin Khalid Cardiac Center, Abha, Aseer, Saudi Arabia; 20 Alhada Armed Forces Hospital, Taif, Makkah, Saudi Arabia; 21 King Khalid Hospital, Najran, Saudi Arabia; 22 King Saud Medical City (KSMC), Riyadh, Saudi Arabia; 23 King Fahd Hospital of the University, AL Khobar, Eastern Province, Saudi Arabia; 24 Cardiac Center in King Abdulaziz Specialist Hospital, Sakaka, AlJawf, Saudi Arabia; 25 Prince Abdallah bin Abdulaziz bin Musaad Cardiac Center (PAAMCC), Arar, Northern Borders, Saudi Arabia; 26 Prince Mohammed bin Abdulaziz Hospital, Riyadh, Saudi Arabia; 27 Adult Cardiology Department, Prince Sultan Cardiac Center (PSCC), Riyadh, Saudi Arabia; 28 King Fahd General Hospital, Jeddah, Makkah, Saudi Arabia; 29 King Abdullah Hospital, Bisha, Aseer, Saudi Arabia; 30 Samtah General Hospital, Samtah, Jazan, Saudi Arabia; 31 International Medical Center Hospital, Jeddah, Makkah, Saudi Arabia; 32 Almana General Hospital, Dammam, Eastern Province, Saudi Arabia; 33 Qateef Central Hospital, Qateef, Eastern Province, Saudi Arabia; 34 Specialized Medical Center (SMC), Riyadh, Saudi Arabia; 35 King Fahd Central Hospital, Jazan, Saudi Arabia; 36 King Salman Hospital, Riyadh, Saudi Arabia; 37 Al Thager Hospital, Jeddah, Makkah, Saudi Arabia; 38 Jazan General Hospital, Jazan, Saudi Arabia; 39 Jubail General Hospital, Jubail, Eastern Province, Saudi Arabia; 40 Alrass Hospital, Alrass, Qassim, Saudi Arabia; 41 Abu Arish General Hospital, Jazan, Saudi Arabia; 42 Sabya General Hospital, Sabya, Jazan, Saudi Arabia; 43 King Faisal Specialist Hospital & Research Centre, Riyadh, Saudi Arabia; 44 Domat Al Jandal General Hospital, Dumah Al Jandal, Saudi Arabia; 45 Sedair Hospital, Riyadh, Saudi Arabia; 46 Department of Medicine, Faculty of Medicine, King Abdulaziz University, Jeddah, Saudi Arabia; 47 Dallah Hospital, Riyadh, Saudi Arabia; 48 Royal Commission Health Services Program in Jubail, Jubail, Saudi Arabia; 49 King Saud University Medical City, Riyadh, Saudi Arabia; 50 King Saud University Medical City, King Saud University, Riyadh, Saudi Arabia; 51 Saudi Heart Association, Riyadh, Saudi Arabia; 52 University of Ottawa Heart Institute, University of Ottawa, Ottawa, Ontario, Canada; Cedars-Sinai Heart Institute, UNITED STATES OF AMERICA

## Abstract

**Background:**

The Saudi Acute Myocardial Infarction Registry (STARS) program aims to evaluate the clinical characteristics, management, and outcomes of a representative sample of patients with acute myocardial infarction (AMI) in Saudi Arabia. This second phase evaluates temporal changes in patient care, demographics, and the management benchmarks for AMI.

**Methods and findings:**

We created a 5-year recurring, multi-center prospective registry that utilizes a snapshot design in 50 hospitals from various healthcare sectors in Saudi Arabia. The study’s recruitment phase spanned from September 3, 2021, to January 6, 2023. During these 16 months, 2,690 patients presenting with acute myocardial infarction (AMI) with or without ST-segment elevation (STEMI or NSTEMI, respectively) were enrolled. The mean age (± SD) of the overall population was 57 (±12.4) years, 70% were Saudi citizens, 82% were men, and (48.8%) of the total patients had STEMI. Fifty-eight percent of patients had diabetes mellitus and 58% had hypertension. Of the total population with STEMI, primary percutaneous coronary intervention (PCI) was performed in 619 patients (47.1%), thrombolytics were given to 584 patients (44.5%), and 110 patients had no reperfusion (8.4%). Among patients who presented within 24 h of symptom onset, the door-to-balloon (DTB) time was 63 min (IQR: 43), with 75.6% achieving DTB < 90 min, whereas the door-to-needle (DTN) was 25 min (IQR: 34), with 57% achieving DTN < 60 min. Thirty-nine percent of patients failed lytic reperfusion and 96% of these required rescue PCI. In 52% of instances, the failure to receive reperfusion therapy was attributed to patients’ late presentation. At presentation, only 8.5% of cases were transferred by the Emergency Medical Services. Approximately one-fourth of patients with NSTEMI did not undergo a coronary angiogram. All-cause mortality was 2.4% with no significant difference between sexes or nationalities.

**Conclusion:**

This nationwide AMI registry revealed younger age at presentation with a high prevalence of risk factors for coronary artery disease. While primary PCI key performance indicators have improved from the previous phase, further progress is needed in EMS utilization and acute revascularization for STEMI and NSTEMI.

## Introduction

The Global Burden of Disease study in 2022 estimated that the leading cause of death in Saudi Arabia was ischemic heart disease, with an age-adjusted mortality rate of 329.5–379.6 per 100,000 population [1]. Registry studies enhance the healthcare of patients presenting with acute myocardial infarction (AMI) by assessing their real-life clinical presentation, management, and outcomes. Saudi Arabia’s healthcare system comprises several independent sectors, making it difficult to standardize an AMI-nationwide registry. We have demonstrated in the previous region’s registries compared to other worldwide registries that Saudi patients have a high-risk factor prevalence for coronary artery disease (CAD) Furthermore, they tend to develop acute coronary syndrome (ACS) at a younger age [2–4]. The limitation of these registries is an under-representation of non-tertiary care centers of the Ministry of Health, peripheral hospitals, and private hospitals. Moreover, there is a clear under-representation of the non-Saudi population, which comprises a significant proportion of healthcare in Saudi Arabia, with different demographics and limited access to certain tertiary care centers. Non-Saudis represent 36.4% of the total population of 34.1 million people in Saudi Arabia [5].

To address the limitations of earlier registries, the persistent burden of ischemic heart disease in Saudi Arabia, and the need to track temporal changes over time, we launched the Saudi Acute Myocardial Infarction Registry (STARS) program, as detailed in Phase 1. [6]. We have conducted the second phase to evaluate temporal changes in patient care, demographics, and AMI management benchmarks in Saudi Arabia following the same phase 1 design to determine whether there has been an impact on patient care.

## Methods

### Study design and population

The study design was described previously [6]. Briefly, this prospective multicenter study utilizes a snapshot design by enrolling AMI patients from both catheterization laboratory hospitals (Cath-Lab hospitals) and non-catheterization laboratory hospitals (Non-Cath-Lab hospitals) during two temporal phases, with the primary objective of mitigating the risk of double-counting the same patient between these hospital types. Given the constrained data collection period of up to four months, each participating hospital rigorously adhered to the recruitment time-frame as stipulated by their respective Institutional Review Board (IRB) approvals. This approach ensured the integrity of our sample and the validity of our subsequent analyses (S2 Table). The study’s recruitment phase spanned from September 3, 2021, to January 6, 2023. During these 16 months, 2,690 patients presenting with acute myocardial infarction (AMI) were enrolled. The affiliated hospitals represent all geographic areas of Saudi Arabia and are from different healthcare sectors (e.g., Ministry of Health, private). The program will be repeated every 5 years to provide a reliable assessment of temporal changes in AMI care in Saudi Arabia S1 File.

### Study objectives and definitions

This study aimed to assess the clinical presentation, management, and in-hospital course of patients, as well as in-hospital mortality rates. In addition, we assessed the use of guideline-recommended medical treatments and the proportion of patients with uncontrolled CAD risk factors. We employed standard definitions for these clinical variables based on the American College of Cardiology and the European Society of Cardiology guidelines [7,8]. Accordingly, the availability of serum troponin data, rather than creatinine kinase-MB fraction, was a mandatory requirement for hospital enrollment in the registry to ensure a highly reliable diagnosis of non-ST elevation myocardial infarction (NSTEMI).

### Study organization

A Registry Steering Committee, consisting of experienced cardiologists from all five major geographic regions of the country, was responsible for recruiting hospitals, monitoring the study progress in their respective regions, watching over the overall study conduct, and approving the final study results. Throughout the hospital stay of each patient, a case report form (CRF) was completed online by dedicated research assistants, physicians, and/or trained nurses working in each hospital. A logbook was maintained to ensure enrollment of all consecutively admitted patients. All CRFs were verified by a cardiologist before being sent electronically to the KFCC (King Fahad Cardiac Center, King Saud University, Riyadh), where they were checked for data queries or entry errors by a dedicated research coordinator and the Principal Investigator before submission for final analysis.

### Ethics statement

Ethics approval was obtained from the Institutional Review Board (IRB), College of Medicine, King Saud University, Riyadh: Ref. No. 21/0267/IRB and the other participating centers from Saudi Arabia: King Fahd Armed Forces Hospital – Jeddah Research and Ethics Committee, REC 400; ARMED FORCES HOSPITALS SOUTHERN REGION, AFHSRMREC/2021/CARDIOLOGY/496; Prince Sultan Military Medical City Scientific Research Center, 1461; SBCC-IRB-MC-2021-01; Specialized Medical Center, H-01-R-056; Armed Forces Hospitals Eastern Province, AFHER-IRB-2021–003; King Khalid Hospital, Najran, KSA: H-l1-N-081; the Royal Commission Health Services Program in Jubail, IRB-RCH-020; King Fahd General Hospital, Jeddah, Makkah, KSA: H-02-J-002. Since the study followed a snapshot design with a maximum data collection period of four months, IRB approval renewal from participating hospitals was not required. All data were fully de-identified for analysis and manuscript preparation. As is customary in STARS-1, STARS-2 consent was waived by the institutional review board, given the observational design of the study and the fact that patient identities remained anonymous. This decision was predicated on the absence of direct interaction or experimental manipulation inherent in the study methodology.

### Statistical methods

Categorical data were summarized as absolute numbers and percentages. Continuous data were summarized as means and standard deviations (SDs) or medians and interquartile ranges (IQRs). Comparisons of categorical variables between groups were made using Chi-Square test or Fisher’s exact test. Continuous data comparisons between groups were made using the student t-test. Adjusted odds ratios with 95% confidence intervals (CI) were estimated using logistic regression models, in which adjustments were made for significant variables in the univariate analysis. All analyses were performed with SAS version 9.2 (SAS Institute, Inc., Cary, NC) and R software (R Foundation for Statistical Computing, Vienna, Austria).

## Results

### Enrolled hospitals and study population

We identified 416 eligible hospitals, and we invited 50 hospitals representing 12% of the healthcare facilities across the 13 provincial administrative regions of the country. Nineteen were non-Cath-lab hospitals (39%). The various healthcare sectors represented in the study are similar to those included in the overall healthcare system in Saudi Arabia (S1, S2 and S3 Figs). Between September 3, 2021, to January 6, 2023, a total of 2,690 patients with AMI were enrolled in the study. Of these, 1313 (48.8%) patients had ST-elevation myocardial infarction (STEMI) and 1377 (51.1%) had NSTEMI. Baseline characteristics, clinical presentation, and cardiac procedures are provided in Table 1. The overall population mean age (± SD) was 57 (±12.4) years, 70% were Saudi citizens, and 82% were men. Of the non-Saudis, 32% were of Arab ethnicity. The prevalence of CAD risk factors was high; 58% of patients had diabetes mellitus (DM), 58% had hypertension (HTN), and 43% were either current or ex-smokers. The mean body mass index was 28.5 (±4.7) kg/m^2^. At presentation, only 8.5% of patients were transferred by the Emergency Medical Services (EMS, i.e., Saudi Red Crescent); 87% had typical chest pain; and 12% had clinical evidence of congestive heart failure.

**Table 1:** Baseline characteristics, clinical presentation, and cardiac procedures in patients with acute Myocardial infarction.

Characteristic	Total (2,690)	STEMI (1313)	NSTEMI (1377)	P value
Male, n (%)	2209 (82.1%)	1170 (89.1%)	1039 (75.5%)	<0.0001
Age, Mean ± SD	57.2 ± 12.4	55.3 ± 11.97	58.95 ± 12.6	<0.0001
Saudis, n (%)	1862 (69.22%)	791 (60.24%)	1071 (77.78%)	<.0001
Ethnicities of non-Saudis, n (%)				
Arab	2099 (78.03%)	926 (70.53%)	1173 (85.19%)	<.0001
South Asian	505 (18.77%)	340 (25.89%)	165 (11.98%)	
Others	86 (3.20%)	47 (3.58%)	39 (2.83%)	
BMI, Mean ± SD	28.53 ± 4.77	28.06 ± 4.4	28.97 ± 5.06	<.0001
Type of STEMI, n (%) 1313				
Anterior	690 (25.65%)	690/1313 = 52%		
Inferior	545 (20.26%)	545/1313 = 42%		
Other	78 (2.90%)	78/1313 = 6%		
Medical History, n (%)				
Angina	740 (27.51%)	252 (19.19%)	488 (35.44%)	<.0001
Myocardial infarction	403 (14.98%)	112 (8.53%)	291 (21.13%)	<.0001
PCI	419 (15.58%)	112 (8.53%)	307 (22.29%)	<.0001
CABG	71 (2.64%)	15 (1.14%)	56 (4.07%)	<.0001
Heart failure	163 (6.06%)	31 (2.36%)	132 (9.59%)	<.0001
Stroke	123 (4.57%)	35 (2.67%)	88 (6.39%)	<.0001
Chronic renal failure	200 (7.43%)	29 (2.21%)	171 (12.42%)	<.0001
Diabetes Mellitus	1559 (57.96%)	685 (52.17%)	874 (63.47%)	<.0001
Hypertension	1574 (58.51%)	643 (48.97%)	931 (67.61%)	<.0001
Hypercholesterolemia	1049 (39%)	444 (33.82%)	605 (43.94%)	<.0001
Current/ex-smoking	1167 (43.38%)	643 (48.97%)	524 (38.05%)	<.0001
Chief complaint, n (%)	2,690			<.0001
Chest pain	2344 (87.14%)	1203 (91.62%)	1141 (82.86%)	
Shortness of breath/Fatigue	176 (6.54%)	29 (2.21%)	147 (10.68%)	
Epigastric/shoulder/back/neck pain	127 (4.72%)	59 (4.49%)	68 (4.94%)	
Cardiac arrest	14 (0.52%)	13 (0.99%)	1 (0.07%)	
Others	29 (1.08%)	9 (0.69%)	20 (1.45%)	
Transferred by EMS, e.g., Red Crescent or Red Cross	229 (8.5%)	156 (11.88%)	73 (5.30%)	<.0001
First Medical Contact before presenting to hospital, n (%)	619(23.3%)	393(29.93%)	226(16.41%)	<.0001
EMS	131 (21.16%)	72 (18.32%)	59 (26.11%)	
Emergency Department	358 (57.83%)	242 (61.58%)	116 (51.33%)	
Clinic/Doctor	129 (20.84%)	78 (19.84%)	51 (22.56%)	
Pharmacy	1 (0.16%)	1 (0.25%)	0	
Status upon hospital arrival				
Heart rate, Mean ± SD	84.16 ± 17.7	83.5 ± 18.98	84.7 ± 16.47	0.0987
Systolic blood pressure, Mean ± SD	134.8 ± 25.45	132.2 ± 26.6	137.4 ± 23.9	<.0001
Heart rate>100 bpm, n (%)	355(13.2%)	175(13.3%)	180(13.1%)	0.8443
Systolic blood pressure<90mmHg, n (%)	68(2.5%)	49(3.7%)	19(1.4%)	0.0001
Cardiac arrest	74 (2.75%)	54 (4.11%)	20 (1.45%)	<.0001
Heart Failure Killip Class, n (%)				0.0009
Class I	2358 (87.65%)	1166 (88.80%)	1192 (86.56%)	
Class II/III	291 (10.85%)	118 (8.98%)	173 (12.56%)	
IV	41 (1.52%)	29 (2.2%)	12 (0.8%)	
Echo-Options, n (%)	2385(89%)	1104(84%)	1281(93%)	<.0001
Normal LV systolic function (EF > 50%)	947(39.7%)	337(30.5%)	610(47.6%)	
Mild LV systolic dysfunction (EF 40–50%)	769(32.2%)	393(35.6%)	376(29.3%)	
Moderate LV systolic dysfunction (EF 30–40%)	460(19.3%)	261(23.6%)	199(15.5%)	
Severe LV systolic dysfunction (EF < 30%)	209(8.76%)	113(10.2%)	96(7.5%)	
Arterial access, n (%)				0.463
Femoral	380 (23.9%)	193 (23.2%)	187(24.8%)	
Radial	1205(76%)	638(76.8%)	567(75.2%)	

Values are the number of patients (%), unless indicated otherwise. MI: myocardial infarction, AMI: acute MI, PCI: Percutaneous coronary intervention, CABG: Coronary artery bypass surgery, BMI: Body mass index, HTN: hypertension, HR: Heart Rate, SBP: systolic blood pressure, CHF: chronic heart failure, STEMI: ST-Elevation Myocardial Infarction, NSTEMI: Non-ST-Elevation Myocardial Infarction, EMS: Emergency Medical Services, LV: left ventricular, and EF: ejection fraction.

Compared to patients with STEMI, those with NSTEMI were more likely to be older, Saudi citizens, and have a history of angina, myocardial infarction, percutaneous coronary intervention (PCI), coronary artery bypass grafting (CABG), heart failure, chronic renal failure, DM, HTN, and/or hypercholesterolemia (Table 1). However, compared to NSTEMI patients, STEMI patients were more often men, current or ex-smokers, and presented with typical ischemic chest pain or cardiac arrest.

### STEMI presentation and management

**Fig 1:**
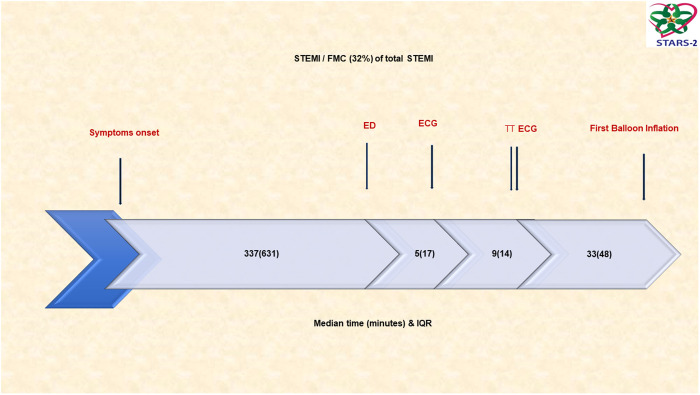
Timeline of patients with ST-segment elevation myocardial infarction who had FMC. The total ischemic time (TIT)/primary PCI 285 min (IQR: 274). The total ischemic time (TIT)/thrombolytics 278 min (IQR: 142). TT: Thrombolytic Time; ED: Emergency Department; PCI: Percutaneous Coronary Intervention; FMC: First Medical Contact.

Of the total population with STEMI, primary PCI was performed in 619 patients (47.1%), thrombolytics were given to 584 patients (44.5%), and 110 patients had no reperfusion (8.4%). Among the 1,313 patients diagnosed with ST-segment elevation myocardial infarction (STEMI), 32% initiated their medical care by seeking first medical contact, which involved visiting a peripheral hospital, primary care clinic, or pharmacy. The median time from symptoms to the Emergency Department (ED) was 337 minutes (min) (IQR: 631), from ED to ECG 5 min (IQR: 7), from ECG to thrombolytics 9 min (IQR: 19), and from ECG to balloon 33 min (IQR: 48). The total ischemic time (TIT) for primary PCI was 285 min (IQR: 274), and the TIT for thrombolytics 278 min (IQR: 142) (Fig 1).

**Fig 2:**
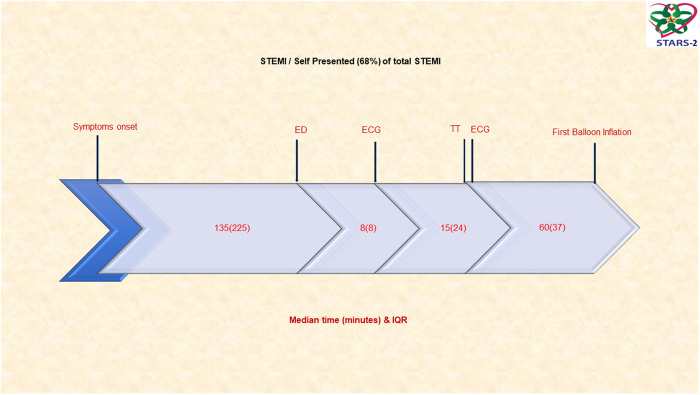
Timeline of patients with ST-segment elevation myocardial infarction who are self-presented. The Total Ischemic Time (TIT)/primary PCI 220 min (IQR: 250). The Total Ischemic Time (TIT)/thrombolytics 300 min (IQR: 400). TT: Thrombolytic Time; ED: Emergency Department; PCI: Percutaneous Coronary Intervention; FMC: First Medical Contact.

On the other hand, 68% of patients self-presented to the ED and the median time from symptoms to ED was 135 min (IQR: 255), from ED to ECG 8 min (IQR: 8), from ECG to thrombolytics 15 min (IQR: 24), and from ECG to balloon 60 min (IQR: 37). The TIT for primary PCI was 220 min (IQR: 250), and the TIT for thrombolytics 300 min (IQR: 400) (Fig 2).

**Fig 3:**
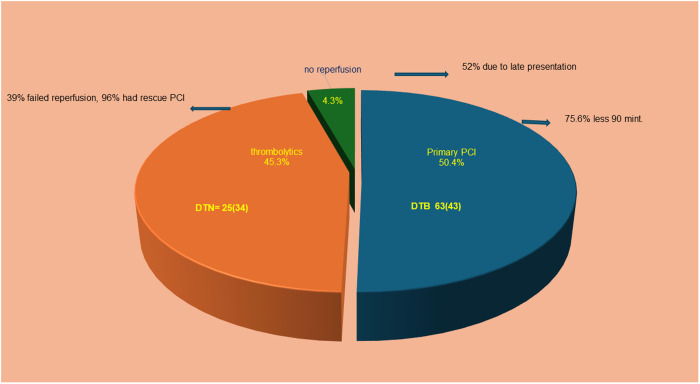
STEMI presented within 24 hrs. DTN: Door To Needle Time. DTB: Door To Balloon Time.

Among the 1206 patients with STEMI who presented within 24 h of symptom onset, primary PCI was performed in 608 (50.4%), thrombolytics were given to 546 (45.3%), and no reperfusion was performed in 52 (4.3%). To elaborate, among patients who presented within 24 h of symptom onset, the door-to-balloon (DTB) time was 63 min (IQR: 43), with 75.6% achieving DTB < 90 min, whereas the door-to-needle (DTN) was 25 min (IQR: 34), with 57% achieving DTN < 60 min. Thirty-nine percent of patients failed thrombolytic reperfusion and 96% of these cases underwent rescue PCI. Of the patients who did not receive reperfusion, 52% were due to late presentation (Fig 3).

At Cath-lab hospitals, DTB time was 63 min (IQR: 44) and DTB < 90 min was achieved in 78% of cases. The DTN time was 35 min (IQR: 34), (58.5%) of those patients failed reperfusion, and all underwent rescue PCI. Seventy-eight (4.5%) patients did not receive reperfusion therapy, mostly due to late presentation (66.7%). Among all PCIs, 77.8% were performed through a radial artery approach (men: 78% vs. women: 76%, p = 0.106), and 18% were treated with aspiration thrombectomy devices.

The median door-to-needle time for men was 26 min (IQR: 33) and for women 20 min (IQR: 31; p = 0.676). However, door-to-needle times <30 min were achieved in 57% of patients, with no significant difference between men and women. The median door-to-balloon time was 63 min (IQR: 43 min); it was 65 min in women (IQR: 59 min) and 63 min in men (IQR: 45 min; p = 0.29). Door-to-balloon times <90 min was achieved in 78% of patients (78% of men vs. 73% of women, p = 0.37).

### In-hospital medications

**Fig 4:**
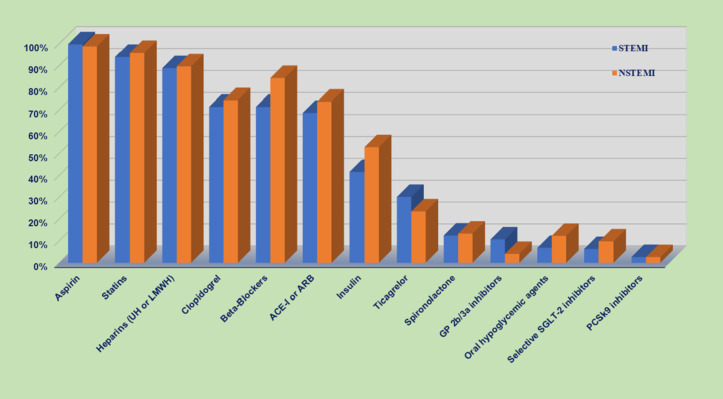
Medication upon the first 24 hours of hospital admission. STEMI: ST-elevation myocardial infarction; NSTEMI: non-ST-elevation myocardial infarction. ACE-I: Angiotensin-Converting Enzyme Inhibitors; ARB: Angiotensin II Receptor Blockers.

**Fig 5:**
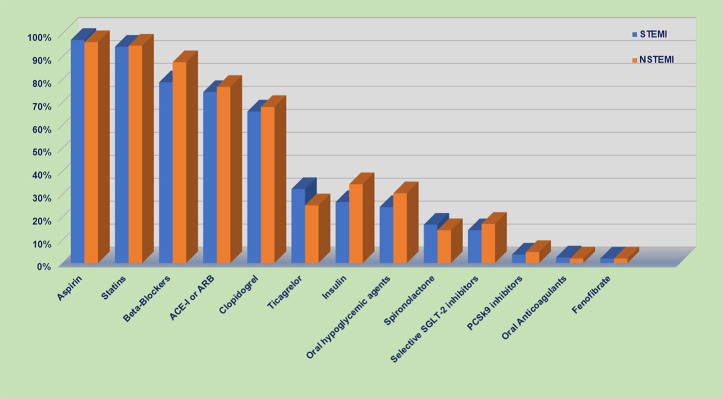
Medication upon discharge. STEMI: ST-elevation myocardial infarction; NSTEMI: non-ST-elevation myocardial infarction. ACE-I: Angiotensin-Converting Enzyme Inhibitors; ARB: Angiotensin II Receptor Blockers.

A high frequency of guideline-recommended treatments was given upon hospital admission (Fig 4). These rates were also high at hospital discharge (Fig 5); 97% of patients received aspirin, 94.5% received statins, 83% received beta-blockers, and 76% received angiotensin-converting enzyme inhibitors (ACE-Is) or angiotensin receptor blockers (ARBs). Most patients received clopidogrel (67%), and fewer received ticagrelor (29%). Compared to patients with NSTEMI, patients with STEMI less frequently received beta-blockers (78.8% vs. 87.6%; p < 0.0001) but more frequently received ticagrelor (32.3% vs. 25.2%; p < 0.0001). A brief comparison between STARS1 vs. STARS2 medication on hospital discharge is shown in (S4 Fig).

### In-hospital procedures and outcomes

Evidence of left ventricular systolic dysfunction was found on echocardiography in 60.3% of all patients. Severe left ventricular systolic dysfunction (defined as < 30% ejection fraction) was found in 8.8% of all patients (Table 1). Seventy-six percent of patients with NSTEMI underwent elective coronary angiography and were more likely to develop atrial fibrillation/flutter or recurrent ischemia compared to patients with STEMI, while patients with STEMI were more likely to develop cardiogenic shock and ventricular tachycardia/fibrillation (Table 2).

**Table 2:** In-hospital course and outcomes of patients with acute myocardial infarction.

Events	Total N = 2,690	STEMI N = 1313(48.8%)	NSTEMI N = 1377(51.1%)	p-value
Recurrent ischemia	363(13.5%)	136(10.4%)	227(16.5)	<.0001
Recurrent myocardial infarction	80(3%)	34(2.6%)	46(3.3%)	0.2517
Atrial fibrillation/flutter	103(3.8%)	33(2.5%)	70(5.1%)	0.0005
Heart failure	322(12)	140(10.7)	182(13.2)	0.0413
Cardiogenic shock	102(3.8)	74(5.6)	28(2)	<.0001
Ventricular tachycardia/fibrillation	103(3.8)	77(5.7)	26(1.9)	<.0001
Stroke	35(1.3)	14(1.1)	21(1.5)	0.2939
Major bleeding	19(0.7)	13(1)	6(0.4)	0.0861
Stent Thrombosis	19(0.7%)	8(0.6%)	11(0.8)	0.5573
Mortality	65(2.4)	37(2.8)	28(2)	0.1853

In-hospital mortality occurred in 2.4% of the patients, with no significant difference between STEMI and NSTEMI (2.81% and 2%, respectively; p = 0.18) and no significant difference between Saudis and non- Saudis (2.4% vs. 2.4%, respectively; p = 0.998; Table 3).

**Table 3:** In-hospital course and outcomes of patients with acute myocardial infarction: A comparison between Saudis vs non-Saudis.

Events	Total N = 2,690	Saudi. N = 1862(69.2%)	Non-Saudi. N = 828(30.8%)	P-value
Recurrent ischemia	363(13.5%)	240(12.9%)	123(14.9%)	0.1684
Recurrent MI	80(3%)	62(3.3%)	18(2.2%)	0.1033
Atrial Fibrillation/Flutter	103(3.8%)	87(4.7%)	16(1.9%)	0.0006
Heart Failure	322(12%)	228(12.2%)	94(11.4%)	0.5105
Cardiogenic Shock	102(3.8%)	64(3.4%)	38(4.6%)	0.1487
VT/VF arrest	103(3.8%)	65(3.5%)	38(4.6%)	0.1705
Stroke	35(1.3%)	29(1.6%)	6(0.7%)	0.0785
Major bleeding	19(0.7%)	14(0.8%)	5(0.6%)	0.6722
Stent thrombosis	19(0.7%)	16(0.9%)	3(0.4%)	0.1554
Mortality	65(2.4%)	45(2.4%)	20(2.4%)	0.9984

VT: Ventricular Tachycardia, VF: Ventricular Fibrillation.

In-hospital mortality in men and women was 2.4% and 2.3%, respectively (p = 0.838). Women had worse in-hospital outcomes with significantly more recurrent ischemia, heart failure, and atrial fibrillation (Table 4). However, the female sex was not a significant predictor when adjusting for confounders. Saudis had more in-hospital atrial fibrillation, which remained significant after adjustment for confounders (OR = 1.98 [95% CI 1.08–3.63], p = 0.028) (S1 Table).

**Table 4:** In-hospital course and outcomes of patients with acute myocardial infarction: A comparison between males and females.

Events	Total N = 2,690	Male N = 2209(82.1%)	Female N = 481(17.9%)	p-value
Recurrent ischemia	363(13.5%)	276(12.5%)	87(18.1%)	0.0011
Recurrent myocardial infarction	80(2.97%)	71(3.2%)	9(1.9%)	0.1161
Atrial fibrillation/flutter	103(3.8)	74(3.4%)	29(6%)	0.0055
Heart failure	322(12%)	248(11.2%)	74(15.4%)	0.0109
Cardiogenic shock	102(3.8%)	84(3.8%)	18(3.8%)	0.9499
Ventricular tachycardia/fibrillation	103(3.8%)	89(4%)	14(2.9%)	0.2467
Stroke	35(1.3%)	29(1.3%)	6(1.25%)	0.9087
Major bleeding	19(0.7%)	13(0.6%)	6(1.3%)	0.1179
Stent Thrombosis	19(0.7%)	19(0.86%)	0	p = 0.035
Mortality	65(2.4)	54(2.4%)	11(2.3%)	P = 0.838

## Discussion

The STARS registry program is the first AMI survey that truly represents the health care system in Saudi Arabia. Unlike prior registries in the region, the STARS program includes all healthcare sectors from all geographic regions of the country. What makes the STARS program different is the snapshot design, which allows the retrieval of high-quality data from consecutive patients with AMI from 50 hospitals, thereby mitigating issues associated with missing data and registry exhaustion. The STARS 2 program is the second phase of this ongoing project that facilitates an assessment of healthcare services in Saudi Arabia every five years. We have shown the following: high prevalence of CAD risk factors, ACS presentation at younger age, low rate of primary PCI, high use of guideline-directed medical therapy (GDMT), and underutilization of EMS services.

The young age at ACS presentation was demonstrated in prior registries in the region [2–4], and this finding remains persistent compared to other registries in the world, despite advances in the healthcare system in Saudi Arabia with time. The mean age in our study was 57 years, compared to 64 years in Northern Europe, and the DM rate in our study was 58%, compared to 26.7% in the STEMI registry of the EURObservational Research Program [9]. This is mainly explained by the high prevalence of CAD risk factors, a sedentary lifestyle, and the high prevalence of obesity [10]. Furthermore, the high prevalence of familial hypercholesterolemia in the Gulf region is approximately 3-fold the estimated prevalence worldwide, demonstrated in the GULF FH Registry and plays a major role in the early presentation of ACS [11]. This highlights the need for region-specific cohort studies aimed at developing customized cardiovascular risk assessment tools. The unique risk profile of the local population may not be adequately represented by existing global models, which could lead to inaccurate predictions and less effective prevention strategies. Tailored tools would ensure more precise risk evaluation and better-informed clinical decision-making. Notably, there is a national transformation initiative in Saudi Arabia (Vision 2030) that includes healthcare sectors with a major focus on primary prevention and infrastructure changes in major cities, including the construction of public parks, such as King Salman Park, and training of primary care physicians [12]. Regarding care in general, we observed high use of GDMT compared to prior registries in the region [2–4], and it is consistent with the rates seen in other registries in the world [9]. For STEMI, we demonstrated a notable improvement in the primary PCI rate (47.1%). In France, a well-developed country, a pharmaco-invasive approach has been adopted in which thrombolytics are followed by rescue PCI in case of failed thrombolytics or elective angiography after successful thrombolytics and it is a reasonable approach, as shown in the FAST AMI study [13]. In a country as vast as Saudi Arabia, the acute revascularization rate is acceptable, particularly in patients who present beyond the recommended timeline for PCI and undergo the pharmaco-invasive approach with rescue PCI for failed thrombolytics or elective angiography for those with successful thrombolytic treatment. Primary PCI rates in the European regions captured in the STEMI registry of the EURObservational Research Program vary between 83.2% (eastern) and 97.4% (western). The centers included in that study were predominantly tertiary care centers; thus, the high rates of primary PCI are understandable [9]. Total ischemic time has been shown to predict mortality in STEMI and has been included in the European STEMI guidelines, with outcomes worsening beyond 120 min [14,15]. More initiatives are required to improve TIT because our times are well beyond the recommended. Over the past few years, there has been a significant expansion in the establishment of cardiac centers, along with the development of various cardiac conferences, such as the Saudi Arabian Cardiovascular Interventional Society (SACIS) and the Gulf Intervention Society (GIS). These advancements have led to a transformative shift in medical education, with many scholarship recipients returning to further strengthen human resources in the field. However, it is crucial to approach direct comparisons with caution. As previously mentioned, demographic differences must be carefully considered, especially when evaluating STARS1 versus STARS2. For NSTEMI, the rates of invasive coronary angiography were 74%, which is higher than previously seen in the first phase of STARS (about half). Nonetheless, the rates are still lower than in high-income countries (91%) [16]. An explanation for this is the potential delays in approving coronary angiography and PCI at catheterization laboratory hospitals. As a result, these patients may only receive medical management and subsequently lose follow-up care. This highlights realistic logistical access to care, which is mainly driven by the various healthcare sectors in Saudi Arabia. The serum troponin test unavailability in many hospitals precluded their enrollment in the study, which has major implications on health care policy and calls for action to make this essential diagnostic test readily available in all hospitals that triage patients with suspected ACS.

While there have been notable improvements in the Saudi Red Crescent initiative, especially during the Hajj season, there remains a significant need for further investment in infrastructure and increased efforts to raise public awareness about utilizing the services of the Red Crescent effectively. Strengthening these aspects will enhance emergency response and ensure greater accessibility for those in need.

When comparing STARS 2 to STARS 1, we found the following. The proportion of Saudi citizens in STARS 2 was 70%, which is higher than the proportion captured in STARS 1 (S3 Table). This partially explains the increase in CAD risk factors, such as DM and HTN. Moreover, the Arab ethnicity among non-Saudis was lower between STARS 1 and STARS 2 (68.7% vs. 32%). Previous publications have demonstrated that non-Saudis predominantly present with STEMI and tend to be younger at the time of AMI presentation [17]. We also noted a decrease in STEMI rate in STARS 2 compared to STARS 1 (48.8% vs. 65.9%), which is likely related to more Cath-lab hospitals in STARS 2 with more Saudi citizens. The primary PCI rate has increased compared to STARS 1 (47.1% vs. 42.5%) and prior registries in the country and region. This is partly related to increased awareness of the Saudi population and improved access to healthcare, along with the number of Cath-lab hospitals and interventional cardiologists providing primary PCI services. Our study also showed improvements in the median door-to-balloon time compared to STARS 1 (63 min [IQR: 43 min] vs. 74 min [IQR: 84 min]). Despite this marked improvement, 24.4% of STEMI patients failed to achieve a door-to-balloon time < 90 min. A brief comparison between STARS1 vs. STARS2 key performance indicators is shown in (S5 and S6 Figs). Further improvement in primary PCI rates with door-to-balloon times < 90 min is crucial. The Western Denmark Heart Registry showed, in PCI-treated STEMI patients who survived the first 90 days, absolute excess mortality of only 2.1% compared to matched individuals of the general population at the 10-year follow-up (26.5% vs 24.5%; HR: 1.04; 95% CI: 1.01–1.08) [18]. Furthermore, in the present study, EMS was utilized in only 8.5% of cases, with marginal improvement compared to STARS 1 (5.2%). To our knowledge, the STARS program is the first to investigate and track the rate of radial access in AMI patients over time (76%). This rate has improved compared to STARS 1 (48.2%). Interestingly, this improvement was also observed among women (68% vs. 29.3% in STARS 1). This rate is comparable to radial access rates in Europe, ranging from 47.9% in Western Europe to 72% in Southern Europe [9]. Furthermore, the radial access group had fewer net adverse clinical events (15.2%) compared to the femoral access group (17.2%; RR = 0.87, 95% CI: 0.78–0.97; p = 0.0128), which was also shown in the Minimizing Adverse Hemorrhagic Events by Trans radial Access Site and Systemic Implementation of Angiox (MATRIX) program [19].

Our study had some limitations. First, hospital enrollment was voluntary with the possibility of under-representing patients in real-life clinical practice. However, the recruitment of various healthcare sectors from all regions of the country helped mitigate this limitation. Second, selection bias could have been introduced with the possibility of missing important unmeasured variables due to the observational nature of registry studies. Third, only 12% of the 416 eligible hospitals were included in our study. This may indicate a relatively small sample size. Nonetheless, the number of patients recruited from Cath-lab hospitals was the majority in our study (1650, 66%). These healthcare centers have a large catchment area and receive referrals from many surrounding hospitals in the respective region, so we believe that the actual representation is much more than 12%. Fourth, a direct comparison between STARS 1 and STARS 2 may not be scientifically valid because many confounding factors and variables are not detected, such as different house staff and different populations (e.g., more Saudis in STARS 2 compared to STARS 1).

In conclusion, this is the second phase of the STARS program with a unique study design. It provides a snapshot of the AMI care in Saudi Arabia. This design will provide potential help to other countries with large geographic areas. In Saudi Arabia, patients present with AMI at a relatively young age have a high prevalence of CAD risk factors. Although we demonstrated improvement in the delivery of primary PCI benchmarks including a noticeable increase in radial access, further improvement is needed in terms of acute revascularization for STEMI and NSTEMI. While EMS use has increased, enhancing its infrastructure and utilization remains essential. Additionally, improving public awareness of EMS services and expanding Cath-lab availability will further strengthen emergency cardiac care. Establishing ACS programs and networks will also enhance overall healthcare access.

## Supporting information

S1 File Summary of study methodology. (DOCX)

S1 Fig Enrolled hospitals vs Total eligible hospitals. (TIF)

S2 Fig Enrolled hospitals and CRF from the Ministry of Health (MOH). (TIF)

S3 Fig Cath-lab vs non-Cath-lab hospitals. (TIF)

S4 Fig Meds at Discharge: STARS-2 Vs. STARS-1. (JPG)

S5 Fig Comparison of Key Performance Indicators in Acute STEMI Revascularization: STARS-2 Vs. STARS-1. (JPG)

S6 Fig Total Ischemic Time (TIT): STARS-2 Vs. STARS-1. (JPG)

S1 Table Adjusted Odds Ratio for Female Gender in Logistic Regression. (DOCX)

S2 Table The start and end of the recruitment period for each study site. (DOCX)

S3 Table Comparison of baseline characteristics between STARS-1 and STARS-2. (DOCX)

S1 Data Raw data. (ZIP)
