# Supplementary material for: The second survey of the Saudi Acute Myocardial Infarction Registry Program: Main results and temporal changes in care (STARS-2 program)
Source: PLoS One. 2025 Sep 2;20(9):e0331215. doi: 10.1371/journal.pone.0331215 (PMC12404464; doi:10.1371/journal.pone.0331215)
Supplement: S1 File — (DOCX) [file pone.0331215.s001.docx]

**S1 File. Summary of study methodology**

To ensure that the study accurately represents the real-life distribution of healthcare in Saudi Arabia, several key measures were implemented:

1. Geographical Representation – One of the primary objectives of the study was to ensure broad regional inclusivity. To achieve this, participants were recruited from all 13 provinces across Saudi Arabia. This approach allowed for comprehensive coverage of urban and rural areas, ensuring that variations in healthcare access, quality, and patient demographics were properly accounted for in the study. By including a diverse geographical sample, we minimized potential biases related to healthcare availability in certain regions.
2. Balanced Hospital Recruitment – A critical factor in maintaining the integrity of the study was ensuring an equitable distribution of participants between cath lab and non-cath lab hospitals. By carefully structuring the recruitment process, we prevented overrepresentation from either hospital type. This was essential for ensuring that results were not skewed toward one particular healthcare setting, thus allowing the findings to more accurately reflect the broader healthcare system. A disproportionate sampling from cath labs could have led to biased conclusions, while under-recruitment from non-cath lab hospitals would have failed to capture key aspects of real-world healthcare practice.
3. Healthcare Sector Inclusion – The study design also focused on incorporating participants from all seven major healthcare sectors to provide a comprehensive analysis of the healthcare landscape in Saudi Arabia. This included representation from government hospitals, military healthcare facilities, private hospitals, and other specialized medical institutions. Specifically, the sample was distributed as follows:
   - 60% of participants were recruited from Ministry of Health hospitals, which form the backbone of Saudi Arabia’s healthcare system. These hospitals serve a large portion of the population and provide critical public healthcare services.
   - 20% of participants came from military healthcare facilities, which cater to service members and their families. These hospitals often feature specialized cardiac care and advanced medical infrastructure.
   - The remaining portion of the study population was drawn from private hospitals, which play a significant role in providing high-quality care, often with access to advanced technologies and specialized treatments.

This distribution closely mirrors the actual healthcare structure in the country, ensuring that the study accurately represents the different healthcare settings available to the population.

1. Recruitment Caps and Patient Allocation – To further refine the study’s sampling methodology, specific recruitment caps were imposed to maintain balance across different hospital types.

- Cath lab hospitals were required to enroll 60 consecutive patients, ensuring consistency in recruitment and avoiding selection biases that could distort results.
- Non-cath lab hospitals, on the other hand, were mandated to enroll at least 25 patients, ensuring sufficient representation while accommodating the potential limitations in patient volume at these facilities.

By implementing these structured recruitment quotas, we effectively mitigated the risk of over- or under-recruitment in different hospital categories. This method ensured that the study population was not overly skewed toward high-volume treatment centers while still providing adequate representation from smaller or less specialized healthcare institutions.
