# Supplementary figures and images for: The second survey of the Saudi Acute Myocardial Infarction Registry Program: Main results and temporal changes in care (STARS-2 program)
Source: PLoS One. 2025 Sep 2;20(9):e0331215. doi: 10.1371/journal.pone.0331215 (PMC12404464; doi:10.1371/journal.pone.0331215)

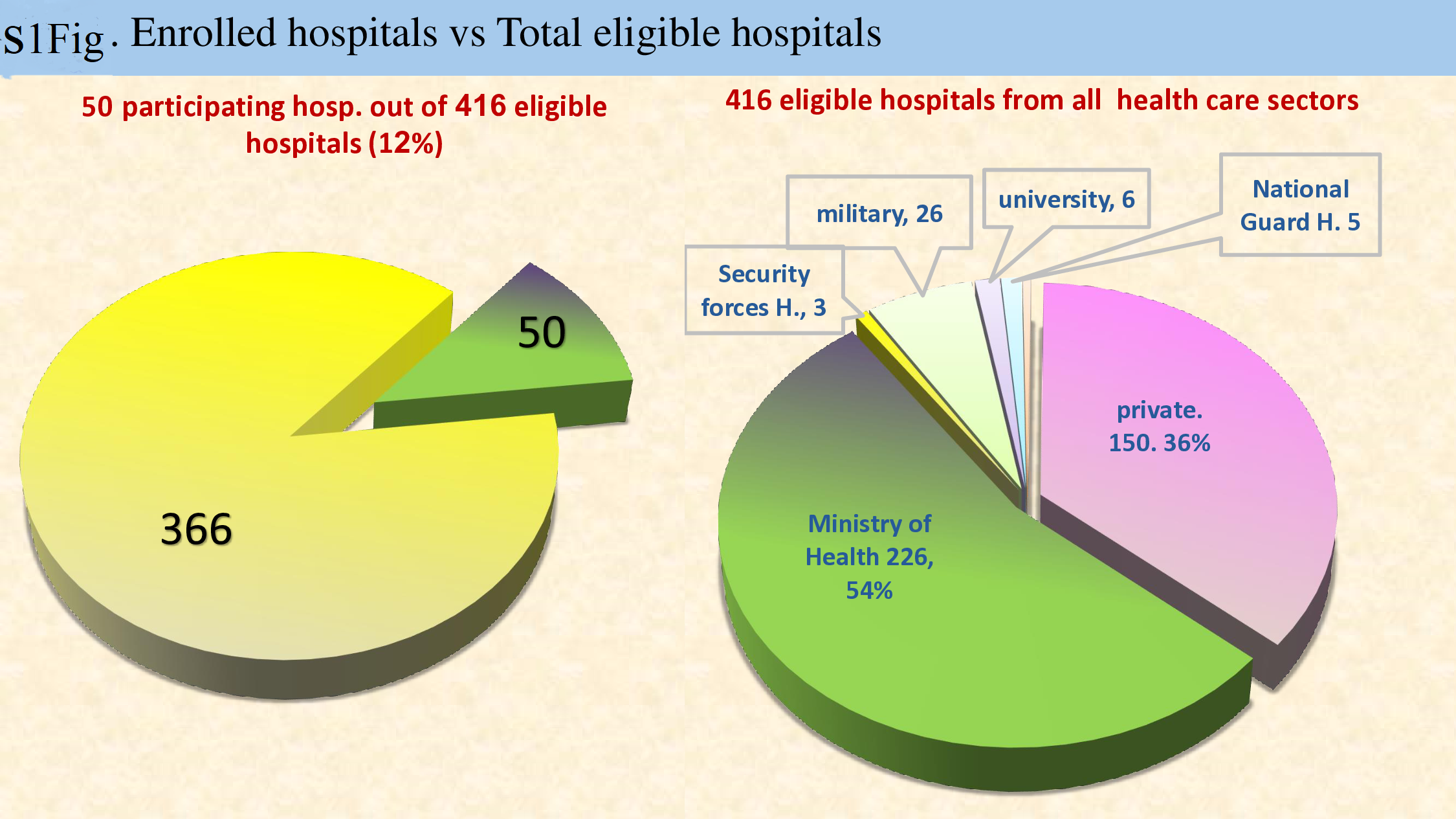

Supplement: S1 Fig — (TIF) [file pone.0331215.s002.tif]

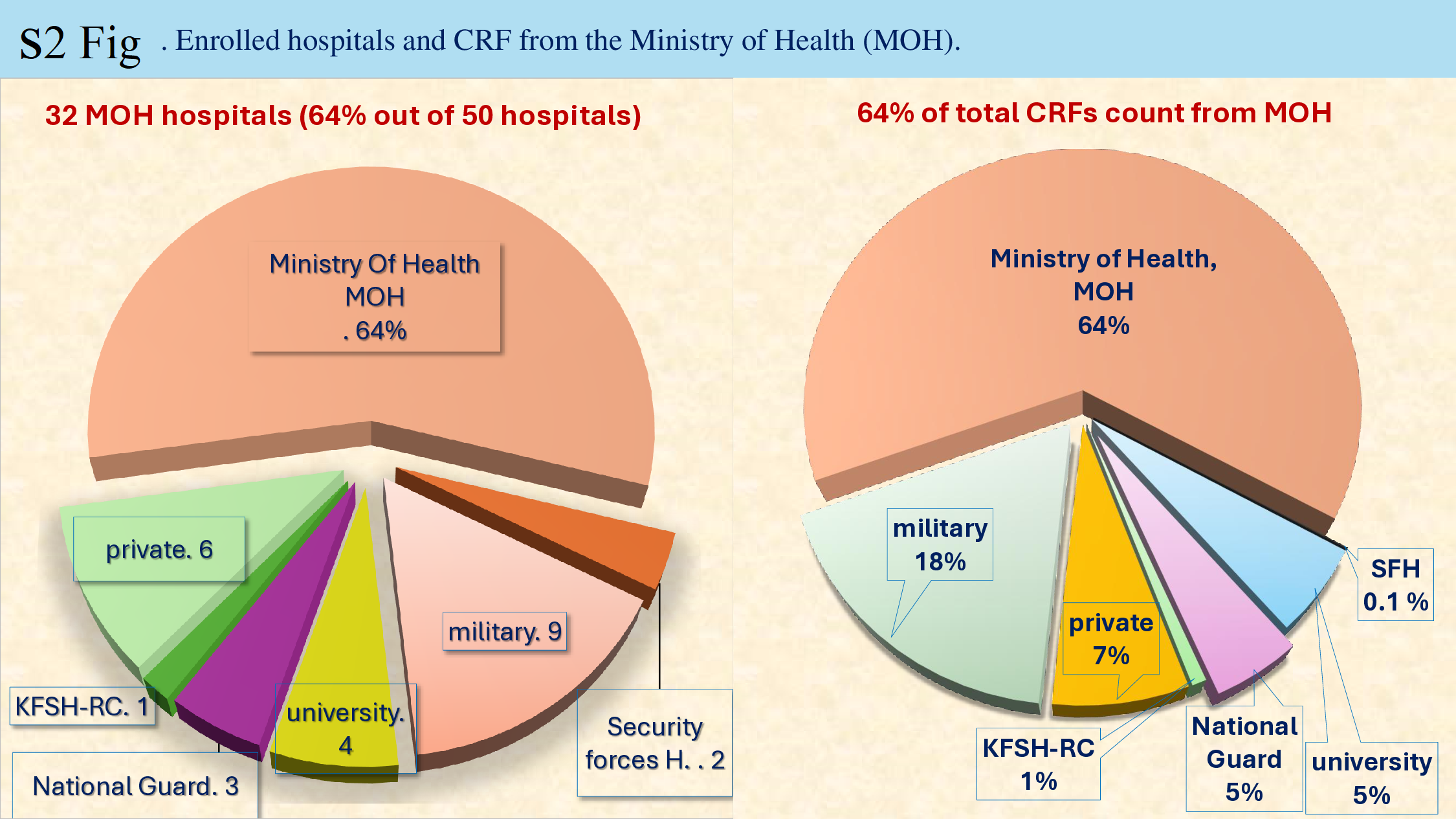

Supplement: S2 Fig — (TIF) [file pone.0331215.s003.tif]

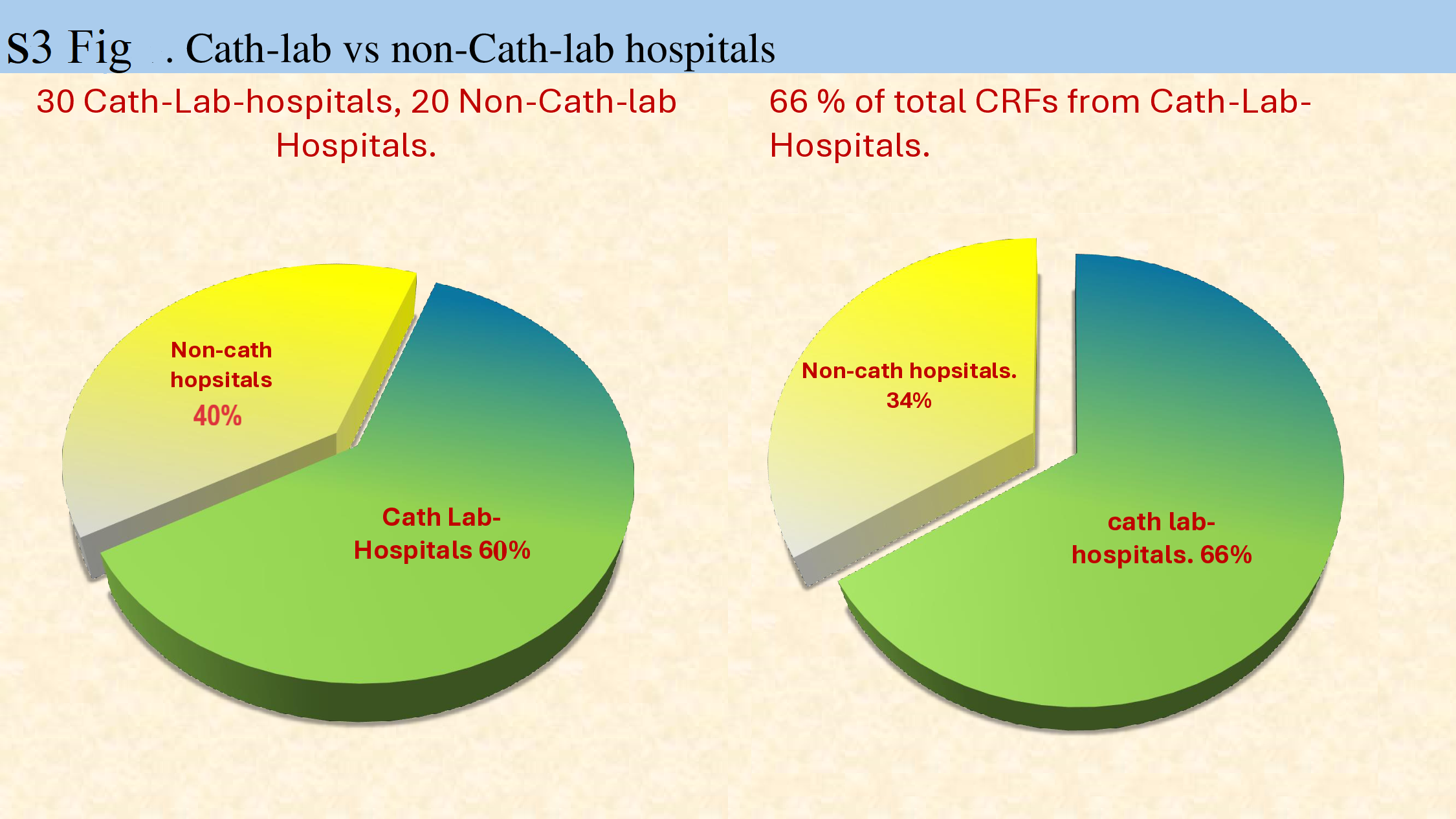

Supplement: S3 Fig — (TIF) [file pone.0331215.s004.tif]

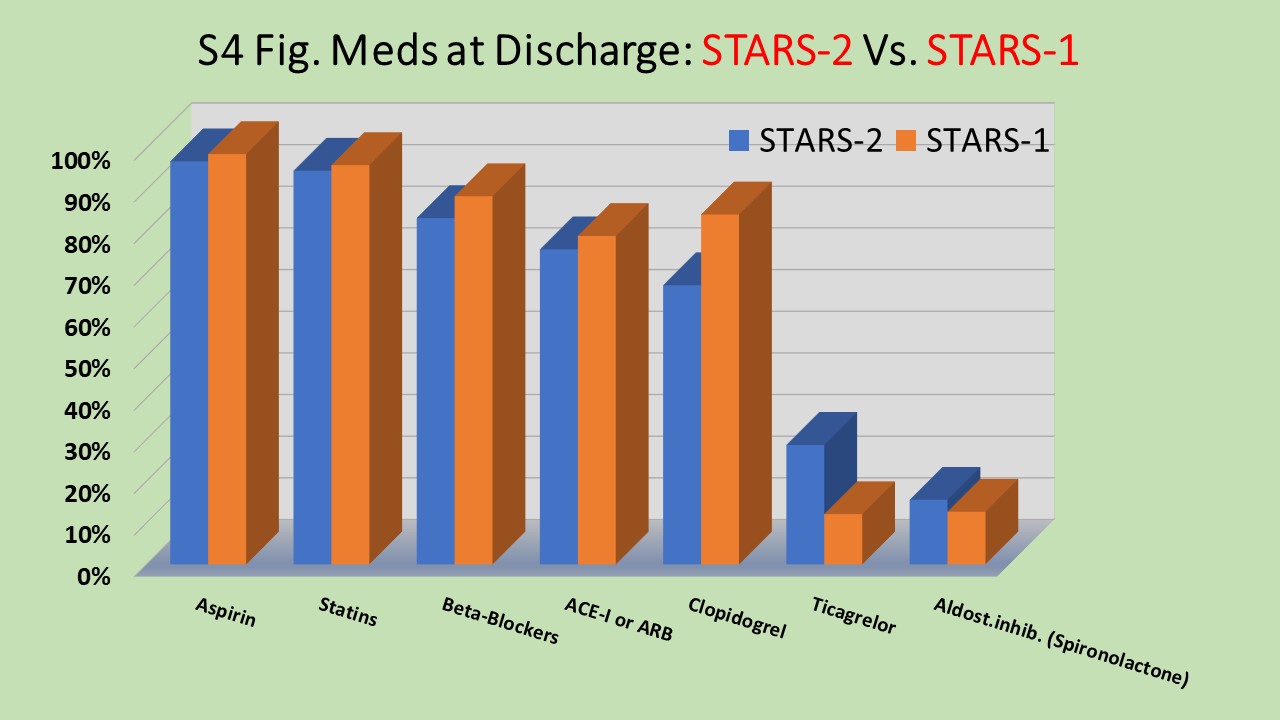

Supplement: S4 Fig — (JPG) [file pone.0331215.s005.jpg]

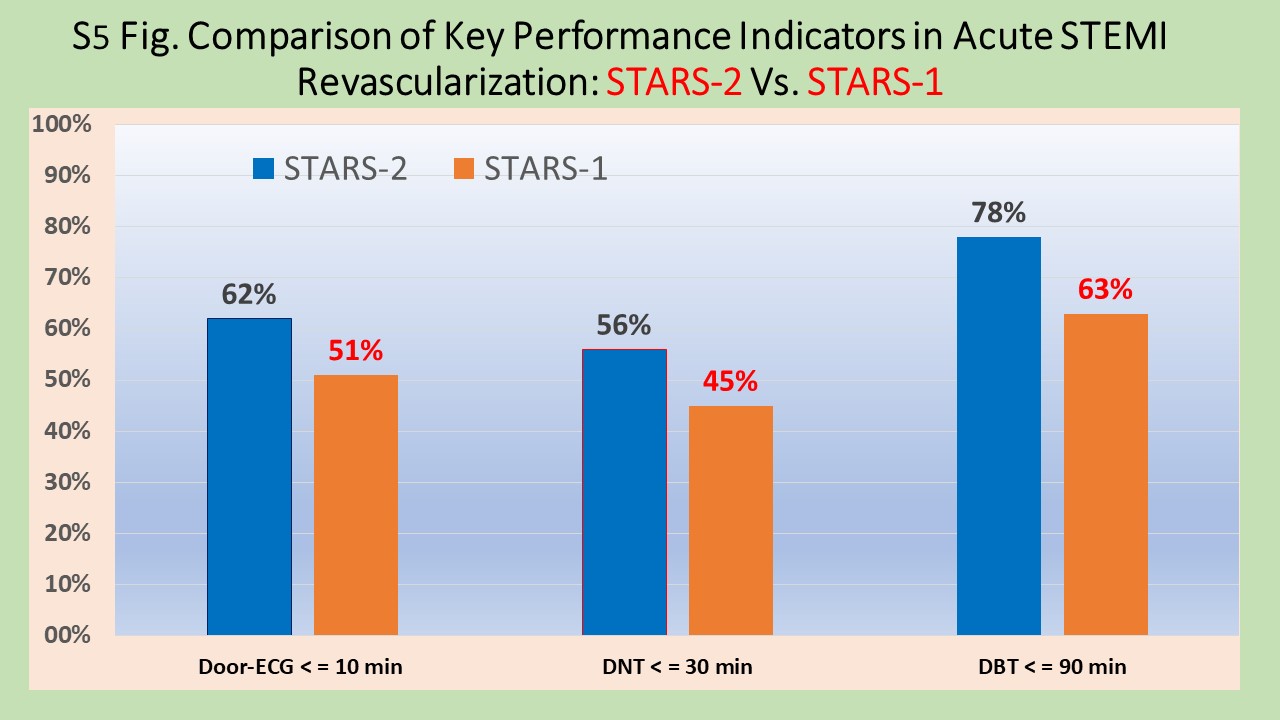

Supplement: S5 Fig — (JPG) [file pone.0331215.s006.jpg]

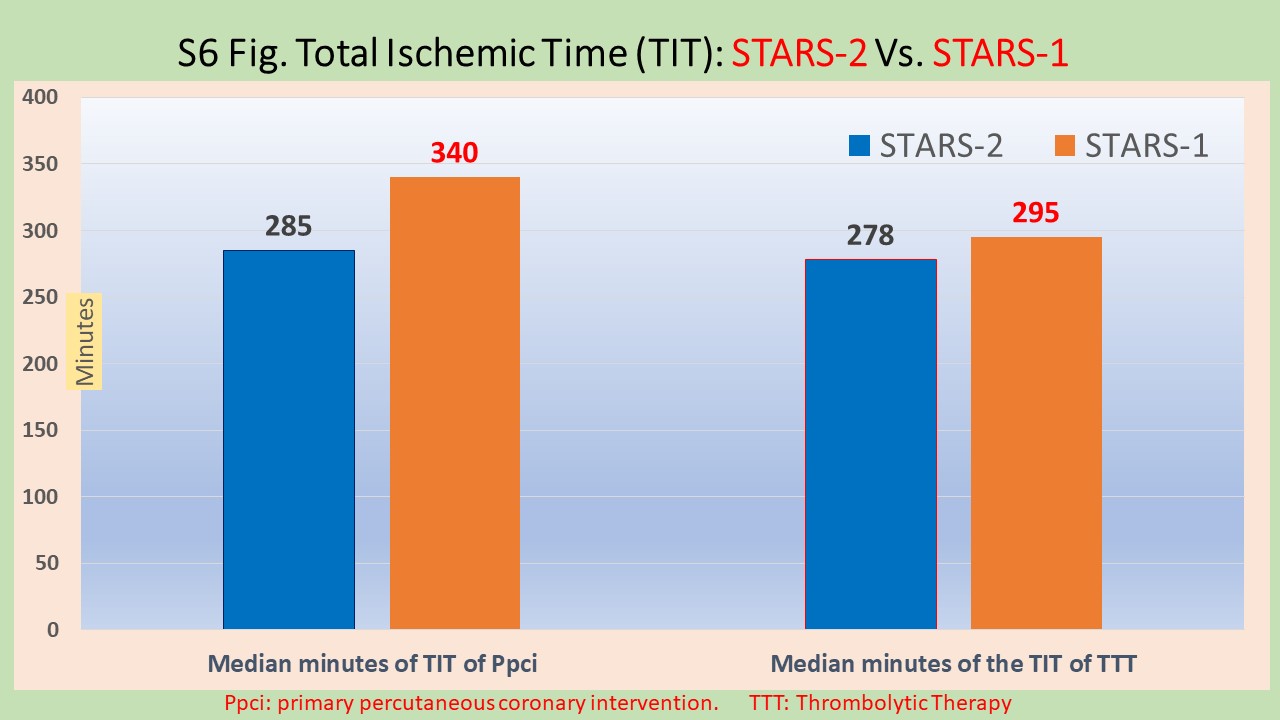

Supplement: S6 Fig — (JPG) [file pone.0331215.s007.jpg]
