# Supplementary material for: The second survey of the Saudi Acute Myocardial Infarction Registry Program: Main results and temporal changes in care (STARS-2 program)
Source: PLoS One. 2025 Sep 2;20(9):e0331215. doi: 10.1371/journal.pone.0331215 (PMC12404464; doi:10.1371/journal.pone.0331215)
Supplement: S1 Table — (DOCX) [file pone.0331215.s008.docx]

**S1 Table.** **Adjusted Odds Ratio for Female Gender in Logistic Regression.**

| **Covariate** | **Level** | **Crude OR(95% CI)** | **P-value** | **Adjusted OR (95% CI)** | **P-value** |
| --- | --- | --- | --- | --- | --- |
| Recurrent Ischemia | Female | 1.55 (1.19,2.01) | 0.001 | 1.32 (0.96,1.8) | 0.083 |
| Heart Failure | Female | 1.44 (1.09,1.9) | 0.011 | 1.21 (0.55,2.66) | 0.632 |
| Atrial Fibrillation/Flutter | Female | 1.89 (1.19,2.88) | 0.006 | 1.08 (0.67,1.77) | 0.747 |

Female had worse in-hospital outcomes with significantly more recurrent ischemia, heart failure and atrial fibrillation, however, female gender was not significant when adjusted for confounders.

Saudis had more in-hospital atrial fibrillation which remained significant after adjustment for confounders (OR= 1.98 [95% 1.08 – 3.63]), p=0.028
