# Supplementary material for: The second survey of the Saudi Acute Myocardial Infarction Registry Program: Main results and temporal changes in care (STARS-2 program)
Source: PLoS One. 2025 Sep 2;20(9):e0331215. doi: 10.1371/journal.pone.0331215 (PMC12404464; doi:10.1371/journal.pone.0331215)
Supplement: S2 Table — (DOCX) [file pone.0331215.s009.docx]

**S2 Table. The start and end of the recruitment period for each study site.**

| user | start date | end date |
| --- | --- | --- |
| STARS2-01 | 03/09/2021 | 24/10/2021 |
| STARS2-02 | 17/09/2022 | 12/11/2022 |
| STARS2-03 | 05/10/2021 | 25/10/2021 |
| STARS2-04 | 12/10/2021 | 15/01/2022 |
| STARS2-05 | 19/09/2021 | 12/11/2021 |
| STARS2-08 | 14/11/2021 | 27/04/2022 |
| STARS2-09 | 08/09/2021 | 05/12/2021 |
| STARS2-10 | 29/11/2021 | 23/12/2021 |
| STARS2-11 | 03/03/2022 | 20/07/2022 |
| STARS2-13 | 16/11/2021 | 08/01/2022 |
| STARS2-14 | 08/09/2021 | 16/01/2022 |
| STARS2-15 | 29/10/2021 | 01/12/2021 |
| STARS2-16 | 27/09/2021 | 30/11/2021 |
| STARS2-17 | 19/05/2022 | 24/08/2022 |
| STARS2-18 | 01/06/2022 | 24/09/2022 |
| STARS2-19 | 03/03/2022 | 12/06/2022 |
| STARS2-21 | 08/06/2022 | 27/09/2022 |
| STARS2-22 | 05/11/2021 | 30/12/2021 |
| STARS2-23 | 07/09/2021 | 06/01/2022 |
| STARS2-24 | 08/03/2022 | 21/07/2022 |
| STARS2-27 | 29/11/2021 | 29/12/2021 |
| STARS2-28 | 17/10/2022 | 07/11/2022 |
| STARS2-29 | 26/09/2021 | 07/01/2022 |
| STARS2-30 | 30/05/2022 | 21/09/2022 |
| STARS2-32 | 28/11/2021 | 11/01/2022 |
| STARS2-34 | 12/10/2022 | 06/01/2023 |
| STARS2-36 | 20/09/2021 | 13/12/2021 |
| STARS2-37 | 07/04/2022 | 28/07/2022 |
| STARS2-39 | 06/10/2021 | 08/12/2021 |
| STARS2-40 | 20/07/2022 | 21/11/2022 |
| STARS2-41 | 26/09/2021 | 02/02/2022 |
| STARS2-43 | 12/11/2021 | 24/03/2022 |
| STARS2-46 | 05/09/2021 | 07/12/2021 |
| STARS2-47 | 08/09/2021 | 11/01/2022 |
| STARS2-48 | 08/09/2021 | 04/01/2022 |
| STARS2-50 | 02/02/2022 | 25/07/2022 |
| STARS2-52 | 12/10/2021 | 09/01/2022 |
| STARS2-54 | 16/08/2022 | 22/12/2022 |
| STARS2-55 | 08/10/2021 | 12/01/2022 |
| STARS2-57 | 08/10/2021 | 14/12/2021 |
| STARS2-58 | 05/06/2022 | 14/09/2022 |
| STARS2-59 | 17/09/2021 | 22/12/2021 |
| STARS2-60 | 07/10/2021 | 06/02/2022 |
| STARS2-62 | 25/05/2022 | 29/09/2022 |
| STARS2-63 | 07/02/2022 | 26/06/2022 |
| STARS2-64 | 06/03/2022 | 01/06/2022 |
| STARS2-65 | 08/02/2022 | 06/06/2022 |
| STARS2-66 | 06/07/2022 | 17/09/2022 |
| STARS2-67 | 05/03/2022 | 11/07/2022 |
| STARS2-68 | 21/03/2022 | 14/09/2022 |
