# Supplementary material for: The second survey of the Saudi Acute Myocardial Infarction Registry Program: Main results and temporal changes in care (STARS-2 program)
Source: PLoS One. 2025 Sep 2;20(9):e0331215. doi: 10.1371/journal.pone.0331215 (PMC12404464; doi:10.1371/journal.pone.0331215)
Supplement: S3 Table — (DOCX) [file pone.0331215.s010.docx]

**S3 Table: Comparison of baseline characteristics between STARS-1 and STARS-2.**

|  | **STARS1** | **STARS2** |
| --- | --- | --- |
| **Duration** | May 2015 and January 2017 | Sep. 2021 and Jan. 2023 |
| **Total population** | 2233 | 2690 |
| **Total STEMI** | 1471 (65.9%) | 1313 (48.8%) |
| **Total NSTEMI** | 762 (34.1%) | 1377 (51.1%) |
| **Total hospitals** | 370 | 416 |
| **No Cath hospitals** | 30 centers were non-Cath Lab hospitals (60%) | 20 centers non-Cath hospitals (39%) |
| **Mean age** | 56 (±13) years | 57 (±12.4) years |
| **Saudi citizens** | 55.6% | 70% |
| **Men** | 85.7% | 82% |
| **Arab ethnicity among non-Saudis** | 68.7% | 78% |
| **DM** | 52.7% o | 58% |
| **HTN** | 51.2% | 59% |
| **Smoking** | 51.3% | 43% |
| **BMI** | 28.43 (±5.4) kg/m2 | 28.5 (±4.7) kg/m^2^ |
| **EMS transfer at presentation** | 5.2% | 8.5% |
| **Cp at presentation** | 88.3% | 87% |
| **HF at presentation** | 16% | 12% |
