# Supplementary material for: The second survey of the Saudi Acute Myocardial Infarction Registry Program: Main results and temporal changes in care (STARS-2 program)
Source: PLoS One. 2025 Sep 2;20(9):e0331215. doi: 10.1371/journal.pone.0331215 (PMC12404464; doi:10.1371/journal.pone.0331215)
Supplement: S1 Data — (ZIP) [file pone.0331215.s011.zip › Raw data/AF and stent thrombosis.pdf]

The FREQ Procedure

Frequency<br>Percent<br>Row Pct<br>Col Pct

Table of STEMI_NSTEMI by Atrial_Fibrillation_Flutter			
	Atrial_Fibrillation_Flutter(Atrial Fibrillation/Flutter)		
STEMI_NSTEMI(STEMI/NSTEMI)	1	2	Total
1	33<br>1.23<br>2.51<br>32.04	1280<br>47.58<br>97.49<br>49.48	1313<br>48.81
2	70<br>2.60<br>5.08<br>67.96	1307<br>48.59<br>94.92<br>50.52	1377<br>51.19
Total	103<br>3.83	2587<br>96.17	2690<br>100.00
Frequency Missing = 1			

Statistics for Table of STEMI_NSTEMI by Atrial_Fibrillation_Flutter

Statistic	DF	Value	Prob
Chi-Square	1	12.0572	0.0005
Likelihood Ratio Chi-Square	1	12.3520	0.0004
Continuity Adj. Chi-Square	1	11.3693	0.0007
Mantel-Haenszel Chi-Square	1	12.0527	0.0005
Phi Coefficient		-0.0669	
Contingency Coefficient		0.0668	
Cramer's V		-0.0669	

Fisher's Exact Test	
Cell (1,1) Frequency (F)	33
Left-sided Pr <= F	0.0003
Right-sided Pr >= F	0.9998
Table Probability (P)	0.0002
Two-sided Pr <= P	0.0006

Sample Size = 2690
Frequency Missing = 1

AF and Stent thrombosis

The FREQ Procedure

Frequency<br>Percent<br>Row Pct<br>Col Pct

Table of STEMI_NSTEMI by Stent_thrombosis			
	Stent_thrombosis(Stent thrombosis)		
STEMI_NSTEMI(STEMI/NSTEMI)	1	2	Total
1	8<br>0.30<br>0.61<br>42.11	1305<br>48.51<br>99.39<br>48.86	1313<br>48.81
2	11<br>0.41<br>0.80<br>57.89	1366<br>50.78<br>99.20<br>51.14	1377<br>51.19
Total	19<br>0.71	2671<br>99.29	2690<br>100.00
Frequency Missing = 1			

Statistics for Table of STEMI_NSTEMI by Stent_thrombosis

Statistic	DF	Value	Prob
Chi-Square	1	0.3443	0.5573
Likelihood Ratio Chi-Square	1	0.3461	0.5563
Continuity Adj. Chi-Square	1	0.1271	0.7215
Mantel-Haenszel Chi-Square	1	0.3442	0.5574
Phi Coefficient		-0.0113	
Contingency Coefficient		0.0113	
Cramer's V		-0.0113	

Fisher's Exact Test	
Cell (1,1) Frequency (F)	8
Left-sided Pr <= F	0.3619
Right-sided Pr >= F	0.7925
Table Probability (P)	0.1544
Two-sided Pr <= P	0.6482

Sample Size = 2690
Frequency Missing = 1
