# Supplementary material for: The second survey of the Saudi Acute Myocardial Infarction Registry Program: Main results and temporal changes in care (STARS-2 program)
Source: PLoS One. 2025 Sep 2;20(9):e0331215. doi: 10.1371/journal.pone.0331215 (PMC12404464; doi:10.1371/journal.pone.0331215)
Supplement: S1 Data — (ZIP) [file pone.0331215.s011.zip › Raw data/HR and SBP.pdf]

HR=1 fast, SBP=1 low

The FREQ Procedure

Frequency<br>Percent<br>Row Pct<br>Col Pct

Table of STEMI_NSTEMI by HR			
	HR		
STEMI_NSTEMI(STEMI/NSTEMI)	0	1	Total
1	1138<br>42.30<br>86.67<br>48.74	175<br>6.51<br>13.33<br>49.30	1313<br>48.81
2	1197<br>44.50<br>86.93<br>51.26	180<br>6.69<br>13.07<br>50.70	1377<br>51.19
Total	2335<br>86.80	355<br>13.20	2690<br>100.00
Frequency Missing = 1			

Statistics for Table of STEMI_NSTEMI by HR

Statistic	DF	Value	Prob
Chi-Square	1	0.0386	0.8443
Likelihood Ratio Chi-Square	1	0.0386	0.8443
Continuity Adj. Chi-Square	1	0.0194	0.8891
Mantel-Haenszel Chi-Square	1	0.0385	0.8444
Phi Coefficient		-0.0038	
Contingency Coefficient		0.0038	
Cramer's V		-0.0038	

Fisher's Exact Test	
Cell (1,1) Frequency (F)	1138
Left-sided Pr <= F	0.4445
Right-sided Pr >= F	0.6001
Table Probability (P)	0.0446
Two-sided Pr <= P	0.8644

Sample Size = 2690
Frequency Missing = 1

The FREQ Procedure

Frequency<br>Percent<br>Row Pct<br>Col Pct

Table of STEMI_NSTEMI by SBP			
	SBP		
STEMI_NSTEMI(STEMI/NSTEMI)	0	1	Total
1	1264<br>46.99<br>96.27<br>48.21	49<br>1.82<br>3.73<br>72.06	1313<br>48.81
2	1358<br>50.48<br>98.62<br>51.79	19<br>0.71<br>1.38<br>27.94	1377<br>51.19
Total	2622<br>97.47	68<br>2.53	2690<br>100.00
Frequency Missing = 1			

Statistics for Table of STEMI_NSTEMI by SBP

Statistic	DF	Value	Prob
Chi-Square	1	15.0911	0.0001
Likelihood Ratio Chi-Square	1	15.5499	<.0001
Continuity Adj. Chi-Square	1	14.1516	0.0002
Mantel-Haenszel Chi-Square	1	15.0855	0.0001
Phi Coefficient		-0.0749	
Contingency Coefficient		0.0747	
Cramer's V		-0.0749	

Fisher's Exact Test	
Cell (1,1) Frequency (F)	1264
Left-sided Pr <= F	<.0001
Right-sided Pr >= F	1.0000
Table Probability (P)	<.0001
Two-sided Pr <= P	0.0001

Sample Size = 2690
Frequency Missing = 1
