# Supplementary material for: The second survey of the Saudi Acute Myocardial Infarction Registry Program: Main results and temporal changes in care (STARS-2 program)
Source: PLoS One. 2025 Sep 2;20(9):e0331215. doi: 10.1371/journal.pone.0331215 (PMC12404464; doi:10.1371/journal.pone.0331215)
Supplement: S1 Data — (ZIP) [file pone.0331215.s011.zip › Raw data/logistic for supp tables (gender) SEE word table.pdf]

# The FREQ Procedure

| Frequency<br>Percent<br>Row Pct<br>Col Pct |
|---|

Table of CHF_Killip_Class by Echo_Options

| CHF_Killip_Class(CHF Killip Class) | Echo_Options(Echo-Options) 1 | 2 | 3 | 4 | Total |
|---|---|---|---|---|---|
| 1 | 921<br>38.62<br>44.24<br>97.25 | 682<br>28.60<br>32.76<br>88.69 | 351<br>14.72<br>16.86<br>76.30 | 128<br>5.37<br>6.15<br>61.24 | 2082<br>87.30 |
| 2 | 15<br>0.63<br>8.38<br>1.58 | 61<br>2.56<br>34.08<br>7.93 | 67<br>2.81<br>37.43<br>14.57 | 36<br>1.51<br>20.11<br>17.22 | 179<br>7.51 |
| 3 | 9<br>0.38<br>9.89<br>0.95 | 20<br>0.84<br>21.98<br>2.60 | 36<br>1.51<br>39.56<br>7.83 | 26<br>1.09<br>28.57<br>12.44 | 91<br>3.82 |
| 4 | 2<br>0.08<br>6.06<br>0.21 | 6<br>0.25<br>18.18<br>0.78 | 6<br>0.25<br>18.18<br>1.30 | 19<br>0.80<br>57.58<br>9.09 | 33<br>1.38 |
| Total | 947<br>39.71 | 769<br>32.24 | 460<br>19.29 | 209<br>8.76 | 2385<br>100.00 |
| Frequency Missing = 306 | | | | | |

## Statistics for Table of CHF_Killip_Class by Echo_Options

| Statistic | DF | Value | Prob |
|---|---|---|---|
| Chi-Square | 9 | 319.4938 | <.0001 |
| Likelihood Ratio Chi-Square | 9 | 273.3622 | <.0001 |
| Mantel-Haenszel Chi-Square | 1 | 251.3404 | <.0001 |
| Phi Coefficient | | 0.3660 | |
| Contingency Coefficient | | 0.3437 | |
| Cramer's V | | 0.2113 | |

**Sample Size = 2385**
**Frequency Missing = 306**

**WARNING: 11% of the data are missing.**

## The FREQ Procedure

| Frequency<br>Percent<br>Row Pct<br>Col Pct |
|---|

| Table of History_of_angina by History_of_MI | | | |
|---|---|---|---|
| History_of_angina(History of angina) | History_of_MI(History of MI) | | |
| | 1 | 2 | Total |
| 1 | 311<br>11.56<br>42.03<br>77.17 | 429<br>15.95<br>57.97<br>18.76 | 740<br>27.51 |
| 2 | 92<br>3.42<br>4.72<br>22.83 | 1858<br>69.07<br>95.28<br>81.24 | 1950<br>72.49 |
| Total | 403<br>14.98 | 2287<br>85.02 | 2690<br>100.00 |
| Frequency Missing = 1 | | | |

## Statistics for Table of History_of_angina by History_of_MI

| Statistic | DF | Value | Prob |
|---|---|---|---|
| Chi-Square | 1 | 586.2413 | <.0001 |
| Likelihood Ratio Chi-Square | 1 | 523.9905 | <.0001 |
| Continuity Adj. Chi-Square | 1 | 583.3158 | <.0001 |
| Mantel-Haenszel Chi-Square | 1 | 586.0234 | <.0001 |
| Phi Coefficient | | 0.4668 | |
| Contingency Coefficient | | 0.4230 | |
| Cramer's V | | 0.4668 | |

| Fisher's Exact Test | |
|---|---|
| Cell (1,1) Frequency (F) | 311 |
| Left-sided Pr <= F | 1.0000 |
| Right-sided Pr >= F | <.0001 |
| | |
| Table Probability (P) | <.0001 |
| Two-sided Pr <= P | <.0001 |

**Sample Size = 2690**
**Frequency Missing = 1**

## The FREQ Procedure

| Frequency<br>Percent<br>Row Pct<br>Col Pct |
|---|

| Table of History_of_angina by History_of_CABG | | | |
|---|---|---|---|
| History_of_angina(History of angina) | History_of_CABG(History of CABG) | | |
| | 1 | 2 | Total |
| 1 | 56<br>2.08<br>7.57<br>78.87 | 684<br>25.43<br>92.43<br>26.12 | 740<br>27.51 |
| 2 | 15<br>0.56<br>0.77<br>21.13 | 1935<br>71.93<br>99.23<br>73.88 | 1950<br>72.49 |
| Total | 71<br>2.64 | 2619<br>97.36 | 2690<br>100.00 |
| Frequency Missing = 1 | | | |

## Statistics for Table of History_of_angina by History_of_CABG

| Statistic | DF | Value | Prob |
|---|---|---|---|
| Chi-Square | 1 | 96.4784 | <.0001 |
| Likelihood Ratio Chi-Square | 1 | 83.5581 | <.0001 |
| Continuity Adj. Chi-Square | 1 | 93.8510 | <.0001 |
| Mantel-Haenszel Chi-Square | 1 | 96.4425 | <.0001 |
| Phi Coefficient | | 0.1894 | |
| Contingency Coefficient | | 0.1861 | |
| Cramer's V | | 0.1894 | |

| Fisher's Exact Test | |
|---|---|
| Cell (1,1) Frequency (F) | 56 |
| Left-sided Pr <= F | 1.0000 |
| Right-sided Pr >= F | <.0001 |
| | |
| Table Probability (P) | <.0001 |
| Two-sided Pr <= P | <.0001 |

**Sample Size = 2690**
**Frequency Missing = 1**

## The FREQ Procedure

| Frequency<br>Percent<br>Row Pct<br>Col Pct |
|---|

Table of CAD by History_of_angina

| CAD | History_of_angina(History of angina) 1 | 2 | Total |
|---|---|---|---|
| 1 | 740<br>27.51<br>81.23<br>100.00 | 171<br>6.36<br>18.77<br>8.77 | 911<br>33.87 |
| 2 | 0<br>0.00<br>0.00<br>0.00 | 1779<br>66.13<br>100.00<br>91.23 | 1779<br>66.13 |
| Total | 740<br>27.51 | 1950<br>72.49 | 2690<br>100.00 |

Frequency Missing = 1

| Frequency<br>Percent<br>Row Pct<br>Col Pct |
|---|

Table of CAD by History_of_MI

| CAD | History_of_MI(History of MI) 1 | 2 | Total |
|---|---|---|---|
| 1 | 403<br>14.98<br>44.24<br>100.00 | 508<br>18.88<br>55.76<br>22.21 | 911<br>33.87 |
| 2 | 0<br>0.00<br>0.00<br>0.00 | 1779<br>66.13<br>100.00<br>77.79 | 1779<br>66.13 |
| Total | 403<br>14.98 | 2287<br>85.02 | 2690<br>100.00 |

Frequency Missing = 1

| Frequency<br>Percent<br>Row Pct<br>Col Pct |
|---|

Table of CAD by History_of_angina

| CAD | History_of_angina(History of angina) 1 | 2 | Total |
|---|---|---|---|
| 1 | 740<br>27.51<br>81.23<br>100.00 | 171<br>6.36<br>18.77<br>8.77 | 911<br>33.87 |
| 2 | 0<br>0.00<br>0.00<br>0.00 | 1779<br>66.13<br>100.00<br>91.23 | 1779<br>66.13 |
| Total | 740<br>27.51 | 1950<br>72.49 | 2690<br>100.00 |

Frequency Missing = 1

## The FREQ Procedure

| Frequency<br>Percent<br>Row Pct<br>Col Pct |
|---|

| Table of CAD by History_of_CABG | | | |
|---|---|---|---|
| | History_of_CABG(History of CABG) | | |
| CAD | 1 | 2 | Total |
| 1 | 71<br>2.64<br>7.79<br>100.00 | 840<br>31.23<br>92.21<br>32.07 | 911<br>33.87 |
| 2 | 0<br>0.00<br>0.00<br>0.00 | 1779<br>66.13<br>100.00<br>67.93 | 1779<br>66.13 |
| Total | 71<br>2.64 | 2619<br>97.36 | 2690<br>100.00 |
| Frequency Missing = 1 | | | |

## The LOGISTIC Procedure

| Model Information | | |
|---|---|---|
| **Data Set** | WAEL.STARS4 | |
| **Response Variable** | Atrial_Fibrillation_Flutter | Atrial Fibrillation/Flutter |
| **Number of Response Levels** | 2 | |
| **Model** | binary logit | |
| **Optimization Technique** | Fisher's scoring | |

| | |
|---|---|
| **Number of Observations Read** | 2691 |
| **Number of Observations Used** | 2690 |

| Response Profile | | |
|---|---|---|
| **Ordered Value** | **Atrial_Fibrillation_Flutter** | **Total Frequency** |
| **1** | 1 | 103 |
| **2** | 2 | 2587 |

**Probability modeled is Atrial_Fibrillation_Flutter='1'.**

**Note:** 1 observation was deleted due to missing values for the response or explanatory variables.

| Class Level Information | | |
|---|---|---|
| **Class** | **Value** | **Design Variables** |
| **Gender** | **1** | 0 |
| | **2** | 1 |

| Model Convergence Status |
|---|
| Convergence criterion (GCONV=1E-8) satisfied. |

| Model Fit Statistics | | |
|---|---|---|
| **Criterion** | **Intercept Only** | **Intercept and Covariates** |
| **AIC** | 876.094 | 871.247 |
| **SC** | 881.991 | 883.042 |
| **-2 Log L** | 874.094 | 867.247 |

| Testing Global Null Hypothesis: BETA=0 | | | |
|---|---|---|---|
| **Test** | **Chi-Square** | **DF** | **Pr > ChiSq** |
| **Likelihood Ratio** | 6.8463 | 1 | 0.0089 |
| **Score** | 7.6995 | 1 | 0.0055 |
| **Wald** | 7.4826 | 1 | 0.0062 |

## The LOGISTIC Procedure

| Type 3 Analysis of Effects | | | |
|---|---|---|---|
| Effect | DF | Wald Chi-Square | Pr > ChiSq |
| Gender | 1 | 7.4826 | 0.0062 |

| Analysis of Maximum Likelihood Estimates | | | | | | |
|---|---|---|---|---|---|---|
| Parameter | | DF | Estimate | Standard Error | Wald Chi-Square | Pr > ChiSq |
| Intercept | | 1 | -3.3622 | 0.1182 | 808.4810 | <.0001 |
| Gender | 2 | 1 | 0.6158 | 0.2251 | 7.4826 | 0.0062 |

| Odds Ratio Estimates | | | |
|---|---|---|---|
| Effect | Point Estimate | 95% Wald Confidence Limits | |
| Gender 2 vs 1 | 1.851 | 1.191 | 2.878 |

| Association of Predicted Probabilities and Observed Responses | | | |
|---|---|---|---|
| Percent Concordant | 23.2 | Somers' D | 0.107 |
| Percent Discordant | 12.6 | Gamma | 0.299 |
| Percent Tied | 64.2 | Tau-a | 0.008 |
| Pairs | 266461 | c | 0.553 |

## The LOGISTIC Procedure

| Model Information | | |
|---|---|---|
| **Data Set** | WAEL.STARS4 | |
| **Response Variable** | Atrial_Fibrillation_Flutter | Atrial Fibrillation/Flutter |
| **Number of Response Levels** | 2 | |
| **Model** | binary logit | |
| **Optimization Technique** | Fisher's scoring | |

| | |
|---|---|
| **Number of Observations Read** | 2691 |
| **Number of Observations Used** | 2385 |

| Response Profile | | |
|---|---|---|
| **Ordered Value** | **Atrial_Fibrillation_Flutter** | **Total Frequency** |
| **1** | 1 | 101 |
| **2** | 2 | 2284 |

**Probability modeled is Atrial_Fibrillation_Flutter='1'.**

**Note:** 306 observations were deleted due to missing values for the response or explanatory variables.

| Class Level Information | | | | |
|---|---|---|---|---|
| **Class** | **Value** | **Design Variables** | | |
| **Gender** | **1** | 0 | | |
| | **2** | 1 | | |
| **Nationality** | **1** | 1 | | |
| | **2** | 0 | | |
| **CAD** | **1** | 1 | | |
| | **2** | 0 | | |
| **History_of_heart_failure** | **1** | 0 | | |
| | **2** | 1 | | |
| **History_of_stroke** | **1** | 0 | | |
| | **2** | 1 | | |
| **History_of_chronic_renal_failure** | **1** | 0 | | |
| | **2** | 1 | | |
| **DM** | **1** | 0 | | |
| | **2** | 1 | | |
| **HTN** | **1** | 0 | | |
| | **2** | 1 | | |
| **CHF_Killip_Class** | **1** | 0 | 0 | 0 |

## The LOGISTIC Procedure

| Class Level Information | | | | |
|---|---|---|---|---|
| Class | Value | Design Variables | | |
| | 2 | 1 | 0 | 0 |
| | 3 | 0 | 1 | 0 |
| | 4 | 0 | 0 | 1 |
| Echo_Options | 1 | 0 | 0 | 0 |
| | 2 | 1 | 0 | 0 |
| | 3 | 0 | 1 | 0 |
| | 4 | 0 | 0 | 1 |

| Model Convergence Status |
|---|
| Convergence criterion (GCONV=1E-8) satisfied. |

| Model Fit Statistics | | |
|---|---|---|
| Criterion | Intercept Only | Intercept and Covariates |
| AIC | 838.352 | 762.487 |
| SC | 844.129 | 860.695 |
| -2 Log L | 836.352 | 728.487 |

| Testing Global Null Hypothesis: BETA=0 | | | |
|---|---|---|---|
| Test | Chi-Square | DF | Pr > ChiSq |
| Likelihood Ratio | 107.8650 | 16 | <.0001 |
| Score | 147.0052 | 16 | <.0001 |
| Wald | 109.7485 | 16 | <.0001 |

| Type 3 Analysis of Effects | | | |
|---|---|---|---|
| Effect | DF | Wald Chi-Square | Pr > ChiSq |
| Gender | 1 | 0.1043 | 0.7468 |
| Nationality | 1 | 4.8023 | 0.0284 |
| History_of_heart_fai | 1 | 1.6825 | 0.1946 |
| History_of_stroke | 1 | 2.9350 | 0.0867 |
| History_of_chronic_r | 1 | 0.3509 | 0.5536 |
| DM | 1 | 0.0223 | 0.8812 |
| HTN | 1 | 0.0061 | 0.9376 |
| CHF_Killip_Class | 3 | 37.4121 | <.0001 |

## The LOGISTIC Procedure

| Type 3 Analysis of Effects | | | |
|---|---|---|---|
| Effect | DF | Wald Chi-Square | Pr > ChiSq |
| Age | 1 | 11.6532 | 0.0006 |
| BMI | 1 | 3.4342 | 0.0639 |
| CAD | 1 | 0.2445 | 0.6210 |
| Echo_Options | 3 | 3.8198 | 0.2816 |

| Analysis of Maximum Likelihood Estimates | | | | | | |
|---|---|---|---|---|---|---|
| Parameter | | DF | Estimate | Standard Error | Wald Chi-Square | Pr > ChiSq |
| Intercept | | 1 | -6.2669 | 1.0505 | 35.5890 | <.0001 |
| Gender | 2 | 1 | 0.0806 | 0.2495 | 0.1043 | 0.7468 |
| Nationality | 1 | 1 | 0.6804 | 0.3105 | 4.8023 | 0.0284 |
| History_of_heart_fai | 2 | 1 | -0.4484 | 0.3457 | 1.6825 | 0.1946 |
| History_of_stroke | 2 | 1 | -0.6013 | 0.3510 | 2.9350 | 0.0867 |
| History_of_chronic_r | 2 | 1 | 0.1926 | 0.3252 | 0.3509 | 0.5536 |
| DM | 2 | 1 | -0.0377 | 0.2524 | 0.0223 | 0.8812 |
| HTN | 2 | 1 | -0.0209 | 0.2669 | 0.0061 | 0.9376 |
| CHF_Killip_Class | 2 | 1 | 1.6842 | 0.2885 | 34.0717 | <.0001 |
| CHF_Killip_Class | 3 | 1 | 0.9501 | 0.4196 | 5.1259 | 0.0236 |
| CHF_Killip_Class | 4 | 1 | 1.7062 | 0.5513 | 9.5771 | 0.0020 |
| Age | | 1 | 0.0314 | 0.00919 | 11.6532 | 0.0006 |
| BMI | | 1 | 0.0373 | 0.0201 | 3.4342 | 0.0639 |
| CAD | 1 | 1 | 0.1170 | 0.2366 | 0.2445 | 0.6210 |
| Echo_Options | 2 | 1 | -0.1125 | 0.2847 | 0.1561 | 0.6928 |
| Echo_Options | 3 | 1 | -0.1656 | 0.3206 | 0.2666 | 0.6056 |
| Echo_Options | 4 | 1 | 0.4526 | 0.3552 | 1.6239 | 0.2025 |

| Odds Ratio Estimates | | | |
|---|---|---|---|
| Effect | Point Estimate | 95% Wald Confidence Limits | |
| Gender 2 vs 1 | 1.084 | 0.665 | 1.768 |
| Nationality 1 vs 2 | 1.975 | 1.075 | 3.629 |
| History_of_heart_fai 2 vs 1 | 0.639 | 0.324 | 1.258 |
| History_of_stroke 2 vs 1 | 0.548 | 0.275 | 1.090 |
| History_of_chronic_r 2 vs 1 | 1.212 | 0.641 | 2.293 |
| DM 2 vs 1 | 0.963 | 0.587 | 1.579 |
| HTN 2 vs 1 | 0.979 | 0.580 | 1.652 |

## The LOGISTIC Procedure

| Odds Ratio Estimates | | | |
|---|---|---|---|
| Effect | Point Estimate | 95% Wald Confidence Limits | |
| CHF_Killip_Class 2 vs 1 | 5.388 | 3.061 | 9.484 |
| CHF_Killip_Class 3 vs 1 | 2.586 | 1.136 | 5.886 |
| CHF_Killip_Class 4 vs 1 | 5.508 | 1.869 | 16.229 |
| Age | 1.032 | 1.013 | 1.051 |
| BMI | 1.038 | 0.998 | 1.080 |
| CAD 1 vs 2 | 1.124 | 0.707 | 1.787 |
| Echo_Options 2 vs 1 | 0.894 | 0.511 | 1.561 |
| Echo_Options 3 vs 1 | 0.847 | 0.452 | 1.589 |
| Echo_Options 4 vs 1 | 1.572 | 0.784 | 3.154 |

| Association of Predicted Probabilities and Observed Responses | | | |
|---|---|---|---|
| Percent Concordant | 78.6 | Somers' D | 0.572 |
| Percent Discordant | 21.4 | Gamma | 0.572 |
| Percent Tied | 0.0 | Tau-a | 0.046 |
| Pairs | 230684 | c | 0.786 |

## The FREQ Procedure

| Frequency<br>Percent<br>Row Pct<br>Col Pct |
| --- |

| Table of Recurrent_ischemia by Gender | | | |
| --- | --- | --- | --- |
| Recurrent_ischemia(Recurrent ischemia) | Gender(Gender) | | |
| | 1 | 2 | Total |
| 1 | 276<br>10.26<br>76.03<br>12.49 | 87<br>3.23<br>23.97<br>18.09 | 363<br>13.49 |
| 2 | 1933<br>71.86<br>83.07<br>87.51 | 394<br>14.65<br>16.93<br>81.91 | 2327<br>86.51 |
| Total | 2209<br>82.12 | 481<br>17.88 | 2690<br>100.00 |
| Frequency Missing = 1 | | | |

## Statistics for Table of Recurrent_ischemia by Gender

| Statistic | DF | Value | Prob |
| --- | --- | --- | --- |
| Chi-Square | 1 | 10.5846 | 0.0011 |
| Likelihood Ratio Chi-Square | 1 | 9.9149 | 0.0016 |
| Continuity Adj. Chi-Square | 1 | 10.1109 | 0.0015 |
| Mantel-Haenszel Chi-Square | 1 | 10.5807 | 0.0011 |
| Phi Coefficient | | -0.0627 | |
| Contingency Coefficient | | 0.0626 | |
| Cramer's V | | -0.0627 | |

| Fisher's Exact Test | |
| --- | --- |
| Cell (1,1) Frequency (F) | 276 |
| Left-sided Pr <= F | 0.0010 |
| Right-sided Pr >= F | 0.9994 |
| | |
| Table Probability (P) | 0.0004 |
| Two-sided Pr <= P | 0.0015 |

**Sample Size = 2690**
**Frequency Missing = 1**

## The FREQ Procedure

| Frequency<br>Percent<br>Row Pct<br>Col Pct |
|---|

| Table of Recurrent_ischemia by Nationality | | | |
|---|---|---|---|
| Recurrent_ischemia(Recurrent ischemia) | Nationality(Nationality) | | |
| | 1 | 2 | Total |
| 1 | 240<br>8.92<br>66.12<br>12.89 | 123<br>4.57<br>33.88<br>14.86 | 363<br>13.49 |
| 2 | 1622<br>60.30<br>69.70<br>87.11 | 705<br>26.21<br>30.30<br>85.14 | 2327<br>86.51 |
| Total | 1862<br>69.22 | 828<br>30.78 | 2690<br>100.00 |
| Frequency Missing = 1 | | | |

## Statistics for Table of Recurrent_ischemia by Nationality

| Statistic | DF | Value | Prob |
|---|---|---|---|
| Chi-Square | 1 | 1.8971 | 0.1684 |
| Likelihood Ratio Chi-Square | 1 | 1.8692 | 0.1716 |
| Continuity Adj. Chi-Square | 1 | 1.7325 | 0.1881 |
| Mantel-Haenszel Chi-Square | 1 | 1.8964 | 0.1685 |
| Phi Coefficient | | -0.0266 | |
| Contingency Coefficient | | 0.0265 | |
| Cramer's V | | -0.0266 | |

| Fisher's Exact Test | |
|---|---|
| Cell (1,1) Frequency (F) | 240 |
| Left-sided Pr <= F | 0.0947 |
| Right-sided Pr >= F | 0.9240 |
| | |
| Table Probability (P) | 0.0187 |
| Two-sided Pr <= P | 0.1787 |

**Sample Size = 2690**
**Frequency Missing = 1**

## The FREQ Procedure

| Frequency<br>Percent<br>Row Pct<br>Col Pct |
|---|

| Table of Recurrent_ischemia by Ethnicity | | | | |
|---|---|---|---|---|
| Recurrent_ischemia(Recurrent ischemia) | Ethnicity(Ethnicity) | | | |
| | 1 | 2 | 3 | Total |
| 1 | 281<br>10.45<br>77.41<br>13.39 | 71<br>2.64<br>19.56<br>14.06 | 11<br>0.41<br>3.03<br>12.79 | 363<br>13.49 |
| 2 | 1818<br>67.58<br>78.13<br>86.61 | 434<br>16.13<br>18.65<br>85.94 | 75<br>2.79<br>3.22<br>87.21 | 2327<br>86.51 |
| Total | 2099<br>78.03 | 505<br>18.77 | 86<br>3.20 | 2690<br>100.00 |
| Frequency Missing = 1 | | | | |

## Statistics for Table of Recurrent_ischemia by Ethnicity

| Statistic | DF | Value | Prob |
|---|---|---|---|
| Chi-Square | 2 | 0.1952 | 0.9070 |
| Likelihood Ratio Chi-Square | 2 | 0.1942 | 0.9075 |
| Mantel-Haenszel Chi-Square | 1 | 0.0341 | 0.8536 |
| Phi Coefficient | | 0.0085 | |
| Contingency Coefficient | | 0.0085 | |
| Cramer's V | | 0.0085 | |

**Sample Size = 2690**
**Frequency Missing = 1**

| Frequency<br>Percent<br>Row Pct<br>Col Pct |
|---|

| Table of Recurrent_ischemia by Type_of_STEMI | | | | |
|---|---|---|---|---|
| Recurrent_ischemia(Recurrent ischemia) | Type_of_STEMI(Type of STEMI) | | | |
| | 1 | 2 | 3 | Total |
| 1 | 71<br>5.41<br>52.21<br>10.29 | 54<br>4.11<br>39.71<br>9.91 | 11<br>0.84<br>8.09<br>14.10 | 136<br>10.36 |
| 2 | 619<br>47.14<br>52.59<br>89.71 | 491<br>37.40<br>41.72<br>90.09 | 67<br>5.10<br>5.69<br>85.90 | 1177<br>89.64 |
| Total | 690<br>52.55 | 545<br>41.51 | 78<br>5.94 | 1313<br>100.00 |
| Frequency Missing = 1378 | | | | |

## The FREQ Procedure

### Statistics for Table of Recurrent_ischemia by Type_of_STEMI

| Statistic | DF | Value | Prob |
|---|---|---|---|
| Chi-Square | 2 | 1.3001 | 0.5220 |
| Likelihood Ratio Chi-Square | 2 | 1.1951 | 0.5502 |
| Mantel-Haenszel Chi-Square | 1 | 0.2563 | 0.6127 |
| Phi Coefficient | | 0.0315 | |
| Contingency Coefficient | | 0.0315 | |
| Cramer's V | | 0.0315 | |

**Sample Size = 1313**
**Frequency Missing = 1378**

**WARNING: 51% of the data are missing.**

| Frequency<br>Percent<br>Row Pct<br>Col Pct |
|---|

Table of Recurrent_ischemia by History_of_angina

| Recurrent_ischemia(Recurrent ischemia) | History_of_angina(History of angina) | | |
|---|---|---|---|
| | 1 | 2 | Total |
| 1 | 175<br>6.51<br>48.21<br>23.65 | 188<br>6.99<br>51.79<br>9.64 | 363<br>13.49 |
| 2 | 565<br>21.00<br>24.28<br>76.35 | 1762<br>65.50<br>75.72<br>90.36 | 2327<br>86.51 |
| Total | 740<br>27.51 | 1950<br>72.49 | 2690<br>100.00 |

Frequency Missing = 1

## The FREQ Procedure

### Statistics for Table of Recurrent_ischemia by History_of_angina

| Statistic | DF | Value | Prob |
|---|---|---|---|
| Chi-Square | 1 | 90.1664 | <.0001 |
| Likelihood Ratio Chi-Square | 1 | 82.4164 | <.0001 |
| Continuity Adj. Chi-Square | 1 | 88.9704 | <.0001 |
| Mantel-Haenszel Chi-Square | 1 | 90.1328 | <.0001 |
| Phi Coefficient | | 0.1831 | |
| Contingency Coefficient | | 0.1801 | |
| Cramer's V | | 0.1831 | |

| Fisher's Exact Test | |
|---|---|
| Cell (1,1) Frequency (F) | 175 |
| Left-sided Pr <= F | 1.0000 |
| Right-sided Pr >= F | <.0001 |
| | |
| Table Probability (P) | <.0001 |
| Two-sided Pr <= P | <.0001 |

**Sample Size = 2690**
**Frequency Missing = 1**

| Frequency<br>Percent<br>Row Pct<br>Col Pct |
|---|

| Table of Recurrent_ischemia by History_of_MI | | | |
|---|---|---|---|
| Recurrent_ischemia(Recurrent ischemia) | History_of_MI(History of MI) | | |
| | 1 | 2 | Total |
| 1 | 110<br>4.09<br>30.30<br>27.30 | 253<br>9.41<br>69.70<br>11.06 | 363<br>13.49 |
| 2 | 293<br>10.89<br>12.59<br>72.70 | 2034<br>75.61<br>87.41<br>88.94 | 2327<br>86.51 |
| Total | 403<br>14.98 | 2287<br>85.02 | 2690<br>100.00 |
| Frequency Missing = 1 | | | |

## The FREQ Procedure

### Statistics for Table of Recurrent_ischemia by History_of_MI

| Statistic | DF | Value | Prob |
|---|---|---|---|
| Chi-Square | 1 | 77.3402 | <.0001 |
| Likelihood Ratio Chi-Square | 1 | 65.3631 | <.0001 |
| Continuity Adj. Chi-Square | 1 | 75.9558 | <.0001 |
| Mantel-Haenszel Chi-Square | 1 | 77.3114 | <.0001 |
| Phi Coefficient | | 0.1696 | |
| Contingency Coefficient | | 0.1672 | |
| Cramer's V | | 0.1696 | |

| Fisher's Exact Test | |
|---|---|
| Cell (1,1) Frequency (F) | 110 |
| Left-sided Pr <= F | 1.0000 |
| Right-sided Pr >= F | <.0001 |
| | |
| Table Probability (P) | <.0001 |
| Two-sided Pr <= P | <.0001 |

**Sample Size = 2690**
**Frequency Missing = 1**

| Frequency<br>Percent<br>Row Pct<br>Col Pct |
|---|

| Table of Recurrent_ischemia by History_of_PCI | | | |
|---|---|---|---|
| Recurrent_ischemia(Recurrent ischemia) | History_of_PCI(History of PCI) | | |
| | 1 | 2 | Total |
| 1 | 93<br>3.46<br>25.62<br>22.20 | 270<br>10.04<br>74.38<br>11.89 | 363<br>13.49 |
| 2 | 326<br>12.12<br>14.01<br>77.80 | 2001<br>74.39<br>85.99<br>88.11 | 2327<br>86.51 |
| Total | 419<br>15.58 | 2271<br>84.42 | 2690<br>100.00 |
| Frequency Missing = 1 | | | |

## The FREQ Procedure

### Statistics for Table of Recurrent_ischemia by History_of_PCI

| Statistic | DF | Value | Prob |
|---|---|---|---|
| Chi-Square | 1 | 32.1897 | <.0001 |
| Likelihood Ratio Chi-Square | 1 | 28.6306 | <.0001 |
| Continuity Adj. Chi-Square | 1 | 31.3128 | <.0001 |
| Mantel-Haenszel Chi-Square | 1 | 32.1777 | <.0001 |
| Phi Coefficient | | 0.1094 | |
| Contingency Coefficient | | 0.1087 | |
| Cramer's V | | 0.1094 | |

| Fisher's Exact Test | |
|---|---|
| Cell (1,1) Frequency (F) | 93 |
| Left-sided Pr <= F | 1.0000 |
| Right-sided Pr >= F | <.0001 |
| | |
| Table Probability (P) | <.0001 |
| Two-sided Pr <= P | <.0001 |

**Sample Size = 2690**
**Frequency Missing = 1**

| Frequency<br>Percent<br>Row Pct<br>Col Pct |
|---|

| Table of Recurrent_ischemia by History_of_CABG | | | |
|---|---|---|---|
| Recurrent_ischemia(Recurrent ischemia) | History_of_CABG(History of CABG) | | |
| | 1 | 2 | Total |
| 1 | 23<br>0.86<br>6.34<br>32.39 | 340<br>12.64<br>93.66<br>12.98 | 363<br>13.49 |
| 2 | 48<br>1.78<br>2.06<br>67.61 | 2279<br>84.72<br>97.94<br>87.02 | 2327<br>86.51 |
| Total | 71<br>2.64 | 2619<br>97.36 | 2690<br>100.00 |
| Frequency Missing = 1 | | | |

## The FREQ Procedure

### Statistics for Table of Recurrent_ischemia by History_of_CABG

| Statistic | DF | Value | Prob |
|---|---|---|---|
| Chi-Square | 1 | 22.3150 | <.0001 |
| Likelihood Ratio Chi-Square | 1 | 17.2123 | <.0001 |
| Continuity Adj. Chi-Square | 1 | 20.6831 | <.0001 |
| Mantel-Haenszel Chi-Square | 1 | 22.3067 | <.0001 |
| Phi Coefficient | | 0.0911 | |
| Contingency Coefficient | | 0.0907 | |
| Cramer's V | | 0.0911 | |

| Fisher's Exact Test | |
|---|---|
| Cell (1,1) Frequency (F) | 23 |
| Left-sided Pr <= F | 1.0000 |
| Right-sided Pr >= F | <.0001 |
| | |
| Table Probability (P) | <.0001 |
| Two-sided Pr <= P | <.0001 |

### Sample Size = 2690
### Frequency Missing = 1

| Frequency<br>Percent<br>Row Pct<br>Col Pct |
|---|

| Table of Recurrent_ischemia by History_of_heart_failure | | | |
|---|---|---|---|
| Recurrent_ischemia(Recurrent ischemia) | History_of_heart_failure(History of heart failure) | | |
| | 1 | 2 | Total |
| 1 | 56<br>2.08<br>15.43<br>34.36 | 307<br>11.41<br>84.57<br>12.15 | 363<br>13.49 |
| 2 | 107<br>3.98<br>4.60<br>65.64 | 2220<br>82.53<br>95.40<br>87.85 | 2327<br>86.51 |
| Total | 163<br>6.06 | 2527<br>93.94 | 2690<br>100.00 |
| Frequency Missing = 1 | | | |

## The FREQ Procedure

### Statistics for Table of Recurrent_ischemia by History_of_heart_failure

| Statistic | DF | Value | Prob |
|---|---|---|---|
| Chi-Square | 1 | 64.6879 | <.0001 |
| Likelihood Ratio Chi-Square | 1 | 49.6437 | <.0001 |
| Continuity Adj. Chi-Square | 1 | 62.7996 | <.0001 |
| Mantel-Haenszel Chi-Square | 1 | 64.6639 | <.0001 |
| Phi Coefficient | | 0.1551 | |
| Contingency Coefficient | | 0.1532 | |
| Cramer's V | | 0.1551 | |

| Fisher's Exact Test | |
|---|---|
| Cell (1,1) Frequency (F) | 56 |
| Left-sided Pr <= F | 1.0000 |
| Right-sided Pr >= F | <.0001 |
| | |
| Table Probability (P) | <.0001 |
| Two-sided Pr <= P | <.0001 |

**Sample Size = 2690**
**Frequency Missing = 1**

| Frequency<br>Percent<br>Row Pct<br>Col Pct |
|---|

| Table of Recurrent_ischemia by History_of_stroke | | | |
|---|---|---|---|
| | History_of_stroke(History of stroke) | | |
| Recurrent_ischemia(Recurrent ischemia) | 1 | 2 | Total |
| 1 | 23<br>0.86<br>6.34<br>18.70 | 340<br>12.64<br>93.66<br>13.25 | 363<br>13.49 |
| 2 | 100<br>3.72<br>4.30<br>81.30 | 2227<br>82.79<br>95.70<br>86.75 | 2327<br>86.51 |
| Total | 123<br>4.57 | 2567<br>95.43 | 2690<br>100.00 |
| Frequency Missing = 1 | | | |

## The FREQ Procedure

### Statistics for Table of Recurrent_ischemia by History_of_stroke

| Statistic | DF | Value | Prob |
|---|---|---|---|
| Chi-Square | 1 | 2.9911 | 0.0837 |
| Likelihood Ratio Chi-Square | 1 | 2.7323 | 0.0983 |
| Continuity Adj. Chi-Square | 1 | 2.5421 | 0.1108 |
| Mantel-Haenszel Chi-Square | 1 | 2.9900 | 0.0838 |
| Phi Coefficient | | 0.0333 | |
| Contingency Coefficient | | 0.0333 | |
| Cramer's V | | 0.0333 | |

| Fisher's Exact Test | |
|---|---|
| Cell (1,1) Frequency (F) | 23 |
| Left-sided Pr <= F | 0.9644 |
| Right-sided Pr >= F | 0.0598 |
| | |
| Table Probability (P) | 0.0242 |
| Two-sided Pr <= P | 0.1033 |

**Sample Size = 2690**
**Frequency Missing = 1**

Frequency
Percent
Row Pct
Col Pct

| Table of Recurrent_ischemia by History_of_chronic_renal_failure | | | |
|---|---|---|---|
| Recurrent_ischemia(Recurrent ischemia) | History_of_chronic_renal_failure(History of chronic renal failure) | | |
| | 1 | 2 | Total |
| 1 | 50<br>1.86<br>13.77<br>25.00 | 313<br>11.64<br>86.23<br>12.57 | 363<br>13.49 |
| 2 | 150<br>5.58<br>6.45<br>75.00 | 2177<br>80.93<br>93.55<br>87.43 | 2327<br>86.51 |
| Total | 200<br>7.43 | 2490<br>92.57 | 2690<br>100.00 |
| Frequency Missing = 1 | | | |

## The FREQ Procedure

### Statistics for Table of Recurrent_ischemia by History_of_chronic_renal_failure

| Statistic | DF | Value | Prob |
|---|---|---|---|
| Chi-Square | 1 | 24.5020 | <.0001 |
| Likelihood Ratio Chi-Square | 1 | 20.7018 | <.0001 |
| Continuity Adj. Chi-Square | 1 | 23.4488 | <.0001 |
| Mantel-Haenszel Chi-Square | 1 | 24.4929 | <.0001 |
| Phi Coefficient | | 0.0954 | |
| Contingency Coefficient | | 0.0950 | |
| Cramer's V | | 0.0954 | |

| Fisher's Exact Test | |
|---|---|
| Cell (1,1) Frequency (F) | 50 |
| Left-sided Pr <= F | 1.0000 |
| Right-sided Pr >= F | <.0001 |
| | |
| Table Probability (P) | <.0001 |
| Two-sided Pr <= P | <.0001 |

**Sample Size = 2690**
**Frequency Missing = 1**

Frequency
Percent
Row Pct
Col Pct

| Table of Recurrent_ischemia by DM | | | |
|---|---|---|---|
| | DM(DM) | | |
| Recurrent_ischemia(Recurrent ischemia) | 1 | 2 | Total |
| 1 | 245<br>9.11<br>67.49<br>15.72 | 118<br>4.39<br>32.51<br>10.43 | 363<br>13.49 |
| 2 | 1314<br>48.85<br>56.47<br>84.28 | 1013<br>37.66<br>43.53<br>89.57 | 2327<br>86.51 |
| Total | 1559<br>57.96 | 1131<br>42.04 | 2690<br>100.00 |
| Frequency Missing = 1 | | | |

## The FREQ Procedure

### Statistics for Table of Recurrent_ischemia by DM

| Statistic | DF | Value | Prob |
|---|---|---|---|
| Chi-Square | 1 | 15.6656 | <.0001 |
| Likelihood Ratio Chi-Square | 1 | 16.0421 | <.0001 |
| Continuity Adj. Chi-Square | 1 | 15.2164 | <.0001 |
| Mantel-Haenszel Chi-Square | 1 | 15.6598 | <.0001 |
| Phi Coefficient | | 0.0763 | |
| Contingency Coefficient | | 0.0761 | |
| Cramer's V | | 0.0763 | |

| Fisher's Exact Test | |
|---|---|
| Cell (1,1) Frequency (F) | 245 |
| Left-sided Pr <= F | 1.0000 |
| Right-sided Pr >= F | <.0001 |
| | |
| Table Probability (P) | <.0001 |
| Two-sided Pr <= P | <.0001 |

**Sample Size = 2690**
**Frequency Missing = 1**

| Frequency<br>Percent<br>Row Pct<br>Col Pct |
|---|

| Table of Recurrent_ischemia by HTN | | | |
|---|---|---|---|
| Recurrent_ischemia(Recurrent ischemia) | HTN(HTN) | | |
| | 1 | 2 | Total |
| 1 | 248<br>9.22<br>68.32<br>15.76 | 115<br>4.28<br>31.68<br>10.30 | 363<br>13.49 |
| 2 | 1326<br>49.29<br>56.98<br>84.24 | 1001<br>37.21<br>43.02<br>89.70 | 2327<br>86.51 |
| Total | 1574<br>58.51 | 1116<br>41.49 | 2690<br>100.00 |
| Frequency Missing = 1 | | | |

## The FREQ Procedure

### Statistics for Table of Recurrent_ischemia by HTN

| Statistic | DF | Value | Prob |
|---|---|---|---|
| Chi-Square | 1 | 16.6238 | <.0001 |
| Likelihood Ratio Chi-Square | 1 | 17.0629 | <.0001 |
| Continuity Adj. Chi-Square | 1 | 16.1601 | <.0001 |
| Mantel-Haenszel Chi-Square | 1 | 16.6176 | <.0001 |
| Phi Coefficient | | 0.0786 | |
| Contingency Coefficient | | 0.0784 | |
| Cramer's V | | 0.0786 | |

| Fisher's Exact Test | |
|---|---|
| Cell (1,1) Frequency (F) | 248 |
| Left-sided Pr <= F | 1.0000 |
| Right-sided Pr >= F | <.0001 |
| | |
| Table Probability (P) | <.0001 |
| Two-sided Pr <= P | <.0001 |

**Sample Size = 2690**
**Frequency Missing = 1**

| Frequency<br>Percent<br>Row Pct<br>Col Pct |
|---|

| Table of Recurrent_ischemia by Hypercholestrolemia | | | |
|---|---|---|---|
| Recurrent_ischemia(Recurrent ischemia) | Hypercholestrolemia(Hypercholestrolemia) | | |
| | 1 | 2 | Total |
| 1 | 201<br>7.47<br>55.37<br>19.16 | 162<br>6.02<br>44.63<br>9.87 | 363<br>13.49 |
| 2 | 848<br>31.52<br>36.44<br>80.84 | 1479<br>54.98<br>63.56<br>90.13 | 2327<br>86.51 |
| Total | 1049<br>39.00 | 1641<br>61.00 | 2690<br>100.00 |
| Frequency Missing = 1 | | | |

## The FREQ Procedure

### Statistics for Table of Recurrent_ischemia by Hypercholestrolemia

| Statistic | DF | Value | Prob |
|---|---|---|---|
| Chi-Square | 1 | 47.3019 | <.0001 |
| Likelihood Ratio Chi-Square | 1 | 46.1076 | <.0001 |
| Continuity Adj. Chi-Square | 1 | 46.5095 | <.0001 |
| Mantel-Haenszel Chi-Square | 1 | 47.2843 | <.0001 |
| Phi Coefficient | | 0.1326 | |
| Contingency Coefficient | | 0.1315 | |
| Cramer's V | | 0.1326 | |

| Fisher's Exact Test | |
|---|---|
| Cell (1,1) Frequency (F) | 201 |
| Left-sided Pr <= F | 1.0000 |
| Right-sided Pr >= F | <.0001 |
| | |
| Table Probability (P) | <.0001 |
| Two-sided Pr <= P | <.0001 |

**Sample Size = 2690**
**Frequency Missing = 1**

| Frequency<br>Percent<br>Row Pct<br>Col Pct |
|---|

| Table of Recurrent_ischemia by Current_or_ex_smoking | | | |
|---|---|---|---|
| Recurrent_ischemia(Recurrent ischemia) | Current_or_ex_smoking(Current or ex-smoking) | | |
| | 1 | 2 | Total |
| 1 | 162<br>6.02<br>44.63<br>13.88 | 201<br>7.47<br>55.37<br>13.20 | 363<br>13.49 |
| 2 | 1005<br>37.36<br>43.19<br>86.12 | 1322<br>49.14<br>56.81<br>86.80 | 2327<br>86.51 |
| Total | 1167<br>43.38 | 1523<br>56.62 | 2690<br>100.00 |
| Frequency Missing = 1 | | | |

## The FREQ Procedure

### Statistics for Table of Recurrent_ischemia by Current_or_ex_smoking

| Statistic | DF | Value | Prob |
|---|---|---|---|
| Chi-Square | 1 | 0.2649 | 0.6068 |
| Likelihood Ratio Chi-Square | 1 | 0.2644 | 0.6071 |
| Continuity Adj. Chi-Square | 1 | 0.2095 | 0.6471 |
| Mantel-Haenszel Chi-Square | 1 | 0.2648 | 0.6068 |
| Phi Coefficient | | 0.0099 | |
| Contingency Coefficient | | 0.0099 | |
| Cramer's V | | 0.0099 | |

| Fisher's Exact Test | |
|---|---|
| Cell (1,1) Frequency (F) | 162 |
| Left-sided Pr <= F | 0.7166 |
| Right-sided Pr >= F | 0.3231 |
| | |
| Table Probability (P) | 0.0397 |
| Two-sided Pr <= P | 0.6089 |

**Sample Size = 2690**
**Frequency Missing = 1**

| Frequency<br>Percent<br>Row Pct<br>Col Pct |
|---|

| Table of Recurrent_ischemia by Chief_complaint | | | | | | |
|---|---|---|---|---|---|---|
| Recurrent_ischemia(Recurrent ischemia) | Chief_complaint(Chief complaint) | | | | | |
| | 1 | 2 | 3 | 4 | 5 | Total |
| 1 | 279<br>10.37<br>76.86<br>11.90 | 56<br>2.08<br>15.43<br>31.82 | 24<br>0.89<br>6.61<br>18.90 | 2<br>0.07<br>0.55<br>14.29 | 2<br>0.07<br>0.55<br>6.90 | 363<br>13.49 |
| 2 | 2065<br>76.77<br>88.74<br>88.10 | 120<br>4.46<br>5.16<br>68.18 | 103<br>3.83<br>4.43<br>81.10 | 12<br>0.45<br>0.52<br>85.71 | 27<br>1.00<br>1.16<br>93.10 | 2327<br>86.51 |
| Total | 2344<br>87.14 | 176<br>6.54 | 127<br>4.72 | 14<br>0.52 | 29<br>1.08 | 2690<br>100.00 |
| Frequency Missing = 1 | | | | | | |

## The FREQ Procedure

### Statistics for Table of Recurrent_ischemia by Chief_complaint

| Statistic | DF | Value | Prob |
|---|---|---|---|
| Chi-Square | 4 | 59.9748 | <.0001 |
| Likelihood Ratio Chi-Square | 4 | 48.3785 | <.0001 |
| Mantel-Haenszel Chi-Square | 1 | 11.1691 | 0.0008 |
| Phi Coefficient | | 0.1493 | |
| Contingency Coefficient | | 0.1477 | |
| Cramer's V | | 0.1493 | |

**Sample Size = 2690**
**Frequency Missing = 1**

| Frequency<br>Percent<br>Row Pct<br>Col Pct |
|---|

Table of Recurrent_ischemia by _1st_medical_contact

| Recurrent_ischemia(Recurrent ischemia) | _1st_medical_contact(1st medical contact) | | | | |
|---|---|---|---|---|---|
| | 1 | 2 | 3 | 4 | Total |
| 1 | 39<br>6.23<br>46.43<br>29.32 | 18<br>2.88<br>21.43<br>4.97 | 1<br>0.16<br>1.19<br>100.00 | 26<br>4.15<br>30.95<br>20.00 | 84<br>13.42 |
| 2 | 94<br>15.02<br>17.34<br>70.68 | 344<br>54.95<br>63.47<br>95.03 | 0<br>0.00<br>0.00<br>0.00 | 104<br>16.61<br>19.19<br>80.00 | 542<br>86.58 |
| Total | 133<br>21.25 | 362<br>57.83 | 1<br>0.16 | 130<br>20.77 | 626<br>100.00 |

Frequency Missing = 2065

## The FREQ Procedure

### Statistics for Table of Recurrent_ischemia by _1st_medical_contact

| Statistic | DF | Value | Prob |
|---|---|---|---|
| Chi-Square | 3 | 62.4856 | <.0001 |
| Likelihood Ratio Chi-Square | 3 | 59.4453 | <.0001 |
| Mantel-Haenszel Chi-Square | 1 | 0.1380 | 0.7102 |
| Phi Coefficient | | 0.3159 | |
| Contingency Coefficient | | 0.3013 | |
| Cramer's V | | 0.3159 | |
| WARNING: 25% of the cells have expected counts less than 5. Chi-Square may not be a valid test. | | | |

**Sample Size = 626**
**Frequency Missing = 2065**

**WARNING: 77% of the data are missing.**

| Frequency<br>Percent<br>Row Pct<br>Col Pct |
|---|

| Table of Recurrent_ischemia by Transferred_by_EMS_e_g__Red_Cres | | | |
|---|---|---|---|
| Recurrent_ischemia(Recurrent ischemia) | Transferred_by_EMS_e_g__Red_Cres(Transferred by EMS e.g. Red Crescent or Red Cross?) | | |
| | 1 | 2 | Total |
| 1 | 73<br>12.41<br>65.18<br>31.88 | 39<br>6.63<br>34.82<br>10.86 | 112<br>19.05 |
| 2 | 156<br>26.53<br>32.77<br>68.12 | 320<br>54.42<br>67.23<br>89.14 | 476<br>80.95 |
| Total | 229<br>38.95 | 359<br>61.05 | 588<br>100.00 |
| Frequency Missing = 2103 | | | |

## The FREQ Procedure

### Statistics for Table of Recurrent_ischemia by Transferred_by_EMS_e_g__Red_Cres

| Statistic | DF | Value | Prob |
|---|---|---|---|
| Chi-Square | 1 | 40.0414 | <.0001 |
| Likelihood Ratio Chi-Square | 1 | 39.1846 | <.0001 |
| Continuity Adj. Chi-Square | 1 | 38.6901 | <.0001 |
| Mantel-Haenszel Chi-Square | 1 | 39.9733 | <.0001 |
| Phi Coefficient | | 0.2610 | |
| Contingency Coefficient | | 0.2525 | |
| Cramer's V | | 0.2610 | |

| Fisher's Exact Test | |
|---|---|
| Cell (1,1) Frequency (F) | 73 |
| Left-sided Pr <= F | 1.0000 |
| Right-sided Pr >= F | <.0001 |
| | |
| Table Probability (P) | <.0001 |
| Two-sided Pr <= P | <.0001 |

**Sample Size = 588**
**Frequency Missing = 2103**

**WARNING: 78% of the data are missing.**

| Frequency<br>Percent<br>Row Pct<br>Col Pct |
|---|

| Table of Recurrent_ischemia by Cardiac_arrest | | | |
|---|---|---|---|
| Recurrent_ischemia(Recurrent ischemia) | Cardiac_arrest(Cardiac arrest) | | |
| | 1 | 2 | Total |
| 1 | 17<br>0.63<br>4.68<br>22.97 | 346<br>12.86<br>95.32<br>13.23 | 363<br>13.49 |
| 2 | 57<br>2.12<br>2.45<br>77.03 | 2270<br>84.39<br>97.55<br>86.77 | 2327<br>86.51 |
| Total | 74<br>2.75 | 2616<br>97.25 | 2690<br>100.00 |
| Frequency Missing = 1 | | | |

## The FREQ Procedure

### Statistics for Table of Recurrent_ischemia by Cardiac_arrest

| Statistic | DF | Value | Prob |
|---|---|---|---|
| Chi-Square | 1 | 5.8564 | 0.0155 |
| Likelihood Ratio Chi-Square | 1 | 5.0215 | 0.0250 |
| Continuity Adj. Chi-Square | 1 | 5.0512 | 0.0246 |
| Mantel-Haenszel Chi-Square | 1 | 5.8542 | 0.0155 |
| Phi Coefficient | | 0.0467 | |
| Contingency Coefficient | | 0.0466 | |
| Cramer's V | | 0.0467 | |

| Fisher's Exact Test | |
|---|---|
| Cell (1,1) Frequency (F) | 17 |
| Left-sided Pr <= F | 0.9923 |
| Right-sided Pr >= F | 0.0168 |
| | |
| Table Probability (P) | 0.0091 |
| Two-sided Pr <= P | 0.0233 |

**Sample Size = 2690**
**Frequency Missing = 1**

Frequency
Percent
Row Pct
Col Pct

| Table of Recurrent_ischemia by CHF_Killip_Class | | | | | |
|---|---|---|---|---|---|
| Recurrent_ischemia(Recurrent ischemia) | CHF_Killip_Class(CHF Killip Class) | | | | |
| | 1 | 2 | 3 | 4 | Total |
| 1 | 257<br>9.55<br>70.80<br>10.90 | 67<br>2.49<br>18.46<br>34.36 | 30<br>1.12<br>8.26<br>31.25 | 9<br>0.33<br>2.48<br>21.95 | 363<br>13.49 |
| 2 | 2101<br>78.10<br>90.29<br>89.10 | 128<br>4.76<br>5.50<br>65.64 | 66<br>2.45<br>2.84<br>68.75 | 32<br>1.19<br>1.38<br>78.05 | 2327<br>86.51 |
| Total | 2358<br>87.66 | 195<br>7.25 | 96<br>3.57 | 41<br>1.52 | 2690<br>100.00 |
| Frequency Missing = 1 | | | | | |

## The FREQ Procedure

### Statistics for Table of Recurrent_ischemia by CHF_Killip_Class

| Statistic | DF | Value | Prob |
|---|---|---|---|
| Chi-Square | 3 | 114.7646 | <.0001 |
| Likelihood Ratio Chi-Square | 3 | 91.2353 | <.0001 |
| Mantel-Haenszel Chi-Square | 1 | 72.9814 | <.0001 |
| Phi Coefficient | | 0.2066 | |
| Contingency Coefficient | | 0.2023 | |
| Cramer's V | | 0.2066 | |

**Sample Size = 2690**
**Frequency Missing = 1**

| Frequency<br>Percent<br>Row Pct<br>Col Pct |
|---|

| Table of Recurrent_ischemia by Echo_Options | | | | | |
|---|---|---|---|---|---|
| Recurrent_ischemia(Recurrent ischemia) | Echo_Options(Echo-Options) | | | | |
| | 1 | 2 | 3 | 4 | Total |
| 1 | 106<br>4.44<br>34.19<br>11.19 | 111<br>4.65<br>35.81<br>14.43 | 63<br>2.64<br>20.32<br>13.70 | 30<br>1.26<br>9.68<br>14.35 | 310<br>13.00 |
| 2 | 841<br>35.26<br>40.53<br>88.81 | 658<br>27.59<br>31.71<br>85.57 | 397<br>16.65<br>19.13<br>86.30 | 179<br>7.51<br>8.63<br>85.65 | 2075<br>87.00 |
| Total | 947<br>39.71 | 769<br>32.24 | 460<br>19.29 | 209<br>8.76 | 2385<br>100.00 |
| Frequency Missing = 306 | | | | | |

## The FREQ Procedure

### Statistics for Table of Recurrent_ischemia by Echo_Options

| Statistic | DF | Value | Prob |
|---|---|---|---|
| Chi-Square | 3 | 4.6684 | 0.1978 |
| Likelihood Ratio Chi-Square | 3 | 4.7300 | 0.1927 |
| Mantel-Haenszel Chi-Square | 1 | 2.6599 | 0.1029 |
| Phi Coefficient | | 0.0442 | |
| Contingency Coefficient | | 0.0442 | |
| Cramer's V | | 0.0442 | |

**Sample Size = 2385**
**Frequency Missing = 306**

**WARNING: 11% of the data are missing.**

| Frequency<br>Percent<br>Row Pct<br>Col Pct |
|---|

| Table of Recurrent_ischemia by Elective_coronary_angiogram | | | |
|---|---|---|---|
| Recurrent_ischemia(Recurrent ischemia) | Elective_coronary_angiogram(Elective coronary angiogram) | | |
| | 1 | 2 | Total |
| 1 | 35<br>2.01<br>27.13<br>8.41 | 94<br>5.40<br>72.87<br>7.10 | 129<br>7.41 |
| 2 | 381<br>21.90<br>23.65<br>91.59 | 1230<br>70.69<br>76.35<br>92.90 | 1611<br>92.59 |
| Total | 416<br>23.91 | 1324<br>76.09 | 1740<br>100.00 |
| Frequency Missing = 951 | | | |

## The FREQ Procedure

### Statistics for Table of Recurrent_ischemia by Elective_coronary_angiogram

| Statistic | DF | Value | Prob |
|---|---|---|---|
| Chi-Square | 1 | 0.7959 | 0.3723 |
| Likelihood Ratio Chi-Square | 1 | 0.7750 | 0.3787 |
| Continuity Adj. Chi-Square | 1 | 0.6160 | 0.4325 |
| Mantel-Haenszel Chi-Square | 1 | 0.7955 | 0.3724 |
| Phi Coefficient | | 0.0214 | |
| Contingency Coefficient | | 0.0214 | |
| Cramer's V | | 0.0214 | |

| Fisher's Exact Test | |
|---|---|
| Cell (1,1) Frequency (F) | 35 |
| Left-sided Pr <= F | 0.8413 |
| Right-sided Pr >= F | 0.2145 |
| | |
| Table Probability (P) | 0.0558 |
| Two-sided Pr <= P | 0.3908 |

**Sample Size = 1740**
**Frequency Missing = 951**

**WARNING: 35% of the data are missing.**

Frequency
Percent
Row Pct
Col Pct

| Table of Recurrent_ischemia by Arterial_access | | | | |
|---|---|---|---|---|
| Recurrent_ischemia(Recurrent ischemia) | Arterial_access(Arterial access) | | | |
| | 1 | 2 | 3 | Total |
| 1 | 26<br>2.22<br>32.10<br>9.22 | 55<br>4.70<br>67.90<br>6.21 | 0<br>0.00<br>0.00<br>0.00 | 81<br>6.93 |
| 2 | 256<br>21.90<br>23.53<br>90.78 | 831<br>71.09<br>76.38<br>93.79 | 1<br>0.09<br>0.09<br>100.00 | 1088<br>93.07 |
| Total | 282<br>24.12 | 886<br>75.79 | 1<br>0.09 | 1169<br>100.00 |
| Frequency Missing = 1522 | | | | |

## The FREQ Procedure

### Statistics for Table of Recurrent_ischemia by Arterial_access

| Statistic | DF | Value | Prob |
|---|---|---|---|
| Chi-Square | 2 | 3.0842 | 0.2139 |
| Likelihood Ratio Chi-Square | 2 | 2.9761 | 0.2258 |
| Mantel-Haenszel Chi-Square | 1 | 3.0658 | 0.0800 |
| Phi Coefficient | | 0.0514 | |
| Contingency Coefficient | | 0.0513 | |
| Cramer's V | | 0.0514 | |
| WARNING: 33% of the cells have expected counts less than 5. Chi-Square may not be a valid test. | | | |

**Sample Size = 1169**
**Frequency Missing = 1522**

**WARNING: 57% of the data are missing.**

| Frequency<br>Percent<br>Row Pct<br>Col Pct |
|---|

| Table of Recurrent_ischemia by Arterial_access_1 | | | |
|---|---|---|---|
| Recurrent_ischemia(Recurrent ischemia) | Arterial_access_1(Arterial access_1) | | |
| | 1 | 2 | Total |
| 1 | 3<br>0.72<br>8.57<br>3.09 | 32<br>7.69<br>91.43<br>10.03 | 35<br>8.41 |
| 2 | 94<br>22.60<br>24.67<br>96.91 | 287<br>68.99<br>75.33<br>89.97 | 381<br>91.59 |
| Total | 97<br>23.32 | 319<br>76.68 | 416<br>100.00 |
| Frequency Missing = 2275 | | | |

## The FREQ Procedure

### Statistics for Table of Recurrent_ischemia by Arterial_access_1

| Statistic | DF | Value | Prob |
|---|---|---|---|
| Chi-Square | 1 | 4.6473 | 0.0311 |
| Likelihood Ratio Chi-Square | 1 | 5.6378 | 0.0176 |
| Continuity Adj. Chi-Square | 1 | 3.7905 | 0.0515 |
| Mantel-Haenszel Chi-Square | 1 | 4.6361 | 0.0313 |
| Phi Coefficient | | -0.1057 | |
| Contingency Coefficient | | 0.1051 | |
| Cramer's V | | -0.1057 | |

| Fisher's Exact Test | |
|---|---|
| Cell (1,1) Frequency (F) | 3 |
| Left-sided Pr <= F | 0.0190 |
| Right-sided Pr >= F | 0.9953 |
| | |
| Table Probability (P) | 0.0143 |
| Two-sided Pr <= P | 0.0352 |

**Sample Size = 416**
**Frequency Missing = 2275**

**WARNING: 85% of the data are missing.**

## The TTEST Procedure

### Variable: Age (Age)

| Recurrent_ischemia | Method | N | Mean | Std Dev | Std Err | Minimum | Maximum |
|---|---|---|---|---|---|---|---|
| **1** | | 363 | 58.0000 | 12.0186 | 0.6308 | 23.0000 | 92.0000 |
| **2** | | 2327 | 57.0318 | 12.4918 | 0.2590 | 19.0000 | 103.0 |
| **Diff (1-2)** | **Pooled** | | 0.9682 | 12.4291 | 0.7014 | | |
| **Diff (1-2)** | **Satterthwaite** | | 0.9682 | | 0.6819 | | |

| Recurrent_ischemia | Method | Mean | 95% CL Mean | | Std Dev | 95% CL Std Dev | |
|---|---|---|---|---|---|---|---|
| **1** | | 58.0000 | 56.7595 | 59.2405 | 12.0186 | 11.2033 | 12.9629 |
| **2** | | 57.0318 | 56.5240 | 57.5396 | 12.4918 | 12.1429 | 12.8614 |
| **Diff (1-2)** | **Pooled** | 0.9682 | -0.4071 | 2.3435 | 12.4291 | 12.1056 | 12.7705 |
| **Diff (1-2)** | **Satterthwaite** | 0.9682 | -0.3716 | 2.3080 | | | |

| Method | Variances | DF | t Value | Pr > \|t\| |
|---|---|---|---|---|
| **Pooled** | Equal | 2688 | 1.38 | 0.1676 |
| **Satterthwaite** | Unequal | 492.11 | 1.42 | 0.1563 |

| Equality of Variances | | | | |
|---|---|---|---|---|
| **Method** | **Num DF** | **Den DF** | **F Value** | **Pr > F** |
| **Folded F** | 2326 | 362 | 1.08 | 0.3484 |

## The TTEST Procedure

### Variable: Age (Age)

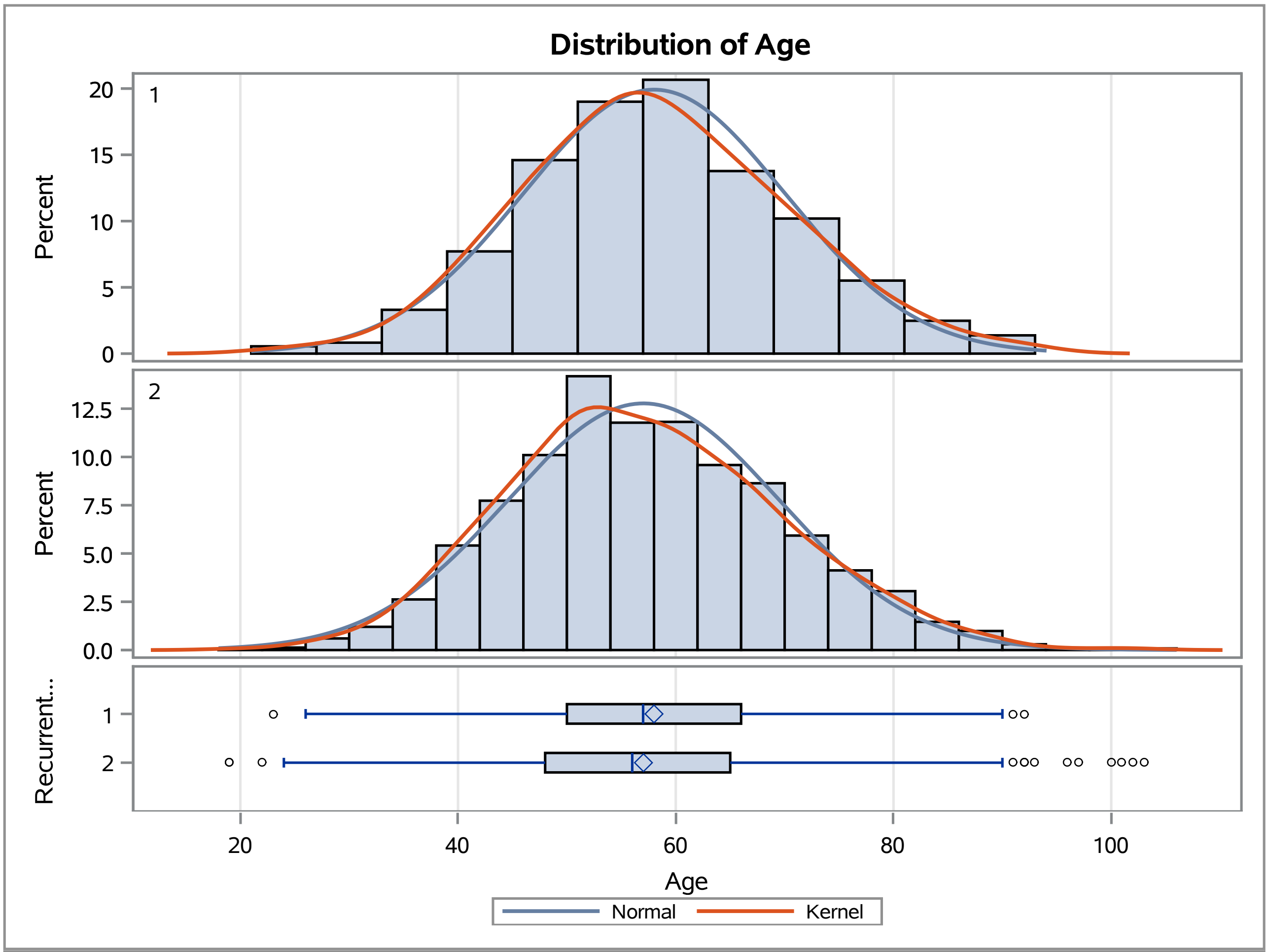


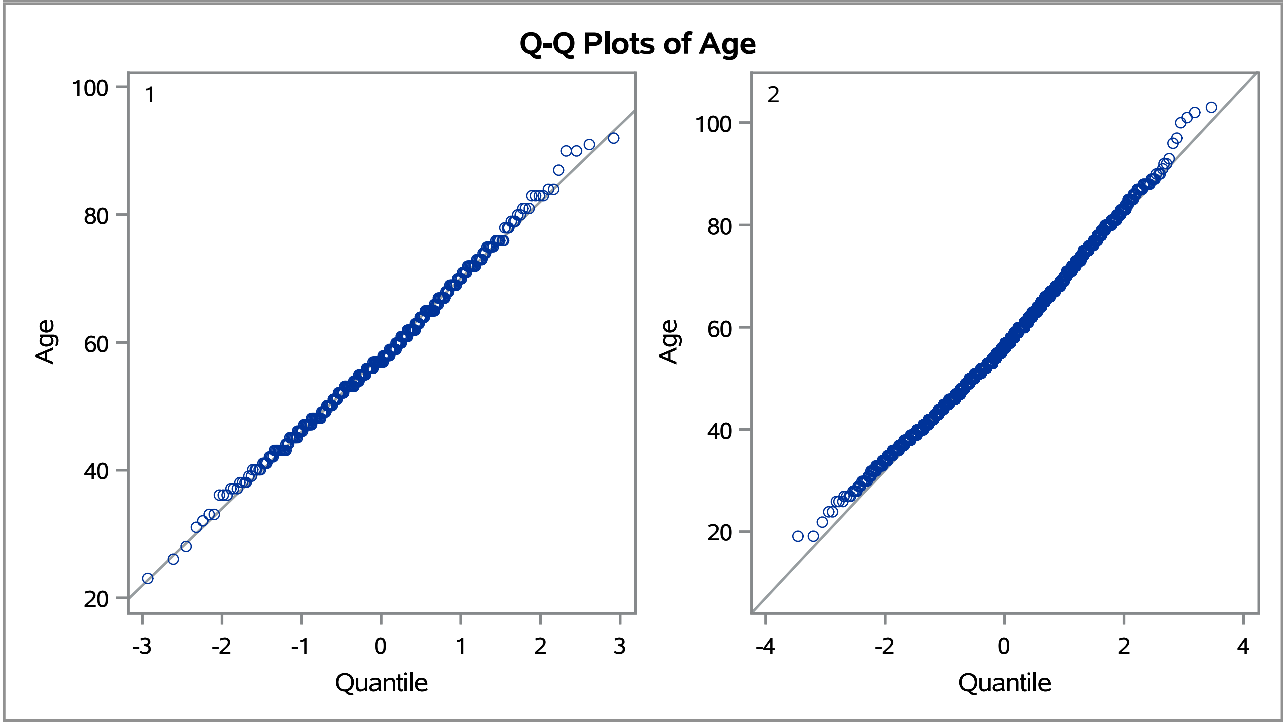

## The TTEST Procedure

### Variable: BMI (BMI)

### Variable: BMI (BMI)

| Recurrent_ischemia | Method | N | Mean | Std Dev | Std Err | Minimum | Maximum |
|---|---|---|---|---|---|---|---|
| 1 | | 363 | 28.8303 | 5.0957 | 0.2675 | 15.1600 | 75.0000 |
| 2 | | 2327 | 28.4853 | 4.7215 | 0.0979 | 17.1000 | 57.1900 |
| Diff (1-2) | Pooled | | 0.3450 | 4.7736 | 0.2694 | | |
| Diff (1-2) | Satterthwaite | | 0.3450 | | 0.2848 | | |

| Recurrent_ischemia | Method | Mean | 95% CL Mean | | Std Dev | 95% CL Std Dev | |
|---|---|---|---|---|---|---|---|
| 1 | | 28.8303 | 28.3043 | 29.3562 | 5.0957 | 4.7500 | 5.4961 |
| 2 | | 28.4853 | 28.2934 | 28.6772 | 4.7215 | 4.5896 | 4.8612 |
| Diff (1-2) | Pooled | 0.3450 | -0.1833 | 0.8732 | 4.7736 | 4.6493 | 4.9047 |
| Diff (1-2) | Satterthwaite | 0.3450 | -0.2147 | 0.9046 | | | |

| Method | Variances | DF | t Value | Pr > \|t\| |
|---|---|---|---|---|
| Pooled | Equal | 2688 | 1.28 | 0.2005 |
| Satterthwaite | Unequal | 464.16 | 1.21 | 0.2264 |

| Equality of Variances | | | | |
|---|---|---|---|---|
| Method | Num DF | Den DF | F Value | Pr > F |
| Folded F | 362 | 2326 | 1.16 | 0.0496 |

## The TTEST Procedure

### Variable: BMI (BMI)

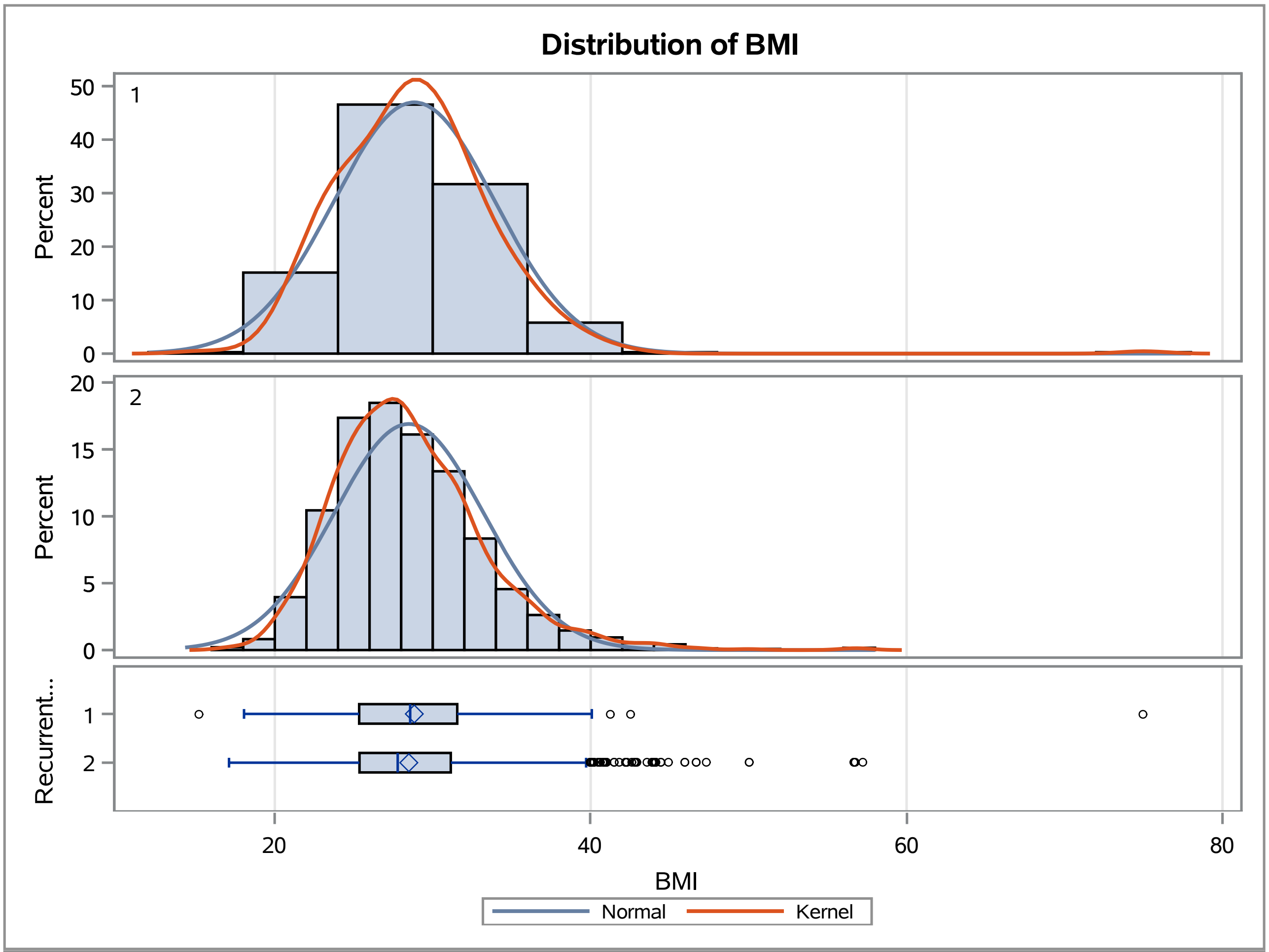


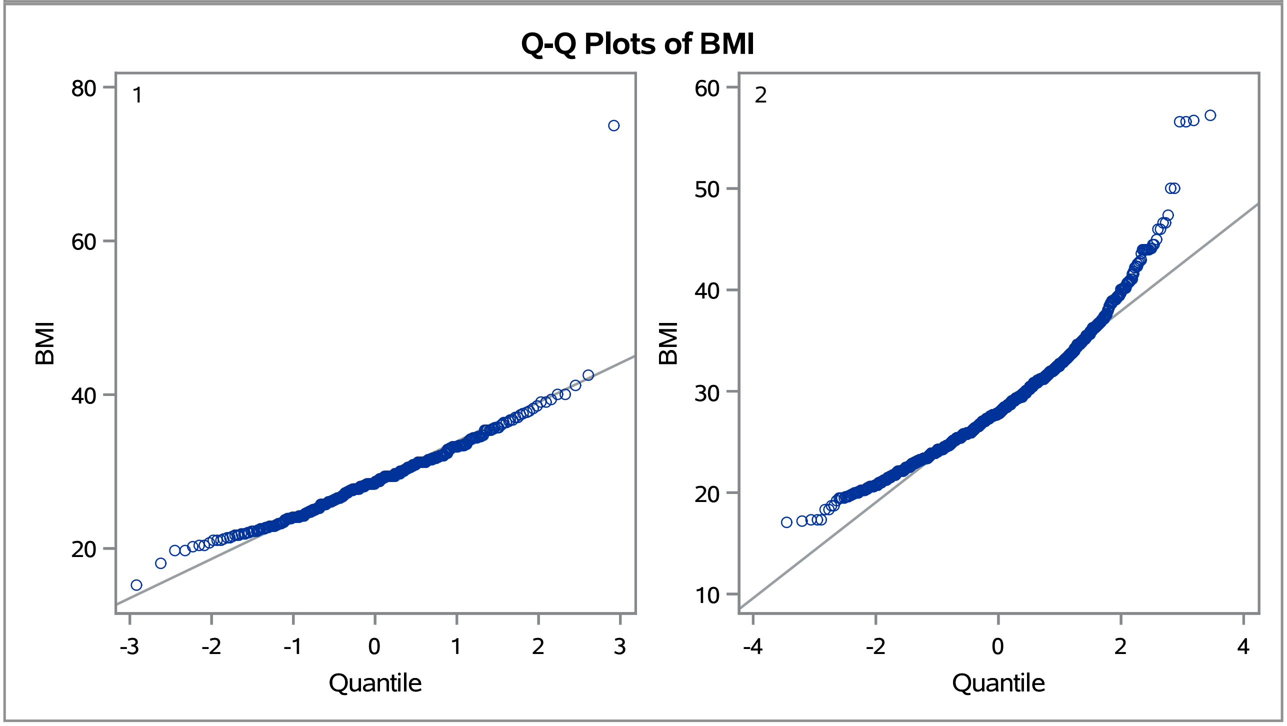

## The TTEST Procedure

### Variable: HR_bpm_ (HR(bpm))

### Variable: HR_bpm_ (HR(bpm))

| Recurrent_ischemia | Method | N | Mean | Std Dev | Std Err | Minimum | Maximum |
|---|---|---|---|---|---|---|---|
| **1** | | 363 | 88.6556 | 20.1859 | 1.0595 | 40.0000 | 230.0 |
| **2** | | 2326 | 83.4630 | 17.2403 | 0.3575 | 10.0000 | 196.0 |
| **Diff (1-2)** | **Pooled** | | 5.1926 | 17.6658 | 0.9969 | | |
| **Diff (1-2)** | **Satterthwaite** | | 5.1926 | | 1.1182 | | |

| Recurrent_ischemia | Method | Mean | 95% CL Mean | | Std Dev | 95% CL Std Dev | |
|---|---|---|---|---|---|---|---|
| **1** | | 88.6556 | 86.5721 | 90.7392 | 20.1859 | 18.8165 | 21.7718 |
| **2** | | 83.4630 | 82.7620 | 84.1640 | 17.2403 | 16.7588 | 17.7506 |
| **Diff (1-2)** | **Pooled** | 5.1926 | 3.2378 | 7.1475 | 17.6658 | 17.2059 | 18.1512 |
| **Diff (1-2)** | **Satterthwaite** | 5.1926 | 2.9951 | 7.3901 | | | |

| Method | Variances | DF | t Value | Pr > \|t\| |
|---|---|---|---|---|
| **Pooled** | Equal | 2687 | 5.21 | <.0001 |
| **Satterthwaite** | Unequal | 448.21 | 4.64 | <.0001 |

| Equality of Variances | | | | |
|---|---|---|---|---|
| **Method** | **Num DF** | **Den DF** | **F Value** | **Pr > F** |
| **Folded F** | 362 | 2325 | 1.37 | <.0001 |

The TTEST Procedure

Variable: HR_bpm_ (HR(bpm))

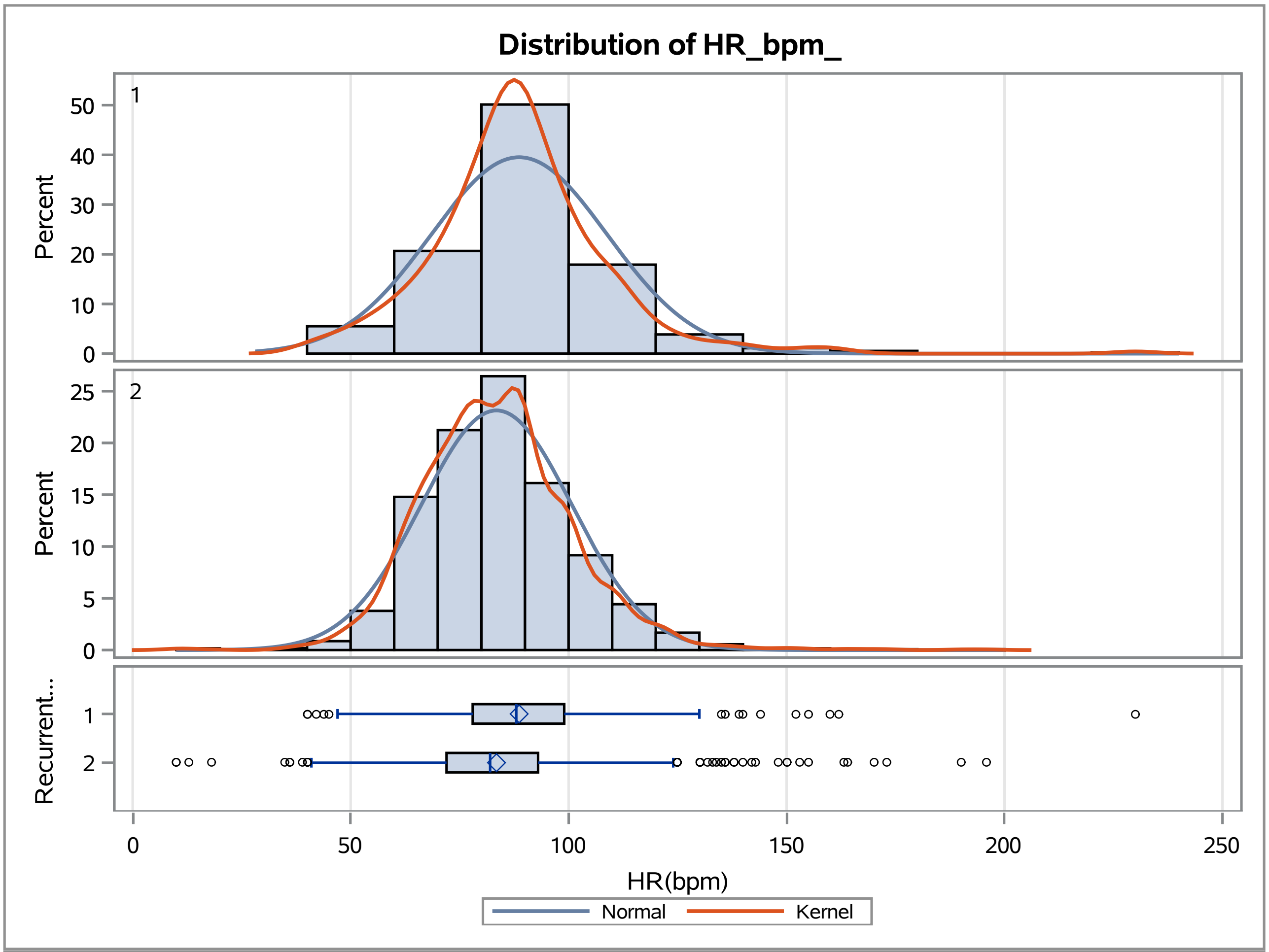


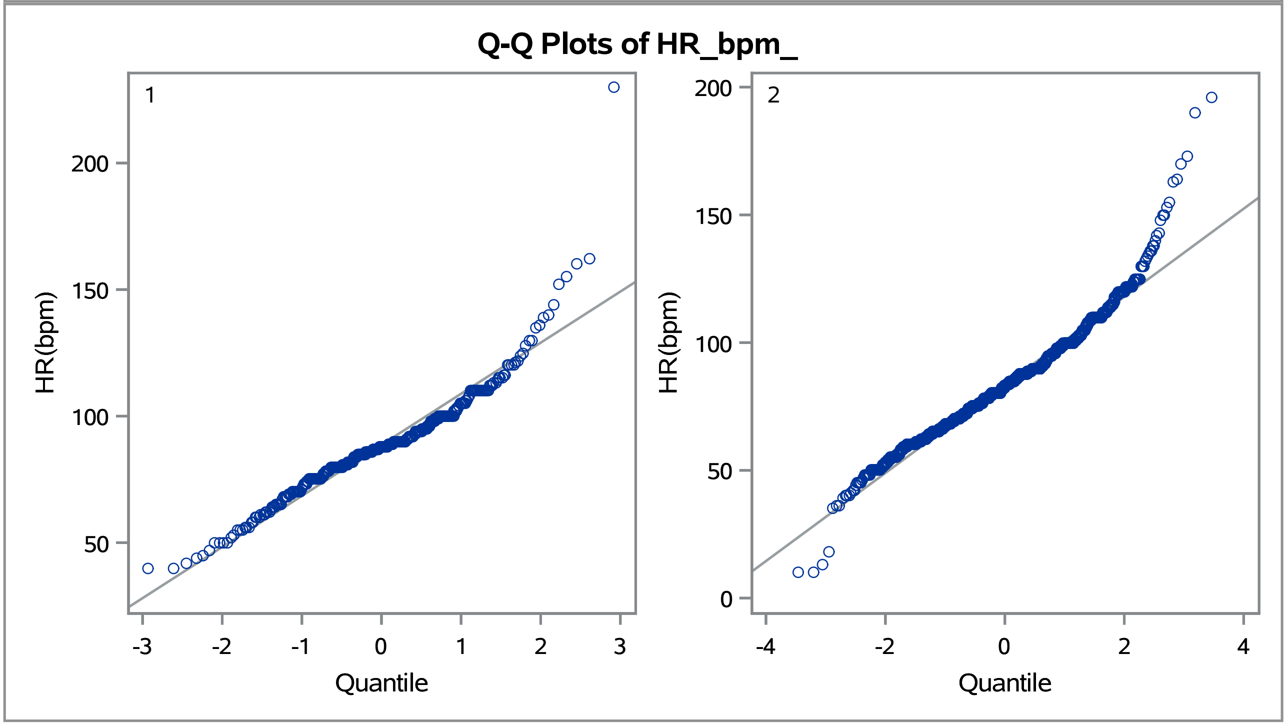

## The TTEST Procedure

### Variable: SBP_mmgH_ (SBP(mmgH))

Variable: SBP_mmgH_ (SBP(mmgH))

| Recurrent_ischemia | Method | N | Mean | Std Dev | Std Err | Minimum | Maximum |
|---|---|---|---|---|---|---|---|
| 1 | | 363 | 139.5 | 27.3135 | 1.4336 | 70.0000 | 220.0 |
| 2 | | 2326 | 134.2 | 25.0828 | 0.5201 | 40.0000 | 244.0 |
| Diff (1-2) | Pooled | | 5.3379 | 25.3947 | 1.4331 | | |
| Diff (1-2) | Satterthwaite | | 5.3379 | | 1.5250 | | |

| Recurrent_ischemia | Method | Mean | 95% CL Mean | | Std Dev | 95% CL Std Dev | |
|---|---|---|---|---|---|---|---|
| 1 | | 139.5 | 136.7 | 142.3 | 27.3135 | 25.4607 | 29.4595 |
| 2 | | 134.2 | 133.1 | 135.2 | 25.0828 | 24.3822 | 25.8252 |
| Diff (1-2) | Pooled | 5.3379 | 2.5278 | 8.1480 | 25.3947 | 24.7336 | 26.0924 |
| Diff (1-2) | Satterthwaite | 5.3379 | 2.3411 | 8.3347 | | | |

| Method | Variances | DF | t Value | Pr > \|t\| |
|---|---|---|---|---|
| Pooled | Equal | 2687 | 3.72 | 0.0002 |
| Satterthwaite | Unequal | 462.31 | 3.50 | 0.0005 |

| Equality of Variances | | | | |
|---|---|---|---|---|
| Method | Num DF | Den DF | F Value | Pr > F |
| Folded F | 362 | 2325 | 1.19 | 0.0281 |

The TTEST Procedure

Variable: SBP_mmgH_ (SBP(mmgH))

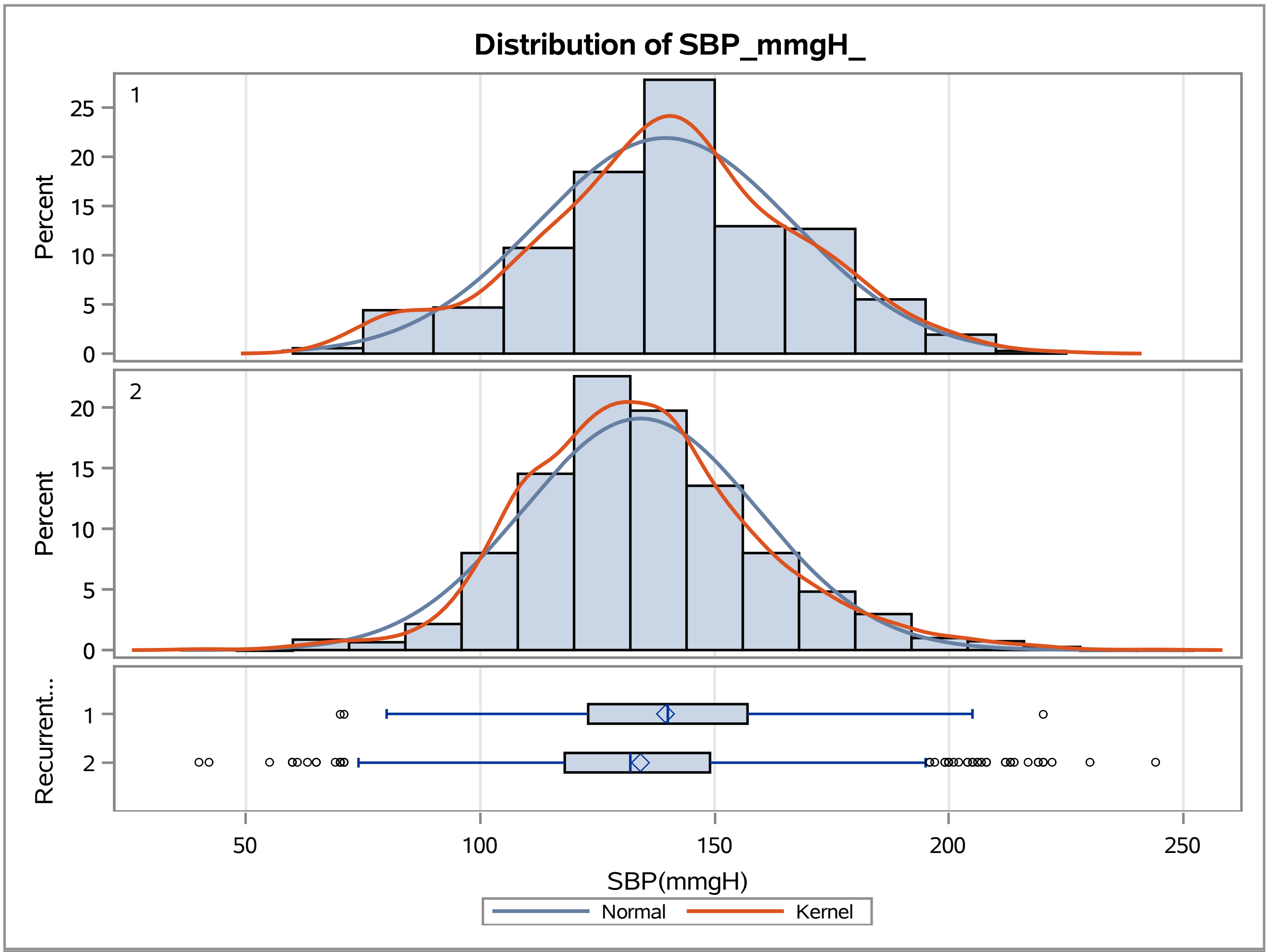


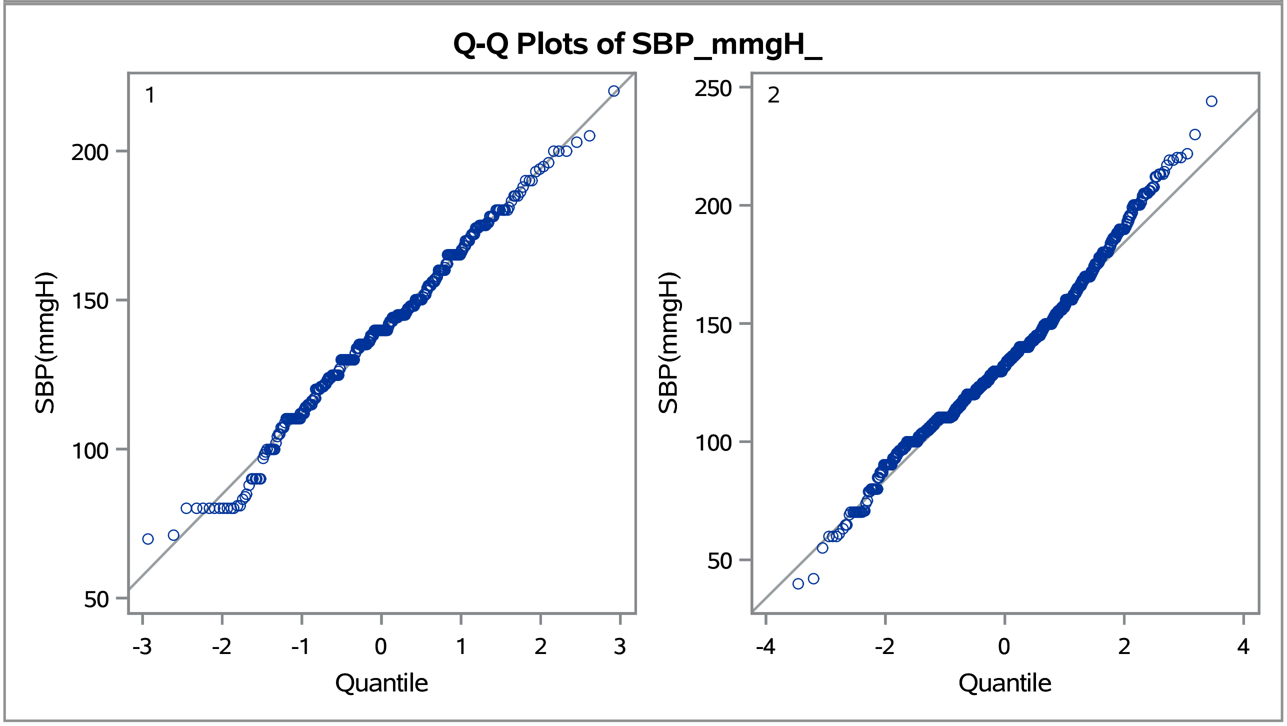

## The FREQ Procedure

| Recurrent ischemia | | | | |
|---|---|---|---|---|
| Recurrent_ischemia | Frequency | Percent | Cumulative Frequency | Cumulative Percent |
| 1 | 363 | 13.49 | 363 | 13.49 |
| 2 | 2327 | 86.51 | 2690 | 100.00 |
| Frequency Missing = 1 | | | | |

## The LOGISTIC Procedure

| Model Information | | |
|---|---|---|
| **Data Set** | WAEL.STARS4 | |
| **Response Variable** | Recurrent_ischemia | Recurrent ischemia |
| **Number of Response Levels** | 2 | |
| **Model** | binary logit | |
| **Optimization Technique** | Fisher's scoring | |

| | |
|---|---|
| **Number of Observations Read** | 2691 |
| **Number of Observations Used** | 2690 |

| Response Profile | | |
|---|---|---|
| **Ordered Value** | **Recurrent_ischemia** | **Total Frequency** |
| **1** | 1 | 363 |
| **2** | 2 | 2327 |

**Probability modeled is Recurrent_ischemia='1'.**

**Note:** 1 observation was deleted due to missing values for the response or explanatory variables.

| Class Level Information | | |
|---|---|---|
| **Class** | **Value** | **Design Variables** |
| **Gender** | **1** | 0 |
| | **2** | 1 |

| Model Convergence Status |
|---|
| Convergence criterion (GCONV=1E-8) satisfied. |

| Model Fit Statistics | | |
|---|---|---|
| **Criterion** | **Intercept Only** | **Intercept and Covariates** |
| **AIC** | 2130.751 | 2122.836 |
| **SC** | 2136.648 | 2134.630 |
| **-2 Log L** | 2128.751 | 2118.836 |

| Testing Global Null Hypothesis: BETA=0 | | | |
|---|---|---|---|
| **Test** | **Chi-Square** | **DF** | **Pr > ChiSq** |
| **Likelihood Ratio** | 9.9149 | 1 | 0.0016 |
| **Score** | 10.5846 | 1 | 0.0011 |
| **Wald** | 10.4597 | 1 | 0.0012 |

## The LOGISTIC Procedure

| Type 3 Analysis of Effects | | | |
|---|---|---|---|
| Effect | DF | Wald Chi-Square | Pr > ChiSq |
| Gender | 1 | 10.4597 | 0.0012 |

| Analysis of Maximum Likelihood Estimates | | | | | | |
|---|---|---|---|---|---|---|
| Parameter | | DF | Estimate | Standard Error | Wald Chi-Square | Pr > ChiSq |
| Intercept | | 1 | -1.9464 | 0.0643 | 915.0013 | <.0001 |
| Gender | 2 | 1 | 0.4360 | 0.1348 | 10.4597 | 0.0012 |

| Odds Ratio Estimates | | | |
|---|---|---|---|
| Effect | Point Estimate | 95% Wald Confidence Limits | |
| Gender 2 vs 1 | 1.546 | 1.187 | 2.014 |

| Association of Predicted Probabilities and Observed Responses | | | |
|---|---|---|---|
| Percent Concordant | 19.9 | Somers' D | 0.070 |
| Percent Discordant | 12.9 | Gamma | 0.215 |
| Percent Tied | 67.2 | Tau-a | 0.016 |
| Pairs | 844701 | c | 0.535 |

## The LOGISTIC Procedure

| Model Information | | |
|---|---|---|
| **Data Set** | WAEL.STARS4 | |
| **Response Variable** | Recurrent_ischemia | Recurrent ischemia |
| **Number of Response Levels** | 2 | |
| **Model** | binary logit | |
| **Optimization Technique** | Fisher's scoring | |

| | |
|---|---|
| **Number of Observations Read** | 2691 |
| **Number of Observations Used** | 2384 |

| Response Profile | | |
|---|---|---|
| **Ordered Value** | **Recurrent_ischemia** | **Total Frequency** |
| **1** | 1 | 310 |
| **2** | 2 | 2074 |

**Probability modeled is Recurrent_ischemia='1'.**

**Note:** 307 observations were deleted due to missing values for the response or explanatory variables.

| Class Level Information | | | | |
|---|---|---|---|---|
| **Class** | **Value** | **Design Variables** | | |
| **Gender** | **1** | 0 | | |
| | **2** | 1 | | |
| **Hypercholestrolemia** | **1** | 0 | | |
| | **2** | 1 | | |
| **CAD** | **1** | 1 | | |
| | **2** | 0 | | |
| **History_of_heart_failure** | **1** | 0 | | |
| | **2** | 1 | | |
| **History_of_chronic_renal_failure** | **1** | 0 | | |
| | **2** | 1 | | |
| **DM** | **1** | 0 | | |
| | **2** | 1 | | |
| **HTN** | **1** | 0 | | |
| | **2** | 1 | | |
| **CHF_Killip_Class** | **1** | 0 | 0 | 0 |
| | **2** | 1 | 0 | 0 |
| | **3** | 0 | 1 | 0 |

## The LOGISTIC Procedure

| Class Level Information | | | | |
|---|---|---|---|---|
| Class | Value | Design Variables | | |
| | 4 | 0 | 0 | 1 |
| Cardiac_arrest | 1 | 0 | | |
| | 2 | 1 | | |
| Echo_Options | 1 | 0 | 0 | 0 |
| | 2 | 1 | 0 | 0 |
| | 3 | 0 | 1 | 0 |
| | 4 | 0 | 0 | 1 |

| Model Convergence Status |
|---|
| Convergence criterion (GCONV=1E-8) satisfied. |

| Model Fit Statistics | | |
|---|---|---|
| Criterion | Intercept Only | Intercept and Covariates |
| AIC | 1844.596 | 1688.687 |
| SC | 1850.372 | 1792.664 |
| -2 Log L | 1842.596 | 1652.687 |

| Testing Global Null Hypothesis: BETA=0 | | | |
|---|---|---|---|
| Test | Chi-Square | DF | Pr > ChiSq |
| Likelihood Ratio | 189.9093 | 17 | <.0001 |
| Score | 221.4511 | 17 | <.0001 |
| Wald | 180.9084 | 17 | <.0001 |

| Type 3 Analysis of Effects | | | |
|---|---|---|---|
| Effect | DF | Wald Chi-Square | Pr > ChiSq |
| Gender | 1 | 3.0010 | 0.0832 |
| History_of_heart_fai | 1 | 7.1318 | 0.0076 |
| History_of_chronic_r | 1 | 0.2662 | 0.6059 |
| DM | 1 | 0.2626 | 0.6083 |
| HTN | 1 | 0.0014 | 0.9706 |
| CHF_Killip_Class | 3 | 50.8557 | <.0001 |
| Age | 1 | 0.6317 | 0.4267 |
| SBP_mmgH_ | 1 | 18.8767 | <.0001 |

## The LOGISTIC Procedure

| Type 3 Analysis of Effects | | | |
|---|---|---|---|
| Effect | DF | Wald Chi-Square | Pr > ChiSq |
| HR_bpm_ | 1 | 1.3531 | 0.2447 |
| CAD | 1 | 14.1189 | 0.0002 |
| Hypercholestrolemia | 1 | 10.6674 | 0.0011 |
| Cardiac_arrest | 1 | 3.8436 | 0.0499 |
| Echo_Options | 3 | 7.4908 | 0.0578 |

| Analysis of Maximum Likelihood Estimates | | | | | | |
|---|---|---|---|---|---|---|
| Parameter | | DF | Estimate | Standard Error | Wald Chi-Square | Pr > ChiSq |
| Intercept | | 1 | -2.3301 | 0.7137 | 10.6573 | 0.0011 |
| Gender | 2 | 1 | 0.2762 | 0.1595 | 3.0010 | 0.0832 |
| History_of_heart_fai | 2 | 1 | -0.6318 | 0.2366 | 7.1318 | 0.0076 |
| History_of_chronic_r | 2 | 1 | -0.1110 | 0.2152 | 0.2662 | 0.6059 |
| DM | 2 | 1 | -0.0755 | 0.1474 | 0.2626 | 0.6083 |
| HTN | 2 | 1 | 0.00581 | 0.1578 | 0.0014 | 0.9706 |
| CHF_Killip_Class | 2 | 1 | 1.3168 | 0.2001 | 43.2999 | <.0001 |
| CHF_Killip_Class | 3 | 1 | 1.0496 | 0.2820 | 13.8573 | 0.0002 |
| CHF_Killip_Class | 4 | 1 | 1.3799 | 0.4653 | 8.7932 | 0.0030 |
| Age | | 1 | -0.00462 | 0.00582 | 0.6317 | 0.4267 |
| SBP_mmgH_ | | 1 | 0.0109 | 0.00251 | 18.8767 | <.0001 |
| HR_bpm_ | | 1 | 0.00427 | 0.00367 | 1.3531 | 0.2447 |
| CAD | 1 | 1 | 0.5283 | 0.1406 | 14.1189 | 0.0002 |
| Hypercholestrolemia | 2 | 1 | -0.4459 | 0.1365 | 10.6674 | 0.0011 |
| Cardiac_arrest | 2 | 1 | -0.6886 | 0.3512 | 3.8436 | 0.0499 |
| Echo_Options | 2 | 1 | 0.0880 | 0.1551 | 0.3219 | 0.5704 |
| Echo_Options | 3 | 1 | -0.3079 | 0.1961 | 2.4659 | 0.1163 |
| Echo_Options | 4 | 1 | -0.5098 | 0.2727 | 3.4942 | 0.0616 |

| Odds Ratio Estimates | | | |
|---|---|---|---|
| Effect | Point Estimate | 95% Wald Confidence Limits | |
| Gender 2 vs 1 | 1.318 | 0.964 | 1.802 |
| History_of_heart_fai 2 vs 1 | 0.532 | 0.334 | 0.845 |
| History_of_chronic_r 2 vs 1 | 0.895 | 0.587 | 1.365 |
| DM 2 vs 1 | 0.927 | 0.695 | 1.238 |
| HTN 2 vs 1 | 1.006 | 0.738 | 1.370 |

## The LOGISTIC Procedure

| Odds Ratio Estimates | | | |
|---|---|---|---|
| Effect | Point Estimate | 95% Wald Confidence Limits | |
| CHF_Killip_Class 2 vs 1 | 3.731 | 2.521 | 5.523 |
| CHF_Killip_Class 3 vs 1 | 2.857 | 1.644 | 4.964 |
| CHF_Killip_Class 4 vs 1 | 3.974 | 1.597 | 9.894 |
| Age | 0.995 | 0.984 | 1.007 |
| SBP_mmgH_ | 1.011 | 1.006 | 1.016 |
| HR_bpm_ | 1.004 | 0.997 | 1.012 |
| CAD 1 vs 2 | 1.696 | 1.288 | 2.234 |
| Hypercholestrolemia 2 vs 1 | 0.640 | 0.490 | 0.837 |
| Cardiac_arrest 2 vs 1 | 0.502 | 0.252 | 1.000 |
| Echo_Options 2 vs 1 | 1.092 | 0.806 | 1.480 |
| Echo_Options 3 vs 1 | 0.735 | 0.500 | 1.079 |
| Echo_Options 4 vs 1 | 0.601 | 0.352 | 1.025 |

| Association of Predicted Probabilities and Observed Responses | | | |
|---|---|---|---|
| Percent Concordant | 72.4 | Somers' D | 0.447 |
| Percent Discordant | 27.6 | Gamma | 0.447 |
| Percent Tied | 0.0 | Tau-a | 0.101 |
| Pairs | 642940 | c | 0.724 |

## The FREQ Procedure

| Frequency<br>Percent<br>Row Pct<br>Col Pct |
|---|

| Table of Heart_Failure by Gender | | | |
|---|---|---|---|
| Heart_Failure(Heart Failure) | Gender(Gender) | | |
| | 1 | 2 | Total |
| 1 | 248<br>9.22<br>77.02<br>11.23 | 74<br>2.75<br>22.98<br>15.38 | 322<br>11.97 |
| 2 | 1961<br>72.90<br>82.81<br>88.77 | 407<br>15.13<br>17.19<br>84.62 | 2368<br>88.03 |
| Total | 2209<br>82.12 | 481<br>17.88 | 2690<br>100.00 |
| Frequency Missing = 1 | | | |

## Statistics for Table of Heart_Failure by Gender

| Statistic | DF | Value | Prob |
|---|---|---|---|
| Chi-Square | 1 | 6.4802 | 0.0109 |
| Likelihood Ratio Chi-Square | 1 | 6.1190 | 0.0134 |
| Continuity Adj. Chi-Square | 1 | 6.0916 | 0.0136 |
| Mantel-Haenszel Chi-Square | 1 | 6.4778 | 0.0109 |
| Phi Coefficient | | -0.0491 | |
| Contingency Coefficient | | 0.0490 | |
| Cramer's V | | -0.0491 | |

| Fisher's Exact Test | |
|---|---|
| Cell (1,1) Frequency (F) | 248 |
| Left-sided Pr <= F | 0.0079 |
| Right-sided Pr >= F | 0.9947 |
| | |
| Table Probability (P) | 0.0027 |
| Two-sided Pr <= P | 0.0130 |

**Sample Size = 2690**
**Frequency Missing = 1**

## The FREQ Procedure

| Frequency<br>Percent<br>Row Pct<br>Col Pct |
|---|

| Table of Heart_Failure by Nationality | | | |
|---|---|---|---|
| Heart_Failure(Heart Failure) | Nationality(Nationality) | | |
| | 1 | 2 | Total |
| 1 | 228<br>8.48<br>70.81<br>12.24 | 94<br>3.49<br>29.19<br>11.35 | 322<br>11.97 |
| 2 | 1634<br>60.74<br>69.00<br>87.76 | 734<br>27.29<br>31.00<br>88.65 | 2368<br>88.03 |
| Total | 1862<br>69.22 | 828<br>30.78 | 2690<br>100.00 |
| Frequency Missing = 1 | | | |

## Statistics for Table of Heart_Failure by Nationality

| Statistic | DF | Value | Prob |
|---|---|---|---|
| Chi-Square | 1 | 0.4330 | 0.5105 |
| Likelihood Ratio Chi-Square | 1 | 0.4367 | 0.5087 |
| Continuity Adj. Chi-Square | 1 | 0.3525 | 0.5527 |
| Mantel-Haenszel Chi-Square | 1 | 0.4328 | 0.5106 |
| Phi Coefficient | | 0.0127 | |
| Contingency Coefficient | | 0.0127 | |
| Cramer's V | | 0.0127 | |

| Fisher's Exact Test | |
|---|---|
| Cell (1,1) Frequency (F) | 228 |
| Left-sided Pr <= F | 0.7640 |
| Right-sided Pr >= F | 0.2778 |
| | |
| Table Probability (P) | 0.0418 |
| Two-sided Pr <= P | 0.5626 |

**Sample Size = 2690**
**Frequency Missing = 1**

## The FREQ Procedure

| Frequency<br>Percent<br>Row Pct<br>Col Pct |
|---|

| Table of Heart_Failure by Ethnicity | | | | |
|---|---|---|---|---|
| | Ethnicity(Ethnicity) | | | |
| Heart_Failure(Heart Failure) | 1 | 2 | 3 | Total |
| 1 | 257<br>9.55<br>79.81<br>12.24 | 55<br>2.04<br>17.08<br>10.89 | 10<br>0.37<br>3.11<br>11.63 | 322<br>11.97 |
| 2 | 1842<br>68.48<br>77.79<br>87.76 | 450<br>16.73<br>19.00<br>89.11 | 76<br>2.83<br>3.21<br>88.37 | 2368<br>88.03 |
| Total | 2099<br>78.03 | 505<br>18.77 | 86<br>3.20 | 2690<br>100.00 |
| Frequency Missing = 1 | | | | |

## Statistics for Table of Heart_Failure by Ethnicity

| Statistic | DF | Value | Prob |
|---|---|---|---|
| Chi-Square | 2 | 0.7169 | 0.6988 |
| Likelihood Ratio Chi-Square | 2 | 0.7312 | 0.6938 |
| Mantel-Haenszel Chi-Square | 1 | 0.5098 | 0.4752 |
| Phi Coefficient | | 0.0163 | |
| Contingency Coefficient | | 0.0163 | |
| Cramer's V | | 0.0163 | |

**Sample Size = 2690**
**Frequency Missing = 1**

| Frequency<br>Percent<br>Row Pct<br>Col Pct |
|---|

| Table of Heart_Failure by Type_of_STEMI | | | | |
|---|---|---|---|---|
| | Type_of_STEMI(Type of STEMI) | | | |
| Heart_Failure(Heart Failure) | 1 | 2 | 3 | Total |
| 1 | 102<br>7.77<br>72.86<br>14.78 | 31<br>2.36<br>22.14<br>5.69 | 7<br>0.53<br>5.00<br>8.97 | 140<br>10.66 |
| 2 | 588<br>44.78<br>50.13<br>85.22 | 514<br>39.15<br>43.82<br>94.31 | 71<br>5.41<br>6.05<br>91.03 | 1173<br>89.34 |
| Total | 690<br>52.55 | 545<br>41.51 | 78<br>5.94 | 1313<br>100.00 |
| Frequency Missing = 1378 | | | | |

## The FREQ Procedure

### Statistics for Table of Heart_Failure by Type_of_STEMI

| Statistic | DF | Value | Prob |
|---|---|---|---|
| Chi-Square | 2 | 26.6870 | <.0001 |
| Likelihood Ratio Chi-Square | 2 | 28.1151 | <.0001 |
| Mantel-Haenszel Chi-Square | 1 | 19.2257 | <.0001 |
| Phi Coefficient | | 0.1426 | |
| Contingency Coefficient | | 0.1411 | |
| Cramer's V | | 0.1426 | |

**Sample Size = 1313**
**Frequency Missing = 1378**

**WARNING: 51% of the data are missing.**

| Frequency<br>Percent<br>Row Pct<br>Col Pct |
|---|

| Table of Heart_Failure by History_of_angina | | | |
|---|---|---|---|
| Heart_Failure(Heart Failure) | History_of_angina(History of angina) | | |
| | 1 | 2 | Total |
| 1 | 146<br>5.43<br>45.34<br>19.73 | 176<br>6.54<br>54.66<br>9.03 | 322<br>11.97 |
| 2 | 594<br>22.08<br>25.08<br>80.27 | 1774<br>65.95<br>74.92<br>90.97 | 2368<br>88.03 |
| Total | 740<br>27.51 | 1950<br>72.49 | 2690<br>100.00 |
| Frequency Missing = 1 | | | |

## The FREQ Procedure

### Statistics for Table of Heart_Failure by History_of_angina

| Statistic | DF | Value | Prob |
|---|---|---|---|
| Chi-Square | 1 | 58.3285 | <.0001 |
| Likelihood Ratio Chi-Square | 1 | 53.6402 | <.0001 |
| Continuity Adj. Chi-Square | 1 | 57.3171 | <.0001 |
| Mantel-Haenszel Chi-Square | 1 | 58.3068 | <.0001 |
| Phi Coefficient | | 0.1473 | |
| Contingency Coefficient | | 0.1457 | |
| Cramer's V | | 0.1473 | |

| Fisher's Exact Test | |
|---|---|
| Cell (1,1) Frequency (F) | 146 |
| Left-sided Pr <= F | 1.0000 |
| Right-sided Pr >= F | <.0001 |
| | |
| Table Probability (P) | <.0001 |
| Two-sided Pr <= P | <.0001 |

**Sample Size = 2690**
**Frequency Missing = 1**

| Frequency<br>Percent<br>Row Pct<br>Col Pct |
|---|

| Table of Heart_Failure by History_of_MI | | | |
|---|---|---|---|
| Heart_Failure(Heart Failure) | History_of_MI(History of MI) | | |
| | 1 | 2 | Total |
| 1 | 97<br>3.61<br>30.12<br>24.07 | 225<br>8.36<br>69.88<br>9.84 | 322<br>11.97 |
| 2 | 306<br>11.38<br>12.92<br>75.93 | 2062<br>76.65<br>87.08<br>90.16 | 2368<br>88.03 |
| Total | 403<br>14.98 | 2287<br>85.02 | 2690<br>100.00 |
| Frequency Missing = 1 | | | |

## The FREQ Procedure

### Statistics for Table of Heart_Failure by History_of_MI

| Statistic | DF | Value | Prob |
|---|---|---|---|
| Chi-Square | 1 | 65.8526 | <.0001 |
| Likelihood Ratio Chi-Square | 1 | 55.4482 | <.0001 |
| Continuity Adj. Chi-Square | 1 | 64.5090 | <.0001 |
| Mantel-Haenszel Chi-Square | 1 | 65.8281 | <.0001 |
| Phi Coefficient | | 0.1565 | |
| Contingency Coefficient | | 0.1546 | |
| Cramer's V | | 0.1565 | |

| Fisher's Exact Test | |
|---|---|
| Cell (1,1) Frequency (F) | 97 |
| Left-sided Pr <= F | 1.0000 |
| Right-sided Pr >= F | <.0001 |
| | |
| Table Probability (P) | <.0001 |
| Two-sided Pr <= P | <.0001 |

**Sample Size = 2690**
**Frequency Missing = 1**

| Frequency<br>Percent<br>Row Pct<br>Col Pct |
|---|

| Table of Heart_Failure by History_of_PCI | | | |
|---|---|---|---|
| Heart_Failure(Heart Failure) | History_of_PCI(History of PCI) | | |
| | 1 | 2 | Total |
| 1 | 66<br>2.45<br>20.50<br>15.75 | 256<br>9.52<br>79.50<br>11.27 | 322<br>11.97 |
| 2 | 353<br>13.12<br>14.91<br>84.25 | 2015<br>74.91<br>85.09<br>88.73 | 2368<br>88.03 |
| Total | 419<br>15.58 | 2271<br>84.42 | 2690<br>100.00 |
| Frequency Missing = 1 | | | |

## The FREQ Procedure

### Statistics for Table of Heart_Failure by History_of_PCI

| Statistic | DF | Value | Prob |
|---|---|---|---|
| Chi-Square | 1 | 6.7352 | 0.0095 |
| Likelihood Ratio Chi-Square | 1 | 6.3064 | 0.0120 |
| Continuity Adj. Chi-Square | 1 | 6.3168 | 0.0120 |
| Mantel-Haenszel Chi-Square | 1 | 6.7327 | 0.0095 |
| Phi Coefficient | | 0.0500 | |
| Contingency Coefficient | | 0.0500 | |
| Cramer's V | | 0.0500 | |

| Fisher's Exact Test | |
|---|---|
| Cell (1,1) Frequency (F) | 66 |
| Left-sided Pr <= F | 0.9954 |
| Right-sided Pr >= F | 0.0072 |
| | |
| Table Probability (P) | 0.0025 |
| Two-sided Pr <= P | 0.0111 |

**Sample Size = 2690**
**Frequency Missing = 1**

| Frequency<br>Percent<br>Row Pct<br>Col Pct |
|---|

| Table of Heart_Failure by History_of_CABG | | | |
|---|---|---|---|
| Heart_Failure(Heart Failure) | History_of_CABG(History of CABG) | | |
| | 1 | 2 | Total |
| 1 | 25<br>0.93<br>7.76<br>35.21 | 297<br>11.04<br>92.24<br>11.34 | 322<br>11.97 |
| 2 | 46<br>1.71<br>1.94<br>64.79 | 2322<br>86.32<br>98.06<br>88.66 | 2368<br>88.03 |
| Total | 71<br>2.64 | 2619<br>97.36 | 2690<br>100.00 |
| Frequency Missing = 1 | | | |

## The FREQ Procedure

### Statistics for Table of Heart_Failure by History_of_CABG

| Statistic | DF | Value | Prob |
|---|---|---|---|
| Chi-Square | 1 | 37.3811 | <.0001 |
| Likelihood Ratio Chi-Square | 1 | 26.7471 | <.0001 |
| Continuity Adj. Chi-Square | 1 | 35.1500 | <.0001 |
| Mantel-Haenszel Chi-Square | 1 | 37.3672 | <.0001 |
| Phi Coefficient | | 0.1179 | |
| Contingency Coefficient | | 0.1171 | |
| Cramer's V | | 0.1179 | |

| Fisher's Exact Test | |
|---|---|
| Cell (1,1) Frequency (F) | 25 |
| Left-sided Pr <= F | 1.0000 |
| Right-sided Pr >= F | <.0001 |
| | |
| Table Probability (P) | <.0001 |
| Two-sided Pr <= P | <.0001 |

**Sample Size = 2690**
**Frequency Missing = 1**

| Frequency<br>Percent<br>Row Pct<br>Col Pct |
|---|

| Table of Heart_Failure by History_of_heart_failure | | | |
|---|---|---|---|
| Heart_Failure(Heart Failure) | History_of_heart_failure(History of heart failure) | | |
| | 1 | 2 | Total |
| 1 | 98<br>3.64<br>30.43<br>60.12 | 224<br>8.33<br>69.57<br>8.86 | 322<br>11.97 |
| 2 | 65<br>2.42<br>2.74<br>39.88 | 2303<br>85.61<br>97.26<br>91.14 | 2368<br>88.03 |
| Total | 163<br>6.06 | 2527<br>93.94 | 2690<br>100.00 |
| Frequency Missing = 1 | | | |

## The FREQ Procedure

### Statistics for Table of Heart_Failure by History_of_heart_failure

| Statistic | DF | Value | Prob |
|---|---|---|---|
| Chi-Square | 1 | 381.8020 | <.0001 |
| Likelihood Ratio Chi-Square | 1 | 238.5302 | <.0001 |
| Continuity Adj. Chi-Square | 1 | 376.9531 | <.0001 |
| Mantel-Haenszel Chi-Square | 1 | 381.6601 | <.0001 |
| Phi Coefficient | | 0.3767 | |
| Contingency Coefficient | | 0.3526 | |
| Cramer's V | | 0.3767 | |

| Fisher's Exact Test | |
|---|---|
| Cell (1,1) Frequency (F) | 98 |
| Left-sided Pr <= F | 1.0000 |
| Right-sided Pr >= F | <.0001 |
| | |
| Table Probability (P) | <.0001 |
| Two-sided Pr <= P | <.0001 |

**Sample Size = 2690**
**Frequency Missing = 1**

| Frequency<br>Percent<br>Row Pct<br>Col Pct |
|---|

| Table of Heart_Failure by History_of_stroke | | | |
|---|---|---|---|
| Heart_Failure(Heart Failure) | History_of_stroke(History of stroke) | | |
| | 1 | 2 | Total |
| 1 | 27<br>1.00<br>8.39<br>21.95 | 295<br>10.97<br>91.61<br>11.49 | 322<br>11.97 |
| 2 | 96<br>3.57<br>4.05<br>78.05 | 2272<br>84.46<br>95.95<br>88.51 | 2368<br>88.03 |
| Total | 123<br>4.57 | 2567<br>95.43 | 2690<br>100.00 |
| Frequency Missing = 1 | | | |

## The FREQ Procedure

### Statistics for Table of Heart_Failure by History_of_stroke

| Statistic | DF | Value | Prob |
|---|---|---|---|
| Chi-Square | 1 | 12.1855 | 0.0005 |
| Likelihood Ratio Chi-Square | 1 | 10.2038 | 0.0014 |
| Continuity Adj. Chi-Square | 1 | 11.2131 | 0.0008 |
| Mantel-Haenszel Chi-Square | 1 | 12.1810 | 0.0005 |
| Phi Coefficient | | 0.0673 | |
| Contingency Coefficient | | 0.0672 | |
| Cramer's V | | 0.0673 | |

| Fisher's Exact Test | |
|---|---|
| Cell (1,1) Frequency (F) | 27 |
| Left-sided Pr <= F | 0.9996 |
| Right-sided Pr >= F | 0.0010 |
| | |
| Table Probability (P) | 0.0005 |
| Two-sided Pr <= P | 0.0015 |

**Sample Size = 2690**
**Frequency Missing = 1**

| Frequency<br>Percent<br>Row Pct<br>Col Pct |
|---|

| Table of Heart_Failure by History_of_chronic_renal_failure | | | |
|---|---|---|---|
| Heart_Failure(Heart Failure) | History_of_chronic_renal_failure(History of chronic renal failure) | | |
| | 1 | 2 | Total |
| 1 | 77<br>2.86<br>23.91<br>38.50 | 245<br>9.11<br>76.09<br>9.84 | 322<br>11.97 |
| 2 | 123<br>4.57<br>5.19<br>61.50 | 2245<br>83.46<br>94.81<br>90.16 | 2368<br>88.03 |
| Total | 200<br>7.43 | 2490<br>92.57 | 2690<br>100.00 |
| Frequency Missing = 1 | | | |

## The FREQ Procedure

### Statistics for Table of Heart_Failure by History_of_chronic_renal_failure

| Statistic | DF | Value | Prob |
|---|---|---|---|
| Chi-Square | 1 | 144.3165 | <.0001 |
| Likelihood Ratio Chi-Square | 1 | 103.0191 | <.0001 |
| Continuity Adj. Chi-Square | 1 | 141.6094 | <.0001 |
| Mantel-Haenszel Chi-Square | 1 | 144.2628 | <.0001 |
| Phi Coefficient | | 0.2316 | |
| Contingency Coefficient | | 0.2256 | |
| Cramer's V | | 0.2316 | |

| Fisher's Exact Test | |
|---|---|
| Cell (1,1) Frequency (F) | 77 |
| Left-sided Pr <= F | 1.0000 |
| Right-sided Pr >= F | <.0001 |
| | |
| Table Probability (P) | <.0001 |
| Two-sided Pr <= P | <.0001 |

**Sample Size = 2690**
**Frequency Missing = 1**

| Frequency<br>Percent<br>Row Pct<br>Col Pct |
|---|

| Table of Heart_Failure by DM | | | |
|---|---|---|---|
| Heart_Failure(Heart Failure) | DM(DM) | | |
| | 1 | 2 | Total |
| 1 | 227<br>8.44<br>70.50<br>14.56 | 95<br>3.53<br>29.50<br>8.40 | 322<br>11.97 |
| 2 | 1332<br>49.52<br>56.25<br>85.44 | 1036<br>38.51<br>43.75<br>91.60 | 2368<br>88.03 |
| Total | 1559<br>57.96 | 1131<br>42.04 | 2690<br>100.00 |
| Frequency Missing = 1 | | | |

## The FREQ Procedure

### Statistics for Table of Heart_Failure by DM

| Statistic | DF | Value | Prob |
|---|---|---|---|
| Chi-Square | 1 | 23.6114 | <.0001 |
| Likelihood Ratio Chi-Square | 1 | 24.4481 | <.0001 |
| Continuity Adj. Chi-Square | 1 | 23.0303 | <.0001 |
| Mantel-Haenszel Chi-Square | 1 | 23.6026 | <.0001 |
| Phi Coefficient | | 0.0937 | |
| Contingency Coefficient | | 0.0933 | |
| Cramer's V | | 0.0937 | |

| Fisher's Exact Test | |
|---|---|
| Cell (1,1) Frequency (F) | 227 |
| Left-sided Pr <= F | 1.0000 |
| Right-sided Pr >= F | <.0001 |
| | |
| Table Probability (P) | <.0001 |
| Two-sided Pr <= P | <.0001 |

**Sample Size = 2690**
**Frequency Missing = 1**

| Frequency<br>Percent<br>Row Pct<br>Col Pct |
|---|

| Table of Heart_Failure by HTN | | | |
|---|---|---|---|
| | HTN(HTN) | | |
| Heart_Failure(Heart Failure) | 1 | 2 | Total |
| 1 | 218<br>8.10<br>67.70<br>13.85 | 104<br>3.87<br>32.30<br>9.32 | 322<br>11.97 |
| 2 | 1356<br>50.41<br>57.26<br>86.15 | 1012<br>37.62<br>42.74<br>90.68 | 2368<br>88.03 |
| Total | 1574<br>58.51 | 1116<br>41.49 | 2690<br>100.00 |
| Frequency Missing = 1 | | | |

## The FREQ Procedure

### Statistics for Table of Heart_Failure by HTN

| Statistic | DF | Value | Prob |
|---|---|---|---|
| Chi-Square | 1 | 12.7229 | 0.0004 |
| Likelihood Ratio Chi-Square | 1 | 13.0386 | 0.0003 |
| Continuity Adj. Chi-Square | 1 | 12.2965 | 0.0005 |
| Mantel-Haenszel Chi-Square | 1 | 12.7181 | 0.0004 |
| Phi Coefficient | | 0.0688 | |
| Contingency Coefficient | | 0.0686 | |
| Cramer's V | | 0.0688 | |

| Fisher's Exact Test | |
|---|---|
| Cell (1,1) Frequency (F) | 218 |
| Left-sided Pr <= F | 0.9999 |
| Right-sided Pr >= F | 0.0002 |
| | |
| Table Probability (P) | <.0001 |
| Two-sided Pr <= P | 0.0004 |

**Sample Size = 2690**
**Frequency Missing = 1**

| Frequency<br>Percent<br>Row Pct<br>Col Pct |
|---|

| Table of Heart_Failure by Hypercholestrolemia | | | |
|---|---|---|---|
| Heart_Failure(Heart Failure) | Hypercholestrolemia(Hypercholestrolemia) | | |
| | 1 | 2 | Total |
| 1 | 163<br>6.06<br>50.62<br>15.54 | 159<br>5.91<br>49.38<br>9.69 | 322<br>11.97 |
| 2 | 886<br>32.94<br>37.42<br>84.46 | 1482<br>55.09<br>62.58<br>90.31 | 2368<br>88.03 |
| Total | 1049<br>39.00 | 1641<br>61.00 | 2690<br>100.00 |
| Frequency Missing = 1 | | | |

## The FREQ Procedure

### Statistics for Table of Heart_Failure by Hypercholestrolemia

| Statistic | DF | Value | Prob |
|---|---|---|---|
| Chi-Square | 1 | 20.7788 | <.0001 |
| Likelihood Ratio Chi-Square | 1 | 20.3252 | <.0001 |
| Continuity Adj. Chi-Square | 1 | 20.2274 | <.0001 |
| Mantel-Haenszel Chi-Square | 1 | 20.7711 | <.0001 |
| Phi Coefficient | | 0.0879 | |
| Contingency Coefficient | | 0.0876 | |
| Cramer's V | | 0.0879 | |

| Fisher's Exact Test | |
|---|---|
| Cell (1,1) Frequency (F) | 163 |
| Left-sided Pr <= F | 1.0000 |
| Right-sided Pr >= F | <.0001 |
| | |
| Table Probability (P) | <.0001 |
| Two-sided Pr <= P | <.0001 |

**Sample Size = 2690**
**Frequency Missing = 1**

| Frequency<br>Percent<br>Row Pct<br>Col Pct |
|---|

| Table of Heart_Failure by Current_or_ex_smoking | | | |
|---|---|---|---|
| Heart_Failure(Heart Failure) | Current_or_ex_smoking(Current or ex-smoking) | | |
| | 1 | 2 | Total |
| 1 | 129<br>4.80<br>40.06<br>11.05 | 193<br>7.17<br>59.94<br>12.67 | 322<br>11.97 |
| 2 | 1038<br>38.59<br>43.83<br>88.95 | 1330<br>49.44<br>56.17<br>87.33 | 2368<br>88.03 |
| Total | 1167<br>43.38 | 1523<br>56.62 | 2690<br>100.00 |
| Frequency Missing = 1 | | | |

## The FREQ Procedure

### Statistics for Table of Heart_Failure by Current_or_ex_smoking

| Statistic | DF | Value | Prob |
|---|---|---|---|
| Chi-Square | 1 | 1.6423 | 0.2000 |
| Likelihood Ratio Chi-Square | 1 | 1.6519 | 0.1987 |
| Continuity Adj. Chi-Square | 1 | 1.4923 | 0.2219 |
| Mantel-Haenszel Chi-Square | 1 | 1.6417 | 0.2001 |
| Phi Coefficient | | -0.0247 | |
| Contingency Coefficient | | 0.0247 | |
| Cramer's V | | -0.0247 | |

| Fisher's Exact Test | |
|---|---|
| Cell (1,1) Frequency (F) | 129 |
| Left-sided Pr <= F | 0.1107 |
| Right-sided Pr >= F | 0.9104 |
| | |
| Table Probability (P) | 0.0211 |
| Two-sided Pr <= P | 0.2085 |

**Sample Size = 2690**
**Frequency Missing = 1**

| Frequency<br>Percent<br>Row Pct<br>Col Pct |
|---|

| Table of Heart_Failure by Chief_complaint | | | | | | |
|---|---|---|---|---|---|---|
| Heart_Failure(Heart Failure) | Chief_complaint(Chief complaint) | | | | | |
| | 1 | 2 | 3 | 4 | 5 | Total |
| 1 | 214<br>7.96<br>66.46<br>9.13 | 78<br>2.90<br>24.22<br>44.32 | 18<br>0.67<br>5.59<br>14.17 | 8<br>0.30<br>2.48<br>57.14 | 4<br>0.15<br>1.24<br>13.79 | 322<br>11.97 |
| 2 | 2130<br>79.18<br>89.95<br>90.87 | 98<br>3.64<br>4.14<br>55.68 | 109<br>4.05<br>4.60<br>85.83 | 6<br>0.22<br>0.25<br>42.86 | 25<br>0.93<br>1.06<br>86.21 | 2368<br>88.03 |
| Total | 2344<br>87.14 | 176<br>6.54 | 127<br>4.72 | 14<br>0.52 | 29<br>1.08 | 2690<br>100.00 |
| Frequency Missing = 1 | | | | | | |

## The FREQ Procedure

### Statistics for Table of Heart_Failure by Chief_complaint

| Statistic | DF | Value | Prob |
|---|---|---|---|
| Chi-Square | 4 | 220.5083 | <.0001 |
| Likelihood Ratio Chi-Square | 4 | 150.7931 | <.0001 |
| Mantel-Haenszel Chi-Square | 1 | 57.9013 | <.0001 |
| Phi Coefficient | | 0.2863 | |
| Contingency Coefficient | | 0.2753 | |
| Cramer's V | | 0.2863 | |

**Sample Size = 2690**
**Frequency Missing = 1**

| Frequency<br>Percent<br>Row Pct<br>Col Pct |
|---|

| Table of Heart_Failure by _1st_medical_contact | | | | | |
|---|---|---|---|---|---|
| Heart_Failure(Heart Failure) | _1st_medical_contact(1st medical contact) | | | | |
| | 1 | 2 | 3 | 4 | Total |
| 1 | 28<br>4.47<br>36.36<br>21.05 | 32<br>5.11<br>41.56<br>8.84 | 0<br>0.00<br>0.00<br>0.00 | 17<br>2.72<br>22.08<br>13.08 | 77<br>12.30 |
| 2 | 105<br>16.77<br>19.13<br>78.95 | 330<br>52.72<br>60.11<br>91.16 | 1<br>0.16<br>0.18<br>100.00 | 113<br>18.05<br>20.58<br>86.92 | 549<br>87.70 |
| Total | 133<br>21.25 | 362<br>57.83 | 1<br>0.16 | 130<br>20.77 | 626<br>100.00 |
| Frequency Missing = 2065 | | | | | |

## The FREQ Procedure

### Statistics for Table of Heart_Failure by _1st_medical_contact

| Statistic | DF | Value | Prob |
|---|---|---|---|
| Chi-Square | 3 | 13.6762 | 0.0034 |
| Likelihood Ratio Chi-Square | 3 | 12.7486 | 0.0052 |
| Mantel-Haenszel Chi-Square | 1 | 1.3998 | 0.2368 |
| Phi Coefficient | | 0.1478 | |
| Contingency Coefficient | | 0.1462 | |
| Cramer's V | | 0.1478 | |
| WARNING: 25% of the cells have expected counts less than 5. Chi-Square may not be a valid test. | | | |

**Sample Size = 626**
**Frequency Missing = 2065**

**WARNING: 77% of the data are missing.**

| Frequency<br>Percent<br>Row Pct<br>Col Pct |
|---|

| Table of Heart_Failure by Transferred_by_EMS_e_g__Red_Cres | | | |
|---|---|---|---|
| Heart_Failure(Heart Failure) | Transferred_by_EMS_e_g__Red_Cres(Transferred by EMS e.g. Red Crescent or Red Cross?) | | |
| | 1 | 2 | Total |
| 1 | 52<br>8.84<br>57.78<br>22.71 | 38<br>6.46<br>42.22<br>10.58 | 90<br>15.31 |
| 2 | 177<br>30.10<br>35.54<br>77.29 | 321<br>54.59<br>64.46<br>89.42 | 498<br>84.69 |
| Total | 229<br>38.95 | 359<br>61.05 | 588<br>100.00 |
| Frequency Missing = 2103 | | | |

## The FREQ Procedure

### Statistics for Table of Heart_Failure by Transferred_by_EMS_e_g__Red_Cres

| Statistic | DF | Value | Prob |
|---|---|---|---|
| Chi-Square | 1 | 15.8496 | <.0001 |
| Likelihood Ratio Chi-Square | 1 | 15.4454 | <.0001 |
| Continuity Adj. Chi-Square | 1 | 14.9282 | 0.0001 |
| Mantel-Haenszel Chi-Square | 1 | 15.8226 | <.0001 |
| Phi Coefficient | | 0.1642 | |
| Contingency Coefficient | | 0.1620 | |
| Cramer's V | | 0.1642 | |

| Fisher's Exact Test | |
|---|---|
| Cell (1,1) Frequency (F) | 52 |
| Left-sided Pr <= F | 1.0000 |
| Right-sided Pr >= F | <.0001 |
| | |
| Table Probability (P) | <.0001 |
| Two-sided Pr <= P | <.0001 |

**Sample Size = 588**
**Frequency Missing = 2103**

**WARNING: 78% of the data are missing.**

| Frequency<br>Percent<br>Row Pct<br>Col Pct |
|---|

| Table of Heart_Failure by Cardiac_arrest | | | |
|---|---|---|---|
| | Cardiac_arrest(Cardiac arrest) | | |
| Heart_Failure(Heart Failure) | 1 | 2 | Total |
| 1 | 38<br>1.41<br>11.80<br>51.35 | 284<br>10.56<br>88.20<br>10.86 | 322<br>11.97 |
| 2 | 36<br>1.34<br>1.52<br>48.65 | 2332<br>86.69<br>98.48<br>89.14 | 2368<br>88.03 |
| Total | 74<br>2.75 | 2616<br>97.25 | 2690<br>100.00 |
| Frequency Missing = 1 | | | |

## The FREQ Procedure

### Statistics for Table of Heart_Failure by Cardiac_arrest

| Statistic | DF | Value | Prob |
|---|---|---|---|
| Chi-Square | 1 | 111.9924 | <.0001 |
| Likelihood Ratio Chi-Square | 1 | 71.1441 | <.0001 |
| Continuity Adj. Chi-Square | 1 | 108.1824 | <.0001 |
| Mantel-Haenszel Chi-Square | 1 | 111.9508 | <.0001 |
| Phi Coefficient | | 0.2040 | |
| Contingency Coefficient | | 0.1999 | |
| Cramer's V | | 0.2040 | |

| Fisher's Exact Test | |
|---|---|
| Cell (1,1) Frequency (F) | 38 |
| Left-sided Pr <= F | 1.0000 |
| Right-sided Pr >= F | <.0001 |
| | |
| Table Probability (P) | <.0001 |
| Two-sided Pr <= P | <.0001 |

**Sample Size = 2690**
**Frequency Missing = 1**

| Frequency<br>Percent<br>Row Pct<br>Col Pct |
|---|

| Table of Heart_Failure by CHF_Killip_Class | | | | | |
|---|---|---|---|---|---|
| Heart_Failure(Heart Failure) | CHF_Killip_Class(CHF Killip Class) | | | | |
| | 1 | 2 | 3 | 4 | Total |
| 1 | 109<br>4.05<br>33.85<br>4.62 | 126<br>4.68<br>39.13<br>64.62 | 66<br>2.45<br>20.50<br>68.75 | 21<br>0.78<br>6.52<br>51.22 | 322<br>11.97 |
| 2 | 2249<br>83.61<br>94.97<br>95.38 | 69<br>2.57<br>2.91<br>35.38 | 30<br>1.12<br>1.27<br>31.25 | 20<br>0.74<br>0.84<br>48.78 | 2368<br>88.03 |
| Total | 2358<br>87.66 | 195<br>7.25 | 96<br>3.57 | 41<br>1.52 | 2690<br>100.00 |
| Frequency Missing = 1 | | | | | |

## The FREQ Procedure

### Statistics for Table of Heart_Failure by CHF_Killip_Class

| Statistic | DF | Value | Prob |
|---|---|---|---|
| Chi-Square | 3 | 987.3493 | <.0001 |
| Likelihood Ratio Chi-Square | 3 | 658.3218 | <.0001 |
| Mantel-Haenszel Chi-Square | 1 | 753.0152 | <.0001 |
| Phi Coefficient | | 0.6058 | |
| Contingency Coefficient | | 0.5182 | |
| Cramer's V | | 0.6058 | |

**Sample Size = 2690**
**Frequency Missing = 1**

| Frequency<br>Percent<br>Row Pct<br>Col Pct |
|---|

| Table of Heart_Failure by Echo_Options | | | | | |
|---|---|---|---|---|---|
| Heart_Failure(Heart Failure) | Echo_Options(Echo-Options) | | | | |
| | 1 | 2 | 3 | 4 | Total |
| 1 | 20<br>0.84<br>6.62<br>2.11 | 86<br>3.61<br>28.48<br>11.18 | 102<br>4.28<br>33.77<br>22.17 | 94<br>3.94<br>31.13<br>44.98 | 302<br>12.66 |
| 2 | 927<br>38.87<br>44.50<br>97.89 | 683<br>28.64<br>32.79<br>88.82 | 358<br>15.01<br>17.19<br>77.83 | 115<br>4.82<br>5.52<br>55.02 | 2083<br>87.34 |
| Total | 947<br>39.71 | 769<br>32.24 | 460<br>19.29 | 209<br>8.76 | 2385<br>100.00 |
| Frequency Missing = 306 | | | | | |

## The FREQ Procedure

### Statistics for Table of Heart_Failure by Echo_Options

| Statistic | DF | Value | Prob |
|---|---|---|---|
| Chi-Square | 3 | 331.8022 | <.0001 |
| Likelihood Ratio Chi-Square | 3 | 305.1354 | <.0001 |
| Mantel-Haenszel Chi-Square | 1 | 313.3613 | <.0001 |
| Phi Coefficient | | 0.3730 | |
| Contingency Coefficient | | 0.3495 | |
| Cramer's V | | 0.3730 | |

**Sample Size = 2385**
**Frequency Missing = 306**

**WARNING: 11% of the data are missing.**

| Frequency<br>Percent<br>Row Pct<br>Col Pct |
|---|

Table of Heart_Failure by Elective_coronary_angiogram

| Heart_Failure(Heart Failure) | Elective_coronary_angiogram(Elective coronary angiogram) 1 | 2 | Total |
|---|---|---|---|
| 1 | 26<br>1.49<br>13.27<br>6.25 | 170<br>9.77<br>86.73<br>12.84 | 196<br>11.26 |
| 2 | 390<br>22.41<br>25.26<br>93.75 | 1154<br>66.32<br>74.74<br>87.16 | 1544<br>88.74 |
| Total | 416<br>23.91 | 1324<br>76.09 | 1740<br>100.00 |
| Frequency Missing = 951 | | | |

## The FREQ Procedure

### Statistics for Table of Heart_Failure by Elective_coronary_angiogram

| Statistic | DF | Value | Prob |
|---|---|---|---|
| Chi-Square | 1 | 13.7525 | 0.0002 |
| Likelihood Ratio Chi-Square | 1 | 15.4084 | <.0001 |
| Continuity Adj. Chi-Square | 1 | 13.1011 | 0.0003 |
| Mantel-Haenszel Chi-Square | 1 | 13.7446 | 0.0002 |
| Phi Coefficient | | -0.0889 | |
| Contingency Coefficient | | 0.0886 | |
| Cramer's V | | -0.0889 | |

| Fisher's Exact Test | |
|---|---|
| Cell (1,1) Frequency (F) | 26 |
| Left-sided Pr <= F | <.0001 |
| Right-sided Pr >= F | 1.0000 |
| | |
| Table Probability (P) | <.0001 |
| Two-sided Pr <= P | 0.0001 |

**Sample Size = 1740**
**Frequency Missing = 951**

**WARNING: 35% of the data are missing.**

| Frequency<br>Percent<br>Row Pct<br>Col Pct |
|---|

| Table of Heart_Failure by Arterial_access | | | | |
|---|---|---|---|---|
| Heart_Failure(Heart Failure) | Arterial_access(Arterial access) | | | |
| | 1 | 2 | 3 | Total |
| 1 | 41<br>3.51<br>28.87<br>14.54 | 101<br>8.64<br>71.13<br>11.40 | 0<br>0.00<br>0.00<br>0.00 | 142<br>12.15 |
| 2 | 241<br>20.62<br>23.47<br>85.46 | 785<br>67.15<br>76.44<br>88.60 | 1<br>0.09<br>0.10<br>100.00 | 1027<br>87.85 |
| Total | 282<br>24.12 | 886<br>75.79 | 1<br>0.09 | 1169<br>100.00 |
| Frequency Missing = 1522 | | | | |

## The FREQ Procedure

### Statistics for Table of Heart_Failure by Arterial_access

| Statistic | DF | Value | Prob |
|---|---|---|---|
| Chi-Square | 2 | 2.1141 | 0.3475 |
| Likelihood Ratio Chi-Square | 2 | 2.1655 | 0.3387 |
| Mantel-Haenszel Chi-Square | 1 | 2.0489 | 0.1523 |
| Phi Coefficient | | 0.0425 | |
| Contingency Coefficient | | 0.0425 | |
| Cramer's V | | 0.0425 | |
| WARNING: 33% of the cells have expected counts less than 5. Chi-Square may not be a valid test. | | | |

**Sample Size = 1169**
**Frequency Missing = 1522**

**WARNING: 57% of the data are missing.**

| Frequency<br>Percent<br>Row Pct<br>Col Pct |
|---|

| Table of Heart_Failure by Arterial_access_1 | | | |
|---|---|---|---|
| Heart_Failure(Heart Failure) | Arterial_access_1(Arterial access_1) | | |
| | 1 | 2 | Total |
| 1 | 7<br>1.68<br>26.92<br>7.22 | 19<br>4.57<br>73.08<br>5.96 | 26<br>6.25 |
| 2 | 90<br>21.63<br>23.08<br>92.78 | 300<br>72.12<br>76.92<br>94.04 | 390<br>93.75 |
| Total | 97<br>23.32 | 319<br>76.68 | 416<br>100.00 |
| Frequency Missing = 2275 | | | |

## The FREQ Procedure

### Statistics for Table of Heart_Failure by Arterial_access_1

| Statistic | DF | Value | Prob |
|---|---|---|---|
| Chi-Square | 1 | 0.2017 | 0.6534 |
| Likelihood Ratio Chi-Square | 1 | 0.1955 | 0.6584 |
| Continuity Adj. Chi-Square | 1 | 0.0439 | 0.8340 |
| Mantel-Haenszel Chi-Square | 1 | 0.2012 | 0.6538 |
| Phi Coefficient | | 0.0220 | |
| Contingency Coefficient | | 0.0220 | |
| Cramer's V | | 0.0220 | |

| Fisher's Exact Test | |
|---|---|
| Cell (1,1) Frequency (F) | 7 |
| Left-sided Pr <= F | 0.7605 |
| Right-sided Pr >= F | 0.4032 |
| | |
| Table Probability (P) | 0.1636 |
| Two-sided Pr <= P | 0.6358 |

**Sample Size = 416**
**Frequency Missing = 2275**

**WARNING: 85% of the data are missing.**

## The TTEST Procedure

### Variable: Age (Age)

| Heart_Failure | Method | N | Mean | Std Dev | Std Err | Minimum | Maximum |
|---|---|---|---|---|---|---|---|
| 1 | | 322 | 61.5373 | 12.6577 | 0.7054 | 28.0000 | 91.0000 |
| 2 | | 2368 | 56.5676 | 12.2830 | 0.2524 | 19.0000 | 103.0 |
| Diff (1-2) | Pooled | | 4.9697 | 12.3283 | 0.7323 | | |
| Diff (1-2) | Satterthwaite | | 4.9697 | | 0.7492 | | |

| Heart_Failure | Method | Mean | 95% CL Mean | | Std Dev | 95% CL Std Dev | |
|---|---|---|---|---|---|---|---|
| 1 | | 61.5373 | 60.1495 | 62.9250 | 12.6577 | 11.7498 | 13.7189 |
| 2 | | 56.5676 | 56.0726 | 57.0625 | 12.2830 | 11.9429 | 12.6432 |
| Diff (1-2) | Pooled | 4.9697 | 3.5339 | 6.4055 | 12.3283 | 12.0074 | 12.6670 |
| Diff (1-2) | Satterthwaite | 4.9697 | 3.4969 | 6.4425 | | | |

| Method | Variances | DF | t Value | Pr > \|t\| |
|---|---|---|---|---|
| Pooled | Equal | 2688 | 6.79 | <.0001 |
| Satterthwaite | Unequal | 407.56 | 6.63 | <.0001 |

| Equality of Variances | | | | |
|---|---|---|---|---|
| Method | Num DF | Den DF | F Value | Pr > F |
| Folded F | 321 | 2367 | 1.06 | 0.4586 |

**The TTEST Procedure**

**Variable: Age (Age)**

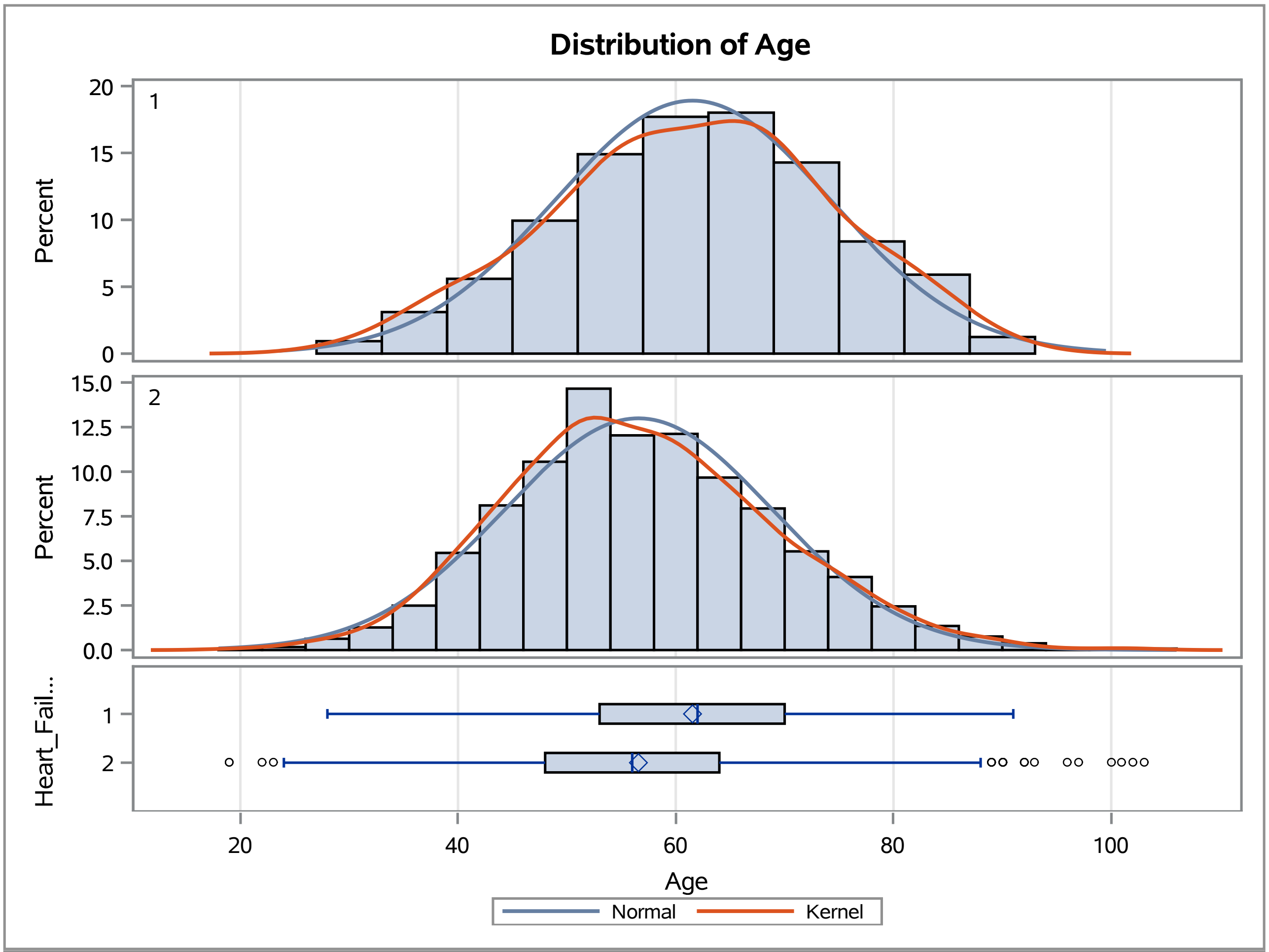


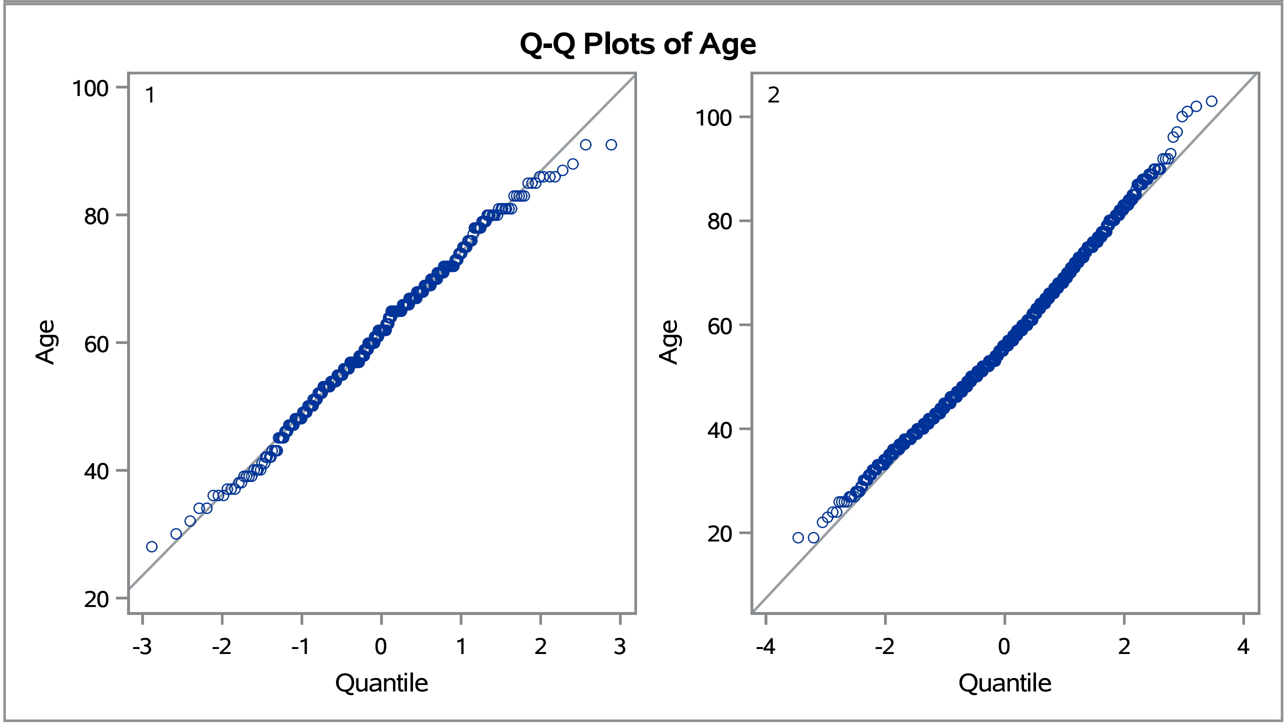

## The TTEST Procedure

### Variable: BMI (BMI)

Variable: BMI (BMI)

| Heart_Failure | Method | N | Mean | Std Dev | Std Err | Minimum | Maximum |
|---|---|---|---|---|---|---|---|
| 1 | | 322 | 28.9041 | 4.9590 | 0.2764 | 19.4800 | 50.0000 |
| 2 | | 2368 | 28.4812 | 4.7473 | 0.0976 | 15.1600 | 75.0000 |
| Diff (1-2) | Pooled | | 0.4229 | 4.7731 | 0.2835 | | |
| Diff (1-2) | Satterthwaite | | 0.4229 | | 0.2931 | | |

| Heart_Failure | Method | Mean | 95% CL Mean | | Std Dev | 95% CL Std Dev | |
|---|---|---|---|---|---|---|---|
| 1 | | 28.9041 | 28.3604 | 29.4478 | 4.9590 | 4.6033 | 5.3747 |
| 2 | | 28.4812 | 28.2899 | 28.6726 | 4.7473 | 4.6159 | 4.8865 |
| Diff (1-2) | Pooled | 0.4229 | -0.1330 | 0.9788 | 4.7731 | 4.6488 | 4.9042 |
| Diff (1-2) | Satterthwaite | 0.4229 | -0.1533 | 0.9990 | | | |

| Method | Variances | DF | t Value | Pr > \|t\| |
|---|---|---|---|---|
| Pooled | Equal | 2688 | 1.49 | 0.1359 |
| Satterthwaite | Unequal | 405.14 | 1.44 | 0.1498 |

| Equality of Variances | | | | |
|---|---|---|---|---|
| Method | Num DF | Den DF | F Value | Pr > F |
| Folded F | 321 | 2367 | 1.09 | 0.2847 |

## The TTEST Procedure

### Variable: BMI (BMI)

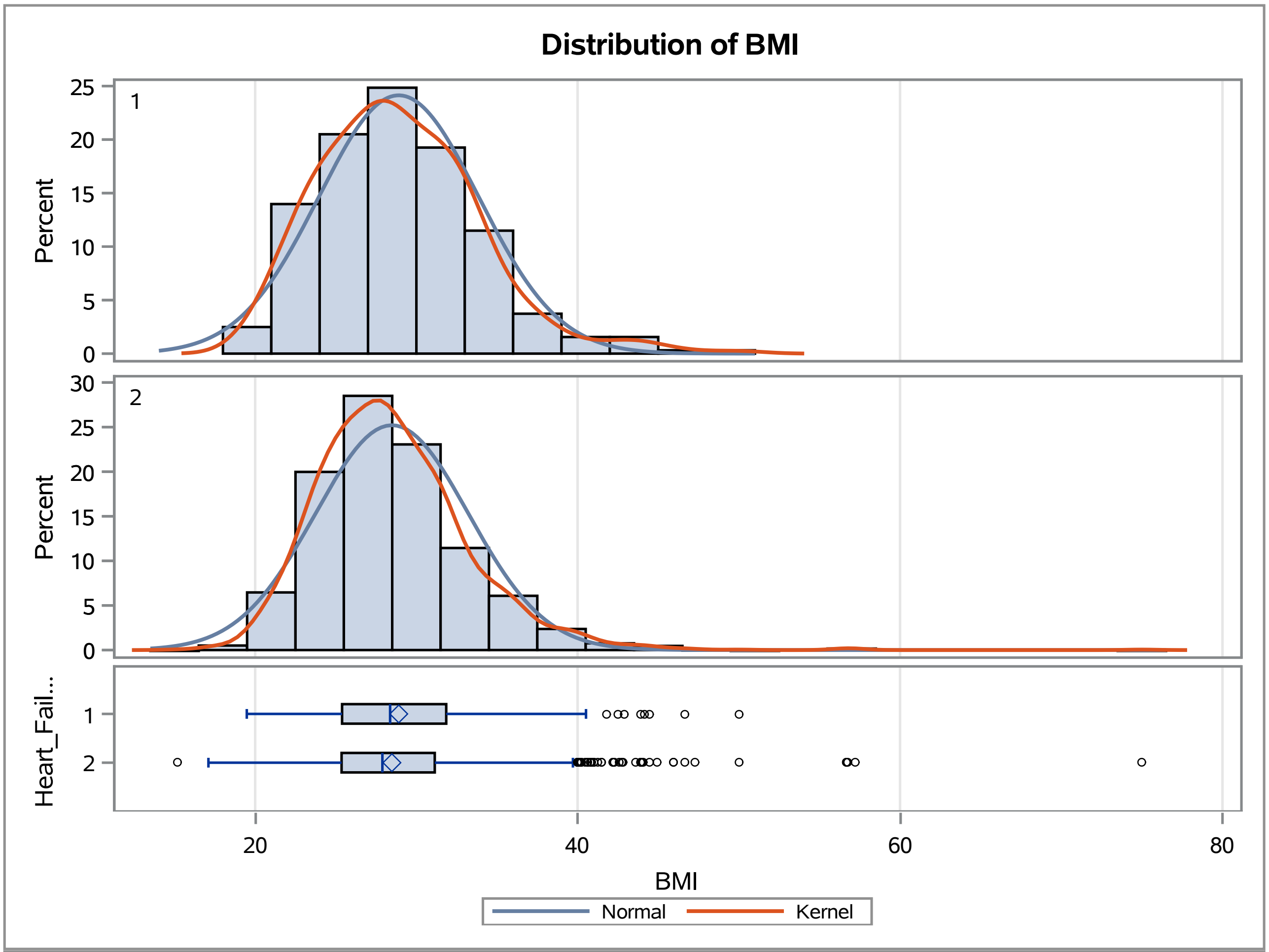


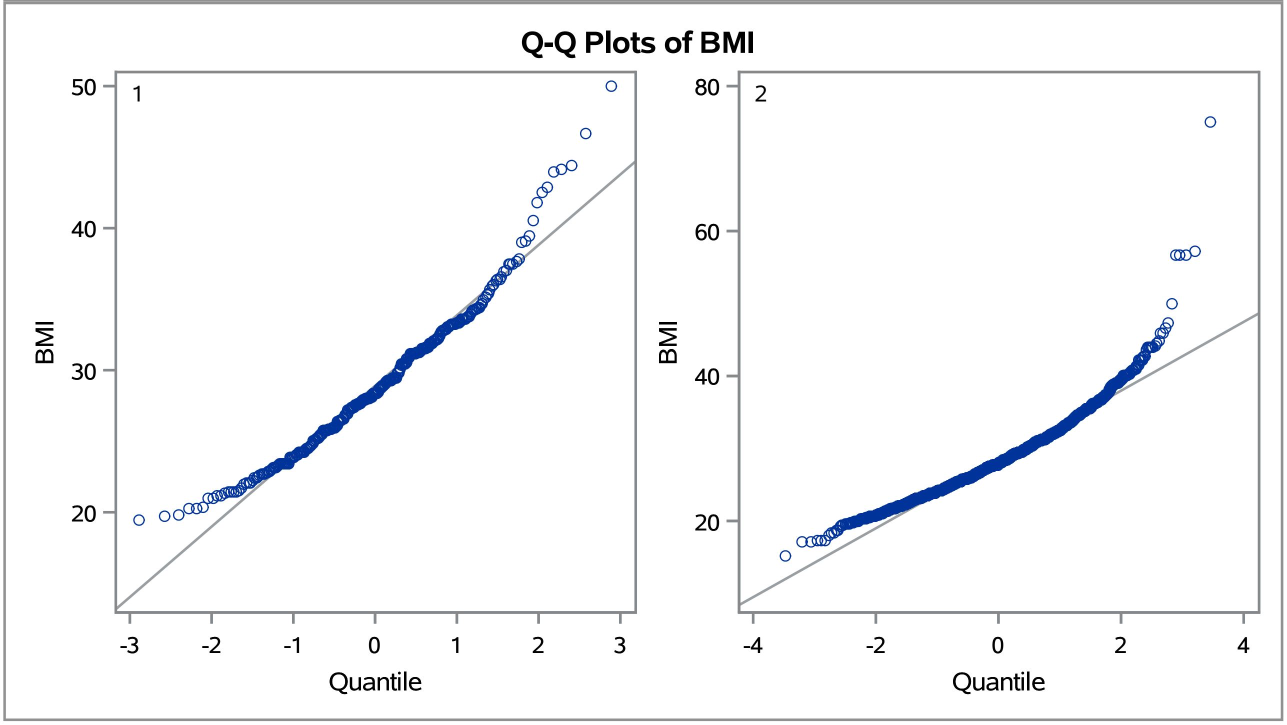

## The TTEST Procedure

### Variable: HR_bpm_ (HR(bpm))

Variable: HR_bpm_ (HR(bpm))

| Heart_Failure | Method | N | Mean | Std Dev | Std Err | Minimum | Maximum |
|---|---|---|---|---|---|---|---|
| 1 | | 321 | 93.3364 | 20.6323 | 1.1516 | 10.0000 | 190.0 |
| 2 | | 2368 | 82.9206 | 16.9511 | 0.3483 | 10.0000 | 230.0 |
| Diff (1-2) | Pooled | | 10.4158 | 17.4304 | 1.0367 | | |
| Diff (1-2) | Satterthwaite | | 10.4158 | | 1.2031 | | |

| Heart_Failure | Method | Mean | 95% CL Mean | | Std Dev | 95% CL Std Dev | |
|---|---|---|---|---|---|---|---|
| 1 | | 93.3364 | 91.0708 | 95.6021 | 20.6323 | 19.1502 | 22.3650 |
| 2 | | 82.9206 | 82.2375 | 83.6037 | 16.9511 | 16.4818 | 17.4482 |
| Diff (1-2) | Pooled | 10.4158 | 8.3830 | 12.4487 | 17.4304 | 16.9766 | 17.9093 |
| Diff (1-2) | Satterthwaite | 10.4158 | 8.0503 | 12.7814 | | | |

| Method | Variances | DF | t Value | Pr > \|t\| |
|---|---|---|---|---|
| Pooled | Equal | 2687 | 10.05 | <.0001 |
| Satterthwaite | Unequal | 380.81 | 8.66 | <.0001 |

| Equality of Variances | | | | |
|---|---|---|---|---|
| Method | Num DF | Den DF | F Value | Pr > F |
| Folded F | 320 | 2367 | 1.48 | <.0001 |

## The TTEST Procedure

### Variable: HR_bpm_ (HR(bpm))

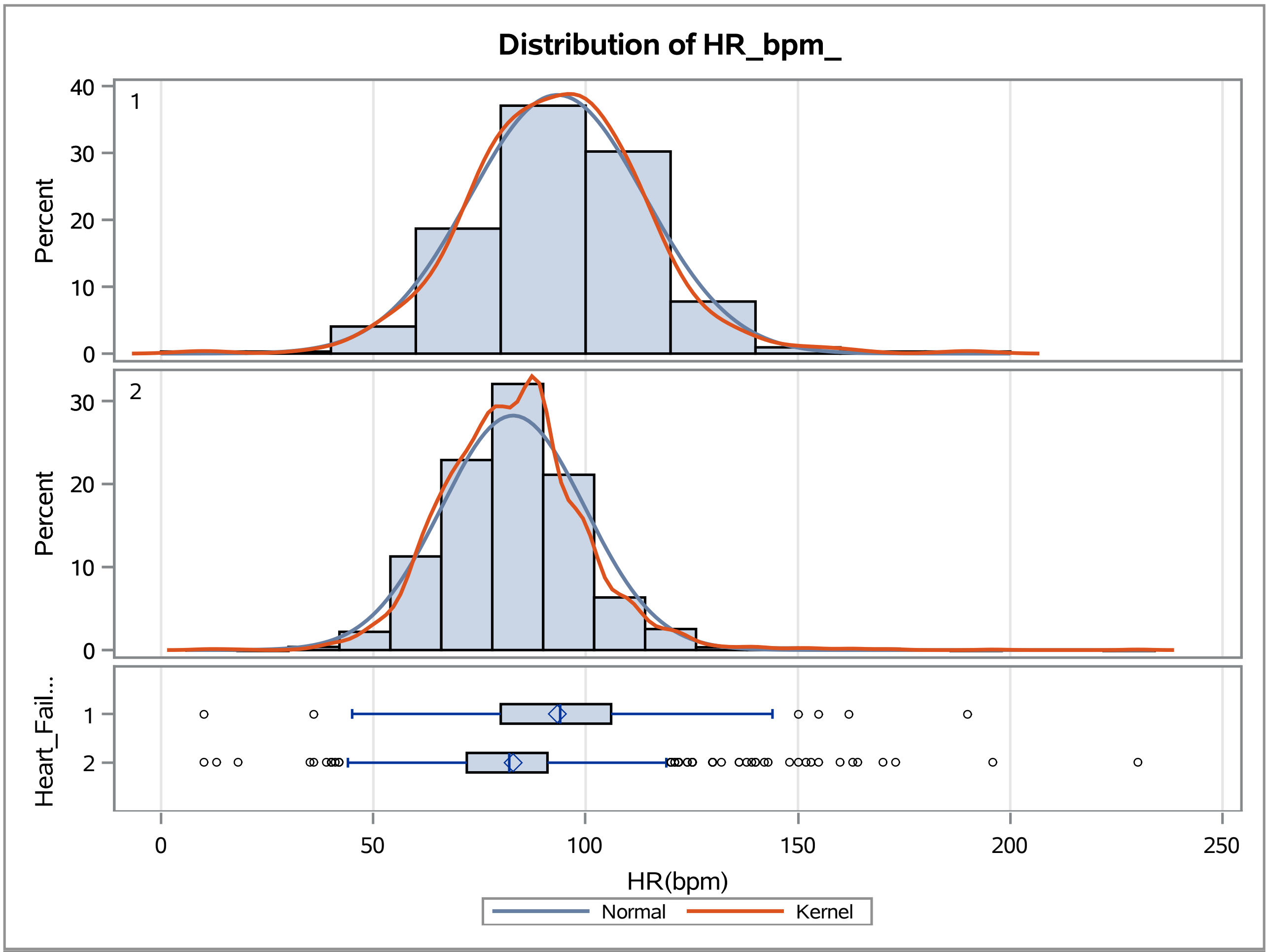


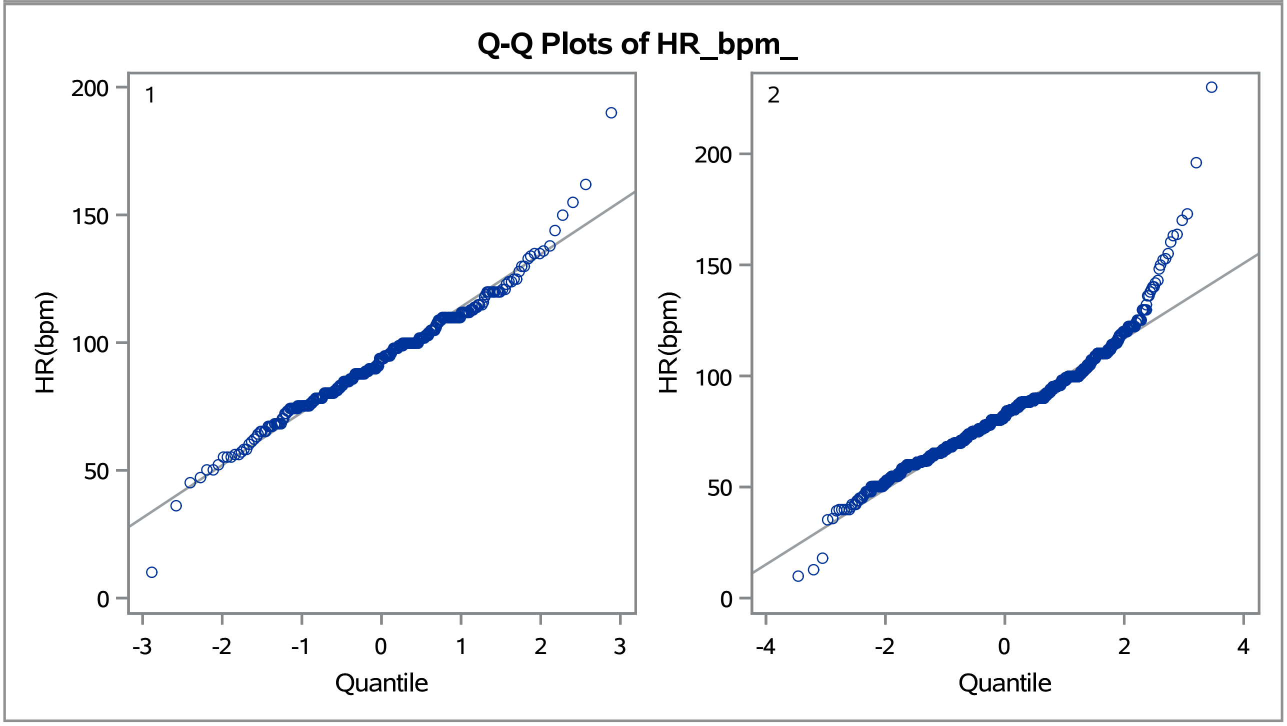

## The TTEST Procedure

### Variable: SBP_mmgH_ (SBP(mmgH))

### Variable: SBP_mmgH_ (SBP(mmgH))

| Heart_Failure | Method | N | Mean | Std Dev | Std Err | Minimum | Maximum |
|---|---|---|---|---|---|---|---|
| 1 | | 321 | 129.8 | 30.2503 | 1.6884 | 40.0000 | 220.0 |
| 2 | | 2368 | 135.6 | 24.6626 | 0.5068 | 55.0000 | 244.0 |
| Diff (1-2) | Pooled | | -5.7172 | 25.3926 | 1.5103 | | |
| Diff (1-2) | Satterthwaite | | -5.7172 | | 1.7628 | | |

| Heart_Failure | Method | Mean | 95% CL Mean | | Std Dev | 95% CL Std Dev | |
|---|---|---|---|---|---|---|---|
| 1 | | 129.8 | 126.5 | 133.2 | 30.2503 | 28.0772 | 32.7907 |
| 2 | | 135.6 | 134.6 | 136.6 | 24.6626 | 23.9797 | 25.3858 |
| Diff (1-2) | Pooled | -5.7172 | -8.6786 | -2.7557 | 25.3926 | 24.7315 | 26.0902 |
| Diff (1-2) | Satterthwaite | -5.7172 | -9.1833 | -2.2510 | | | |

| Method | Variances | DF | t Value | Pr > \|t\| |
|---|---|---|---|---|
| Pooled | Equal | 2687 | -3.79 | 0.0002 |
| Satterthwaite | Unequal | 379.85 | -3.24 | 0.0013 |

| Equality of Variances | | | | |
|---|---|---|---|---|
| Method | Num DF | Den DF | F Value | Pr > F |
| Folded F | 320 | 2367 | 1.50 | <.0001 |

## The TTEST Procedure

### Variable: SBP_mmgH_ (SBP(mmgH))

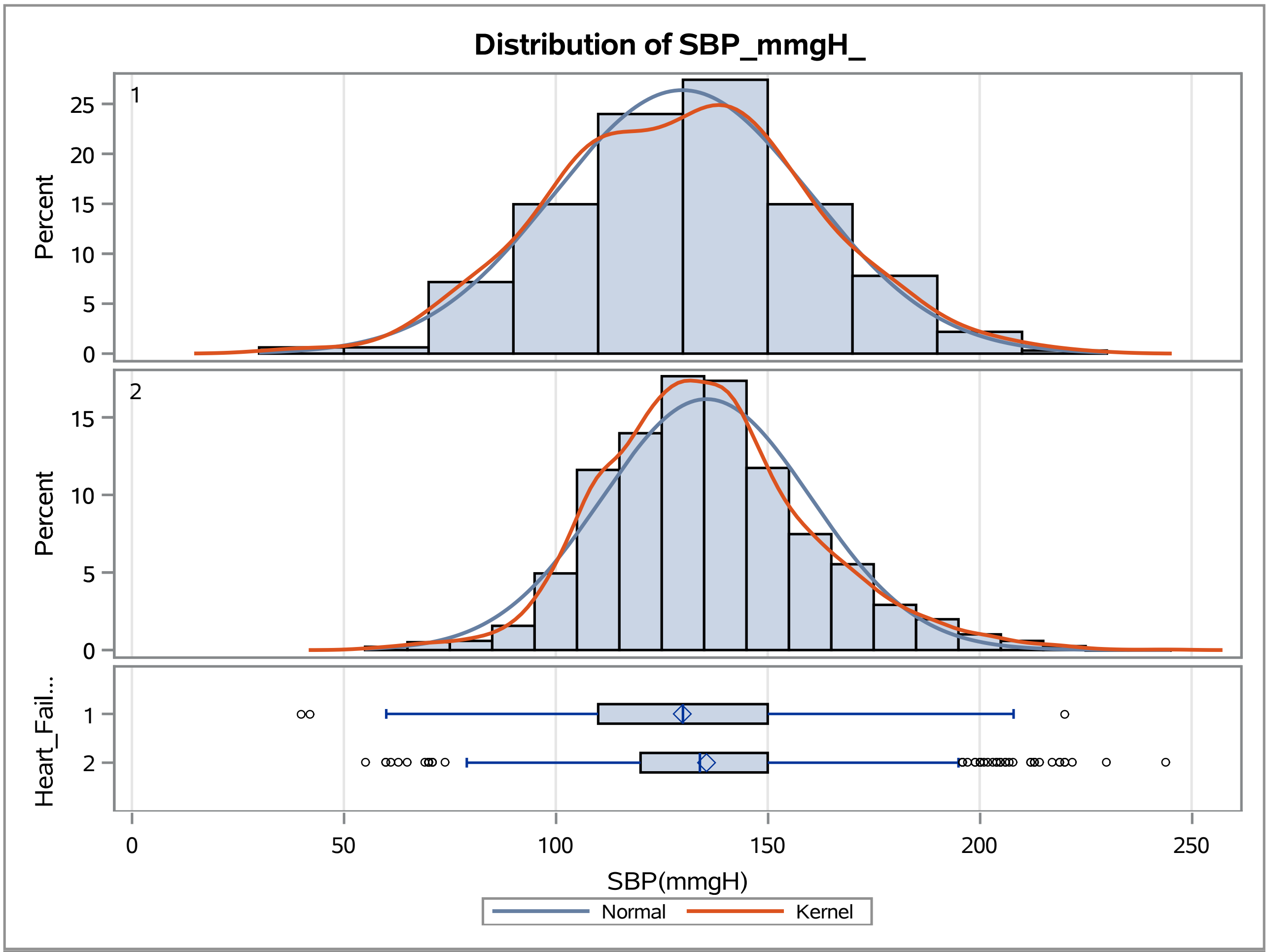


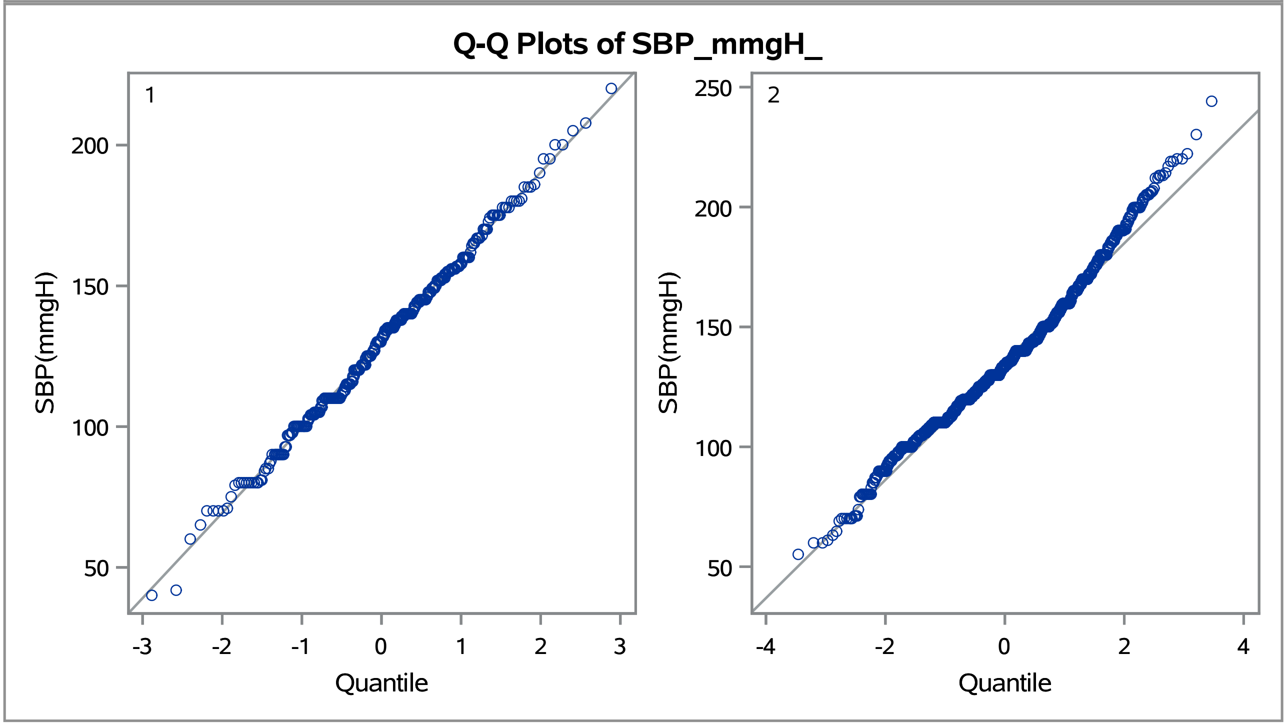

## The LOGISTIC Procedure

| Model Information | | |
|---|---|---|
| **Data Set** | WAEL.STARS4 | |
| **Response Variable** | Heart_Failure | Heart Failure |
| **Number of Response Levels** | 2 | |
| **Model** | binary logit | |
| **Optimization Technique** | Fisher's scoring | |

| | |
|---|---|
| **Number of Observations Read** | 2691 |
| **Number of Observations Used** | 2690 |

| Response Profile | | |
|---|---|---|
| **Ordered Value** | **Heart_Failure** | **Total Frequency** |
| **1** | 1 | 322 |
| **2** | 2 | 2368 |

**Probability modeled is Heart_Failure='1'.**

**Note:** 1 observation was deleted due to missing values for the response or explanatory variables.

| Class Level Information | | |
|---|---|---|
| **Class** | **Value** | **Design Variables** |
| **Gender** | **1** | 0 |
| | **2** | 1 |

| Model Convergence Status |
|---|
| Convergence criterion (GCONV=1E-8) satisfied. |

| Model Fit Statistics | | |
|---|---|---|
| **Criterion** | **Intercept Only** | **Intercept and Covariates** |
| **AIC** | 1972.866 | 1968.747 |
| **SC** | 1978.764 | 1980.542 |
| **-2 Log L** | 1970.866 | 1964.747 |

| Testing Global Null Hypothesis: BETA=0 | | | |
|---|---|---|---|
| **Test** | **Chi-Square** | **DF** | **Pr > ChiSq** |
| **Likelihood Ratio** | 6.1189 | 1 | 0.0134 |
| **Score** | 6.4802 | 1 | 0.0109 |
| **Wald** | 6.4398 | 1 | 0.0112 |

## The LOGISTIC Procedure

| Type 3 Analysis of Effects | | | |
|---|---|---|---|
| Effect | DF | Wald Chi-Square | Pr > ChiSq |
| Gender | 1 | 6.4398 | 0.0112 |

| Analysis of Maximum Likelihood Estimates | | | | | | |
|---|---|---|---|---|---|---|
| Parameter | | DF | Estimate | Standard Error | Wald Chi-Square | Pr > ChiSq |
| Intercept | | 1 | -2.0678 | 0.0674 | 941.3314 | <.0001 |
| Gender | 2 | 1 | 0.3634 | 0.1432 | 6.4398 | 0.0112 |

| Odds Ratio Estimates | | | |
|---|---|---|---|
| Effect | Point Estimate | 95% Wald Confidence Limits | |
| Gender 2 vs 1 | 1.438 | 1.086 | 1.904 |

| Association of Predicted Probabilities and Observed Responses | | | |
|---|---|---|---|
| Percent Concordant | 19.0 | Somers' D | 0.058 |
| Percent Discordant | 13.2 | Gamma | 0.180 |
| Percent Tied | 67.7 | Tau-a | 0.012 |
| Pairs | 762496 | c | 0.529 |

## The LOGISTIC Procedure

| Model Information | | |
|---|---|---|
| **Data Set** | WAEL.STARS4 | |
| **Response Variable** | Heart_Failure | Heart Failure |
| **Number of Response Levels** | 2 | |
| **Model** | binary logit | |
| **Optimization Technique** | Fisher's scoring | |

| | |
|---|---|
| **Number of Observations Read** | 2691 |
| **Number of Observations Used** | 1104 |

| Response Profile | | |
|---|---|---|
| **Ordered Value** | **Heart_Failure** | **Total Frequency** |
| **1** | 1 | 123 |
| **2** | 2 | 981 |

**Probability modeled is Heart_Failure='1'.**

**Note:** 1587 observations were deleted due to missing values for the response or explanatory variables.

| Class Level Information | | | | |
|---|---|---|---|---|
| **Class** | **Value** | **Design Variables** | | |
| **Gender** | **1** | 0 | | |
| | **2** | 1 | | |
| **Hypercholestrolemia** | **1** | 0 | | |
| | **2** | 1 | | |
| **CAD** | **1** | 1 | | |
| | **2** | 0 | | |
| **History_of_heart_failure** | **1** | 0 | | |
| | **2** | 1 | | |
| **History_of_stroke** | **1** | 0 | | |
| | **2** | 1 | | |
| **History_of_chronic_renal_failure** | **1** | 0 | | |
| | **2** | 1 | | |
| **DM** | **1** | 0 | | |
| | **2** | 1 | | |
| **HTN** | **1** | 0 | | |
| | **2** | 1 | | |
| **CHF_Killip_Class** | **1** | 0 | 0 | 0 |

## The LOGISTIC Procedure

| Class Level Information | | | | |
|---|---|---|---|---|
| Class | Value | Design Variables | | |
| | 2 | 1 | 0 | 0 |
| | 3 | 0 | 1 | 0 |
| | 4 | 0 | 0 | 1 |
| Cardiac_arrest | 1 | 0 | | |
| | 2 | 1 | | |
| Type_of_STEMI | 1 | 0 | 0 | |
| | 2 | 1 | 0 | |
| | 3 | 0 | 1 | |
| Echo_Options | 1 | 0 | 0 | 0 |
| | 2 | 1 | 0 | 0 |
| | 3 | 0 | 1 | 0 |
| | 4 | 0 | 0 | 1 |

| Model Convergence Status |
|---|
| Convergence criterion (GCONV=1E-8) satisfied. |

| Model Fit Statistics | | |
|---|---|---|
| Criterion | Intercept Only | Intercept and Covariates |
| AIC | 773.607 | 478.130 |
| SC | 778.613 | 583.270 |
| -2 Log L | 771.607 | 436.130 |

| Testing Global Null Hypothesis: BETA=0 | | | |
|---|---|---|---|
| Test | Chi-Square | DF | Pr > ChiSq |
| Likelihood Ratio | 335.4770 | 20 | <.0001 |
| Score | 459.7944 | 20 | <.0001 |
| Wald | 186.0616 | 20 | <.0001 |

## The LOGISTIC Procedure

| Type 3 Analysis of Effects | | | |
|---|---|---|---|
| Effect | DF | Wald Chi-Square | Pr > ChiSq |
| Gender | 1 | 0.2294 | 0.6320 |
| History_of_heart_fai | 1 | 0.1708 | 0.6794 |
| History_of_chronic_r | 1 | 8.3751 | 0.0038 |
| DM | 1 | 0.7460 | 0.3877 |
| HTN | 1 | 4.4523 | 0.0349 |
| CHF_Killip_Class | 3 | 112.7628 | <.0001 |
| Age | 1 | 1.3080 | 0.2528 |
| SBP_mmgH_ | 1 | 0.5533 | 0.4570 |
| HR_bpm_ | 1 | 10.1307 | 0.0015 |
| CAD | 1 | 0.3357 | 0.5623 |
| Hypercholestrolemia | 1 | 2.5671 | 0.1091 |
| Cardiac_arrest | 1 | 3.7979 | 0.0513 |
| History_of_stroke | 1 | 0.0421 | 0.8373 |
| Type_of_STEMI | 2 | 1.5418 | 0.4626 |
| Echo_Options | 3 | 38.3649 | <.0001 |

| Analysis of Maximum Likelihood Estimates | | | | | | |
|---|---|---|---|---|---|---|
| Parameter | | DF | Estimate | Standard Error | Wald Chi-Square | Pr > ChiSq |
| Intercept | | 1 | -4.1937 | 1.7152 | 5.9779 | 0.0145 |
| Gender | 2 | 1 | 0.1924 | 0.4017 | 0.2294 | 0.6320 |
| History_of_heart_fai | 2 | 1 | -0.2587 | 0.6260 | 0.1708 | 0.6794 |
| History_of_chronic_r | 2 | 1 | -1.8195 | 0.6287 | 8.3751 | 0.0038 |
| DM | 2 | 1 | -0.2441 | 0.2826 | 0.7460 | 0.3877 |
| HTN | 2 | 1 | 0.6437 | 0.3051 | 4.4523 | 0.0349 |
| CHF_Killip_Class | 2 | 1 | 2.8412 | 0.3254 | 76.2556 | <.0001 |
| CHF_Killip_Class | 3 | 1 | 3.9239 | 0.5272 | 55.4037 | <.0001 |
| CHF_Killip_Class | 4 | 1 | 1.2109 | 0.5587 | 4.6973 | 0.0302 |
| Age | | 1 | 0.0133 | 0.0116 | 1.3080 | 0.2528 |
| SBP_mmgH_ | | 1 | -0.00355 | 0.00477 | 0.5533 | 0.4570 |
| HR_bpm_ | | 1 | 0.0196 | 0.00617 | 10.1307 | 0.0015 |
| CAD | 1 | 1 | 0.1792 | 0.3093 | 0.3357 | 0.5623 |
| Hypercholestrolemia | 2 | 1 | -0.4677 | 0.2919 | 2.5671 | 0.1091 |
| Cardiac_arrest | 2 | 1 | -0.9374 | 0.4810 | 3.7979 | 0.0513 |
| History_of_stroke | 2 | 1 | 0.1786 | 0.8700 | 0.0421 | 0.8373 |

## The LOGISTIC Procedure

| Analysis of Maximum Likelihood Estimates | | | | | | |
|---|---|---|---|---|---|---|
| Parameter | | DF | Estimate | Standard Error | Wald Chi-Square | Pr > ChiSq |
| Type_of_STEMI | 2 | 1 | 0.1859 | 0.3233 | 0.3306 | 0.5653 |
| Type_of_STEMI | 3 | 1 | 0.6637 | 0.5541 | 1.4347 | 0.2310 |
| Echo_Options | 2 | 1 | 1.6412 | 0.5950 | 7.6077 | 0.0058 |
| Echo_Options | 3 | 1 | 2.1557 | 0.6058 | 12.6630 | 0.0004 |
| Echo_Options | 4 | 1 | 3.4480 | 0.6323 | 29.7411 | <.0001 |

| Odds Ratio Estimates | | | |
|---|---|---|---|
| Effect | Point Estimate | 95% Wald Confidence Limits | |
| Gender 2 vs 1 | 1.212 | 0.552 | 2.664 |
| History_of_heart_fai 2 vs 1 | 0.772 | 0.226 | 2.633 |
| History_of_chronic_r 2 vs 1 | 0.162 | 0.047 | 0.556 |
| DM 2 vs 1 | 0.783 | 0.450 | 1.363 |
| HTN 2 vs 1 | 1.904 | 1.047 | 3.461 |
| CHF_Killip_Class 2 vs 1 | 17.136 | 9.057 | 32.423 |
| CHF_Killip_Class 3 vs 1 | 50.598 | 18.005 | 142.186 |
| CHF_Killip_Class 4 vs 1 | 3.356 | 1.123 | 10.033 |
| Age | 1.013 | 0.991 | 1.037 |
| SBP_mmgH_ | 0.996 | 0.987 | 1.006 |
| HR_bpm_ | 1.020 | 1.008 | 1.032 |
| CAD 1 vs 2 | 1.196 | 0.652 | 2.194 |
| Hypercholestrolemia 2 vs 1 | 0.626 | 0.353 | 1.110 |
| Cardiac_arrest 2 vs 1 | 0.392 | 0.153 | 1.005 |
| History_of_stroke 2 vs 1 | 1.196 | 0.217 | 6.578 |
| Type_of_STEMI 2 vs 1 | 1.204 | 0.639 | 2.270 |
| Type_of_STEMI 3 vs 1 | 1.942 | 0.656 | 5.753 |
| Echo_Options 2 vs 1 | 5.162 | 1.608 | 16.569 |
| Echo_Options 3 vs 1 | 8.634 | 2.634 | 28.306 |
| Echo_Options 4 vs 1 | 31.439 | 9.105 | 108.554 |

| Association of Predicted Probabilities and Observed Responses | | | |
|---|---|---|---|
| Percent Concordant | 92.1 | Somers' D | 0.843 |
| Percent Discordant | 7.9 | Gamma | 0.843 |
| Percent Tied | 0.0 | Tau-a | 0.167 |
| Pairs | 120663 | c | 0.921 |
