# Supplementary material for: The second survey of the Saudi Acute Myocardial Infarction Registry Program: Main results and temporal changes in care (STARS-2 program)
Source: PLoS One. 2025 Sep 2;20(9):e0331215. doi: 10.1371/journal.pone.0331215 (PMC12404464; doi:10.1371/journal.pone.0331215)
Supplement: S1 Data — (ZIP) [file pone.0331215.s011.zip › Raw data/medications upon 24hrs.pdf]

*Medications upon admissionvar definitions:141 Heparin, 142, Glycoprotien inh, 148 SGLT-2,150 GLP-1, 152 Icosapest ethyl, 153 omega-3, 154 genfibozil,155 fenofibrate, 207 Icosapent ethyl, 208 omega-3, 209 Gemfibrate, 210 fenofibrate

The FREQ Procedure

Frequency<br>Percent<br>Row Pct<br>Col Pct

Table of STEMI_NSTEMI by ACE_I_or_ARB_24hrs_of_Hospital_A

	ACE_I_or_ARB_24hrs_of_Hospital_A(ACE-I or ARB-24hrs of Hospital Admission)		
STEMI_NSTEMI(STEMI/NSTEMI)	1	2	Total
1	901<br>33.49<br>68.62<br>47.03	412<br>15.32<br>31.38<br>53.23	1313<br>48.81
2	1015<br>37.73<br>73.71<br>52.97	362<br>13.46<br>26.29<br>46.77	1377<br>51.19
Total	1916<br>71.23	774<br>28.77	2690<br>100.00
Frequency Missing = 1			

Statistics for Table of STEMI_NSTEMI by ACE_I_or_ARB_24hrs_of_Hospital_A

Statistic	DF	Value	Prob
Chi-Square	1	8.4950	0.0036
Likelihood Ratio Chi-Square	1	8.4963	0.0036
Continuity Adj. Chi-Square	1	8.2485	0.0041
Mantel-Haenszel Chi-Square	1	8.4918	0.0036
Phi Coefficient		-0.0562	
Contingency Coefficient		0.0561	
Cramer's V		-0.0562	

Fisher's Exact Test	
Cell (1,1) Frequency (F)	901
Left-sided Pr <= F	0.0020
Right-sided Pr >= F	0.9984
Table Probability (P)	0.0005
Two-sided Pr <= P	0.0038

Sample Size = 2690
Frequency Missing = 1

*Medications upon admissionvar definitions:141 Heparin, 142, Glycoprotien inh, 148 SGLT-2,150 GLP-1, 152 Icosapest ethyl, 153 omega-3, 154 genfibozil,155 fenofibrate, 207 Icosapent ethyl, 208 omega-3, 209 Gemfibrate, 210 fenofibrate

The FREQ Procedure

Frequency<br>Percent<br>Row Pct<br>Col Pct

Table of STEMI_NSTEMI by Aldosterone_Inhibitor__Spironola

	Aldosterone_Inhibitor__Spironola(Aldosterone Inhibitor (Spironolactone)-24hrs of Hospital Admission)		
STEMI_NSTEMI(STEMI/NSTEMI)	1	2	Total
1	164<br>6.10<br>12.49<br>46.99	1149<br>42.71<br>87.51<br>49.08	1313<br>48.81
2	185<br>6.88<br>13.44<br>53.01	1192<br>44.31<br>86.56<br>50.92	1377<br>51.19
Total	349<br>12.97	2341<br>87.03	2690<br>100.00
Frequency Missing = 1			

Statistics for Table of STEMI_NSTEMI by Aldosterone_Inhibitor__Spironola

Statistic	DF	Value	Prob
Chi-Square	1	0.5311	0.4662
Likelihood Ratio Chi-Square	1	0.5314	0.4660
Continuity Adj. Chi-Square	1	0.4507	0.5020
Mantel-Haenszel Chi-Square	1	0.5309	0.4662
Phi Coefficient		-0.0141	
Contingency Coefficient		0.0140	
Cramer's V		-0.0141	

Fisher's Exact Test	
Cell (1,1) Frequency (F)	164
Left-sided Pr <= F	0.2511
Right-sided Pr >= F	0.7841
Table Probability (P)	0.0351
Two-sided Pr <= P	0.4911

Sample Size = 2690
Frequency Missing = 1

*Medications upon admissionvar definitions:141 Heparin, 142, Glycoprotien inh, 148 SGLT-2,150 GLP-1, 152 Icosapest ethyl, 153 omega-3, 154 genfibozil,155 fenofibrate, 207 Icosapent ethyl, 208 omega-3, 209 Gemfibrate, 210 fenofibrate

The FREQ Procedure

Frequency<br>Percent<br>Row Pct<br>Col Pct

Table of STEMI_NSTEMI by Aspirin_24hrs_of_Hospital_Admiss			
	Aspirin_24hrs_of_Hospital_Admiss(Aspirin-24hrs of Hospital Admission)		
STEMI_NSTEMI(STEMI/NSTEMI)	1	2	Total
1	1311<br>48.74<br>99.85<br>49.06	2<br>0.07<br>0.15<br>11.11	1313<br>48.81
2	1361<br>50.59<br>98.84<br>50.94	16<br>0.59<br>1.16<br>88.89	1377<br>51.19
Total	2672<br>99.33	18<br>0.67	2690<br>100.00
Frequency Missing = 1			

Statistics for Table of STEMI_NSTEMI by Aspirin_24hrs_of_Hospital_Admiss

Statistic	DF	Value	Prob
Chi-Square	1	10.3077	0.0013
Likelihood Ratio Chi-Square	1	11.8082	0.0006
Continuity Adj. Chi-Square	1	8.8446	0.0029
Mantel-Haenszel Chi-Square	1	10.3038	0.0013
Phi Coefficient		0.0619	
Contingency Coefficient		0.0618	
Cramer's V		0.0619	

Fisher's Exact Test	
Cell (1,1) Frequency (F)	1311
Left-sided Pr <= F	0.9999
Right-sided Pr >= F	0.0009
Table Probability (P)	0.0008
Two-sided Pr <= P	0.0013

Sample Size = 2690
Frequency Missing = 1

***Medications upon admissionvar definitions:141 Heparin, 142, Glycoprotien inh, 148 SGLT-2,150 GLP-1, 152 Icosapest ethyl, 153 omega-3, 154 genfibozil,155 fenofibrate, 207 Icosapent ethyl, 208 omega-3, 209 Gemfibrate, 210 fenofibrate**

The FREQ Procedure

Frequency<br>Percent<br>Row Pct<br>Col Pct

Table of STEMI_NSTEMI by Beta_Blockers_24hrs_of_Hospital			
	Beta_Blockers_24hrs_of_Hospital(Beta-Blockers-24hrs of Hospital Admission)		
STEMI_NSTEMI(STEMI/NSTEMI)	1	2	Total
1	937<br>34.83<br>71.36<br>44.62	376<br>13.98<br>28.64<br>63.73	1313<br>48.81
2	1163<br>43.23<br>84.46<br>55.38	214<br>7.96<br>15.54<br>36.27	1377<br>51.19
Total	2100<br>78.07	590<br>21.93	2690<br>100.00
Frequency Missing = 1			

Statistics for Table of STEMI_NSTEMI by Beta_Blockers_24hrs_of_Hospital

Statistic	DF	Value	Prob
Chi-Square	1	67.3187	<.0001
Likelihood Ratio Chi-Square	1	67.9041	<.0001
Continuity Adj. Chi-Square	1	66.5560	<.0001
Mantel-Haenszel Chi-Square	1	67.2937	<.0001
Phi Coefficient		-0.1582	
Contingency Coefficient		0.1563	
Cramer's V		-0.1582	

Fisher's Exact Test	
Cell (1,1) Frequency (F)	937
Left-sided Pr <= F	<.0001
Right-sided Pr >= F	1.0000
Table Probability (P)	<.0001
Two-sided Pr <= P	<.0001

Sample Size = 2690
Frequency Missing = 1

*Medications upon admissionvar definitions:141 Heparin, 142, Glycoprotien inh, 148 SGLT-2,150 GLP-1, 152 Icosapest ethyl, 153 omega-3, 154 genfibozil,155 fenofibrate, 207 Icosapent ethyl, 208 omega-3, 209 Gemfibrate, 210 fenofibrate

The FREQ Procedure

Frequency<br>Percent<br>Row Pct<br>Col Pct

Table of STEMI_NSTEMI by Bivaluridin____24_hours_of_hopsi			
	Bivaluridin____24_hours_of_hopsi(Bivaluridin -- 24 hours of hopsital addmission)		
STEMI_NSTEMI(STEMI/NSTEMI)	1	2	Total
1	9<br>0.33<br>0.69<br>45.00	1304<br>48.48<br>99.31<br>48.84	1313<br>48.81
2	11<br>0.41<br>0.80<br>55.00	1366<br>50.78<br>99.20<br>51.16	1377<br>51.19
Total	20<br>0.74	2670<br>99.26	2690<br>100.00
Frequency Missing = 1			

Statistics for Table of STEMI_NSTEMI by Bivaluridin____24_hours_of_hopsi

Statistic	DF	Value	Prob
Chi-Square	1	0.1171	0.7322
Likelihood Ratio Chi-Square	1	0.1173	0.7319
Continuity Adj. Chi-Square	1	0.0138	0.9063
Mantel-Haenszel Chi-Square	1	0.1170	0.7323
Phi Coefficient		-0.0066	
Contingency Coefficient		0.0066	
Cramer's V		-0.0066	

Fisher's Exact Test	
Cell (1,1) Frequency (F)	9
Left-sided Pr <= F	0.4541
Right-sided Pr >= F	0.7135
Table Probability (P)	0.1676
Two-sided Pr <= P	0.8241

Sample Size = 2690
Frequency Missing = 1

*Medications upon admissionvar definitions:141 Heparin, 142, Glycoprotien inh, 148 SGLT-2,150 GLP-1, 152 Icosapest ethyl, 153 omega-3, 154 genfibozil,155 fenofibrate, 207 Icosapent ethyl, 208 omega-3, 209 Gemfibrate, 210 fenofibrate

The FREQ Procedure

Frequency<br>Percent<br>Row Pct<br>Col Pct

Table of STEMI_NSTEMI by Clopidogrel__24hrs_of_Hospital_A			
	Clopidogrel__24hrs_of_Hospital_A(Clopidogrel -24hrs of Hospital Admission)		
STEMI_NSTEMI(STEMI/NSTEMI)	1	2	Total
1	937<br>34.83<br>71.36<br>47.78	376<br>13.98<br>28.64<br>51.58	1313<br>48.81
2	1024<br>38.07<br>74.36<br>52.22	353<br>13.12<br>25.64<br>48.42	1377<br>51.19
Total	1961<br>72.90	729<br>27.10	2690<br>100.00
Frequency Missing = 1			

Statistics for Table of STEMI_NSTEMI by Clopidogrel__24hrs_of_Hospital_A

Statistic	DF	Value	Prob
Chi-Square	1	3.0645	0.0800
Likelihood Ratio Chi-Square	1	3.0640	0.0800
Continuity Adj. Chi-Square	1	2.9144	0.0878
Mantel-Haenszel Chi-Square	1	3.0633	0.0801
Phi Coefficient		-0.0338	
Contingency Coefficient		0.0337	
Cramer's V		-0.0338	

Fisher's Exact Test	
Cell (1,1) Frequency (F)	937
Left-sided Pr <= F	0.0439
Right-sided Pr >= F	0.9636
Table Probability (P)	0.0075
Two-sided Pr <= P	0.0827

Sample Size = 2690
Frequency Missing = 1

*Medications upon admissionvar definitions:141 Heparin, 142, Glycoprotien inh, 148 SGLT-2,150 GLP-1, 152 Icosapest ethyl, 153 omega-3, 154 genfibozil,155 fenofibrate, 207 Icosapent ethyl, 208 omega-3, 209 Gemfibrate, 210 fenofibrate

The FREQ Procedure

Frequency<br>Percent<br>Row Pct<br>Col Pct

Table of STEMI_NSTEMI by Insulin__24hrs_of_Hospital_Admis			
	Insulin__24hrs_of_Hospital_Admis(Insulin--24hrs of Hospital Admission)		
STEMI_NSTEMI(STEMI/NSTEMI)	1	2	Total
1	547<br>20.33<br>41.66<br>42.83	766<br>28.48<br>58.34<br>54.21	1313<br>48.81
2	730<br>27.14<br>53.01<br>57.17	647<br>24.05<br>46.99<br>45.79	1377<br>51.19
Total	1277<br>47.47	1413<br>52.53	2690<br>100.00
Frequency Missing = 1			

Statistics for Table of STEMI_NSTEMI by Insulin__24hrs_of_Hospital_Admis

Statistic	DF	Value	Prob
Chi-Square	1	34.7437	<.0001
Likelihood Ratio Chi-Square	1	34.8263	<.0001
Continuity Adj. Chi-Square	1	34.2899	<.0001
Mantel-Haenszel Chi-Square	1	34.7308	<.0001
Phi Coefficient		-0.1136	
Contingency Coefficient		0.1129	
Cramer's V		-0.1136	

Fisher's Exact Test	
Cell (1,1) Frequency (F)	547
Left-sided Pr <= F	<.0001
Right-sided Pr >= F	1.0000
Table Probability (P)	<.0001
Two-sided Pr <= P	<.0001

Sample Size = 2690
Frequency Missing = 1

*Medications upon admissionvar definitions:141 Heparin, 142, Glycoprotien inh, 148 SGLT-2,150 GLP-1, 152 Icosapest ethyl, 153 omega-3, 154 genfibozil,155 fenofibrate, 207 Icosapent ethyl, 208 omega-3, 209 Gemfibrate, 210 fenofibrate

The FREQ Procedure

Frequency<br>Percent<br>Row Pct<br>Col Pct

Table of STEMI_NSTEMI by Novel_oral_anticoagulants__NOACs			
	Novel_oral_anticoagulants__NOACs(Novel oral anticoagulants (NOACs)--24hrs of Hospital Admission)		
STEMI_NSTEMI(STEMI/NSTEMI)	1	2	Total
1	6<br>0.22<br>0.46<br>54.55	1307<br>48.59<br>99.54<br>48.79	1313<br>48.81
2	5<br>0.19<br>0.36<br>45.45	1372<br>51.00<br>99.64<br>51.21	1377<br>51.19
Total	11<br>0.41	2679<br>99.59	2690<br>100.00
Frequency Missing = 1			

Statistics for Table of STEMI_NSTEMI by Novel_oral_anticoagulants__NOACs

Statistic	DF	Value	Prob
Chi-Square	1	0.1454	0.7030
Likelihood Ratio Chi-Square	1	0.1455	0.7029
Continuity Adj. Chi-Square	1	0.0063	0.9370
Mantel-Haenszel Chi-Square	1	0.1453	0.7030
Phi Coefficient		0.0074	
Contingency Coefficient		0.0074	
Cramer's V		0.0074	

Fisher's Exact Test	
Cell (1,1) Frequency (F)	6
Left-sided Pr <= F	0.7522
Right-sided Pr >= F	0.4678
Table Probability (P)	0.2200
Two-sided Pr <= P	0.7693

Sample Size = 2690
Frequency Missing = 1

*Medications upon admissionvar definitions:141 Heparin, 142, Glycoprotien inh, 148 SGLT-2,150 GLP-1, 152 Icosapest ethyl, 153 omega-3, 154 genfibozil,155 fenofibrate, 207 Icosapent ethyl, 208 omega-3, 209 Gemfibrate, 210 fenofibrate

The FREQ Procedure

Frequency<br>Percent<br>Row Pct<br>Col Pct

Table of STEMI_NSTEMI by Oral_hypoglycemic_agents__24hrs			
	Oral_hypoglycemic_agents__24hrs(Oral hypoglycemic agents--24hrs of Hospital Admission)		
STEMI_NSTEMI(STEMI/NSTEMI)	1	2	Total
1	90<br>3.35<br>6.85<br>34.48	1223<br>45.46<br>93.15<br>50.35	1313<br>48.81
2	171<br>6.36<br>12.42<br>65.52	1206<br>44.83<br>87.58<br>49.65	1377<br>51.19
Total	261<br>9.70	2429<br>90.30	2690<br>100.00
Frequency Missing = 1			

Statistics for Table of STEMI_NSTEMI by Oral_hypoglycemic_agents__24hrs

Statistic	DF	Value	Prob
Chi-Square	1	23.7477	<.0001
Likelihood Ratio Chi-Square	1	24.1540	<.0001
Continuity Adj. Chi-Square	1	23.1169	<.0001
Mantel-Haenszel Chi-Square	1	23.7388	<.0001
Phi Coefficient		-0.0940	
Contingency Coefficient		0.0935	
Cramer's V		-0.0940	

Fisher's Exact Test	
Cell (1,1) Frequency (F)	90
Left-sided Pr <= F	<.0001
Right-sided Pr >= F	1.0000
Table Probability (P)	<.0001
Two-sided Pr <= P	<.0001

Sample Size = 2690
Frequency Missing = 1

*Medications upon admissionvar definitions:141 Heparin, 142, Glycoprotien inh, 148 SGLT-2,150 GLP-1, 152 Icosapest ethyl, 153 omega-3, 154 genfibozil,155 fenofibrate, 207 Icosapent ethyl, 208 omega-3, 209 Gemfibrate, 210 fenofibrate

The FREQ Procedure

Frequency<br>Percent<br>Row Pct<br>Col Pct

Table of STEMI_NSTEMI by PCSk9_inhibitors__24hrs_of_Hospi			
	PCSk9_inhibitors__24hrs_of_Hospi(PCSk9 inhibitors--24hrs of Hospital Admission)		
STEMI_NSTEMI(STEMI/NSTEMI)	1	2	Total
1	36<br>1.34<br>2.74<br>49.32	1277<br>47.47<br>97.26<br>48.80	1313<br>48.81
2	37<br>1.38<br>2.69<br>50.68	1340<br>49.81<br>97.31<br>51.20	1377<br>51.19
Total	73<br>2.71	2617<br>97.29	2690<br>100.00
Frequency Missing = 1			

Statistics for Table of STEMI_NSTEMI by PCSk9_inhibitors__24hrs_of_Hospi

Statistic	DF	Value	Prob
Chi-Square	1	0.0076	0.9303
Likelihood Ratio Chi-Square	1	0.0076	0.9303
Continuity Adj. Chi-Square	1	0.0000	1.0000
Mantel-Haenszel Chi-Square	1	0.0076	0.9303
Phi Coefficient		0.0017	
Contingency Coefficient		0.0017	
Cramer's V		0.0017	

Fisher's Exact Test	
Cell (1,1) Frequency (F)	36
Left-sided Pr <= F	0.5819
Right-sided Pr >= F	0.5121
Table Probability (P)	0.0940
Two-sided Pr <= P	1.0000

Sample Size = 2690
Frequency Missing = 1

*Medications upon admissionvar definitions:141 Heparin, 142, Glycoprotien inh, 148 SGLT-2,150 GLP-1, 152 Icosapest ethyl, 153 omega-3, 154 genfibozil,155 fenofibrate, 207 Icosapent ethyl, 208 omega-3, 209 Gemfibrate, 210 fenofibrate

The FREQ Procedure

Frequency<br>Percent<br>Row Pct<br>Col Pct

Table of STEMI_NSTEMI by Prasugrel__24hrs_of_Hospital_Adm			
	Prasugrel__24hrs_of_Hospital_Adm(Prasugrel -24hrs of Hospital Admission)		
STEMI_NSTEMI(STEMI/NSTEMI)	1	2	Total
1	8<br>0.30<br>0.61<br>61.54	1305<br>48.51<br>99.39<br>48.75	1313<br>48.81
2	5<br>0.19<br>0.36<br>38.46	1372<br>51.00<br>99.64<br>51.25	1377<br>51.19
Total	13<br>0.48	2677<br>99.52	2690<br>100.00
Frequency Missing = 1			

Statistics for Table of STEMI_NSTEMI by Prasugrel__24hrs_of_Hospital_Adm

Statistic	DF	Value	Prob
Chi-Square	1	0.8470	0.3574
Likelihood Ratio Chi-Square	1	0.8528	0.3558
Continuity Adj. Chi-Square	1	0.4124	0.5207
Mantel-Haenszel Chi-Square	1	0.8467	0.3575
Phi Coefficient		0.0177	
Contingency Coefficient		0.0177	
Cramer's V		0.0177	

Fisher's Exact Test	
Cell (1,1) Frequency (F)	8
Left-sided Pr <= F	0.8850
Right-sided Pr >= F	0.2608
Table Probability (P)	0.1458
Two-sided Pr <= P	0.4133

Sample Size = 2690
Frequency Missing = 1

***Medications upon admissionvar definitions:141 Heparin, 142, Glycoprotien inh, 148 SGLT-2,150 GLP-1, 152 Icosapest ethyl, 153 omega-3, 154 genfibozil,155 fenofibrate, 207 Icosapent ethyl, 208 omega-3, 209 Gemfibrate, 210 fenofibrate**

The FREQ Procedure

Frequency<br>Percent<br>Row Pct<br>Col Pct

Table of STEMI_NSTEMI by Statins_24hrs_of_Hospital_Admiss			
	Statins_24hrs_of_Hospital_Admiss(Statins-24hrs of Hospital Admission)		
STEMI_NSTEMI(STEMI/NSTEMI)	1	2	Total
1	1235<br>45.91<br>94.06<br>48.34	78<br>2.90<br>5.94<br>57.78	1313<br>48.81
2	1320<br>49.07<br>95.86<br>51.66	57<br>2.12<br>4.14<br>42.22	1377<br>51.19
Total	2555<br>94.98	135<br>5.02	2690<br>100.00
Frequency Missing = 1			

Statistics for Table of STEMI_NSTEMI by Statins_24hrs_of_Hospital_Admiss

Statistic	DF	Value	Prob
Chi-Square	1	4.5744	0.0325
Likelihood Ratio Chi-Square	1	4.5855	0.0322
Continuity Adj. Chi-Square	1	4.2043	0.0403
Mantel-Haenszel Chi-Square	1	4.5727	0.0325
Phi Coefficient		-0.0412	
Contingency Coefficient		0.0412	
Cramer's V		-0.0412	

Fisher's Exact Test	
Cell (1,1) Frequency (F)	1235
Left-sided Pr <= F	0.0201
Right-sided Pr >= F	0.9871
Table Probability (P)	0.0072
Two-sided Pr <= P	0.0341

Sample Size = 2690
Frequency Missing = 1

*Medications upon admissionvar definitions:141 Heparin, 142, Glycoprotien inh, 148 SGLT-2,150 GLP-1, 152 Icosapest ethyl, 153 omega-3, 154 genfibozil,155 fenofibrate, 207 Icosapent ethyl, 208 omega-3, 209 Gemfibrate, 210 fenofibrate

The FREQ Procedure

Frequency<br>Percent<br>Row Pct<br>Col Pct

Table of STEMI_NSTEMI by Ticagrel_24hrs_of_Hospital_Admis			
	Ticagrel_24hrs_of_Hospital_Admis(Ticagrel-24hrs of Hospital Admission)		
STEMI_NSTEMI(STEMI/NSTEMI)	1	2	Total
1	397<br>14.76<br>30.24<br>54.99	916<br>34.05<br>69.76<br>46.54	1313<br>48.81
2	325<br>12.08<br>23.60<br>45.01	1052<br>39.11<br>76.40<br>53.46	1377<br>51.19
Total	722<br>26.84	1968<br>73.16	2690<br>100.00
Frequency Missing = 1			

Statistics for Table of STEMI_NSTEMI by Ticagrel_24hrs_of_Hospital_Admis

Statistic	DF	Value	Prob
Chi-Square	1	15.0643	0.0001
Likelihood Ratio Chi-Square	1	15.0751	0.0001
Continuity Adj. Chi-Square	1	14.7283	0.0001
Mantel-Haenszel Chi-Square	1	15.0587	0.0001
Phi Coefficient		0.0748	
Contingency Coefficient		0.0746	
Cramer's V		0.0748	

Fisher's Exact Test	
Cell (1,1) Frequency (F)	397
Left-sided Pr <= F	1.0000
Right-sided Pr >= F	<.0001
Table Probability (P)	<.0001
Two-sided Pr <= P	0.0001

Sample Size = 2690
Frequency Missing = 1

*Medications upon admissionvar definitions:141 Heparin, 142, Glycoprotien inh, 148 SGLT-2,150 GLP-1, 152 Icosapest ethyl, 153 omega-3, 154 genfibozil,155 fenofibrate, 207 Icosapent ethyl, 208 omega-3, 209 Gemfibrate, 210 fenofibrate

The FREQ Procedure

Frequency<br>Percent<br>Row Pct<br>Col Pct

Table of STEMI_NSTEMI by VAR141			
	VAR141(Heparins (UH or LMWH)--24 hours of hopsital addmission)		
STEMI_NSTEMI(STEMI/NSTEMI)	1	2	Total
1	1168<br>43.42<br>88.96<br>48.59	145<br>5.39<br>11.04<br>50.70	1313<br>48.81
2	1236<br>45.95<br>89.76<br>51.41	141<br>5.24<br>10.24<br>49.30	1377<br>51.19
Total	2404<br>89.37	286<br>10.63	2690<br>100.00
Frequency Missing = 1			

Statistics for Table of STEMI_NSTEMI by VAR141

Statistic	DF	Value	Prob
Chi-Square	1	0.4570	0.4990
Likelihood Ratio Chi-Square	1	0.4568	0.4991
Continuity Adj. Chi-Square	1	0.3763	0.5396
Mantel-Haenszel Chi-Square	1	0.4568	0.4991
Phi Coefficient		-0.0130	
Contingency Coefficient		0.0130	
Cramer's V		-0.0130	

Fisher's Exact Test	
Cell (1,1) Frequency (F)	1168
Left-sided Pr <= F	0.2697
Right-sided Pr >= F	0.7699
Table Probability (P)	0.0397
Two-sided Pr <= P	0.5316

Sample Size = 2690
Frequency Missing = 1

*Medications upon admissionvar definitions:141 Heparin, 142, Glycoprotien inh, 148 SGLT-2,150 GLP-1, 152 Icosapest ethyl, 153 omega-3, 154 genfibozil,155 fenofibrate, 207 Icosapent ethyl, 208 omega-3, 209 Gemfibrate, 210 fenofibrate

The FREQ Procedure

Frequency<br>Percent<br>Row Pct<br>Col Pct

Table of STEMI_NSTEMI by VAR142			
	VAR142(GP 2b/3a inhibitors -- 24 hours of hopsital addmission)		
STEMI_NSTEMI(STEMI/NSTEMI)	1	2	Total
1	142<br>5.28<br>10.81<br>71.36	1171<br>43.53<br>89.19<br>47.01	1313<br>48.81
2	57<br>2.12<br>4.14<br>28.64	1320<br>49.07<br>95.86<br>52.99	1377<br>51.19
Total	199<br>7.40	2491<br>92.60	2690<br>100.00
Frequency Missing = 1			

Statistics for Table of STEMI_NSTEMI by VAR142

Statistic	DF	Value	Prob
Chi-Square	1	43.7211	<.0001
Likelihood Ratio Chi-Square	1	44.8950	<.0001
Continuity Adj. Chi-Square	1	42.7521	<.0001
Mantel-Haenszel Chi-Square	1	43.7048	<.0001
Phi Coefficient		0.1275	
Contingency Coefficient		0.1265	
Cramer's V		0.1275	

Fisher's Exact Test	
Cell (1,1) Frequency (F)	142
Left-sided Pr <= F	1.0000
Right-sided Pr >= F	<.0001
Table Probability (P)	<.0001
Two-sided Pr <= P	<.0001

Sample Size = 2690
Frequency Missing = 1

*Medications upon admissionvar definitions:141 Heparin, 142, Glycoprotien inh, 148 SGLT-2,150 GLP-1, 152 Icosapest ethyl, 153 omega-3, 154 genfibozil,155 fenofibrate, 207 Icosapent ethyl, 208 omega-3, 209 Gemfibrate, 210 fenofibrate

The FREQ Procedure

Frequency<br>Percent<br>Row Pct<br>Col Pct

Table of STEMI_NSTEMI by VAR150			
	VAR150(GLP-1 agonists--24hrs of Hospital Admission)		
STEMI_NSTEMI(STEMI/NSTEMI)	1	2	Total
1	0<br>0.00<br>0.00<br>0.00	1313<br>48.81<br>100.00<br>48.88	1313<br>48.81
2	4<br>0.15<br>0.29<br>100.00	1373<br>51.04<br>99.71<br>51.12	1377<br>51.19
Total	4<br>0.15	2686<br>99.85	2690<br>100.00
Frequency Missing = 1			

Statistics for Table of STEMI_NSTEMI by VAR150

Statistic	DF	Value	Prob
Chi-Square	1	3.8198	0.0507
Likelihood Ratio Chi-Square	1	5.3628	0.0206
Continuity Adj. Chi-Square	1	2.1139	0.1460
Mantel-Haenszel Chi-Square	1	3.8183	0.0507
Phi Coefficient		-0.0377	
Contingency Coefficient		0.0377	
Cramer's V		-0.0377	
WARNING: 50% of the cells have expected counts less than 5. Chi-Square may not be a valid test.			

Fisher's Exact Test	
Cell (1,1) Frequency (F)	0
Left-sided Pr <= F	0.0685
Right-sided Pr >= F	1.0000
Table Probability (P)	0.0685
Two-sided Pr <= P	0.1251

Sample Size = 2690
Frequency Missing = 1

*Medications upon admissionvar definitions:141 Heparin, 142, Glycoprotien inh, 148 SGLT-2,150 GLP-1, 152 Icosapest ethyl, 153 omega-3, 154 genfibozil,155 fenofibrate, 207 Icosapent ethyl, 208 omega-3, 209 Gemfibrate, 210 fenofibrate

The FREQ Procedure

Frequency<br>Percent<br>Row Pct<br>Col Pct

Table of STEMI_NSTEMI by VAR152			
	VAR152(Icosapent ethyl (e.g. VASCEPA®)--24hrs of Hospital Admission)		
STEMI_NSTEMI(STEMI/NSTEMI)	1	2	Total
1	0<br>0.00<br>0.00<br>0.00	1313<br>48.81<br>100.00<br>48.83	1313<br>48.81
2	1<br>0.04<br>0.07<br>100.00	1376<br>51.15<br>99.93<br>51.17	1377<br>51.19
Total	1<br>0.04	2689<br>99.96	2690<br>100.00
Frequency Missing = 1			

*Medications upon admissionvar definitions:141 Heparin, 142, Glycoprotien inh, 148 SGLT-2,150 GLP-1, 152 Icosapest ethyl, 153 omega-3, 154 genfibozil,155 fenofibrate, 207 Icosapent ethyl, 208 omega-3, 209 Gemfibrate, 210 fenofibrate

The FREQ Procedure

Statistics for Table of STEMI_NSTEMI by VAR152

Statistic	DF	Value	Prob
Chi-Square	1	0.9539	0.3287
Likelihood Ratio Chi-Square	1	1.3396	0.2471
Continuity Adj. Chi-Square	1	0.0000	1.0000
Mantel-Haenszel Chi-Square	1	0.9535	0.3288
Phi Coefficient		-0.0188	
Contingency Coefficient		0.0188	
Cramer's V		-0.0188	
WARNING: 50% of the cells have expected counts less than 5. Chi-Square may not be a valid test.			

Fisher's Exact Test	
Cell (1,1) Frequency (F)	0
Left-sided Pr <= F	0.5119
Right-sided Pr >= F	1.0000
Table Probability (P)	0.5119
Two-sided Pr <= P	1.0000

Sample Size = 2690
Frequency Missing = 1

Frequency<br>Percent<br>Row Pct<br>Col Pct

Table of STEMI_NSTEMI by VAR153			
	VAR153(Omega-3-acid ethyl esters 90 (e.g.Omacor®)--24hrs of Hospital Admission)		
STEMI_NSTEMI(STEMI/NSTEMI)	1	2	Total
1	3<br>0.11<br>0.23<br>37.50	1310<br>48.70<br>99.77<br>48.84	1313<br>48.81
2	5<br>0.19<br>0.36<br>62.50	1372<br>51.00<br>99.64<br>51.16	1377<br>51.19
Total	8<br>0.30	2682<br>99.70	2690<br>100.00
Frequency Missing = 1			

*Medications upon admissionvar definitions:141 Heparin, 142, Glycoprotien inh, 148 SGLT-2,150 GLP-1, 152 Icosapest ethyl, 153 omega-3, 154 genfibozil,155 fenofibrate, 207 Icosapent ethyl, 208 omega-3, 209 Gemfibrate, 210 fenofibrate

The FREQ Procedure

Statistics for Table of STEMI_NSTEMI by VAR153

Statistic	DF	Value	Prob
Chi-Square	1	0.4108	0.5216
Likelihood Ratio Chi-Square	1	0.4159	0.5190
Continuity Adj. Chi-Square	1	0.0822	0.7743
Mantel-Haenszel Chi-Square	1	0.4107	0.5216
Phi Coefficient		-0.0124	
Contingency Coefficient		0.0124	
Cramer's V		-0.0124	
WARNING: 50% of the cells have expected counts less than 5. Chi-Square may not be a valid test.			

Fisher's Exact Test	
Cell (1,1) Frequency (F)	3
Left-sided Pr <= F	0.3894
Right-sided Pr >= F	0.8397
Table Probability (P)	0.2291
Two-sided Pr <= P	0.7268

Sample Size = 2690
Frequency Missing = 1

Frequency<br>Percent<br>Row Pct<br>Col Pct

Table of STEMI_NSTEMI by VAR154			
	VAR154(Gemfibrozil (e.g. Lopid®)--24hrs of Hospital Admission)		
STEMI_NSTEMI(STEMI/NSTEMI)	1	2	Total
1	0<br>0.00<br>0.00<br>0.00	1313<br>48.81<br>100.00<br>48.85	1313<br>48.81
2	2<br>0.07<br>0.15<br>100.00	1375<br>51.12<br>99.85<br>51.15	1377<br>51.19
Total	2<br>0.07	2688<br>99.93	2690<br>100.00
Frequency Missing = 1			

***Medications upon admissionvar definitions:141 Heparin, 142, Glycoprotien inh, 148 SGLT-2,150 GLP-1, 152 Icosapest ethyl, 153 omega-3, 154 genfibozil,155 fenofibrate, 207 Icosapent ethyl, 208 omega-3, 209 Gemfibrate, 210 fenofibrate**

The FREQ Procedure

Statistics for Table of STEMI_NSTEMI by VAR154

Statistic	DF	Value	Prob
Chi-Square	1	1.9085	0.1671
Likelihood Ratio Chi-Square	1	2.6800	0.1016
Continuity Adj. Chi-Square	1	0.4541	0.5004
Mantel-Haenszel Chi-Square	1	1.9078	0.1672
Phi Coefficient		-0.0266	
Contingency Coefficient		0.0266	
Cramer's V		-0.0266	
WARNING: 50% of the cells have expected counts less than 5. Chi-Square may not be a valid test.			

Fisher's Exact Test	
Cell (1,1) Frequency (F)	0
Left-sided Pr <= F	0.2619
Right-sided Pr >= F	1.0000
Table Probability (P)	0.2619
Two-sided Pr <= P	0.5001

Sample Size = 2690
Frequency Missing = 1

Frequency<br>Percent<br>Row Pct<br>Col Pct

Table of STEMI_NSTEMI by VAR155			
	VAR155(Fenofibrate (e.g. Lipanthyl®)--24hrs of Hospital Admission)		
STEMI_NSTEMI(STEMI/NSTEMI)	1	2	Total
1	9<br>0.33<br>0.69<br>37.50	1304<br>48.48<br>99.31<br>48.91	1313<br>48.81
2	15<br>0.56<br>1.09<br>62.50	1362<br>50.63<br>98.91<br>51.09	1377<br>51.19
Total	24<br>0.89	2666<br>99.11	2690<br>100.00
Frequency Missing = 1			

*Medications upon admissionvar definitions:141 Heparin, 142, Glycoprotien inh, 148 SGLT-2,150 GLP-1, 152 Icosapest ethyl, 153 omega-3, 154 genfibozil,155 fenofibrate, 207 Icosapent ethyl, 208 omega-3, 209 Gemfibrate, 210 fenofibrate

The FREQ Procedure

Statistics for Table of STEMI_NSTEMI by VAR155

Statistic	DF	Value	Prob
Chi-Square	1	1.2398	0.2655
Likelihood Ratio Chi-Square	1	1.2551	0.2626
Continuity Adj. Chi-Square	1	0.8252	0.3637
Mantel-Haenszel Chi-Square	1	1.2394	0.2656
Phi Coefficient		-0.0215	
Contingency Coefficient		0.0215	
Cramer's V		-0.0215	

Fisher's Exact Test	
Cell (1,1) Frequency (F)	9
Left-sided Pr <= F	0.1821
Right-sided Pr >= F	0.9071
Table Probability (P)	0.0892
Two-sided Pr <= P	0.3086

Sample Size = 2690
Frequency Missing = 1
