# Supplementary material for: The second survey of the Saudi Acute Myocardial Infarction Registry Program: Main results and temporal changes in care (STARS-2 program)
Source: PLoS One. 2025 Sep 2;20(9):e0331215. doi: 10.1371/journal.pone.0331215 (PMC12404464; doi:10.1371/journal.pone.0331215)
Supplement: S1 Data — (ZIP) [file pone.0331215.s011.zip › Raw data/other results.pdf]

The FREQ Procedure

Frequency<br>Percent<br>Row Pct<br>Col Pct

Table of STEMI_NSTEMI by ACE_I_or_ARB_At_Hospital_Dischar			
	ACE_I_or_ARB_At_Hospital_Dischar(ACE-I or ARB-At Hospital Discharge)		
STEMI_NSTEMI(STEMI/NSTEMI)	1	2	Total
1	951<br>36.23<br>74.53<br>47.86	325<br>12.38<br>25.47<br>50.94	1276<br>48.61
2	1036<br>39.47<br>76.80<br>52.14	313<br>11.92<br>23.20<br>49.06	1349<br>51.39
Total	1987<br>75.70	638<br>24.30	2625<br>100.00
Frequency Missing = 66			

Statistics for Table of STEMI_NSTEMI by ACE_I_or_ARB_At_Hospital_Dischar

Statistic	DF	Value	Prob
Chi-Square	1	1.8332	0.1758
Likelihood Ratio Chi-Square	1	1.8326	0.1758
Continuity Adj. Chi-Square	1	1.7120	0.1907
Mantel-Haenszel Chi-Square	1	1.8325	0.1758
Phi Coefficient		-0.0264	
Contingency Coefficient		0.0264	
Cramer's V		-0.0264	

Fisher's Exact Test	
Cell (1,1) Frequency (F)	951
Left-sided Pr <= F	0.0954
Right-sided Pr >= F	0.9191
Table Probability (P)	0.0145
Two-sided Pr <= P	0.1868

Sample Size = 2625
Frequency Missing = 66

The FREQ Procedure

Frequency<br>Percent<br>Row Pct<br>Col Pct

Table of STEMI_NSTEMI by Aldosterone_Inhibitor__Spironol1			
	Aldosterone_Inhibitor__Spironol1(Aldosterone Inhibitor (Spironolactone)-At Hospital Discharge)		
**STEMI_NSTEMI(STEMI/NSTEMI)**	**1**	**2**	**Total**
**1**	214<br>8.15<br>16.77<br>52.45	1062<br>40.46<br>83.23<br>47.90	1276<br>48.61
**2**	194<br>7.39<br>14.38<br>47.55	1155<br>44.00<br>85.62<br>52.10	1349<br>51.39
**Total**	408<br>15.54	2217<br>84.46	2625<br>100.00
**Frequency Missing = 66**			

Statistics for Table of STEMI_NSTEMI by Aldosterone_Inhibitor__Spironol1

Statistic	DF	Value	Prob
Chi-Square	1	2.8537	0.0912
Likelihood Ratio Chi-Square	1	2.8528	0.0912
Continuity Adj. Chi-Square	1	2.6745	0.1020
Mantel-Haenszel Chi-Square	1	2.8526	0.0912
Phi Coefficient		0.0330	
Contingency Coefficient		0.0330	
Cramer's V		0.0330	

Fisher's Exact Test	
Cell (1,1) Frequency (F)	214
Left-sided Pr <= F	0.9593
Right-sided Pr >= F	0.0510
Table Probability (P)	0.0103
Two-sided Pr <= P	0.0949

Sample Size = 2625
Frequency Missing = 66

The FREQ Procedure

Frequency<br>Percent<br>Row Pct<br>Col Pct

Table of STEMI_NSTEMI by Aspirin_At_Hospital_Discharge			
	Aspirin_At_Hospital_Discharge(Aspirin-At Hospital Discharge)		
STEMI_NSTEMI(STEMI/NSTEMI)	1	2	Total
1	1241<br>47.28<br>97.26<br>48.88	35<br>1.33<br>2.74<br>40.70	1276<br>48.61
2	1298<br>49.45<br>96.22<br>51.12	51<br>1.94<br>3.78<br>59.30	1349<br>51.39
Total	2539<br>96.72	86<br>3.28	2625<br>100.00
Frequency Missing = 66			

Statistics for Table of STEMI_NSTEMI by Aspirin_At_Hospital_Discharge

Statistic	DF	Value	Prob
Chi-Square	1	2.2280	0.1355
Likelihood Ratio Chi-Square	1	2.2435	0.1342
Continuity Adj. Chi-Square	1	1.9126	0.1667
Mantel-Haenszel Chi-Square	1	2.2272	0.1356
Phi Coefficient		0.0291	
Contingency Coefficient		0.0291	
Cramer's V		0.0291	

Fisher's Exact Test	
Cell (1,1) Frequency (F)	1241
Left-sided Pr <= F	0.9459
Right-sided Pr >= F	0.0830
Table Probability (P)	0.0289
Two-sided Pr <= P	0.1539

Sample Size = 2625
Frequency Missing = 66

The FREQ Procedure

Frequency<br>Percent<br>Row Pct<br>Col Pct

Table of STEMI_NSTEMI by Beta_Blockers_At_Hospital_Discha

STEMI_NSTEMI(STEMI/NSTEMI)	Beta_Blockers_At_Hospital_Discha(Beta-Blockers-At Hospital Discharge) 1	2	Total
1	1005<br>38.29<br>78.76<br>45.97	271<br>10.32<br>21.24<br>61.73	1276<br>48.61
2	1181<br>44.99<br>87.55<br>54.03	168<br>6.40<br>12.45<br>38.27	1349<br>51.39
Total	2186<br>83.28	439<br>16.72	2625<br>100.00
Frequency Missing = 66			

Statistics for Table of STEMI_NSTEMI by Beta_Blockers_At_Hospital_Discha

Statistic	DF	Value	Prob
Chi-Square	1	36.3345	<.0001
Likelihood Ratio Chi-Square	1	36.5482	<.0001
Continuity Adj. Chi-Square	1	35.7064	<.0001
Mantel-Haenszel Chi-Square	1	36.3206	<.0001
Phi Coefficient		-0.1177	
Contingency Coefficient		0.1168	
Cramer's V		-0.1177	

Fisher's Exact Test	
Cell (1,1) Frequency (F)	1005
Left-sided Pr <= F	<.0001
Right-sided Pr >= F	1.0000
Table Probability (P)	<.0001
Two-sided Pr <= P	<.0001

Sample Size = 2625
Frequency Missing = 66

The FREQ Procedure

Frequency<br>Percent<br>Row Pct<br>Col Pct

Table of STEMI_NSTEMI by Clopidogrel__At_Hospital_Dischar			
	Clopidogrel__At_Hospital_Dischar(Clopidogrel -At Hospital Discharge)		
STEMI_NSTEMI(STEMI/NSTEMI)	1	2	Total
1	843<br>32.11<br>66.07<br>47.90	433<br>16.50<br>33.93<br>50.06	1276<br>48.61
2	917<br>34.93<br>67.98<br>52.10	432<br>16.46<br>32.02<br>49.94	1349<br>51.39
Total	1760<br>67.05	865<br>32.95	2625<br>100.00
Frequency Missing = 66			

Statistics for Table of STEMI_NSTEMI by Clopidogrel__At_Hospital_Dischar

Statistic	DF	Value	Prob
Chi-Square	1	1.0833	0.2980
Likelihood Ratio Chi-Square	1	1.0831	0.2980
Continuity Adj. Chi-Square	1	0.9985	0.3177
Mantel-Haenszel Chi-Square	1	1.0828	0.2981
Phi Coefficient		-0.0203	
Contingency Coefficient		0.0203	
Cramer's V		-0.0203	

Fisher's Exact Test	
Cell (1,1) Frequency (F)	843
Left-sided Pr <= F	0.1588
Right-sided Pr >= F	0.8604
Table Probability (P)	0.0193
Two-sided Pr <= P	0.2994

Sample Size = 2625
Frequency Missing = 66

The FREQ Procedure

Frequency<br>Percent<br>Row Pct<br>Col Pct

Table of STEMI_NSTEMI by GLP_1_agonists_At_Hospital_Dicha			
	GLP_1_agonists_At_Hospital_Dicha(GLP-1 agonists-At Hospital Dicharge)		
STEMI_NSTEMI(STEMI/NSTEMI)	1	2	Total
1	2<br>0.08<br>0.16<br>13.33	1274<br>48.53<br>99.84<br>48.81	1276<br>48.61
2	13<br>0.50<br>0.96<br>86.67	1336<br>50.90<br>99.04<br>51.19	1349<br>51.39
Total	15<br>0.57	2610<br>99.43	2625<br>100.00
Frequency Missing = 66			

Statistics for Table of STEMI_NSTEMI by GLP_1_agonists_At_Hospital_Dicha

Statistic	DF	Value	Prob
Chi-Square	1	7.5152	0.0061
Likelihood Ratio Chi-Square	1	8.4568	0.0036
Continuity Adj. Chi-Square	1	6.1620	0.0131
Mantel-Haenszel Chi-Square	1	7.5123	0.0061
Phi Coefficient		-0.0535	
Contingency Coefficient		0.0534	
Cramer's V		-0.0535	

Fisher's Exact Test	
Cell (1,1) Frequency (F)	2
Left-sided Pr <= F	0.0049
Right-sided Pr >= F	0.9993
Table Probability (P)	0.0042
Two-sided Pr <= P	0.0075

Sample Size = 2625
Frequency Missing = 66

The FREQ Procedure

Frequency<br>Percent<br>Row Pct<br>Col Pct

Table of STEMI_NSTEMI by Insulin_At_Hospital_Dicharge			
	Insulin_At_Hospital_Dicharge(Insulin-At Hospital Dicharge)		
STEMI_NSTEMI(STEMI/NSTEMI)	1	2	Total
1	341<br>12.98<br>26.68<br>42.36	937<br>35.67<br>73.32<br>51.43	1278<br>48.65
2	464<br>17.66<br>34.40<br>57.64	885<br>33.69<br>65.60<br>48.57	1349<br>51.35
Total	805<br>30.64	1822<br>69.36	2627<br>100.00
Frequency Missing = 64			

Statistics for Table of STEMI_NSTEMI by Insulin_At_Hospital_Dicharge

Statistic	DF	Value	Prob
Chi-Square	1	18.3724	<.0001
Likelihood Ratio Chi-Square	1	18.4327	<.0001
Continuity Adj. Chi-Square	1	18.0112	<.0001
Mantel-Haenszel Chi-Square	1	18.3654	<.0001
Phi Coefficient		-0.0836	
Contingency Coefficient		0.0833	
Cramer's V		-0.0836	

Fisher's Exact Test	
Cell (1,1) Frequency (F)	341
Left-sided Pr <= F	<.0001
Right-sided Pr >= F	1.0000
Table Probability (P)	<.0001
Two-sided Pr <= P	<.0001

Sample Size = 2627
Frequency Missing = 64

The FREQ Procedure

Frequency<br>Percent<br>Row Pct<br>Col Pct

Table of STEMI_NSTEMI by Novel_oral_anticoagulants__NOAC1			
	Novel_oral_anticoagulants__NOAC1(Novel oral anticoagulants (NOACs)-At Hospital Dicharge)		
STEMI_NSTEMI(STEMI/NSTEMI)	1	2	Total
1	20<br>0.76<br>1.57<br>41.67	1256<br>47.85<br>98.43<br>48.74	1276<br>48.61
2	28<br>1.07<br>2.08<br>58.33	1321<br>50.32<br>97.92<br>51.26	1349<br>51.39
Total	48<br>1.83	2577<br>98.17	2625<br>100.00
Frequency Missing = 66			

Statistics for Table of STEMI_NSTEMI by Novel_oral_anticoagulants__NOAC1

Statistic	DF	Value	Prob
Chi-Square	1	0.9435	0.3314
Likelihood Ratio Chi-Square	1	0.9489	0.3300
Continuity Adj. Chi-Square	1	0.6816	0.4090
Mantel-Haenszel Chi-Square	1	0.9431	0.3315
Phi Coefficient		-0.0190	
Contingency Coefficient		0.0190	
Cramer's V		-0.0190	

Fisher's Exact Test	
Cell (1,1) Frequency (F)	20
Left-sided Pr <= F	0.2048
Right-sided Pr >= F	0.8682
Table Probability (P)	0.0729
Two-sided Pr <= P	0.3827

Sample Size = 2625
Frequency Missing = 66

The FREQ Procedure

Frequency<br>Percent<br>Row Pct<br>Col Pct

Table of STEMI_NSTEMI by Oral_hypoglycemic_agents_At_Hosp			
	Oral_hypoglycemic_agents_At_Hosp(Oral hypoglycemic agents-At Hospital Dicharge)		
STEMI_NSTEMI(STEMI/NSTEMI)	1	2	Total
1	312<br>11.89<br>24.45<br>43.15	964<br>36.72<br>75.55<br>50.68	1276<br>48.61
2	411<br>15.66<br>30.47<br>56.85	938<br>35.73<br>69.53<br>49.32	1349<br>51.39
Total	723<br>27.54	1902<br>72.46	2625<br>100.00
Frequency Missing = 66			

Statistics for Table of STEMI_NSTEMI by Oral_hypoglycemic_agents_At_Hosp

Statistic	DF	Value	Prob
Chi-Square	1	11.8905	0.0006
Likelihood Ratio Chi-Square	1	11.9238	0.0006
Continuity Adj. Chi-Square	1	11.5910	0.0007
Mantel-Haenszel Chi-Square	1	11.8860	0.0006
Phi Coefficient		-0.0673	
Contingency Coefficient		0.0672	
Cramer's V		-0.0673	

Fisher's Exact Test	
Cell (1,1) Frequency (F)	312
Left-sided Pr <= F	0.0003
Right-sided Pr >= F	0.9998
Table Probability (P)	<.0001
Two-sided Pr <= P	0.0006

Sample Size = 2625
Frequency Missing = 66

The FREQ Procedure

Frequency<br>Percent<br>Row Pct<br>Col Pct

Table of STEMI_NSTEMI by PCSk9_inhibitors_At_Hospital_Dic			
	PCSk9_inhibitors_At_Hospital_Dic(PCSk9 inhibitors-At Hospital Dicharge)		
STEMI_NSTEMI(STEMI/NSTEMI)	1	2	Total
1	48<br>1.83<br>3.76<br>42.11	1228<br>46.78<br>96.24<br>48.90	1276<br>48.61
2	66<br>2.51<br>4.89<br>57.89	1283<br>48.88<br>95.11<br>51.10	1349<br>51.39
Total	114<br>4.34	2511<br>95.66	2625<br>100.00
Frequency Missing = 66			

Statistics for Table of STEMI_NSTEMI by PCSk9_inhibitors_At_Hospital_Dic

Statistic	DF	Value	Prob
Chi-Square	1	2.0183	0.1554
Likelihood Ratio Chi-Square	1	2.0285	0.1544
Continuity Adj. Chi-Square	1	1.7553	0.1852
Mantel-Haenszel Chi-Square	1	2.0175	0.1555
Phi Coefficient		-0.0277	
Contingency Coefficient		0.0277	
Cramer's V		-0.0277	

Fisher's Exact Test	
Cell (1,1) Frequency (F)	48
Left-sided Pr <= F	0.0924
Right-sided Pr >= F	0.9356
Table Probability (P)	0.0280
Two-sided Pr <= P	0.1797

Sample Size = 2625
Frequency Missing = 66

The FREQ Procedure

Frequency<br>Percent<br>Row Pct<br>Col Pct

Table of STEMI_NSTEMI by Prasugrel__At_Hospital_Discharge			
	Prasugrel__At_Hospital_Discharge(Prasugrel -At Hospital Discharge)		
STEMI_NSTEMI(STEMI/NSTEMI)	1	2	Total
1	3<br>0.11<br>0.24<br>30.00	1273<br>48.50<br>99.76<br>48.68	1276<br>48.61
2	7<br>0.27<br>0.52<br>70.00	1342<br>51.12<br>99.48<br>51.32	1349<br>51.39
Total	10<br>0.38	2615<br>99.62	2625<br>100.00
Frequency Missing = 66			

Statistics for Table of STEMI_NSTEMI by Prasugrel__At_Hospital_Discharge

Statistic	DF	Value	Prob
Chi-Square	1	1.3916	0.2381
Likelihood Ratio Chi-Square	1	1.4362	0.2308
Continuity Adj. Chi-Square	1	0.7443	0.3883
Mantel-Haenszel Chi-Square	1	1.3911	0.2382
Phi Coefficient		-0.0230	
Contingency Coefficient		0.0230	
Cramer's V		-0.0230	
WARNING: 25% of the cells have expected counts less than 5. Chi-Square may not be a valid test.			

Fisher's Exact Test	
Cell (1,1) Frequency (F)	3
Left-sided Pr <= F	0.1952
Right-sided Pr >= F	0.9352
Table Probability (P)	0.1304
Two-sided Pr <= P	0.3447

Sample Size = 2625
Frequency Missing = 66

The FREQ Procedure

Frequency<br>Percent<br>Row Pct<br>Col Pct

Table of STEMI_NSTEMI by Selective_SGLT_2_inhibitors_At_H

	Selective_SGLT_2_inhibitors_At_H(Selective SGLT-2 inhibitors-At Hospital Dicharge)		
STEMI_NSTEMI(STEMI/NSTEMI)	1	2	Total
1	183<br>6.97<br>14.34<br>44.31	1093<br>41.64<br>85.66<br>49.41	1276<br>48.61
2	230<br>8.76<br>17.05<br>55.69	1119<br>42.63<br>82.95<br>50.59	1349<br>51.39
Total	413<br>15.73	2212<br>84.27	2625<br>100.00

Frequency Missing = 66

Statistics for Table of STEMI_NSTEMI by Selective_SGLT_2_inhibitors_At_H

Statistic	DF	Value	Prob
Chi-Square	1	3.6270	0.0568
Likelihood Ratio Chi-Square	1	3.6355	0.0566
Continuity Adj. Chi-Square	1	3.4256	0.0642
Mantel-Haenszel Chi-Square	1	3.6256	0.0569
Phi Coefficient		-0.0372	
Contingency Coefficient		0.0371	
Cramer's V		-0.0372	

Fisher's Exact Test	
Cell (1,1) Frequency (F)	183
Left-sided Pr <= F	0.0320
Right-sided Pr >= F	0.9750
Table Probability (P)	0.0070
Two-sided Pr <= P	0.0605

Sample Size = 2625
Frequency Missing = 66

The FREQ Procedure

Frequency<br>Percent<br>Row Pct<br>Col Pct

Table of STEMI_NSTEMI by Ticagrel_At_Hospital_Discharge			
	Ticagrel_At_Hospital_Discharge(Ticagrel-At Hospital Discharge)		
STEMI_NSTEMI(STEMI/NSTEMI)	1	2	Total
1	412<br>15.70<br>32.29<br>54.79	864<br>32.91<br>67.71<br>46.13	1276<br>48.61
2	340<br>12.95<br>25.20<br>45.21	1009<br>38.44<br>74.80<br>53.87	1349<br>51.39
Total	752<br>28.65	1873<br>71.35	2625<br>100.00
Frequency Missing = 66			

Statistics for Table of STEMI_NSTEMI by Ticagrel_At_Hospital_Discharge

Statistic	DF	Value	Prob
Chi-Square	1	16.1013	<.0001
Likelihood Ratio Chi-Square	1	16.1104	<.0001
Continuity Adj. Chi-Square	1	15.7566	<.0001
Mantel-Haenszel Chi-Square	1	16.0951	<.0001
Phi Coefficient		0.0783	
Contingency Coefficient		0.0781	
Cramer's V		0.0783	

Fisher's Exact Test	
Cell (1,1) Frequency (F)	412
Left-sided Pr <= F	1.0000
Right-sided Pr >= F	<.0001
Table Probability (P)	<.0001
Two-sided Pr <= P	<.0001

Sample Size = 2625
Frequency Missing = 66

The FREQ Procedure

Frequency<br>Percent<br>Row Pct<br>Col Pct

Table of STEMI_NSTEMI by VAR148			
	VAR148(Selective SGLT-2 inhibitors--24hrs of Hospital Admission)		
STEMI_NSTEMI(STEMI/NSTEMI)	1	2	Total
1	84<br>3.12<br>6.40<br>38.18	1229<br>45.69<br>93.60<br>49.76	1313<br>48.81
2	136<br>5.06<br>9.88<br>61.82	1241<br>46.13<br>90.12<br>50.24	1377<br>51.19
Total	220<br>8.18	2470<br>91.82	2690<br>100.00
Frequency Missing = 1			

Statistics for Table of STEMI_NSTEMI by VAR148

Statistic	DF	Value	Prob
Chi-Square	1	10.8327	0.0010
Likelihood Ratio Chi-Square	1	10.9435	0.0009
Continuity Adj. Chi-Square	1	10.3743	0.0013
Mantel-Haenszel Chi-Square	1	10.8286	0.0010
Phi Coefficient		-0.0635	
Contingency Coefficient		0.0633	
Cramer's V		-0.0635	

Fisher's Exact Test	
Cell (1,1) Frequency (F)	84
Left-sided Pr <= F	0.0006
Right-sided Pr >= F	0.9996
Table Probability (P)	0.0002
Two-sided Pr <= P	0.0012

Sample Size = 2690
Frequency Missing = 1

The FREQ Procedure

Frequency<br>Percent<br>Row Pct<br>Col Pct

Table of STEMI_NSTEMI by VAR207			
	VAR207(Icosapent ethyl (e.g. VASCEPA®)-At Hospital Dicharge)		
STEMI_NSTEMI(STEMI/NSTEMI)	1	2	Total
1	2<br>0.08<br>0.16<br>100.00	1274<br>48.53<br>99.84<br>48.57	1276<br>48.61
2	0<br>0.00<br>0.00<br>0.00	1349<br>51.39<br>100.00<br>51.43	1349<br>51.39
Total	2<br>0.08	2623<br>99.92	2625<br>100.00
Frequency Missing = 66			

Statistics for Table of STEMI_NSTEMI by VAR207

Statistic	DF	Value	Prob
Chi-Square	1	2.1160	0.1458
Likelihood Ratio Chi-Square	1	2.8870	0.0893
Continuity Adj. Chi-Square	1	0.5580	0.4551
Mantel-Haenszel Chi-Square	1	2.1152	0.1458
Phi Coefficient		0.0284	
Contingency Coefficient		0.0284	
Cramer's V		0.0284	
WARNING: 50% of the cells have expected counts less than 5. Chi-Square may not be a valid test.			

Fisher's Exact Test	
Cell (1,1) Frequency (F)	2
Left-sided Pr <= F	1.0000
Right-sided Pr >= F	0.2362
Table Probability (P)	0.2362
Two-sided Pr <= P	0.2362

Sample Size = 2625
Frequency Missing = 66

The FREQ Procedure

Frequency<br>Percent<br>Row Pct<br>Col Pct

Table of STEMI_NSTEMI by VAR208			
	VAR208(Omega-3-acid ethyl esters 90 (e.g.Omacor®)-At Hospital Dicharge)		
STEMI_NSTEMI(STEMI/NSTEMI)	1	2	Total
1	6<br>0.23<br>0.47<br>42.86	1270<br>48.38<br>99.53<br>48.64	1276<br>48.61
2	8<br>0.30<br>0.59<br>57.14	1341<br>51.09<br>99.41<br>51.36	1349<br>51.39
Total	14<br>0.53	2611<br>99.47	2625<br>100.00
Frequency Missing = 66			

Statistics for Table of STEMI_NSTEMI by VAR208

Statistic	DF	Value	Prob
Chi-Square	1	0.1864	0.6659
Likelihood Ratio Chi-Square	1	0.1873	0.6652
Continuity Adj. Chi-Square	1	0.0268	0.8700
Mantel-Haenszel Chi-Square	1	0.1864	0.6660
Phi Coefficient		-0.0084	
Contingency Coefficient		0.0084	
Cramer's V		-0.0084	

Fisher's Exact Test	
Cell (1,1) Frequency (F)	6
Left-sided Pr <= F	0.4364
Right-sided Pr >= F	0.7568
Table Probability (P)	0.1931
Two-sided Pr <= P	0.7911

Sample Size = 2625
Frequency Missing = 66

The FREQ Procedure

Frequency<br>Percent<br>Row Pct<br>Col Pct

Table of STEMI_NSTEMI by VAR209

STEMI_NSTEMI(STEMI/NSTEMI)	VAR209(Gemfibrozil (e.g. Lopid®)-At Hospital Dicharge)	
	2	Total
1	1276<br>48.61<br>100.00<br>48.61	1276<br>48.61
2	1349<br>51.39<br>100.00<br>51.39	1349<br>51.39
Total	2625<br>100.00	2625<br>100.00

Frequency Missing = 66

Frequency<br>Percent<br>Row Pct<br>Col Pct

Table of STEMI_NSTEMI by VAR210

STEMI_NSTEMI(STEMI/NSTEMI)	VAR210(Fenofibrate (e.g. Lipanthyl®)-At Hospital Dicharge)		
	1	2	Total
1	16<br>0.61<br>1.25<br>41.03	1260<br>48.00<br>98.75<br>48.72	1276<br>48.61
2	23<br>0.88<br>1.70<br>58.97	1326<br>50.51<br>98.30<br>51.28	1349<br>51.39
Total	39<br>1.49	2586<br>98.51	2625<br>100.00

Frequency Missing = 66

The FREQ Procedure

Statistics for Table of STEMI_NSTEMI by VAR210

Statistic	DF	Value	Prob
Chi-Square	1	0.9115	0.3397
Likelihood Ratio Chi-Square	1	0.9175	0.3381
Continuity Adj. Chi-Square	1	0.6294	0.4276
Mantel-Haenszel Chi-Square	1	0.9111	0.3398
Phi Coefficient		-0.0186	
Contingency Coefficient		0.0186	
Cramer's V		-0.0186	

Fisher's Exact Test	
Cell (1,1) Frequency (F)	16
Left-sided Pr <= F	0.2142
Right-sided Pr >= F	0.8680
Table Probability (P)	0.0821
Two-sided Pr <= P	0.4201

Sample Size = 2625
Frequency Missing = 66

Frequency<br>Percent<br>Row Pct<br>Col Pct

Table of STEMI_NSTEMI by Statins_At_Hospital_Discharge			
	Statins_At_Hospital_Discharge(Statins-At Hospital Discharge)		
STEMI_NSTEMI(STEMI/NSTEMI)	1	2	Total
1	1203<br>45.83<br>94.28<br>48.51	73<br>2.78<br>5.72<br>50.34	1276<br>48.61
2	1277<br>48.65<br>94.66<br>51.49	72<br>2.74<br>5.34<br>49.66	1349<br>51.39
Total	2480<br>94.48	145<br>5.52	2625<br>100.00
Frequency Missing = 66			

The FREQ Procedure

Statistics for Table of STEMI_NSTEMI by Statins_At_Hospital_Discharge

Statistic	DF	Value	Prob
Chi-Square	1	0.1850	0.6671
Likelihood Ratio Chi-Square	1	0.1849	0.6672
Continuity Adj. Chi-Square	1	0.1188	0.7304
Mantel-Haenszel Chi-Square	1	0.1849	0.6672
Phi Coefficient		-0.0084	
Contingency Coefficient		0.0084	
Cramer's V		-0.0084	

Fisher's Exact Test	
Cell (1,1) Frequency (F)	1203
Left-sided Pr <= F	0.3650
Right-sided Pr >= F	0.6970
Table Probability (P)	0.0620
Two-sided Pr <= P	0.6703

Sample Size = 2625
Frequency Missing = 66

*in-hospital outcomes;*1: >50, 2: 40-50%, 3: 30-40%, 4:<30%;

The FREQ Procedure

Frequency
Percent
Row Pct
Col Pct

Table of STEMI_NSTEMI by Echo_Options					
	Echo_Options(Echo-Options)				
STEMI_NSTEMI(STEMI/NSTEMI)	1	2	3	4	Total
1	337<br>14.13<br>30.53<br>35.59	393<br>16.48<br>35.60<br>51.11	261<br>10.94<br>23.64<br>56.74	113<br>4.74<br>10.24<br>54.07	1104<br>46.29
2	610<br>25.58<br>47.62<br>64.41	376<br>15.77<br>29.35<br>48.89	199<br>8.34<br>15.53<br>43.26	96<br>4.03<br>7.49<br>45.93	1281<br>53.71
Total	947<br>39.71	769<br>32.24	460<br>19.29	209<br>8.76	2385<br>100.00
Frequency Missing = 306					

Statistics for Table of STEMI_NSTEMI by Echo_Options

Statistic	DF	Value	Prob
Chi-Square	3	76.0985	<.0001
Likelihood Ratio Chi-Square	3	76.8223	<.0001
Mantel-Haenszel Chi-Square	1	59.3866	<.0001
Phi Coefficient		0.1786	
Contingency Coefficient		0.1758	
Cramer's V		0.1786	

Sample Size = 2385
Frequency Missing = 306

WARNING: 11% of the data are missing.

Frequency
Percent
Row Pct
Col Pct

Table of STEMI_NSTEMI by Elective_coronary_angiogram			
	Elective_coronary_angiogram(Elective coronary angiogram)		
STEMI_NSTEMI(STEMI/NSTEMI)	1	2	Total
1	225<br>12.93<br>27.47<br>54.09	594<br>34.14<br>72.53<br>44.86	819<br>47.07
2	191<br>10.98<br>20.74<br>45.91	730<br>41.95<br>79.26<br>55.14	921<br>52.93
Total	416<br>23.91	1324<br>76.09	1740<br>100.00
Frequency Missing = 951			

*in-hospital outcomes;*1: >50, 2: 40-50%, 3: 30-40%, 4:<30%;

The FREQ Procedure

Statistics for Table of STEMI_NSTEMI by Elective_coronary_angiogram

Statistic	DF	Value	Prob
Chi-Square	1	10.8065	0.0010
Likelihood Ratio Chi-Square	1	10.7937	0.0010
Continuity Adj. Chi-Square	1	10.4395	0.0012
Mantel-Haenszel Chi-Square	1	10.8002	0.0010
Phi Coefficient		0.0788	
Contingency Coefficient		0.0786	
Cramer's V		0.0788	

Fisher's Exact Test	
Cell (1,1) Frequency (F)	225
Left-sided Pr <= F	0.9996
Right-sided Pr >= F	0.0006
Table Probability (P)	0.0002
Two-sided Pr <= P	0.0011

Sample Size = 1740
Frequency Missing = 951

WARNING: 35% of the data are missing.

Frequency<br>Percent<br>Row Pct<br>Col Pct

Table of STEMI_NSTEMI by Recurrent_MI			
	Recurrent_MI(Recurrent MI)		
STEMI_NSTEMI(STEMI/NSTEMI)	1	2	Total
1	34<br>1.26<br>2.59<br>42.50	1279<br>47.55<br>97.41<br>49.00	1313<br>48.81
2	46<br>1.71<br>3.34<br>57.50	1331<br>49.48<br>96.66<br>51.00	1377<br>51.19
Total	80<br>2.97	2610<br>97.03	2690<br>100.00
Frequency Missing = 1			

*in-hospital outcomes;*1: >50, 2: 40-50%, 3: 30-40%, 4:<30%;

The FREQ Procedure

Statistics for Table of STEMI_NSTEMI by Recurrent_MI

Statistic	DF	Value	Prob
Chi-Square	1	1.3141	0.2517
Likelihood Ratio Chi-Square	1	1.3201	0.2506
Continuity Adj. Chi-Square	1	1.0667	0.3017
Mantel-Haenszel Chi-Square	1	1.3136	0.2517
Phi Coefficient		-0.0221	
Contingency Coefficient		0.0221	
Cramer's V		-0.0221	

Fisher's Exact Test	
Cell (1,1) Frequency (F)	34
Left-sided Pr <= F	0.1508
Right-sided Pr >= F	0.8964
Table Probability (P)	0.0472
Two-sided Pr <= P	0.2587

Sample Size = 2690
Frequency Missing = 1

Frequency<br>Percent<br>Row Pct<br>Col Pct

Table of STEMI_NSTEMI by Recurrent_ischemia			
	Recurrent_ischemia(Recurrent ischemia)		
STEMI_NSTEMI(STEMI/NSTEMI)	1	2	Total
1	136<br>5.06<br>10.36<br>37.47	1177<br>43.75<br>89.64<br>50.58	1313<br>48.81
2	227<br>8.44<br>16.49<br>62.53	1150<br>42.75<br>83.51<br>49.42	1377<br>51.19
Total	363<br>13.49	2327<br>86.51	2690<br>100.00
Frequency Missing = 1			

*in-hospital outcomes;*1: >50, 2: 40-50%, 3: 30-40%, 4:<30%;

The FREQ Procedure

Statistics for Table of STEMI_NSTEMI by Recurrent_ischemia

Statistic	DF	Value	Prob
Chi-Square	1	21.6155	<.0001
Likelihood Ratio Chi-Square	1	21.8483	<.0001
Continuity Adj. Chi-Square	1	21.0938	<.0001
Mantel-Haenszel Chi-Square	1	21.6075	<.0001
Phi Coefficient		-0.0896	
Contingency Coefficient		0.0893	
Cramer's V		-0.0896	

Fisher's Exact Test	
Cell (1,1) Frequency (F)	136
Left-sided Pr <= F	<.0001
Right-sided Pr >= F	1.0000
Table Probability (P)	<.0001
Two-sided Pr <= P	<.0001

Sample Size = 2690
Frequency Missing = 1

Frequency<br>Percent<br>Row Pct<br>Col Pct

Table of STEMI_NSTEMI by Stroke			
	Stroke(Stroke)		
STEMI_NSTEMI(STEMI/NSTEMI)	1	2	Total
1	14<br>0.52<br>1.07<br>40.00	1299<br>48.29<br>98.93<br>48.93	1313<br>48.81
2	21<br>0.78<br>1.53<br>60.00	1356<br>50.41<br>98.47<br>51.07	1377<br>51.19
Total	35<br>1.30	2655<br>98.70	2690<br>100.00
Frequency Missing = 1			

*in-hospital outcomes;*1: >50, 2: 40-50%, 3: 30-40%, 4:<30%;

The FREQ Procedure

Statistics for Table of STEMI_NSTEMI by Stroke

Statistic	DF	Value	Prob
Chi-Square	1	1.1017	0.2939
Likelihood Ratio Chi-Square	1	1.1105	0.2920
Continuity Adj. Chi-Square	1	0.7734	0.3792
Mantel-Haenszel Chi-Square	1	1.1013	0.2940
Phi Coefficient		-0.0202	
Contingency Coefficient		0.0202	
Cramer's V		-0.0202	

Fisher's Exact Test	
Cell (1,1) Frequency (F)	14
Left-sided Pr <= F	0.1898
Right-sided Pr >= F	0.8891
Table Probability (P)	0.0789
Two-sided Pr <= P	0.3122

Sample Size = 2690
Frequency Missing = 1

Frequency<br>Percent<br>Row Pct<br>Col Pct

Table of STEMI_NSTEMI by Cardiogenic_Shock			
	Cardiogenic_Shock(Cardiogenic Shock)		
STEMI_NSTEMI(STEMI/NSTEMI)	1	2	Total
1	74<br>2.75<br>5.64<br>72.55	1239<br>46.06<br>94.36<br>47.87	1313<br>48.81
2	28<br>1.04<br>2.03<br>27.45	1349<br>50.15<br>97.97<br>52.13	1377<br>51.19
Total	102<br>3.79	2588<br>96.21	2690<br>100.00
Frequency Missing = 1			

*in-hospital outcomes;*1: >50, 2: 40-50%, 3: 30-40%, 4:<30%;

The FREQ Procedure

Statistics for Table of STEMI_NSTEMI by Cardiogenic_Shock

Statistic	DF	Value	Prob
Chi-Square	1	23.9114	<.0001
Likelihood Ratio Chi-Square	1	24.6667	<.0001
Continuity Adj. Chi-Square	1	22.9341	<.0001
Mantel-Haenszel Chi-Square	1	23.9025	<.0001
Phi Coefficient		0.0943	
Contingency Coefficient		0.0939	
Cramer's V		0.0943	

Fisher's Exact Test	
Cell (1,1) Frequency (F)	74
Left-sided Pr <= F	1.0000
Right-sided Pr >= F	<.0001
Table Probability (P)	<.0001
Two-sided Pr <= P	<.0001

Sample Size = 2690
Frequency Missing = 1

Frequency<br>Percent<br>Row Pct<br>Col Pct

Table of STEMI_NSTEMI by Heart_Failure			
	Heart_Failure(Heart Failure)		
STEMI_NSTEMI(STEMI/NSTEMI)	1	2	Total
1	140<br>5.20<br>10.66<br>43.48	1173<br>43.61<br>89.34<br>49.54	1313<br>48.81
2	182<br>6.77<br>13.22<br>56.52	1195<br>44.42<br>86.78<br>50.46	1377<br>51.19
Total	322<br>11.97	2368<br>88.03	2690<br>100.00
Frequency Missing = 1			

*in-hospital outcomes;*1: >50, 2: 40-50%, 3: 30-40%, 4:<30%;

The FREQ Procedure

Statistics for Table of STEMI_NSTEMI by Heart_Failure

Statistic	DF	Value	Prob
Chi-Square	1	4.1623	0.0413
Likelihood Ratio Chi-Square	1	4.1755	0.0410
Continuity Adj. Chi-Square	1	3.9234	0.0476
Mantel-Haenszel Chi-Square	1	4.1608	0.0414
Phi Coefficient		-0.0393	
Contingency Coefficient		0.0393	
Cramer's V		-0.0393	

Fisher's Exact Test	
Cell (1,1) Frequency (F)	140
Left-sided Pr <= F	0.0237
Right-sided Pr >= F	0.9822
Table Probability (P)	0.0059
Two-sided Pr <= P	0.0434

Sample Size = 2690
Frequency Missing = 1

Frequency<br>Percent<br>Row Pct<br>Col Pct

Table of STEMI_NSTEMI by Major_bleeding			
	Major_bleeding(Major bleeding)		
STEMI_NSTEMI(STEMI/NSTEMI)	1	2	Total
1	13<br>0.48<br>0.99<br>68.42	1300<br>48.33<br>99.01<br>48.67	1313<br>48.81
2	6<br>0.22<br>0.44<br>31.58	1371<br>50.97<br>99.56<br>51.33	1377<br>51.19
Total	19<br>0.71	2671<br>99.29	2690<br>100.00
Frequency Missing = 1			

*in-hospital outcomes;*1: >50, 2: 40-50%, 3: 30-40%, 4:<30%;

The FREQ Procedure

Statistics for Table of STEMI_NSTEMI by Major_bleeding

Statistic	DF	Value	Prob
Chi-Square	1	2.9452	0.0861
Likelihood Ratio Chi-Square	1	3.0054	0.0830
Continuity Adj. Chi-Square	1	2.2078	0.1373
Mantel-Haenszel Chi-Square	1	2.9442	0.0862
Phi Coefficient		0.0331	
Contingency Coefficient		0.0331	
Cramer's V		0.0331	

Fisher's Exact Test	
Cell (1,1) Frequency (F)	13
Left-sided Pr <= F	0.9753
Right-sided Pr >= F	0.0679
Table Probability (P)	0.0433
Two-sided Pr <= P	0.1073

Sample Size = 2690
Frequency Missing = 1

Frequency
Percent
Row Pct
Col Pct

Table of STEMI_NSTEMI by VT_VF_arrest			
	VT_VF_arrest(VT/VF arrest)		
STEMI_NSTEMI(STEMI/NSTEMI)	1	2	Total
1	77<br>2.86<br>5.86<br>74.76	1236<br>45.95<br>94.14<br>47.78	1313<br>48.81
2	26<br>0.97<br>1.89<br>25.24	1351<br>50.22<br>98.11<br>52.22	1377<br>51.19
Total	103<br>3.83	2587<br>96.17	2690<br>100.00
Frequency Missing = 1			

*in-hospital outcomes;*1: >50, 2: 40-50%, 3: 30-40%, 4:<30%;

The FREQ Procedure

Statistics for Table of STEMI_NSTEMI by VT_VF_arrest

Statistic	DF	Value	Prob
Chi-Square	1	28.8582	<.0001
Likelihood Ratio Chi-Square	1	29.9922	<.0001
Continuity Adj. Chi-Square	1	27.7885	<.0001
Mantel-Haenszel Chi-Square	1	28.8475	<.0001
Phi Coefficient		0.1036	
Contingency Coefficient		0.1030	
Cramer's V		0.1036	

Fisher's Exact Test	
Cell (1,1) Frequency (F)	77
Left-sided Pr <= F	1.0000
Right-sided Pr >= F	<.0001
Table Probability (P)	<.0001
Two-sided Pr <= P	<.0001

Sample Size = 2690
Frequency Missing = 1

Frequency
Percent
Row Pct
Col Pct

Table of STEMI_NSTEMI by Discharge_Status			
	Discharge_Status(Discharge Status)		
STEMI_NSTEMI(STEMI/NSTEMI)	1	2	Total
1	1276<br>47.43<br>97.18<br>48.61	37<br>1.38<br>2.82<br>56.92	1313<br>48.81
2	1349<br>50.15<br>97.97<br>51.39	28<br>1.04<br>2.03<br>43.08	1377<br>51.19
Total	2625<br>97.58	65<br>2.42	2690<br>100.00
Frequency Missing = 1			

*in-hospital outcomes;*1: >50, 2: 40-50%, 3: 30-40%, 4:<30%;

The FREQ Procedure

Statistics for Table of STEMI_NSTEMI by Discharge_Status

Statistic	DF	Value	Prob
Chi-Square	1	1.7546	0.1853
Likelihood Ratio Chi-Square	1	1.7577	0.1849
Continuity Adj. Chi-Square	1	1.4376	0.2305
Mantel-Haenszel Chi-Square	1	1.7539	0.1854
Phi Coefficient		-0.0255	
Contingency Coefficient		0.0255	
Cramer's V		-0.0255	

Fisher's Exact Test	
Cell (1,1) Frequency (F)	1276
Left-sided Pr <= F	0.1152
Right-sided Pr >= F	0.9266
Table Probability (P)	0.0418
Two-sided Pr <= P	0.2095

Sample Size = 2690
Frequency Missing = 1

*in-hospital outcomes;*1: >50, 2: 40-50%, 3: 30-40%, 4:<30%;

The FREQ Procedure

Frequency<br>Percent<br>Row Pct<br>Col Pct

Table of Gender by Echo_Options

Gender(Gender)	Echo_Options(Echo-Options) 1	2	3	4	Total
1	757<br>31.74<br>39.00<br>79.94	625<br>26.21<br>32.20<br>81.27	381<br>15.97<br>19.63<br>82.83	178<br>7.46<br>9.17<br>85.17	1941<br>81.38
2	190<br>7.97<br>42.79<br>20.06	144<br>6.04<br>32.43<br>18.73	79<br>3.31<br>17.79<br>17.17	31<br>1.30<br>6.98<br>14.83	444<br>18.62
Total	947<br>39.71	769<br>32.24	460<br>19.29	209<br>8.76	2385<br>100.00

Frequency Missing = 306

Statistics for Table of Gender by Echo_Options

Statistic	DF	Value	Prob
Chi-Square	3	3.9216	0.2701
Likelihood Ratio Chi-Square	3	4.0255	0.2587
Mantel-Haenszel Chi-Square	1	3.8483	0.0498
Phi Coefficient		0.0405	
Contingency Coefficient		0.0405	
Cramer's V		0.0405	

Sample Size = 2385
Frequency Missing = 306

WARNING: 11% of the data are missing.

Frequency<br>Percent<br>Row Pct<br>Col Pct

Table of Gender by Elective_coronary_angiogram

Gender(Gender)	Elective_coronary_angiogram(Elective coronary angiogram) 1	2	Total
1	356<br>20.46<br>24.88<br>85.58	1075<br>61.78<br>75.12<br>81.19	1431<br>82.24
2	60<br>3.45<br>19.42<br>14.42	249<br>14.31<br>80.58<br>18.81	309<br>17.76
Total	416<br>23.91	1324<br>76.09	1740<br>100.00

Frequency Missing = 951

*in-hospital outcomes;*1: >50, 2: 40-50%, 3: 30-40%, 4:<30%;

The FREQ Procedure

Statistics for Table of Gender by Elective_coronary_angiogram

Statistic	DF	Value	Prob
Chi-Square	1	4.1647	0.0413
Likelihood Ratio Chi-Square	1	4.3229	0.0376
Continuity Adj. Chi-Square	1	3.8700	0.0492
Mantel-Haenszel Chi-Square	1	4.1624	0.0413
Phi Coefficient		0.0489	
Contingency Coefficient		0.0489	
Cramer's V		0.0489	

Fisher's Exact Test	
Cell (1,1) Frequency (F)	356
Left-sided Pr <= F	0.9841
Right-sided Pr >= F	0.0231
Table Probability (P)	0.0072
Two-sided Pr <= P	0.0469

Sample Size = 1740
Frequency Missing = 951

WARNING: 35% of the data are missing.

Frequency<br>Percent<br>Row Pct<br>Col Pct

Table of Gender by Recurrent_MI			
	Recurrent_MI(Recurrent MI)		
Gender(Gender)	1	2	Total
1	71<br>2.64<br>3.21<br>88.75	2138<br>79.48<br>96.79<br>81.92	2209<br>82.12
2	9<br>0.33<br>1.87<br>11.25	472<br>17.55<br>98.13<br>18.08	481<br>17.88
Total	80<br>2.97	2610<br>97.03	2690<br>100.00
Frequency Missing = 1			

*in-hospital outcomes;*1: >50, 2: 40-50%, 3: 30-40%, 4:<30%;

The FREQ Procedure

Statistics for Table of Gender by Recurrent_MI

Statistic	DF	Value	Prob
Chi-Square	1	2.4690	0.1161
Likelihood Ratio Chi-Square	1	2.7594	0.0967
Continuity Adj. Chi-Square	1	2.0255	0.1547
Mantel-Haenszel Chi-Square	1	2.4681	0.1162
Phi Coefficient		0.0303	
Contingency Coefficient		0.0303	
Cramer's V		0.0303	

Fisher's Exact Test	
Cell (1,1) Frequency (F)	71
Left-sided Pr <= F	0.9637
Right-sided Pr >= F	0.0719
Table Probability (P)	0.0356
Two-sided Pr <= P	0.1380

Sample Size = 2690
Frequency Missing = 1

Frequency<br>Percent<br>Row Pct<br>Col Pct

Table of Gender by Recurrent_ischemia			
	Recurrent_ischemia(Recurrent ischemia)		
Gender(Gender)	1	2	Total
1	276<br>10.26<br>12.49<br>76.03	1933<br>71.86<br>87.51<br>83.07	2209<br>82.12
2	87<br>3.23<br>18.09<br>23.97	394<br>14.65<br>81.91<br>16.93	481<br>17.88
Total	363<br>13.49	2327<br>86.51	2690<br>100.00
Frequency Missing = 1			

*in-hospital outcomes;*1: >50, 2: 40-50%, 3: 30-40%, 4:<30%;

The FREQ Procedure

Statistics for Table of Gender by Recurrent_ischemia

Statistic	DF	Value	Prob
Chi-Square	1	10.5846	0.0011
Likelihood Ratio Chi-Square	1	9.9149	0.0016
Continuity Adj. Chi-Square	1	10.1109	0.0015
Mantel-Haenszel Chi-Square	1	10.5807	0.0011
Phi Coefficient		-0.0627	
Contingency Coefficient		0.0626	
Cramer's V		-0.0627	

Fisher's Exact Test	
Cell (1,1) Frequency (F)	276
Left-sided Pr <= F	0.0010
Right-sided Pr >= F	0.9994
Table Probability (P)	0.0004
Two-sided Pr <= P	0.0015

Sample Size = 2690
Frequency Missing = 1

Frequency<br>Percent<br>Row Pct<br>Col Pct

Table of Gender by Stroke			
	Stroke(Stroke)		
Gender(Gender)	1	2	Total
1	29<br>1.08<br>1.31<br>82.86	2180<br>81.04<br>98.69<br>82.11	2209<br>82.12
2	6<br>0.22<br>1.25<br>17.14	475<br>17.66<br>98.75<br>17.89	481<br>17.88
Total	35<br>1.30	2655<br>98.70	2690<br>100.00
Frequency Missing = 1			

*in-hospital outcomes;*1: >50, 2: 40-50%, 3: 30-40%, 4:<30%;

The FREQ Procedure

Statistics for Table of Gender by Stroke

Statistic	DF	Value	Prob
Chi-Square	1	0.0132	0.9087
Likelihood Ratio Chi-Square	1	0.0133	0.9082
Continuity Adj. Chi-Square	1	0.0000	1.0000
Mantel-Haenszel Chi-Square	1	0.0132	0.9087
Phi Coefficient		0.0022	
Contingency Coefficient		0.0022	
Cramer's V		0.0022	

Fisher's Exact Test	
Cell (1,1) Frequency (F)	29
Left-sided Pr <= F	0.6153
Right-sided Pr >= F	0.5610
Table Probability (P)	0.1763
Two-sided Pr <= P	1.0000

Sample Size = 2690
Frequency Missing = 1

Frequency<br>Percent<br>Row Pct<br>Col Pct

Table of Gender by Cardiogenic_Shock			
	Cardiogenic_Shock(Cardiogenic Shock)		
Gender(Gender)	1	2	Total
1	84<br>3.12<br>3.80<br>82.35	2125<br>79.00<br>96.20<br>82.11	2209<br>82.12
2	18<br>0.67<br>3.74<br>17.65	463<br>17.21<br>96.26<br>17.89	481<br>17.88
Total	102<br>3.79	2588<br>96.21	2690<br>100.00
Frequency Missing = 1			

*in-hospital outcomes;*1: >50, 2: 40-50%, 3: 30-40%, 4:<30%;

The FREQ Procedure

Statistics for Table of Gender by Cardiogenic_Shock

Statistic	DF	Value	Prob
Chi-Square	1	0.0040	0.9499
Likelihood Ratio Chi-Square	1	0.0040	0.9498
Continuity Adj. Chi-Square	1	0.0000	1.0000
Mantel-Haenszel Chi-Square	1	0.0040	0.9499
Phi Coefficient		0.0012	
Contingency Coefficient		0.0012	
Cramer's V		0.0012	

Fisher's Exact Test	
Cell (1,1) Frequency (F)	84
Left-sided Pr <= F	0.5671
Right-sided Pr >= F	0.5378
Table Probability (P)	0.1049
Two-sided Pr <= P	1.0000

Sample Size = 2690
Frequency Missing = 1

Frequency<br>Percent<br>Row Pct<br>Col Pct

Table of Gender by Heart_Failure			
	Heart_Failure(Heart Failure)		
Gender(Gender)	1	2	Total
1	248<br>9.22<br>11.23<br>77.02	1961<br>72.90<br>88.77<br>82.81	2209<br>82.12
2	74<br>2.75<br>15.38<br>22.98	407<br>15.13<br>84.62<br>17.19	481<br>17.88
Total	322<br>11.97	2368<br>88.03	2690<br>100.00
Frequency Missing = 1			

*in-hospital outcomes;*1: >50, 2: 40-50%, 3: 30-40%, 4:<30%;

The FREQ Procedure

Statistics for Table of Gender by Heart_Failure

Statistic	DF	Value	Prob
Chi-Square	1	6.4802	0.0109
Likelihood Ratio Chi-Square	1	6.1190	0.0134
Continuity Adj. Chi-Square	1	6.0916	0.0136
Mantel-Haenszel Chi-Square	1	6.4778	0.0109
Phi Coefficient		-0.0491	
Contingency Coefficient		0.0490	
Cramer's V		-0.0491	

Fisher's Exact Test	
Cell (1,1) Frequency (F)	248
Left-sided Pr <= F	0.0079
Right-sided Pr >= F	0.9947
Table Probability (P)	0.0027
Two-sided Pr <= P	0.0130

Sample Size = 2690
Frequency Missing = 1

Frequency<br>Percent<br>Row Pct<br>Col Pct

Table of Gender by Major_bleeding			
	Major_bleeding(Major bleeding)		
Gender(Gender)	1	2	Total
1	13<br>0.48<br>0.59<br>68.42	2196<br>81.64<br>99.41<br>82.22	2209<br>82.12
2	6<br>0.22<br>1.25<br>31.58	475<br>17.66<br>98.75<br>17.78	481<br>17.88
Total	19<br>0.71	2671<br>99.29	2690<br>100.00
Frequency Missing = 1			

*in-hospital outcomes;*1: >50, 2: 40-50%, 3: 30-40%, 4:<30%;

The FREQ Procedure

Statistics for Table of Gender by Major_bleeding

Statistic	DF	Value	Prob
Chi-Square	1	2.4451	0.1179
Likelihood Ratio Chi-Square	1	2.0976	0.1475
Continuity Adj. Chi-Square	1	1.5959	0.2065
Mantel-Haenszel Chi-Square	1	2.4442	0.1180
Phi Coefficient		-0.0301	
Contingency Coefficient		0.0301	
Cramer's V		-0.0301	
WARNING: 25% of the cells have expected counts less than 5. Chi-Square may not be a valid test.			

Fisher's Exact Test	
Cell (1,1) Frequency (F)	13
Left-sided Pr <= F	0.1075
Right-sided Pr >= F	0.9608
Table Probability (P)	0.0683
Two-sided Pr <= P	0.1309

Sample Size = 2690
Frequency Missing = 1

Frequency<br>Percent<br>Row Pct<br>Col Pct

Table of Gender by VT_VF_arrest			
	VT_VF_arrest(VT/VF arrest)		
Gender(Gender)	1	2	Total
1	89<br>3.31<br>4.03<br>86.41	2120<br>78.81<br>95.97<br>81.95	2209<br>82.12
2	14<br>0.52<br>2.91<br>13.59	467<br>17.36<br>97.09<br>18.05	481<br>17.88
Total	103<br>3.83	2587<br>96.17	2690<br>100.00
Frequency Missing = 1			

*in-hospital outcomes;*1: >50, 2: 40-50%, 3: 30-40%, 4:<30%;

The FREQ Procedure

Statistics for Table of Gender by VT_VF_arrest

Statistic	DF	Value	Prob
Chi-Square	1	1.3416	0.2467
Likelihood Ratio Chi-Square	1	1.4342	0.2311
Continuity Adj. Chi-Square	1	1.0551	0.3043
Mantel-Haenszel Chi-Square	1	1.3411	0.2468
Phi Coefficient		0.0223	
Contingency Coefficient		0.0223	
Cramer's V		0.0223	

Fisher's Exact Test	
Cell (1,1) Frequency (F)	89
Left-sided Pr <= F	0.9050
Right-sided Pr >= F	0.1516
Table Probability (P)	0.0565
Two-sided Pr <= P	0.2943

Sample Size = 2690
Frequency Missing = 1

Frequency<br>Percent<br>Row Pct<br>Col Pct

Table of Gender by Atrial_Fibrillation_Flutter			
	Atrial_Fibrillation_Flutter(Atrial Fibrillation/Flutter)		
Gender(Gender)	1	2	Total
1	74<br>2.75<br>3.35<br>71.84	2135<br>79.37<br>96.65<br>82.53	2209<br>82.12
2	29<br>1.08<br>6.03<br>28.16	452<br>16.80<br>93.97<br>17.47	481<br>17.88
Total	103<br>3.83	2587<br>96.17	2690<br>100.00
Frequency Missing = 1			

*in-hospital outcomes;*1: >50, 2: 40-50%, 3: 30-40%, 4:<30%;

The FREQ Procedure

Statistics for Table of Gender by Atrial_Fibrillation_Flutter

Statistic	DF	Value	Prob
Chi-Square	1	7.6995	0.0055
Likelihood Ratio Chi-Square	1	6.8463	0.0089
Continuity Adj. Chi-Square	1	6.9891	0.0082
Mantel-Haenszel Chi-Square	1	7.6966	0.0055
Phi Coefficient		-0.0535	
Contingency Coefficient		0.0534	
Cramer's V		-0.0535	

Fisher's Exact Test	
Cell (1,1) Frequency (F)	74
Left-sided Pr <= F	0.0058
Right-sided Pr >= F	0.9971
Table Probability (P)	0.0029
Two-sided Pr <= P	0.0083

Sample Size = 2690
Frequency Missing = 1

Frequency<br>Percent<br>Row Pct<br>Col Pct

Table of Gender by Discharge_Status			
	Discharge_Status(Discharge Status)		
Gender(Gender)	1	2	Total
1	2155<br>80.11<br>97.56<br>82.10	54<br>2.01<br>2.44<br>83.08	2209<br>82.12
2	470<br>17.47<br>97.71<br>17.90	11<br>0.41<br>2.29<br>16.92	481<br>17.88
Total	2625<br>97.58	65<br>2.42	2690<br>100.00
Frequency Missing = 1			

*in-hospital outcomes;*1: >50, 2: 40-50%, 3: 30-40%, 4:<30%;

The FREQ Procedure

Statistics for Table of Gender by Discharge_Status

Statistic	DF	Value	Prob
Chi-Square	1	0.0416	0.8383
Likelihood Ratio Chi-Square	1	0.0422	0.8372
Continuity Adj. Chi-Square	1	0.0016	0.9679
Mantel-Haenszel Chi-Square	1	0.0416	0.8384
Phi Coefficient		-0.0039	
Contingency Coefficient		0.0039	
Cramer's V		-0.0039	

Fisher's Exact Test	
Cell (1,1) Frequency (F)	2155
Left-sided Pr <= F	0.4975
Right-sided Pr >= F	0.6323
Table Probability (P)	0.1297
Two-sided Pr <= P	1.0000

Sample Size = 2690
Frequency Missing = 1

*in-hospital outcomes;*1: >50, 2: 40-50%, 3: 30-40%, 4:<30%;

The FREQ Procedure

Frequency<br>Percent<br>Row Pct<br>Col Pct

Table of Nationality by Echo_Options					
	Echo_Options(Echo-Options)				
Nationality(Nationality)	1	2	3	4	Total
1	688<br>28.85<br>41.00<br>72.65	529<br>22.18<br>31.53<br>68.79	325<br>13.63<br>19.37<br>70.65	136<br>5.70<br>8.10<br>65.07	1678<br>70.36
2	259<br>10.86<br>36.63<br>27.35	240<br>10.06<br>33.95<br>31.21	135<br>5.66<br>19.09<br>29.35	73<br>3.06<br>10.33<br>34.93	707<br>29.64
Total	947<br>39.71	769<br>32.24	460<br>19.29	209<br>8.76	2385<br>100.00
Frequency Missing = 306					

Statistics for Table of Nationality by Echo_Options

Statistic	DF	Value	Prob
Chi-Square	3	6.1115	0.1063
Likelihood Ratio Chi-Square	3	6.0542	0.1090
Mantel-Haenszel Chi-Square	1	3.8546	0.0496
Phi Coefficient		0.0506	
Contingency Coefficient		0.0506	
Cramer's V		0.0506	

Sample Size = 2385
Frequency Missing = 306

WARNING: 11% of the data are missing.

Frequency<br>Percent<br>Row Pct<br>Col Pct

Table of Nationality by Elective_coronary_angiogram			
	Elective_coronary_angiogram(Elective coronary angiogram)		
Nationality(Nationality)	1	2	Total
1	300<br>17.24<br>24.59<br>72.12	920<br>52.87<br>75.41<br>69.49	1220<br>70.11
2	116<br>6.67<br>22.31<br>27.88	404<br>23.22<br>77.69<br>30.51	520<br>29.89
Total	416<br>23.91	1324<br>76.09	1740<br>100.00
Frequency Missing = 951			

*in-hospital outcomes;*1: >50, 2: 40-50%, 3: 30-40%, 4:<30%;

The FREQ Procedure

Statistics for Table of Nationality by Elective_coronary_angiogram

Statistic	DF	Value	Prob
Chi-Square	1	1.0441	0.3069
Likelihood Ratio Chi-Square	1	1.0537	0.3046
Continuity Adj. Chi-Square	1	0.9224	0.3368
Mantel-Haenszel Chi-Square	1	1.0435	0.3070
Phi Coefficient		0.0245	
Contingency Coefficient		0.0245	
Cramer's V		0.0245	

Fisher's Exact Test	
Cell (1,1) Frequency (F)	300
Left-sided Pr <= F	0.8608
Right-sided Pr >= F	0.1685
Table Probability (P)	0.0293
Two-sided Pr <= P	0.3262

Sample Size = 1740
Frequency Missing = 951

WARNING: 35% of the data are missing.

Frequency<br>Percent<br>Row Pct<br>Col Pct

Table of Nationality by Recurrent_MI			
	Recurrent_MI(Recurrent MI)		
Nationality(Nationality)	1	2	Total
1	62<br>2.30<br>3.33<br>77.50	1800<br>66.91<br>96.67<br>68.97	1862<br>69.22
2	18<br>0.67<br>2.17<br>22.50	810<br>30.11<br>97.83<br>31.03	828<br>30.78
Total	80<br>2.97	2610<br>97.03	2690<br>100.00
Frequency Missing = 1			

*in-hospital outcomes;*1: >50, 2: 40-50%, 3: 30-40%, 4:<30%;

The FREQ Procedure

Statistics for Table of Nationality by Recurrent_MI

Statistic	DF	Value	Prob
Chi-Square	1	2.6535	0.1033
Likelihood Ratio Chi-Square	1	2.8091	0.0937
Continuity Adj. Chi-Square	1	2.2681	0.1321
Mantel-Haenszel Chi-Square	1	2.6526	0.1034
Phi Coefficient		0.0314	
Contingency Coefficient		0.0314	
Cramer's V		0.0314	

Fisher's Exact Test	
Cell (1,1) Frequency (F)	62
Left-sided Pr <= F	0.9633
Right-sided Pr >= F	0.0632
Table Probability (P)	0.0264
Two-sided Pr <= P	0.1108

Sample Size = 2690
Frequency Missing = 1

Frequency<br>Percent<br>Row Pct<br>Col Pct

Table of Nationality by Recurrent_ischemia			
	Recurrent_ischemia(Recurrent ischemia)		
Nationality(Nationality)	1	2	Total
1	240<br>8.92<br>12.89<br>66.12	1622<br>60.30<br>87.11<br>69.70	1862<br>69.22
2	123<br>4.57<br>14.86<br>33.88	705<br>26.21<br>85.14<br>30.30	828<br>30.78
Total	363<br>13.49	2327<br>86.51	2690<br>100.00
Frequency Missing = 1			

*in-hospital outcomes;*1: >50, 2: 40-50%, 3: 30-40%, 4:<30%;

The FREQ Procedure

Statistics for Table of Nationality by Recurrent_ischemia

Statistic	DF	Value	Prob
Chi-Square	1	1.8971	0.1684
Likelihood Ratio Chi-Square	1	1.8692	0.1716
Continuity Adj. Chi-Square	1	1.7325	0.1881
Mantel-Haenszel Chi-Square	1	1.8964	0.1685
Phi Coefficient		-0.0266	
Contingency Coefficient		0.0265	
Cramer's V		-0.0266	

Fisher's Exact Test	
Cell (1,1) Frequency (F)	240
Left-sided Pr <= F	0.0947
Right-sided Pr >= F	0.9240
Table Probability (P)	0.0187
Two-sided Pr <= P	0.1787

Sample Size = 2690
Frequency Missing = 1

Frequency
Percent
Row Pct
Col Pct

Table of Nationality by Stroke			
	Stroke(Stroke)		
Nationality(Nationality)	1	2	Total
1	29<br>1.08<br>1.56<br>82.86	1833<br>68.14<br>98.44<br>69.04	1862<br>69.22
2	6<br>0.22<br>0.72<br>17.14	822<br>30.56<br>99.28<br>30.96	828<br>30.78
Total	35<br>1.30	2655<br>98.70	2690<br>100.00
Frequency Missing = 1			

*in-hospital outcomes;*1: >50, 2: 40-50%, 3: 30-40%, 4:<30%;

The FREQ Procedure

Statistics for Table of Nationality by Stroke

Statistic	DF	Value	Prob
Chi-Square	1	3.0956	0.0785
Likelihood Ratio Chi-Square	1	3.4472	0.0634
Continuity Adj. Chi-Square	1	2.4810	0.1152
Mantel-Haenszel Chi-Square	1	3.0944	0.0786
Phi Coefficient		0.0339	
Contingency Coefficient		0.0339	
Cramer's V		0.0339	

Fisher's Exact Test	
Cell (1,1) Frequency (F)	29
Left-sided Pr <= F	0.9791
Right-sided Pr >= F	0.0525
Table Probability (P)	0.0316
Two-sided Pr <= P	0.0964

Sample Size = 2690
Frequency Missing = 1

Frequency<br>Percent<br>Row Pct<br>Col Pct

Table of Nationality by Cardiogenic_Shock			
	Cardiogenic_Shock(Cardiogenic Shock)		
Nationality(Nationality)	1	2	Total
1	64<br>2.38<br>3.44<br>62.75	1798<br>66.84<br>96.56<br>69.47	1862<br>69.22
2	38<br>1.41<br>4.59<br>37.25	790<br>29.37<br>95.41<br>30.53	828<br>30.78
Total	102<br>3.79	2588<br>96.21	2690<br>100.00
Frequency Missing = 1			

*in-hospital outcomes;*1: >50, 2: 40-50%, 3: 30-40%, 4:<30%;

The FREQ Procedure

Statistics for Table of Nationality by Cardiogenic_Shock

Statistic	DF	Value	Prob
Chi-Square	1	2.0857	0.1487
Likelihood Ratio Chi-Square	1	2.0178	0.1555
Continuity Adj. Chi-Square	1	1.7819	0.1819
Mantel-Haenszel Chi-Square	1	2.0850	0.1488
Phi Coefficient		-0.0278	
Contingency Coefficient		0.0278	
Cramer's V		-0.0278	

Fisher's Exact Test	
Cell (1,1) Frequency (F)	64
Left-sided Pr <= F	0.0924
Right-sided Pr >= F	0.9380
Table Probability (P)	0.0304
Two-sided Pr <= P	0.1557

Sample Size = 2690
Frequency Missing = 1

Frequency<br>Percent<br>Row Pct<br>Col Pct

Table of Nationality by Heart_Failure			
	Heart_Failure(Heart Failure)		
Nationality(Nationality)	1	2	Total
1	228<br>8.48<br>12.24<br>70.81	1634<br>60.74<br>87.76<br>69.00	1862<br>69.22
2	94<br>3.49<br>11.35<br>29.19	734<br>27.29<br>88.65<br>31.00	828<br>30.78
Total	322<br>11.97	2368<br>88.03	2690<br>100.00
Frequency Missing = 1			

*in-hospital outcomes;*1: >50, 2: 40-50%, 3: 30-40%, 4:<30%;

The FREQ Procedure

Statistics for Table of Nationality by Heart_Failure

Statistic	DF	Value	Prob
Chi-Square	1	0.4330	0.5105
Likelihood Ratio Chi-Square	1	0.4367	0.5087
Continuity Adj. Chi-Square	1	0.3525	0.5527
Mantel-Haenszel Chi-Square	1	0.4328	0.5106
Phi Coefficient		0.0127	
Contingency Coefficient		0.0127	
Cramer's V		0.0127	

Fisher's Exact Test	
Cell (1,1) Frequency (F)	228
Left-sided Pr <= F	0.7640
Right-sided Pr >= F	0.2778
Table Probability (P)	0.0418
Two-sided Pr <= P	0.5626

Sample Size = 2690
Frequency Missing = 1

Frequency<br>Percent<br>Row Pct<br>Col Pct

Table of Nationality by Major_bleeding			
	Major_bleeding(Major bleeding)		
Nationality(Nationality)	1	2	Total
1	14<br>0.52<br>0.75<br>73.68	1848<br>68.70<br>99.25<br>69.19	1862<br>69.22
2	5<br>0.19<br>0.60<br>26.32	823<br>30.59<br>99.40<br>30.81	828<br>30.78
Total	19<br>0.71	2671<br>99.29	2690<br>100.00
Frequency Missing = 1			

*in-hospital outcomes;*1: >50, 2: 40-50%, 3: 30-40%, 4:<30%;

The FREQ Procedure

Statistics for Table of Nationality by Major_bleeding

Statistic	DF	Value	Prob
Chi-Square	1	0.1790	0.6722
Likelihood Ratio Chi-Square	1	0.1843	0.6677
Continuity Adj. Chi-Square	1	0.0302	0.8621
Mantel-Haenszel Chi-Square	1	0.1790	0.6723
Phi Coefficient		0.0082	
Contingency Coefficient		0.0082	
Cramer's V		0.0082	

Fisher's Exact Test	
Cell (1,1) Frequency (F)	14
Left-sided Pr <= F	0.7429
Right-sided Pr >= F	0.4438
Table Probability (P)	0.1867
Two-sided Pr <= P	0.8061

Sample Size = 2690
Frequency Missing = 1

Frequency<br>Percent<br>Row Pct<br>Col Pct

Table of Nationality by VT_VF_arrest			
	VT_VF_arrest(VT/VF arrest)		
Nationality(Nationality)	1	2	Total
1	65<br>2.42<br>3.49<br>63.11	1797<br>66.80<br>96.51<br>69.46	1862<br>69.22
2	38<br>1.41<br>4.59<br>36.89	790<br>29.37<br>95.41<br>30.54	828<br>30.78
Total	103<br>3.83	2587<br>96.17	2690<br>100.00
Frequency Missing = 1			

*in-hospital outcomes;*1: >50, 2: 40-50%, 3: 30-40%, 4:<30%;

The FREQ Procedure

Statistics for Table of Nationality by VT_VF_arrest

Statistic	DF	Value	Prob
Chi-Square	1	1.8782	0.1705
Likelihood Ratio Chi-Square	1	1.8200	0.1773
Continuity Adj. Chi-Square	1	1.5917	0.2071
Mantel-Haenszel Chi-Square	1	1.8775	0.1706
Phi Coefficient		-0.0264	
Contingency Coefficient		0.0264	
Cramer's V		-0.0264	

Fisher's Exact Test	
Cell (1,1) Frequency (F)	65
Left-sided Pr <= F	0.1047
Right-sided Pr >= F	0.9287
Table Probability (P)	0.0334
Two-sided Pr <= P	0.1912

Sample Size = 2690
Frequency Missing = 1

Frequency<br>Percent<br>Row Pct<br>Col Pct

Table of Nationality by Atrial_Fibrillation_Flutter			
	Atrial_Fibrillation_Flutter(Atrial Fibrillation/Flutter)		
Nationality(Nationality)	1	2	Total
1	87<br>3.23<br>4.67<br>84.47	1775<br>65.99<br>95.33<br>68.61	1862<br>69.22
2	16<br>0.59<br>1.93<br>15.53	812<br>30.19<br>98.07<br>31.39	828<br>30.78
Total	103<br>3.83	2587<br>96.17	2690<br>100.00
Frequency Missing = 1			

*in-hospital outcomes;*1: >50, 2: 40-50%, 3: 30-40%, 4:<30%;

The FREQ Procedure

Statistics for Table of Nationality by Atrial_Fibrillation_Flutter

Statistic	DF	Value	Prob
Chi-Square	1	11.6853	0.0006
Likelihood Ratio Chi-Square	1	13.2004	0.0003
Continuity Adj. Chi-Square	1	10.9530	0.0009
Mantel-Haenszel Chi-Square	1	11.6809	0.0006
Phi Coefficient		0.0659	
Contingency Coefficient		0.0658	
Cramer's V		0.0659	

Fisher's Exact Test	
Cell (1,1) Frequency (F)	87
Left-sided Pr <= F	0.9999
Right-sided Pr >= F	0.0002
Table Probability (P)	0.0001
Two-sided Pr <= P	0.0004

Sample Size = 2690
Frequency Missing = 1

Frequency
Percent
Row Pct
Col Pct

Table of Nationality by Discharge_Status			
	Discharge_Status(Discharge Status)		
Nationality(Nationality)	1	2	Total
1	1817<br>67.55<br>97.58<br>69.22	45<br>1.67<br>2.42<br>69.23	1862<br>69.22
2	808<br>30.04<br>97.58<br>30.78	20<br>0.74<br>2.42<br>30.77	828<br>30.78
Total	2625<br>97.58	65<br>2.42	2690<br>100.00
Frequency Missing = 1			

*in-hospital outcomes;*1: >50, 2: 40-50%, 3: 30-40%, 4:<30%;

The FREQ Procedure

Statistics for Table of Nationality by Discharge_Status

Statistic	DF	Value	Prob
Chi-Square	1	0.0000	0.9984
Likelihood Ratio Chi-Square	1	0.0000	0.9984
Continuity Adj. Chi-Square	1	0.0000	1.0000
Mantel-Haenszel Chi-Square	1	0.0000	0.9984
Phi Coefficient		-0.0000	
Contingency Coefficient		0.0000	
Cramer's V		-0.0000	

Fisher's Exact Test	
Cell (1,1) Frequency (F)	1817
Left-sided Pr <= F	0.5597
Right-sided Pr >= F	0.5483
Table Probability (P)	0.1080
Two-sided Pr <= P	1.0000

Sample Size = 2690
Frequency Missing = 1

The FREQ Procedure

Frequency<br>Percent<br>Row Pct<br>Col Pct

Table of Atrial_Fibrillation_Flutter by Gender			
Atrial_Fibrillation_Flutter(Atrial Fibrillation/Flutter)	Gender(Gender)		
	1	2	Total
1	74<br>2.75<br>71.84<br>3.35	29<br>1.08<br>28.16<br>6.03	103<br>3.83
2	2135<br>79.37<br>82.53<br>96.65	452<br>16.80<br>17.47<br>93.97	2587<br>96.17
Total	2209<br>82.12	481<br>17.88	2690<br>100.00
Frequency Missing = 1			

Statistics for Table of Atrial_Fibrillation_Flutter by Gender

Statistic	DF	Value	Prob
Chi-Square	1	7.6995	0.0055
Likelihood Ratio Chi-Square	1	6.8463	0.0089
Continuity Adj. Chi-Square	1	6.9891	0.0082
Mantel-Haenszel Chi-Square	1	7.6966	0.0055
Phi Coefficient		-0.0535	
Contingency Coefficient		0.0534	
Cramer's V		-0.0535	

Fisher's Exact Test	
Cell (1,1) Frequency (F)	74
Left-sided Pr <= F	0.0058
Right-sided Pr >= F	0.9971
Table Probability (P)	0.0029
Two-sided Pr <= P	0.0083

Sample Size = 2690
Frequency Missing = 1

The FREQ Procedure

Frequency<br>Percent<br>Row Pct<br>Col Pct

Table of Atrial_Fibrillation_Flutter by Nationality			
Atrial_Fibrillation_Flutter(Atrial Fibrillation/Flutter)	Nationality(Nationality)		
	1	2	Total
1	87<br>3.23<br>84.47<br>4.67	16<br>0.59<br>15.53<br>1.93	103<br>3.83
2	1775<br>65.99<br>68.61<br>95.33	812<br>30.19<br>31.39<br>98.07	2587<br>96.17
Total	1862<br>69.22	828<br>30.78	2690<br>100.00
Frequency Missing = 1			

Statistics for Table of Atrial_Fibrillation_Flutter by Nationality

Statistic	DF	Value	Prob
Chi-Square	1	11.6853	0.0006
Likelihood Ratio Chi-Square	1	13.2004	0.0003
Continuity Adj. Chi-Square	1	10.9530	0.0009
Mantel-Haenszel Chi-Square	1	11.6809	0.0006
Phi Coefficient		0.0659	
Contingency Coefficient		0.0658	
Cramer's V		0.0659	

Fisher's Exact Test	
Cell (1,1) Frequency (F)	87
Left-sided Pr <= F	0.9999
Right-sided Pr >= F	0.0002
Table Probability (P)	0.0001
Two-sided Pr <= P	0.0004

Sample Size = 2690
Frequency Missing = 1

The FREQ Procedure

Frequency<br>Percent<br>Row Pct<br>Col Pct

Table of Atrial_Fibrillation_Flutter by Ethnicity

Atrial_Fibrillation_Flutter(Atrial Fibrillation/Flutter)	Ethnicity(Ethnicity)			
	1	2	3	Total
1	96<br>3.57<br>93.20<br>4.57	6<br>0.22<br>5.83<br>1.19	1<br>0.04<br>0.97<br>1.16	103<br>3.83
2	2003<br>74.46<br>77.43<br>95.43	499<br>18.55<br>19.29<br>98.81	85<br>3.16<br>3.29<br>98.84	2587<br>96.17
Total	2099<br>78.03	505<br>18.77	86<br>3.20	2690<br>100.00

Frequency Missing = 1

Statistics for Table of Atrial_Fibrillation_Flutter by Ethnicity

Statistic	DF	Value	Prob
Chi-Square	2	14.3850	0.0008
Likelihood Ratio Chi-Square	2	18.2392	0.0001
Mantel-Haenszel Chi-Square	1	12.8491	0.0003
Phi Coefficient		0.0731	
Contingency Coefficient		0.0729	
Cramer's V		0.0731	

Sample Size = 2690
Frequency Missing = 1

Frequency<br>Percent<br>Row Pct<br>Col Pct

Table of Atrial_Fibrillation_Flutter by Type_of_STEMI

Atrial_Fibrillation_Flutter(Atrial Fibrillation/Flutter)	Type_of_STEMI(Type of STEMI)			
	1	2	3	Total
1	18<br>1.37<br>54.55<br>2.61	11<br>0.84<br>33.33<br>2.02	4<br>0.30<br>12.12<br>5.13	33<br>2.51
2	672<br>51.18<br>52.50<br>97.39	534<br>40.67<br>41.72<br>97.98	74<br>5.64<br>5.78<br>94.87	1280<br>97.49
Total	690<br>52.55	545<br>41.51	78<br>5.94	1313<br>100.00

Frequency Missing = 1378

The FREQ Procedure

Statistics for Table of Atrial_Fibrillation_Flutter by Type_of_STEMI

Statistic	DF	Value	Prob
Chi-Square	2	2.7473	0.2532
Likelihood Ratio Chi-Square	2	2.2902	0.3182
Mantel-Haenszel Chi-Square	1	0.1613	0.6880
Phi Coefficient		0.0457	
Contingency Coefficient		0.0457	
Cramer's V		0.0457	

Sample Size = 1313
Frequency Missing = 1378

WARNING: 51% of the data are missing.

Frequency<br>Percent<br>Row Pct<br>Col Pct

Table of Atrial_Fibrillation_Flutter by History_of_angina			
Atrial_Fibrillation_Flutter(Atrial Fibrillation/Flutter)	History_of_angina(History of angina)		
	1	2	Total
1	47<br>1.75<br>45.63<br>6.35	56<br>2.08<br>54.37<br>2.87	103<br>3.83
2	693<br>25.76<br>26.79<br>93.65	1894<br>70.41<br>73.21<br>97.13	2587<br>96.17
Total	740<br>27.51	1950<br>72.49	2690<br>100.00
Frequency Missing = 1			

The FREQ Procedure

Statistics for Table of Atrial_Fibrillation_Flutter by History_of_angina

Statistic	DF	Value	Prob
Chi-Square	1	17.6373	<.0001
Likelihood Ratio Chi-Square	1	16.0306	<.0001
Continuity Adj. Chi-Square	1	16.7051	<.0001
Mantel-Haenszel Chi-Square	1	17.6308	<.0001
Phi Coefficient		0.0810	
Contingency Coefficient		0.0807	
Cramer's V		0.0810	

Fisher's Exact Test	
Cell (1,1) Frequency (F)	47
Left-sided Pr <= F	1.0000
Right-sided Pr >= F	<.0001
Table Probability (P)	<.0001
Two-sided Pr <= P	<.0001

Sample Size = 2690
Frequency Missing = 1

Frequency
Percent
Row Pct
Col Pct

Table of Atrial_Fibrillation_Flutter by History_of_MI			
Atrial_Fibrillation_Flutter(Atrial Fibrillation/Flutter)	History_of_MI(History of MI)		
	1	2	Total
1	30<br>1.12<br>29.13<br>7.44	73<br>2.71<br>70.87<br>3.19	103<br>3.83
2	373<br>13.87<br>14.42<br>92.56	2214<br>82.30<br>85.58<br>96.81	2587<br>96.17
Total	403<br>14.98	2287<br>85.02	2690<br>100.00
Frequency Missing = 1			

The FREQ Procedure

Statistics for Table of Atrial_Fibrillation_Flutter by History_of_MI

Statistic	DF	Value	Prob
Chi-Square	1	16.8236	<.0001
Likelihood Ratio Chi-Square	1	13.9731	0.0002
Continuity Adj. Chi-Square	1	15.6887	<.0001
Mantel-Haenszel Chi-Square	1	16.8174	<.0001
Phi Coefficient		0.0791	
Contingency Coefficient		0.0788	
Cramer's V		0.0791	

Fisher's Exact Test	
Cell (1,1) Frequency (F)	30
Left-sided Pr <= F	0.9999
Right-sided Pr >= F	0.0001
Table Probability (P)	<.0001
Two-sided Pr <= P	0.0002

Sample Size = 2690
Frequency Missing = 1

Frequency<br>Percent<br>Row Pct<br>Col Pct

Table of Atrial_Fibrillation_Flutter by History_of_PCI			
Atrial_Fibrillation_Flutter(Atrial Fibrillation/Flutter)	History_of_PCI(History of PCI)		
	1	2	Total
1	22<br>0.82<br>21.36<br>5.25	81<br>3.01<br>78.64<br>3.57	103<br>3.83
2	397<br>14.76<br>15.35<br>94.75	2190<br>81.41<br>84.65<br>96.43	2587<br>96.17
Total	419<br>15.58	2271<br>84.42	2690<br>100.00
Frequency Missing = 1			

The FREQ Procedure

Statistics for Table of Atrial_Fibrillation_Flutter by History_of_PCI

Statistic	DF	Value	Prob
Chi-Square	1	2.7238	0.0989
Likelihood Ratio Chi-Square	1	2.5019	0.1137
Continuity Adj. Chi-Square	1	2.2857	0.1306
Mantel-Haenszel Chi-Square	1	2.7228	0.0989
Phi Coefficient		0.0318	
Contingency Coefficient		0.0318	
Cramer's V		0.0318	

Fisher's Exact Test	
Cell (1,1) Frequency (F)	22
Left-sided Pr <= F	0.9587
Right-sided Pr >= F	0.0693
Table Probability (P)	0.0281
Two-sided Pr <= P	0.1257

Sample Size = 2690
Frequency Missing = 1

Frequency<br>Percent<br>Row Pct<br>Col Pct

Table of Atrial_Fibrillation_Flutter by History_of_CABG			
Atrial_Fibrillation_Flutter(Atrial Fibrillation/Flutter)	History_of_CABG(History of CABG)		
	1	2	Total
1	15<br>0.56<br>14.56<br>21.13	88<br>3.27<br>85.44<br>3.36	103<br>3.83
2	56<br>2.08<br>2.16<br>78.87	2531<br>94.09<br>97.84<br>96.64	2587<br>96.17
Total	71<br>2.64	2619<br>97.36	2690<br>100.00
Frequency Missing = 1			

The FREQ Procedure

Statistics for Table of Atrial_Fibrillation_Flutter by History_of_CABG

Statistic	DF	Value	Prob
Chi-Square	1	59.2551	<.0001
Likelihood Ratio Chi-Square	1	30.6595	<.0001
Continuity Adj. Chi-Square	1	54.5286	<.0001
Mantel-Haenszel Chi-Square	1	59.2331	<.0001
Phi Coefficient		0.1484	
Contingency Coefficient		0.1468	
Cramer's V		0.1484	
WARNING: 25% of the cells have expected counts less than 5. Chi-Square may not be a valid test.			

Fisher's Exact Test	
Cell (1,1) Frequency (F)	15
Left-sided Pr <= F	1.0000
Right-sided Pr >= F	<.0001
Table Probability (P)	<.0001
Two-sided Pr <= P	<.0001

Sample Size = 2690
Frequency Missing = 1

Frequency<br>Percent<br>Row Pct<br>Col Pct

Table of Atrial_Fibrillation_Flutter by History_of_heart_failure			
Atrial_Fibrillation_Flutter(Atrial Fibrillation/Flutter)	History_of_heart_failure(History of heart failure)		
	1	2	Total
1	23<br>0.86<br>22.33<br>14.11	80<br>2.97<br>77.67<br>3.17	103<br>3.83
2	140<br>5.20<br>5.41<br>85.89	2447<br>90.97<br>94.59<br>96.83	2587<br>96.17
Total	163<br>6.06	2527<br>93.94	2690<br>100.00
Frequency Missing = 1			

The FREQ Procedure

Statistics for Table of Atrial_Fibrillation_Flutter by History_of_heart_failure

Statistic	DF	Value	Prob
Chi-Square	1	49.8096	<.0001
Likelihood Ratio Chi-Square	1	31.5417	<.0001
Continuity Adj. Chi-Square	1	46.8818	<.0001
Mantel-Haenszel Chi-Square	1	49.7911	<.0001
Phi Coefficient		0.1361	
Contingency Coefficient		0.1348	
Cramer's V		0.1361	

Fisher's Exact Test	
Cell (1,1) Frequency (F)	23
Left-sided Pr <= F	1.0000
Right-sided Pr >= F	<.0001
Table Probability (P)	<.0001
Two-sided Pr <= P	<.0001

Sample Size = 2690
Frequency Missing = 1

Frequency<br>Percent<br>Row Pct<br>Col Pct

Table of Atrial_Fibrillation_Flutter by History_of_stroke			
Atrial_Fibrillation_Flutter(Atrial Fibrillation/Flutter)	History_of_stroke(History of stroke)		
	1	2	Total
1	13<br>0.48<br>12.62<br>10.57	90<br>3.35<br>87.38<br>3.51	103<br>3.83
2	110<br>4.09<br>4.25<br>89.43	2477<br>92.08<br>95.75<br>96.49	2587<br>96.17
Total	123<br>4.57	2567<br>95.43	2690<br>100.00
Frequency Missing = 1			

The FREQ Procedure

Statistics for Table of Atrial_Fibrillation_Flutter by History_of_stroke

Statistic	DF	Value	Prob
Chi-Square	1	15.9014	<.0001
Likelihood Ratio Chi-Square	1	11.1607	0.0008
Continuity Adj. Chi-Square	1	14.0412	0.0002
Mantel-Haenszel Chi-Square	1	15.8955	<.0001
Phi Coefficient		0.0769	
Contingency Coefficient		0.0767	
Cramer's V		0.0769	
WARNING: 25% of the cells have expected counts less than 5. Chi-Square may not be a valid test.			

Fisher's Exact Test	
Cell (1,1) Frequency (F)	13
Left-sided Pr <= F	0.9998
Right-sided Pr >= F	0.0006
Table Probability (P)	0.0005
Two-sided Pr <= P	0.0006

Sample Size = 2690
Frequency Missing = 1

Frequency<br>Percent<br>Row Pct<br>Col Pct

Table of Atrial_Fibrillation_Flutter by History_of_chronic_renal_failure			
Atrial_Fibrillation_Flutter(Atrial Fibrillation/Flutter)	History_of_chronic_renal_failure(History of chronic renal failure)		
	1	2	Total
1	19<br>0.71<br>18.45<br>9.50	84<br>3.12<br>81.55<br>3.37	103<br>3.83
2	181<br>6.73<br>7.00<br>90.50	2406<br>89.44<br>93.00<br>96.63	2587<br>96.17
Total	200<br>7.43	2490<br>92.57	2690<br>100.00
Frequency Missing = 1			

The FREQ Procedure

Statistics for Table of Atrial_Fibrillation_Flutter by History_of_chronic_renal_failure

Statistic	DF	Value	Prob
Chi-Square	1	18.8701	<.0001
Likelihood Ratio Chi-Square	1	13.9884	0.0002
Continuity Adj. Chi-Square	1	17.2430	<.0001
Mantel-Haenszel Chi-Square	1	18.8631	<.0001
Phi Coefficient		0.0838	
Contingency Coefficient		0.0835	
Cramer's V		0.0838	

Fisher's Exact Test	
Cell (1,1) Frequency (F)	19
Left-sided Pr <= F	1.0000
Right-sided Pr >= F	0.0001
Table Probability (P)	<.0001
Two-sided Pr <= P	0.0001

Sample Size = 2690
Frequency Missing = 1

Frequency<br>Percent<br>Row Pct<br>Col Pct

Table of Atrial_Fibrillation_Flutter by DM			
Atrial_Fibrillation_Flutter(Atrial Fibrillation/Flutter)	DM(DM)		
	1	2	Total
1	73<br>2.71<br>70.87<br>4.68	30<br>1.12<br>29.13<br>2.65	103<br>3.83
2	1486<br>55.24<br>57.44<br>95.32	1101<br>40.93<br>42.56<br>97.35	2587<br>96.17
Total	1559<br>57.96	1131<br>42.04	2690<br>100.00
Frequency Missing = 1			

The FREQ Procedure

Statistics for Table of Atrial_Fibrillation_Flutter by DM

Statistic	DF	Value	Prob
Chi-Square	1	7.3351	0.0068
Likelihood Ratio Chi-Square	1	7.6341	0.0057
Continuity Adj. Chi-Square	1	6.7942	0.0091
Mantel-Haenszel Chi-Square	1	7.3324	0.0068
Phi Coefficient		0.0522	
Contingency Coefficient		0.0521	
Cramer's V		0.0522	

Fisher's Exact Test	
Cell (1,1) Frequency (F)	73
Left-sided Pr <= F	0.9979
Right-sided Pr >= F	0.0040
Table Probability (P)	0.0019
Two-sided Pr <= P	0.0079

Sample Size = 2690
Frequency Missing = 1

Frequency<br>Percent<br>Row Pct<br>Col Pct

Table of Atrial_Fibrillation_Flutter by HTN			
	HTN(HTN)		
Atrial_Fibrillation_Flutter(Atrial Fibrillation/Flutter)	1	2	Total
1	76<br>2.83<br>73.79<br>4.83	27<br>1.00<br>26.21<br>2.42	103<br>3.83
2	1498<br>55.69<br>57.90<br>95.17	1089<br>40.48<br>42.10<br>97.58	2587<br>96.17
Total	1574<br>58.51	1116<br>41.49	2690<br>100.00
Frequency Missing = 1			

The FREQ Procedure

Statistics for Table of Atrial_Fibrillation_Flutter by HTN

Statistic	DF	Value	Prob
Chi-Square	1	10.2920	0.0013
Likelihood Ratio Chi-Square	1	10.8547	0.0010
Continuity Adj. Chi-Square	1	9.6482	0.0019
Mantel-Haenszel Chi-Square	1	10.2882	0.0013
Phi Coefficient		0.0619	
Contingency Coefficient		0.0617	
Cramer's V		0.0619	

Fisher's Exact Test	
Cell (1,1) Frequency (F)	76
Left-sided Pr <= F	0.9997
Right-sided Pr >= F	0.0007
Table Probability (P)	0.0004
Two-sided Pr <= P	0.0015

Sample Size = 2690
Frequency Missing = 1

Frequency<br>Percent<br>Row Pct<br>Col Pct

Table of Atrial_Fibrillation_Flutter by Hypercholestrolemia			
	Hypercholestrolemia(Hypercholestrolemia)		
Atrial_Fibrillation_Flutter(Atrial Fibrillation/Flutter)	1	2	Total
1	49<br>1.82<br>47.57<br>4.67	54<br>2.01<br>52.43<br>3.29	103<br>3.83
2	1000<br>37.17<br>38.65<br>95.33	1587<br>59.00<br>61.35<br>96.71	2587<br>96.17
Total	1049<br>39.00	1641<br>61.00	2690<br>100.00
Frequency Missing = 1			

The FREQ Procedure

Statistics for Table of Atrial_Fibrillation_Flutter by Hypercholestrolemia

Statistic	DF	Value	Prob
Chi-Square	1	3.3116	0.0688
Likelihood Ratio Chi-Square	1	3.2458	0.0716
Continuity Adj. Chi-Square	1	2.9473	0.0860
Mantel-Haenszel Chi-Square	1	3.3104	0.0688
Phi Coefficient		0.0351	
Contingency Coefficient		0.0351	
Cramer's V		0.0351	

Fisher's Exact Test	
Cell (1,1) Frequency (F)	49
Left-sided Pr <= F	0.9718
Right-sided Pr >= F	0.0440
Table Probability (P)	0.0158
Two-sided Pr <= P	0.0796

Sample Size = 2690
Frequency Missing = 1

Frequency
Percent
Row Pct
Col Pct

Table of Atrial_Fibrillation_Flutter by Current_or_ex_smoking			
Atrial_Fibrillation_Flutter(Atrial Fibrillation/Flutter)	Current_or_ex_smoking(Current or ex-smoking)		
	1	2	Total
1	39<br>1.45<br>37.86<br>3.34	64<br>2.38<br>62.14<br>4.20	103<br>3.83
2	1128<br>41.93<br>43.60<br>96.66	1459<br>54.24<br>56.40<br>95.80	2587<br>96.17
Total	1167<br>43.38	1523<br>56.62	2690<br>100.00
Frequency Missing = 1			

The FREQ Procedure

Statistics for Table of Atrial_Fibrillation_Flutter by Current_or_ex_smoking

Statistic	DF	Value	Prob
Chi-Square	1	1.3281	0.2491
Likelihood Ratio Chi-Square	1	1.3436	0.2464
Continuity Adj. Chi-Square	1	1.1047	0.2932
Mantel-Haenszel Chi-Square	1	1.3276	0.2492
Phi Coefficient		-0.0222	
Contingency Coefficient		0.0222	
Cramer's V		-0.0222	

Fisher's Exact Test	
Cell (1,1) Frequency (F)	39
Left-sided Pr <= F	0.1465
Right-sided Pr >= F	0.8956
Table Probability (P)	0.0421
Two-sided Pr <= P	0.2659

Sample Size = 2690
Frequency Missing = 1

Frequency<br>Percent<br>Row Pct<br>Col Pct

Table of Atrial_Fibrillation_Flutter by Chief_complaint						
Atrial_Fibrillation_Flutter(Atrial Fibrillation/Flutter)	Chief_complaint(Chief complaint)					
	1	2	3	4	5	Total
1	62<br>2.30<br>60.19<br>2.65	26<br>0.97<br>25.24<br>14.77	10<br>0.37<br>9.71<br>7.87	2<br>0.07<br>1.94<br>14.29	3<br>0.11<br>2.91<br>10.34	103<br>3.83
2	2282<br>84.83<br>88.21<br>97.35	150<br>5.58<br>5.80<br>85.23	117<br>4.35<br>4.52<br>92.13	12<br>0.45<br>0.46<br>85.71	26<br>0.97<br>1.01<br>89.66	2587<br>96.17
Total	2344<br>87.14	176<br>6.54	127<br>4.72	14<br>0.52	29<br>1.08	2690<br>100.00
Frequency Missing = 1						

The FREQ Procedure

Statistics for Table of Atrial_Fibrillation_Flutter by Chief_complaint

Statistic	DF	Value	Prob
Chi-Square	4	79.3083	<.0001
Likelihood Ratio Chi-Square	4	53.1252	<.0001
Mantel-Haenszel Chi-Square	1	40.7867	<.0001
Phi Coefficient		0.1717	
Contingency Coefficient		0.1692	
Cramer's V		0.1717	
WARNING: 30% of the cells have expected counts less than 5. Chi-Square may not be a valid test.			

Sample Size = 2690
Frequency Missing = 1

Frequency
Percent
Row Pct
Col Pct

Table of Atrial_Fibrillation_Flutter by _1st_medical_contact					
Atrial_Fibrillation_Flutter(Atrial Fibrillation/Flutter)	_1st_medical_contact(1st medical contact)				
	1	2	3	4	Total
1	10<br>1.60<br>38.46<br>7.52	12<br>1.92<br>46.15<br>3.31	0<br>0.00<br>0.00<br>0.00	4<br>0.64<br>15.38<br>3.08	26<br>4.15
2	123<br>19.65<br>20.50<br>92.48	350<br>55.91<br>58.33<br>96.69	1<br>0.16<br>0.17<br>100.00	126<br>20.13<br>21.00<br>96.92	600<br>95.85
Total	133<br>21.25	362<br>57.83	1<br>0.16	130<br>20.77	626<br>100.00
Frequency Missing = 2065					

The FREQ Procedure

Statistics for Table of Atrial_Fibrillation_Flutter by _1st_medical_contact

Statistic	DF	Value	Prob
Chi-Square	3	4.8451	0.1835
Likelihood Ratio Chi-Square	3	4.2612	0.2346
Mantel-Haenszel Chi-Square	1	2.1383	0.1437
Phi Coefficient		0.0880	
Contingency Coefficient		0.0876	
Cramer's V		0.0880	
WARNING: 25% of the cells have expected counts less than 5. Chi-Square may not be a valid test.			

Sample Size = 626
Frequency Missing = 2065

WARNING: 77% of the data are missing.

Frequency<br>Percent<br>Row Pct<br>Col Pct

Table of Atrial_Fibrillation_Flutter by Transferred_by_EMS_e_g__Red_Cres			
Atrial_Fibrillation_Flutter(Atrial Fibrillation/Flutter)	Transferred_by_EMS_e_g__Red_Cres(Transferred by EMS e.g. Red Crescent or Red Cross?)		
	1	2	Total
1	18<br>3.06<br>52.94<br>7.86	16<br>2.72<br>47.06<br>4.46	34<br>5.78
2	211<br>35.88<br>38.09<br>92.14	343<br>58.33<br>61.91<br>95.54	554<br>94.22
Total	229<br>38.95	359<br>61.05	588<br>100.00
Frequency Missing = 2103			

The FREQ Procedure

Statistics for Table of Atrial_Fibrillation_Flutter by Transferred_by_EMS_e_g__Red_Cres

Statistic	DF	Value	Prob
Chi-Square	1	2.9727	0.0847
Likelihood Ratio Chi-Square	1	2.8932	0.0890
Continuity Adj. Chi-Square	1	2.3808	0.1228
Mantel-Haenszel Chi-Square	1	2.9677	0.0849
Phi Coefficient		0.0711	
Contingency Coefficient		0.0709	
Cramer's V		0.0711	

Fisher's Exact Test	
Cell (1,1) Frequency (F)	18
Left-sided Pr <= F	0.9703
Right-sided Pr >= F	0.0628
Table Probability (P)	0.0331
Two-sided Pr <= P	0.1027

Sample Size = 588
Frequency Missing = 2103

WARNING: 78% of the data are missing.

Frequency<br>Percent<br>Row Pct<br>Col Pct

Table of Atrial_Fibrillation_Flutter by Cardiac_arrest			
Atrial_Fibrillation_Flutter(Atrial Fibrillation/Flutter)	Cardiac_arrest(Cardiac arrest)		
	1	2	Total
1	6<br>0.22<br>5.83<br>8.11	97<br>3.61<br>94.17<br>3.71	103<br>3.83
2	68<br>2.53<br>2.63<br>91.89	2519<br>93.64<br>97.37<br>96.29	2587<br>96.17
Total	74<br>2.75	2616<br>97.25	2690<br>100.00
Frequency Missing = 1			

The FREQ Procedure

Statistics for Table of Atrial_Fibrillation_Flutter by Cardiac_arrest

Statistic	DF	Value	Prob
Chi-Square	1	3.7838	0.0518
Likelihood Ratio Chi-Square	1	2.9182	0.0876
Continuity Adj. Chi-Square	1	2.6832	0.1014
Mantel-Haenszel Chi-Square	1	3.7824	0.0518
Phi Coefficient		0.0375	
Contingency Coefficient		0.0375	
Cramer's V		0.0375	
WARNING: 25% of the cells have expected counts less than 5. Chi-Square may not be a valid test.			

Fisher's Exact Test	
Cell (1,1) Frequency (F)	6
Left-sided Pr <= F	0.9785
Right-sided Pr >= F	0.0616
Table Probability (P)	0.0401
Two-sided Pr <= P	0.0616

Sample Size = 2690
Frequency Missing = 1

Frequency
Percent
Row Pct
Col Pct

Table of Atrial_Fibrillation_Flutter by CHF_Killip_Class					
Atrial_Fibrillation_Flutter(Atrial Fibrillation/Flutter)	CHF_Killip_Class(CHF Killip Class)				
	1	2	3	4	Total
1	59<br>2.19<br>57.28<br>2.50	30<br>1.12<br>29.13<br>15.38	9<br>0.33<br>8.74<br>9.38	5<br>0.19<br>4.85<br>12.20	103<br>3.83
2	2299<br>85.46<br>88.87<br>97.50	165<br>6.13<br>6.38<br>84.62	87<br>3.23<br>3.36<br>90.63	36<br>1.34<br>1.39<br>87.80	2587<br>96.17
Total	2358<br>87.66	195<br>7.25	96<br>3.57	41<br>1.52	2690<br>100.00
Frequency Missing = 1					

The FREQ Procedure

Statistics for Table of Atrial_Fibrillation_Flutter by CHF_Killip_Class

Statistic	DF	Value	Prob
Chi-Square	3	97.7974	<.0001
Likelihood Ratio Chi-Square	3	64.8168	<.0001
Mantel-Haenszel Chi-Square	1	60.2621	<.0001
Phi Coefficient		0.1907	
Contingency Coefficient		0.1873	
Cramer's V		0.1907	
WARNING: 25% of the cells have expected counts less than 5. Chi-Square may not be a valid test.			

Sample Size = 2690
Frequency Missing = 1

Frequency<br>Percent<br>Row Pct<br>Col Pct

Table of Atrial_Fibrillation_Flutter by Echo_Options

Atrial_Fibrillation_Flutter(Atrial Fibrillation/Flutter)	Echo_Options(Echo-Options)				
	1	2	3	4	Total
1	29<br>1.22<br>28.71<br>3.06	28<br>1.17<br>27.72<br>3.64	23<br>0.96<br>22.77<br>5.00	21<br>0.88<br>20.79<br>10.05	101<br>4.23
2	918<br>38.49<br>40.19<br>96.94	741<br>31.07<br>32.44<br>96.36	437<br>18.32<br>19.13<br>95.00	188<br>7.88<br>8.23<br>89.95	2284<br>95.77
Total	947<br>39.71	769<br>32.24	460<br>19.29	209<br>8.76	2385<br>100.00
Frequency Missing = 306					

The FREQ Procedure

Statistics for Table of Atrial_Fibrillation_Flutter by Echo_Options

Statistic	DF	Value	Prob
Chi-Square	3	21.9573	<.0001
Likelihood Ratio Chi-Square	3	17.6135	0.0005
Mantel-Haenszel Chi-Square	1	16.6619	<.0001
Phi Coefficient		0.0960	
Contingency Coefficient		0.0955	
Cramer's V		0.0960	

Sample Size = 2385
Frequency Missing = 306

WARNING: 11% of the data are missing.

Frequency<br>Percent<br>Row Pct<br>Col Pct

Table of Atrial_Fibrillation_Flutter by Elective_coronary_angiogram			
Atrial_Fibrillation_Flutter(Atrial Fibrillation/Flutter)	Elective_coronary_angiogram(Elective coronary angiogram)		
	1	2	Total
1	11<br>0.63<br>15.71<br>2.64	59<br>3.39<br>84.29<br>4.46	70<br>4.02
2	405<br>23.28<br>24.25<br>97.36	1265<br>72.70<br>75.75<br>95.54	1670<br>95.98
Total	416<br>23.91	1324<br>76.09	1740<br>100.00
Frequency Missing = 951			

The FREQ Procedure

Statistics for Table of Atrial_Fibrillation_Flutter by Elective_coronary_angiogram

Statistic	DF	Value	Prob
Chi-Square	1	2.6916	0.1009
Likelihood Ratio Chi-Square	1	2.9436	0.0862
Continuity Adj. Chi-Square	1	2.2428	0.1342
Mantel-Haenszel Chi-Square	1	2.6901	0.1010
Phi Coefficient		-0.0393	
Contingency Coefficient		0.0393	
Cramer's V		-0.0393	

Fisher's Exact Test	
Cell (1,1) Frequency (F)	11
Left-sided Pr <= F	0.0627
Right-sided Pr >= F	0.9676
Table Probability (P)	0.0303
Two-sided Pr <= P	0.1156

Sample Size = 1740
Frequency Missing = 951

WARNING: 35% of the data are missing.

Frequency<br>Percent<br>Row Pct<br>Col Pct

Table of Atrial_Fibrillation_Flutter by Arterial_access				
Atrial_Fibrillation_Flutter(Atrial Fibrillation/Flutter)	Arterial_access(Arterial access)			
	1	2	3	Total
1	13<br>1.11<br>28.89<br>4.61	32<br>2.74<br>71.11<br>3.61	0<br>0.00<br>0.00<br>0.00	45<br>3.85
2	269<br>23.01<br>23.93<br>95.39	854<br>73.05<br>75.98<br>96.39	1<br>0.09<br>0.09<br>100.00	1124<br>96.15
Total	282<br>24.12	886<br>75.79	1<br>0.09	1169<br>100.00
Frequency Missing = 1522				

The FREQ Procedure

Statistics for Table of Atrial_Fibrillation_Flutter by Arterial_access

Statistic	DF	Value	Prob
Chi-Square	2	0.6159	0.7349
Likelihood Ratio Chi-Square	2	0.6318	0.7291
Mantel-Haenszel Chi-Square	1	0.5971	0.4397
Phi Coefficient		0.0230	
Contingency Coefficient		0.0229	
Cramer's V		0.0230	
WARNING: 33% of the cells have expected counts less than 5. Chi-Square may not be a valid test.			

Sample Size = 1169
Frequency Missing = 1522

WARNING: 57% of the data are missing.

Frequency<br>Percent<br>Row Pct<br>Col Pct

Table of Atrial_Fibrillation_Flutter by Arterial_access_1			
Atrial_Fibrillation_Flutter(Atrial Fibrillation/Flutter)	Arterial_access_1(Arterial access_1)		
	1	2	Total
1	3<br>0.72<br>27.27<br>3.09	8<br>1.92<br>72.73<br>2.51	11<br>2.64
2	94<br>22.60<br>23.21<br>96.91	311<br>74.76<br>76.79<br>97.49	405<br>97.36
Total	97<br>23.32	319<br>76.68	416<br>100.00
Frequency Missing = 2275			

The FREQ Procedure

Statistics for Table of Atrial_Fibrillation_Flutter by Arterial_access_1

Statistic	DF	Value	Prob
Chi-Square	1	0.0989	0.7532
Likelihood Ratio Chi-Square	1	0.0954	0.7574
Continuity Adj. Chi-Square	1	0.0000	1.0000
Mantel-Haenszel Chi-Square	1	0.0986	0.7535
Phi Coefficient		0.0154	
Contingency Coefficient		0.0154	
Cramer's V		0.0154	
WARNING: 25% of the cells have expected counts less than 5. Chi-Square may not be a valid test.			

Fisher's Exact Test	
Cell (1,1) Frequency (F)	3
Left-sided Pr <= F	0.7606
Right-sided Pr >= F	0.4929
Table Probability (P)	0.2535
Two-sided Pr <= P	0.7233

Sample Size = 416
Frequency Missing = 2275

WARNING: 85% of the data are missing.

The TTEST Procedure

Variable: Age (Age)

Atrial_Fibrillation_Flutter	Method	N	Mean	Std Dev	Std Err	Minimum	Maximum
**1**		103	63.6796	12.5840	1.2399	32.0000	91.0000
**2**		2587	56.9030	12.3566	0.2429	19.0000	103.0
**Diff (1-2)**	**Pooled**		6.7766	12.3653	1.2424		
**Diff (1-2)**	**Satterthwaite**		6.7766		1.2635		

Atrial_Fibrillation_Flutter	Method	Mean	95% CL Mean		Std Dev	95% CL Std Dev	
**1**		63.6796	61.2202	66.1390	12.5840	11.0688	14.5837
**2**		56.9030	56.4266	57.3794	12.3566	12.0288	12.7028
**Diff (1-2)**	**Pooled**	6.7766	4.3405	9.2128	12.3653	12.0434	12.7049
**Diff (1-2)**	**Satterthwaite**	6.7766	4.2726	9.2806			

Method	Variances	DF	t Value	Pr > |t|
**Pooled**	Equal	2688	5.45	<.0001
**Satterthwaite**	Unequal	109.98	5.36	<.0001

Equality of Variances				
**Method**	**Num DF**	**Den DF**	**F Value**	**Pr > F**
**Folded F**	102	2586	1.04	0.7636

The TTEST Procedure

Variable: Age (Age)

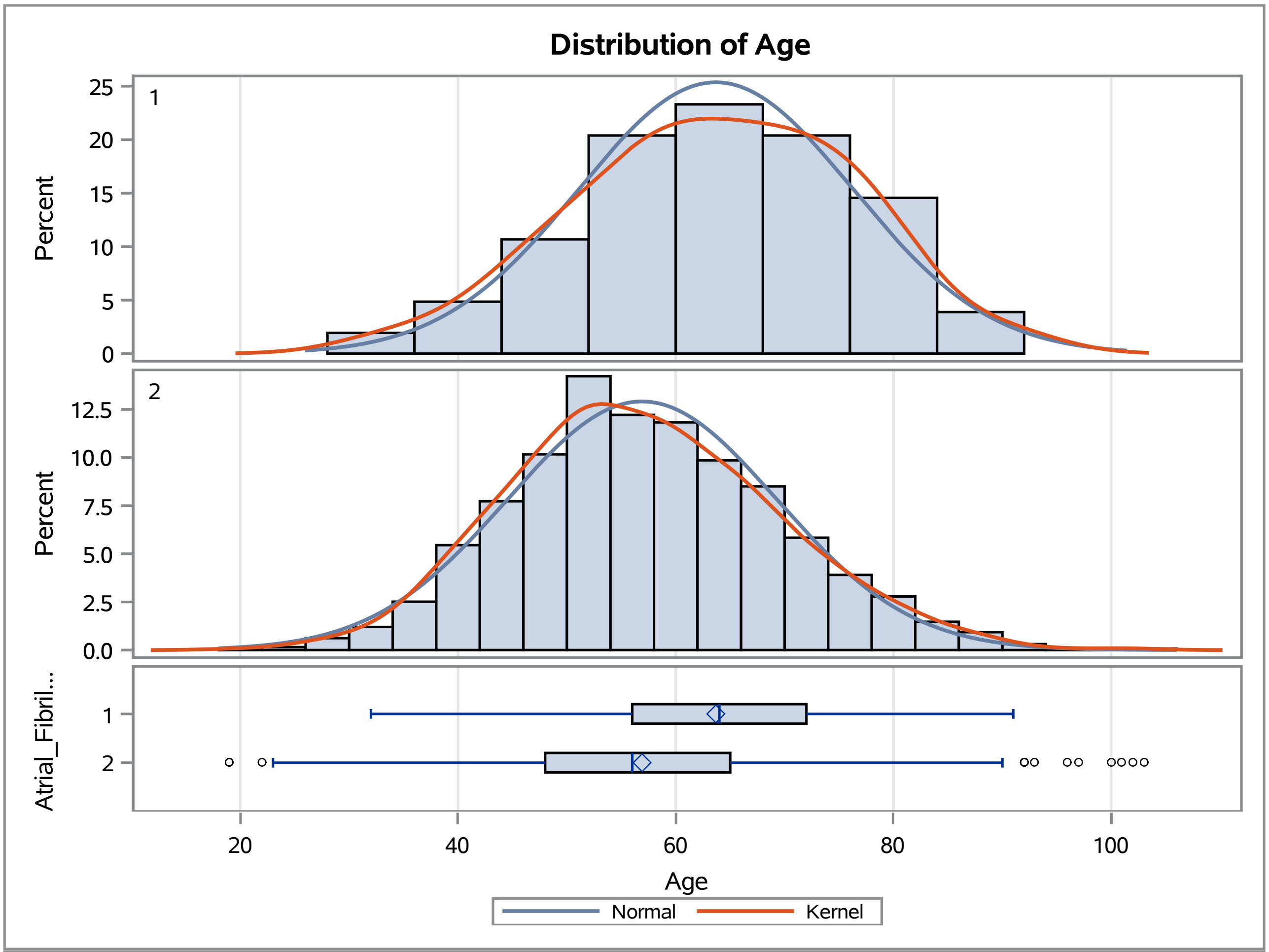

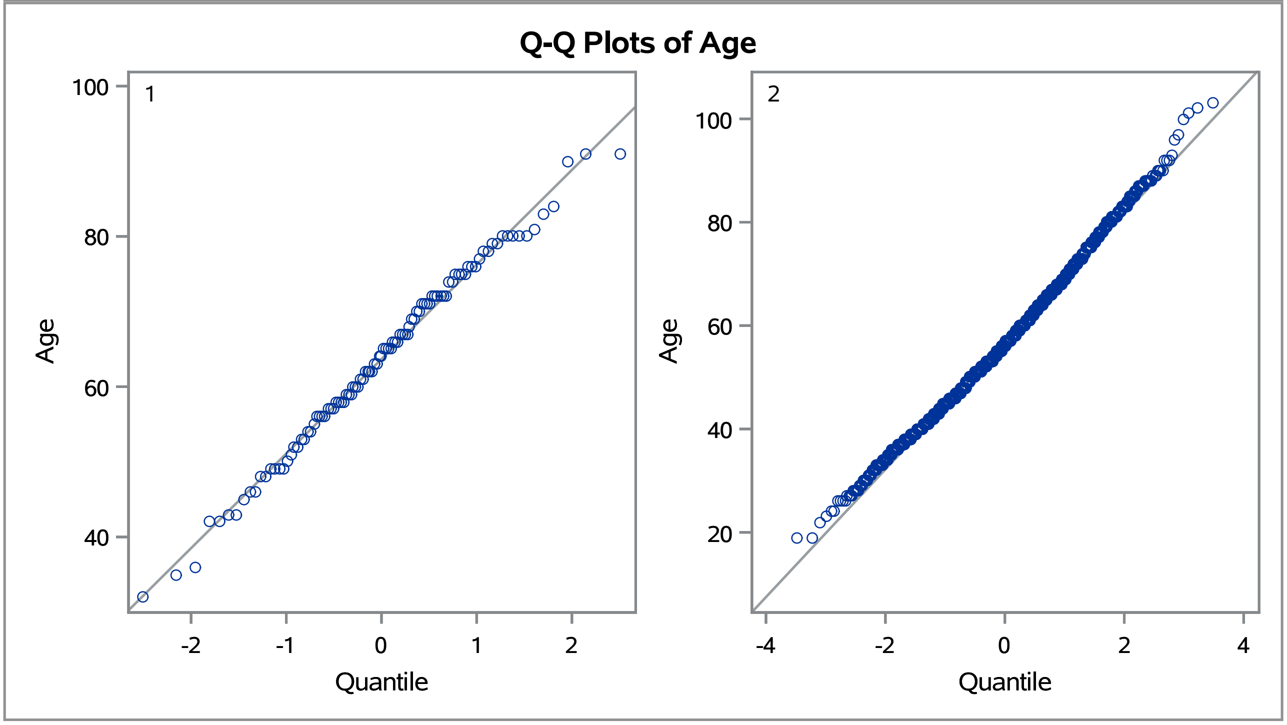

The TTEST Procedure

Variable: BMI (BMI)

Variable: BMI (BMI)

Atrial_Fibrillation_Flutter	Method	N	Mean	Std Dev	Std Err	Minimum	Maximum
1		103	29.5186	5.2609	0.5184	18.0500	44.0200
2		2587	28.4926	4.7506	0.0934	15.1600	75.0000
Diff (1-2)	Pooled		1.0261	4.7710	0.4794		
Diff (1-2)	Satterthwaite		1.0261		0.5267		

Atrial_Fibrillation_Flutter	Method	Mean	95% CL Mean		Std Dev	95% CL Std Dev	
1		29.5186	28.4905	30.5468	5.2609	4.6274	6.0969
2		28.4926	28.3094	28.6757	4.7506	4.6246	4.8837
Diff (1-2)	Pooled	1.0261	0.0861	1.9660	4.7710	4.6468	4.9020
Diff (1-2)	Satterthwaite	1.0261	-0.0179	2.0700			

Method	Variances	DF	t Value	Pr > |t|
Pooled	Equal	2688	2.14	0.0324
Satterthwaite	Unequal	108.73	1.95	0.0540

Equality of Variances				
Method	Num DF	Den DF	F Value	Pr > F
Folded F	102	2586	1.23	0.1289

The TTEST Procedure

Variable: BMI (BMI)

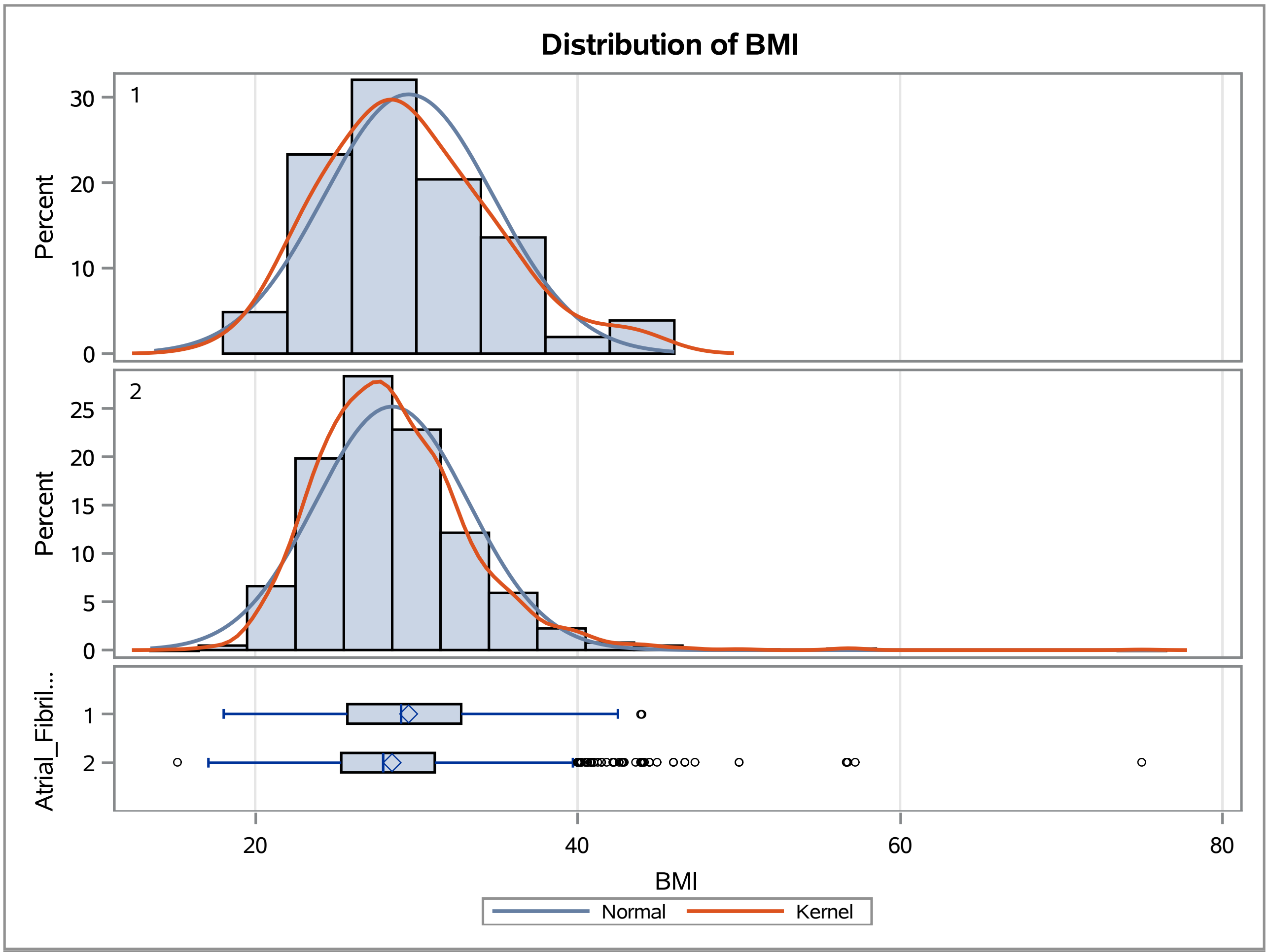

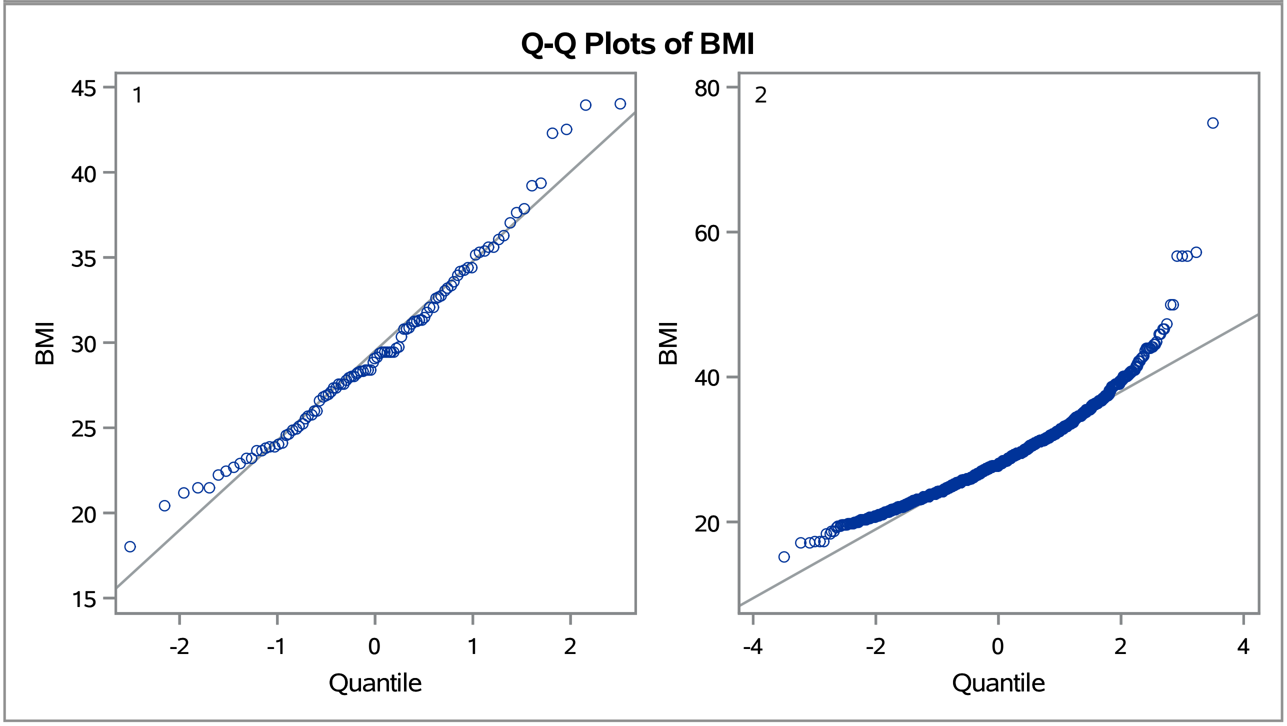

The TTEST Procedure

Variable: HR_bpm_ (HR(bpm))

Variable: HR_bpm_ (HR(bpm))

Atrial_Fibrillation_Flutter	Method	N	Mean	Std Dev	Std Err	Minimum	Maximum
1		103	100.1	24.2253	2.3870	52.0000	173.0
2		2586	83.5278	17.1448	0.3371	10.0000	230.0
Diff (1-2)	Pooled		16.6081	17.4661	1.7549		
Diff (1-2)	Satterthwaite		16.6081		2.4107		

Atrial_Fibrillation_Flutter	Method	Mean	95% CL Mean		Std Dev	95% CL Std Dev	
1		100.1	95.4013	104.9	24.2253	21.3083	28.0748
2		83.5278	82.8667	84.1889	17.1448	16.6900	17.6253
Diff (1-2)	Pooled	16.6081	13.1669	20.0492	17.4661	17.0113	17.9459
Diff (1-2)	Satterthwaite	16.6081	11.8287	21.3874			

Method	Variances	DF	t Value	Pr > |t|
Pooled	Equal	2687	9.46	<.0001
Satterthwaite	Unequal	106.11	6.89	<.0001

Equality of Variances				
Method	Num DF	Den DF	F Value	Pr > F
Folded F	102	2585	2.00	<.0001

The TTEST Procedure

Variable: HR_bpm_ (HR(bpm))

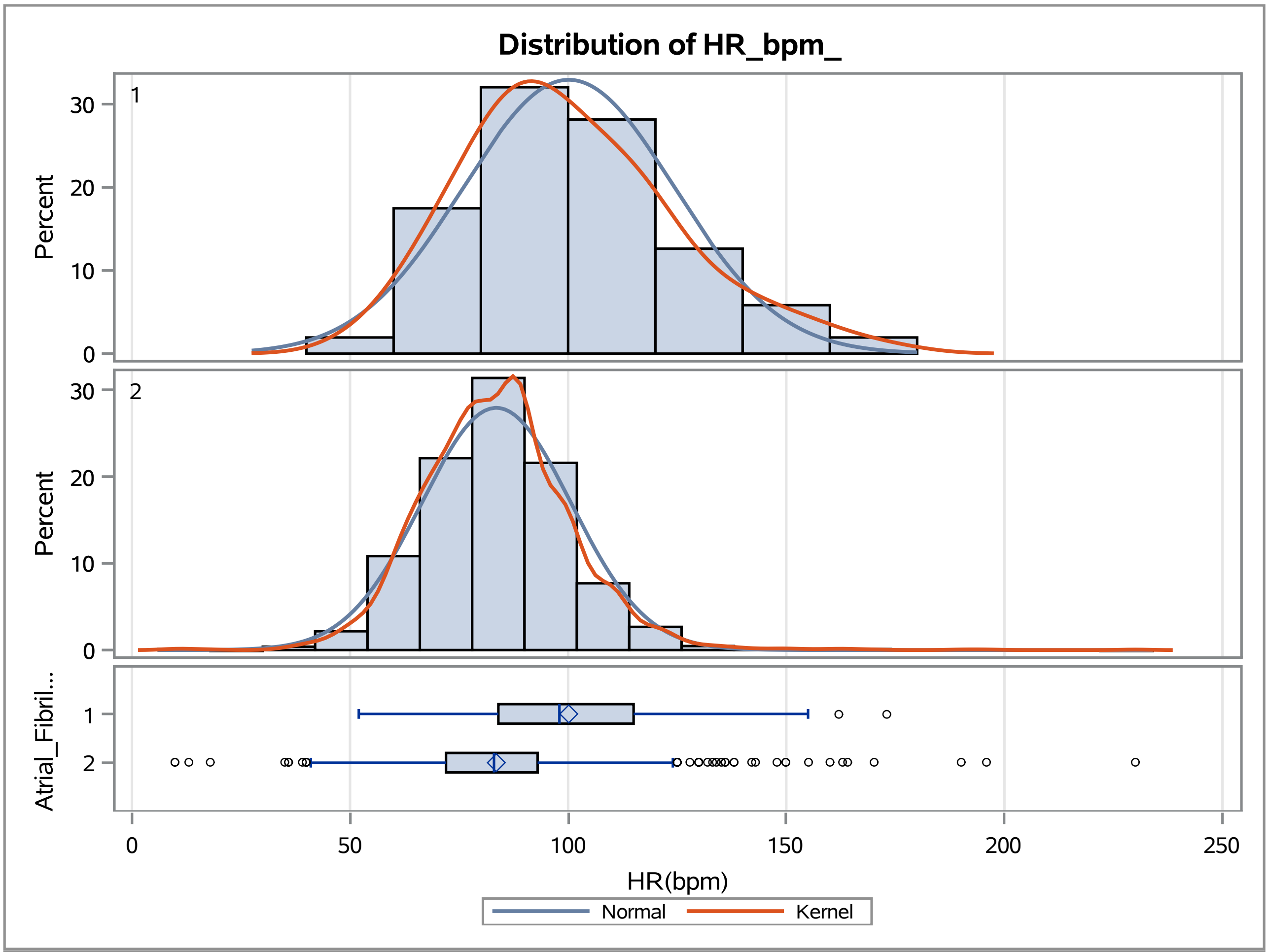

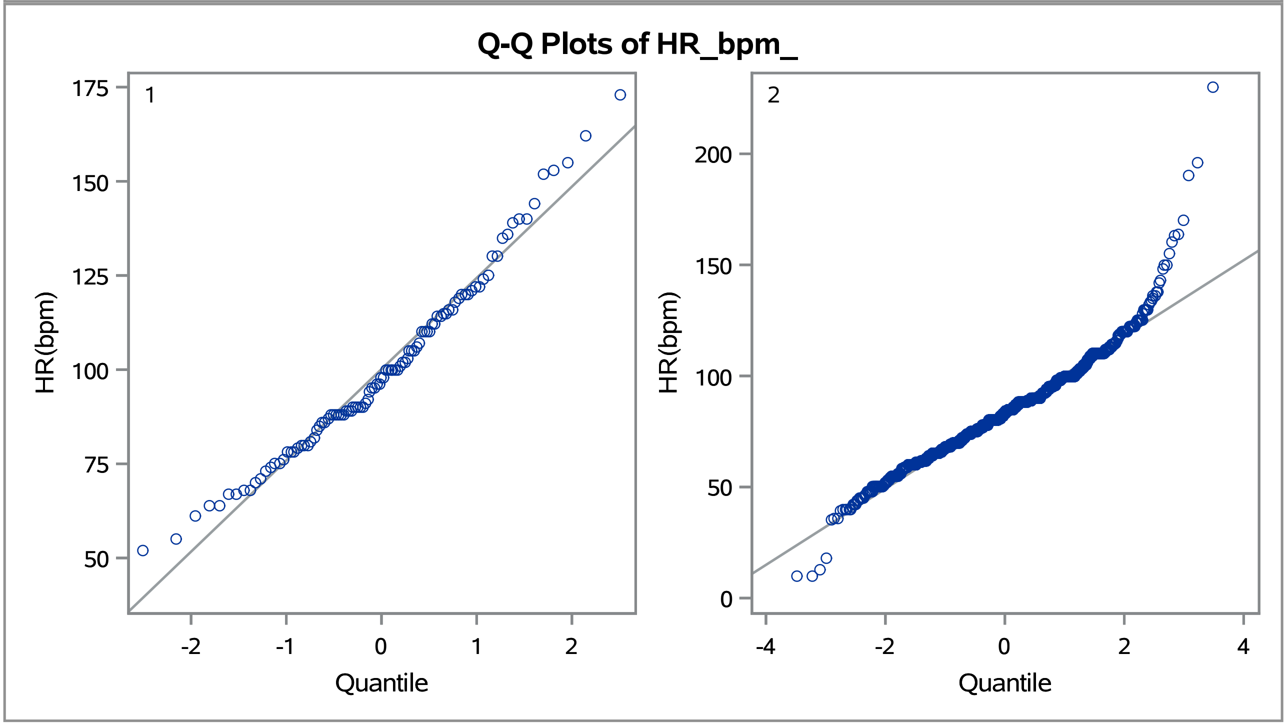

The TTEST Procedure

Variable: SBP_mmgH_ (SBP(mmgH))

Variable: SBP_mmgH_ (SBP(mmgH))

Atrial_Fibrillation_Flutter	Method	N	Mean	Std Dev	Std Err	Minimum	Maximum
1		103	133.7	25.4856	2.5112	70.0000	190.0
2		2586	134.9	25.4580	0.5006	40.0000	244.0
Diff (1-2)	Pooled		-1.2539	25.4591	2.5580		
Diff (1-2)	Satterthwaite		-1.2539		2.5606		

Atrial_Fibrillation_Flutter	Method	Mean	95% CL Mean		Std Dev	95% CL Std Dev	
1		133.7	128.7	138.7	25.4856	22.4169	29.5355
2		134.9	133.9	135.9	25.4580	24.7827	26.1715
Diff (1-2)	Pooled	-1.2539	-6.2698	3.7620	25.4591	24.7963	26.1585
Diff (1-2)	Satterthwaite	-1.2539	-6.3283	3.8204			

Method	Variances	DF	t Value	Pr > |t|
Pooled	Equal	2687	-0.49	0.6240
Satterthwaite	Unequal	110.26	-0.49	0.6253

Equality of Variances				
Method	Num DF	Den DF	F Value	Pr > F
Folded F	102	2585	1.00	0.9528

The TTEST Procedure

Variable: SBP_mmgH_ (SBP(mmgH))

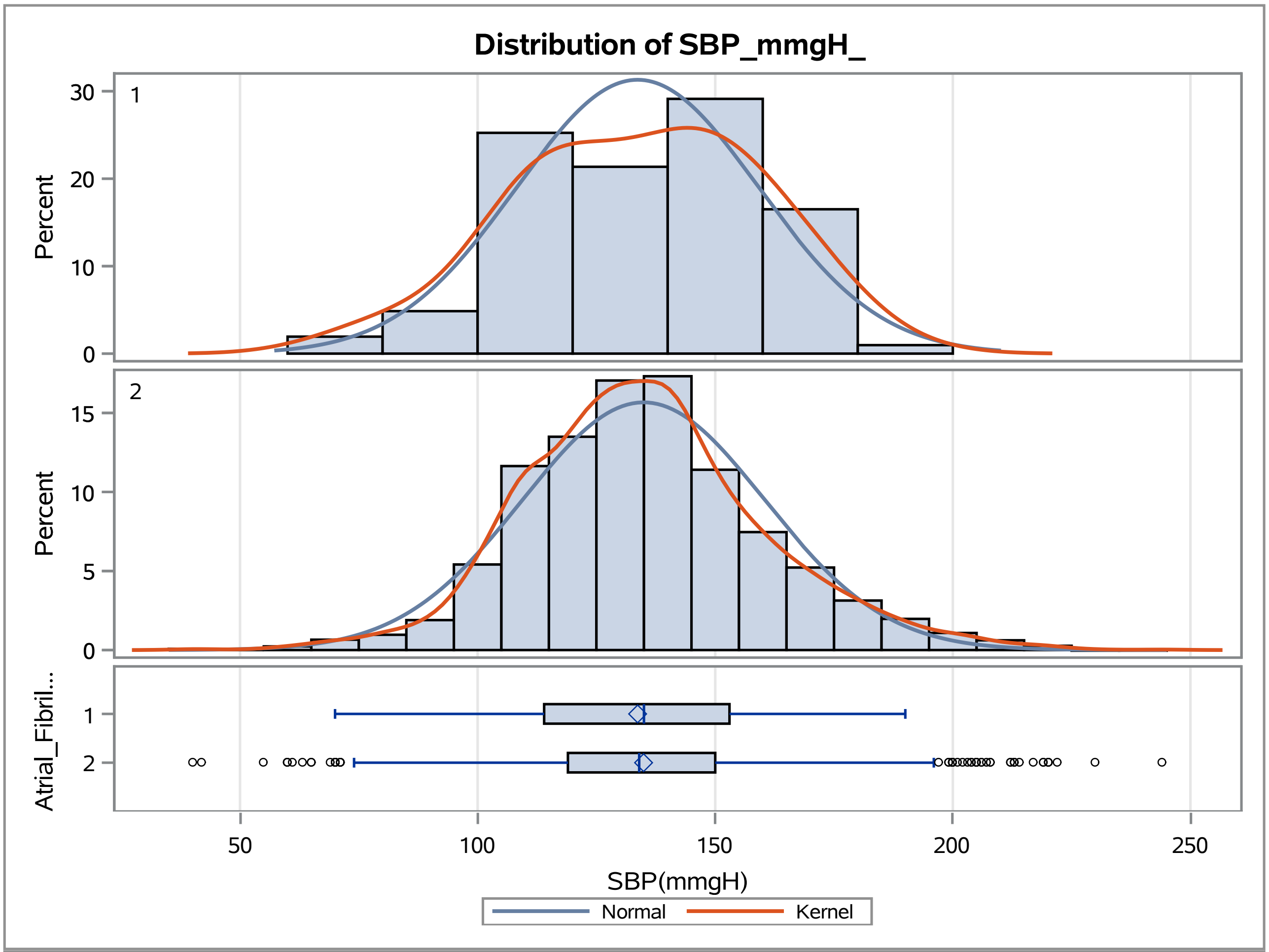

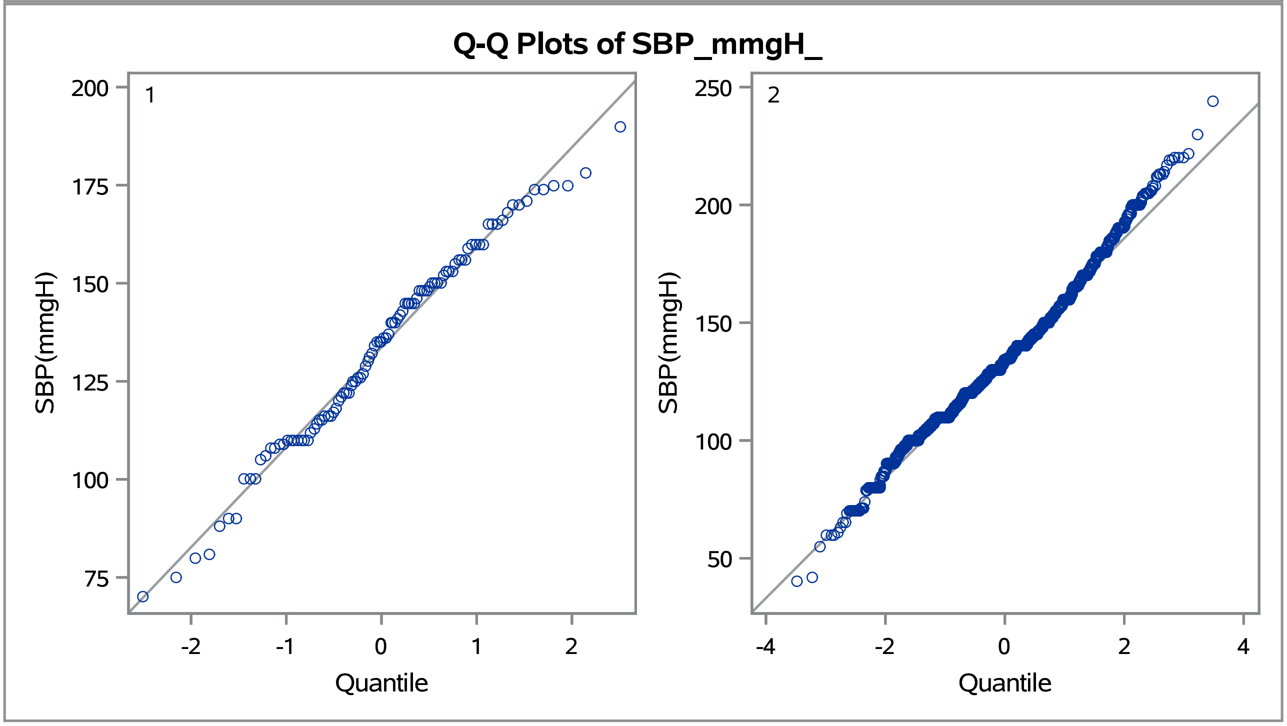

The FREQ Procedure

Atrial Fibrillation/Flutter				
**Atrial_Fibrillation_Flutter**	**Frequency**	**Percent**	**Cumulative Frequency**	**Cumulative Percent**
**1**	103	3.83	103	3.83
**2**	2587	96.17	2690	100.00
**Frequency Missing = 1**				

The LOGISTIC Procedure

Model Information		
**Data Set**	WAEL.STARS4	
**Response Variable**	Atrial_Fibrillation_Flutter	Atrial Fibrillation/Flutter
**Number of Response Levels**	2	
**Model**	binary logit	
**Optimization Technique**	Fisher's scoring	

**Number of Observations Read**	2691
**Number of Observations Used**	2689

Response Profile		
**Ordered Value**	**Atrial_Fibrillation_Flutter**	**Total Frequency**
**1**	1	103
**2**	2	2586

Probability modeled is Atrial_Fibrillation_Flutter='1'.

Note: 2 observations were deleted due to missing values for the response or explanatory variables.

Class Level Information				
**Class**	**Value**	**Design Variables**		
**Gender**	**1**	0		
	**2**	1		
**Nationality**	**1**	0		
	**2**	1		
**History_of_angina**	**1**	0		
	**2**	1		
**History_of_heart_failure**	**1**	0		
	**2**	1		
**History_of_stroke**	**1**	0		
	**2**	1		
**History_of_chronic_renal_failure**	**1**	0		
	**2**	1		
**DM**	**1**	0		
	**2**	1		
**HTN**	**1**	0		
	**2**	1		
**CHF_Killip_Class**	**1**	0	0	0

The LOGISTIC Procedure

Class Level Information				
Class	Value	Design Variables		
	2	1	0	0
	3	0	1	0
	4	0	0	1

Model Convergence Status
Convergence criterion (GCONV=1E-8) satisfied.

Model Fit Statistics		
Criterion	Intercept Only	Intercept and Covariates
AIC	876.016	749.849
SC	881.913	838.303
-2 Log L	874.016	719.849

Testing Global Null Hypothesis: BETA=0			
Test	Chi-Square	DF	Pr > ChiSq
Likelihood Ratio	154.1665	14	<.0001
Score	203.0465	14	<.0001
Wald	145.0560	14	<.0001

Type 3 Analysis of Effects			
Effect	DF	Wald Chi-Square	Pr > ChiSq
Gender	1	0.1063	0.7444
Nationality	1	4.8017	0.0284
History_of_angina	1	0.0365	0.8484
History_of_heart_fai	1	3.3297	0.0680
History_of_stroke	1	2.3984	0.1215
History_of_chronic_r	1	0.5131	0.4738
DM	1	0.0448	0.8325
HTN	1	0.0911	0.7628
CHF_Killip_Class	3	28.6321	<.0001
Age	1	13.6234	0.0002
BMI	1	3.1152	0.0776
HR_bpm_	1	49.7794	<.0001

The LOGISTIC Procedure

Analysis of Maximum Likelihood Estimates						
Parameter		DF	Estimate	Standard Error	Wald Chi-Square	Pr > ChiSq
Intercept		1	-8.6958	1.1238	59.8738	<.0001
Gender	2	1	-0.0820	0.2514	0.1063	0.7444
Nationality	2	1	-0.6767	0.3088	4.8017	0.0284
History_of_angina	2	1	-0.0462	0.2417	0.0365	0.8484
History_of_heart_fai	2	1	-0.6242	0.3421	3.3297	0.0680
History_of_stroke	2	1	-0.5458	0.3524	2.3984	0.1215
History_of_chronic_r	2	1	0.2418	0.3375	0.5131	0.4738
DM	2	1	-0.0536	0.2536	0.0448	0.8325
HTN	2	1	-0.0817	0.2706	0.0911	0.7628
CHF_Killip_Class	2	1	1.4110	0.2750	26.3178	<.0001
CHF_Killip_Class	3	1	0.5067	0.4281	1.4011	0.2365
CHF_Killip_Class	4	1	1.3961	0.5776	5.8422	0.0156
Age		1	0.0332	0.00900	13.6234	0.0002
BMI		1	0.0362	0.0205	3.1152	0.0776
HR_bpm_		1	0.0354	0.00502	49.7794	<.0001

Odds Ratio Estimates			
Effect	Point Estimate	95% Wald Confidence Limits	
Gender 2 vs 1	0.921	0.563	1.508
Nationality 2 vs 1	0.508	0.277	0.931
History_of_angina 2 vs 1	0.955	0.595	1.534
History_of_heart_fai 2 vs 1	0.536	0.274	1.047
History_of_stroke 2 vs 1	0.579	0.290	1.156
History_of_chronic_r 2 vs 1	1.274	0.657	2.468
DM 2 vs 1	0.948	0.577	1.558
HTN 2 vs 1	0.922	0.542	1.566
CHF_Killip_Class 2 vs 1	4.100	2.391	7.029
CHF_Killip_Class 3 vs 1	1.660	0.717	3.841
CHF_Killip_Class 4 vs 1	4.040	1.302	12.531
Age	1.034	1.016	1.052
BMI	1.037	0.996	1.079
HR_bpm_	1.036	1.026	1.046

The LOGISTIC Procedure

Association of Predicted Probabilities and Observed Responses			
**Percent Concordant**	83.0	**Somers' D**	0.661
**Percent Discordant**	17.0	**Gamma**	0.661
**Percent Tied**	0.0	**Tau-a**	0.049
**Pairs**	266358	**c**	0.830

*1/12 follow-up 224: 1-month re-admission vs withina month??, cause 1: cardiac, 2:non-cardiac, 3: unknown, ;

The FREQ Procedure

Frequency<br>Percent<br>Row Pct<br>Col Pct

Table of STEMI_NSTEMI by the_P_T__is			
	the_P_T__is(the P.T. is)		
STEMI_NSTEMI(STEMI/NSTEMI)	1	2	Total
1	1151<br>43.85<br>90.20<br>47.92	125<br>4.76<br>9.80<br>56.05	1276<br>48.61
2	1251<br>47.66<br>92.74<br>52.08	98<br>3.73<br>7.26<br>43.95	1349<br>51.39
Total	2402<br>91.50	223<br>8.50	2625<br>100.00
Frequency Missing = 66			

Statistics for Table of STEMI_NSTEMI by the_P_T__is

Statistic	DF	Value	Prob
Chi-Square	1	5.4063	0.0201
Likelihood Ratio Chi-Square	1	5.4111	0.0200
Continuity Adj. Chi-Square	1	5.0856	0.0241
Mantel-Haenszel Chi-Square	1	5.4043	0.0201
Phi Coefficient		-0.0454	
Contingency Coefficient		0.0453	
Cramer's V		-0.0454	

Fisher's Exact Test	
Cell (1,1) Frequency (F)	1151
Left-sided Pr <= F	0.0120
Right-sided Pr >= F	0.9917
Table Probability (P)	0.0038
Two-sided Pr <= P	0.0209

Sample Size = 2625
Frequency Missing = 66

*1/12 follow-up 224: 1-month re-admission vs withina month??, cause 1: cardiac, 2:non-cardiac, 3: unknown, ;

The FREQ Procedure

Frequency<br>Percent<br>Row Pct<br>Col Pct

Table of STEMI_NSTEMI by VAR224

	VAR224(1-month-re-admission)			
STEMI_NSTEMI(STEMI/NSTEMI)	1	2	3	Total
1	90<br>3.75<br>7.81<br>47.62	27<br>1.12<br>2.34<br>38.57	1035<br>43.07<br>89.84<br>48.27	1152<br>47.94
2	99<br>4.12<br>7.91<br>52.38	43<br>1.79<br>3.44<br>61.43	1109<br>46.15<br>88.65<br>51.73	1251<br>52.06
Total	189<br>7.87	70<br>2.91	2144<br>89.22	2403<br>100.00

Frequency Missing = 288

Statistics for Table of STEMI_NSTEMI by VAR224

Statistic	DF	Value	Prob
Chi-Square	2	2.5655	0.2773
Likelihood Ratio Chi-Square	2	2.5932	0.2735
Mantel-Haenszel Chi-Square	1	0.3258	0.5681
Phi Coefficient		0.0327	
Contingency Coefficient		0.0327	
Cramer's V		0.0327	

Sample Size = 2403
Frequency Missing = 288

WARNING: 11% of the data are missing.

Frequency<br>Percent<br>Row Pct<br>Col Pct

Table of STEMI_NSTEMI by readmission_within_30_days

	readmission_within_30_days(readmission within 30 days)		
STEMI_NSTEMI(STEMI/NSTEMI)	1	2	Total
1	60<br>31.75<br>66.67<br>50.00	30<br>15.87<br>33.33<br>43.48	90<br>47.62
2	60<br>31.75<br>60.61<br>50.00	39<br>20.63<br>39.39<br>56.52	99<br>52.38
Total	120<br>63.49	69<br>36.51	189<br>100.00

Frequency Missing = 2502

*1/12 follow-up 224: 1-month re-admission vs withina month??, cause 1: cardiac, 2:non-cardiac, 3: unknown, ;

The FREQ Procedure

Statistics for Table of STEMI_NSTEMI by readmission_within_30_days

Statistic	DF	Value	Prob
Chi-Square	1	0.7470	0.3874
Likelihood Ratio Chi-Square	1	0.7485	0.3869
Continuity Adj. Chi-Square	1	0.5085	0.4758
Mantel-Haenszel Chi-Square	1	0.7431	0.3887
Phi Coefficient		0.0629	
Contingency Coefficient		0.0627	
Cramer's V		0.0629	

Fisher's Exact Test	
Cell (1,1) Frequency (F)	60
Left-sided Pr <= F	0.8451
Right-sided Pr >= F	0.2381
Table Probability (P)	0.0832
Two-sided Pr <= P	0.4501

Sample Size = 189
Frequency Missing = 2502

WARNING: 93% of the data are missing.

Frequency<br>Percent<br>Row Pct<br>Col Pct

Table of STEMI_NSTEMI by cause				
	cause(cause)			
STEMI_NSTEMI(STEMI/NSTEMI)	1	2	3	Total
1	66<br>34.92<br>73.33<br>49.25	24<br>12.70<br>26.67<br>44.44	0<br>0.00<br>0.00<br>0.00	90<br>47.62
2	68<br>35.98<br>68.69<br>50.75	30<br>15.87<br>30.30<br>55.56	1<br>0.53<br>1.01<br>100.00	99<br>52.38
Total	134<br>70.90	54<br>28.57	1<br>0.53	189<br>100.00
Frequency Missing = 2502				

*1/12 follow-up 224: 1-month re-admission vs withina month??, cause 1: cardiac, 2:non-cardiac, 3: unknown, ;

The FREQ Procedure

Statistics for Table of STEMI_NSTEMI by cause

Statistic	DF	Value	Prob
Chi-Square	2	1.2708	0.5297
Likelihood Ratio Chi-Square	2	1.6555	0.4370
Mantel-Haenszel Chi-Square	1	0.6849	0.4079
Phi Coefficient		0.0820	
Contingency Coefficient		0.0817	
Cramer's V		0.0820	
WARNING: 33% of the cells have expected counts less than 5. Chi-Square may not be a valid test.			

Sample Size = 189
Frequency Missing = 2502

WARNING: 93% of the data are missing.

Frequency<br>Percent<br>Row Pct<br>Col Pct

Table of STEMI_NSTEMI by _1_Month_mortality			
	_1_Month_mortality(1-Month mortality)		
STEMI_NSTEMI(STEMI/NSTEMI)	1	2	Total
1	1143<br>47.59<br>99.30<br>47.86	8<br>0.33<br>0.70<br>57.14	1151<br>47.92
2	1245<br>51.83<br>99.52<br>52.14	6<br>0.25<br>0.48<br>42.86	1251<br>52.08
Total	2388<br>99.42	14<br>0.58	2402<br>100.00
Frequency Missing = 289			

*1/12 follow-up 224: 1-month re-admission vs withina month??, cause 1: cardiac, 2:non-cardiac, 3: unknown, ;

The FREQ Procedure

Statistics for Table of STEMI_NSTEMI by _1_Month_mortality

Statistic	DF	Value	Prob
Chi-Square	1	0.4801	0.4884
Likelihood Ratio Chi-Square	1	0.4804	0.4882
Continuity Adj. Chi-Square	1	0.1803	0.6711
Mantel-Haenszel Chi-Square	1	0.4799	0.4885
Phi Coefficient		-0.0141	
Contingency Coefficient		0.0141	
Cramer's V		-0.0141	

Fisher's Exact Test	
Cell (1,1) Frequency (F)	1143
Left-sided Pr <= F	0.3352
Right-sided Pr >= F	0.8316
Table Probability (P)	0.1669
Two-sided Pr <= P	0.5952

Sample Size = 2402
Frequency Missing = 289

WARNING: 11% of the data are missing.

Frequency<br>Percent<br>Row Pct<br>Col Pct

Table of STEMI_NSTEMI by death_within_30_days			
	death_within_30_days(death within 30 days)		
STEMI_NSTEMI(STEMI/NSTEMI)	1	2	Total
1	4<br>33.33<br>57.14<br>50.00	3<br>25.00<br>42.86<br>75.00	7<br>58.33
2	4<br>33.33<br>80.00<br>50.00	1<br>8.33<br>20.00<br>25.00	5<br>41.67
Total	8<br>66.67	4<br>33.33	12<br>100.00
Frequency Missing = 2679			

*1/12 follow-up 224: 1-month re-admission vs withina month??, cause 1: cardiac, 2:non-cardiac, 3: unknown, ;

The FREQ Procedure

Statistics for Table of STEMI_NSTEMI by death_within_30_days

Statistic	DF	Value	Prob
Chi-Square	1	0.6857	0.4076
Likelihood Ratio Chi-Square	1	0.7116	0.3989
Continuity Adj. Chi-Square	1	0.0429	0.8360
Mantel-Haenszel Chi-Square	1	0.6286	0.4279
Phi Coefficient		-0.2390	
Contingency Coefficient		0.2325	
Cramer's V		-0.2390	
WARNING: 100% of the cells have expected counts less than 5. Chi-Square may not be a valid test.			

Fisher's Exact Test	
Cell (1,1) Frequency (F)	4
Left-sided Pr <= F	0.4242
Right-sided Pr >= F	0.9293
Table Probability (P)	0.3535
Two-sided Pr <= P	0.5758

Sample Size = 12
Frequency Missing = 2679

WARNING: 100% of the data are missing.

*1/12 follow-up 224: 1-month re-admission vs withina month??, cause 1: cardiac, 2:non-cardiac, 3: unknown, ;

The FREQ Procedure

Geographical Region				
Geographical_Region	Frequency	Percent	Cumulative Frequency	Cumulative Percent
1	686	25.50	686	25.50
2	124	4.61	810	30.11
3	121	4.50	931	34.61
4	337	12.53	1268	47.14
5	60	2.23	1328	49.37
6	80	2.97	1408	52.34
7	426	15.84	1834	68.18
8	345	12.83	2179	81.00
9	3	0.11	2182	81.12
10	61	2.27	2243	83.38
12	75	2.79	2318	86.17
13	372	13.83	2690	100.00
Frequency Missing = 1				

HEALTH CARE SECTOR				
HEALTH_CARE_SECTOR	Frequency	Percent	Cumulative Frequency	Cumulative Percent
1	1706	63.42	1706	63.42
2	145	5.39	1851	68.81
3	451	16.77	2302	85.58
4	123	4.57	2425	90.15
5	20	0.74	2445	90.89
6	2	0.07	2447	90.97
7	243	9.03	2690	100.00
Frequency Missing = 1				

*1/12 follow-up 224: 1-month re-admission vs withina month??, cause 1: cardiac, 2:non-cardiac, 3: unknown, ;

The FREQ Procedure

Cath Lab Hospital				
**Cath_Lab_Hospital**	**Frequency**	**Percent**	**Cumulative Frequency**	**Cumulative Percent**
**1**	1740	64.68	1740	64.68
**2**	950	35.32	2690	100.00
**Frequency Missing = 1**				

The FREQ Procedure

Frequency<br>Percent<br>Row Pct<br>Col Pct

Table of Gender by Was_the_Pt__given_a_thrombolytic			
	Was_the_Pt__given_a_thrombolytic(Was the Pt. given a thrombolytic outside your hospital?)		
Gender(Gender)	1	2	Total
1	173<br>13.18<br>14.79<br>90.10	997<br>75.93<br>85.21<br>88.94	1170<br>89.11
2	19<br>1.45<br>13.29<br>9.90	124<br>9.44<br>86.71<br>11.06	143<br>10.89
Total	192<br>14.62	1121<br>85.38	1313<br>100.00

Frequency<br>Percent<br>Row Pct<br>Col Pct

Table of Gender by Was_thrombolytic_therapy_given_i			
	Was_thrombolytic_therapy_given_i(Was thrombolytic therapy given inside your hospital)		
Gender(Gender)	1	2	Total
1	356<br>29.82<br>33.55<br>86.62	705<br>59.05<br>66.45<br>90.04	1061<br>88.86
2	55<br>4.61<br>41.35<br>13.38	78<br>6.53<br>58.65<br>9.96	133<br>11.14
Total	411<br>34.42	783<br>65.58	1194<br>100.00
Frequency Missing = 119			

Frequency<br>Percent<br>Row Pct<br>Col Pct

Table of Gender by Thrombolytics_given__outside_of			
	Thrombolytics_given__outside_of(Thrombolytics given (outside of your hospital))		
Gender(Gender)	1	2	Total
1	132<br>33.25<br>37.71<br>91.67	218<br>54.91<br>62.29<br>86.17	350<br>88.16
2	12<br>3.02<br>25.53<br>8.33	35<br>8.82<br>74.47<br>13.83	47<br>11.84
Total	144<br>36.27	253<br>63.73	397<br>100.00
Frequency Missing = 916			

The FREQ Procedure

Frequency<br>Percent<br>Row Pct<br>Col Pct

Table of Gender by Arterial_access				
	Arterial_access(Arterial access)			
Gender(Gender)	1	2	3	Total
1	118<br>19.47<br>21.49<br>89.39	430<br>70.96<br>78.32<br>90.91	1<br>0.17<br>0.18<br>100.00	549<br>90.59
2	14<br>2.31<br>24.56<br>10.61	43<br>7.10<br>75.44<br>9.09	0<br>0.00<br>0.00<br>0.00	57<br>9.41
Total	132<br>21.78	473<br>78.05	1<br>0.17	606<br>100.00
Frequency Missing = 707				

Frequency<br>Percent<br>Row Pct<br>Col Pct

Table of Gender by Arterial_access_1			
	Arterial_access_1(Arterial access_1)		
Gender(Gender)	1	2	Total
1	52<br>23.11<br>25.62<br>86.67	151<br>67.11<br>74.38<br>91.52	203<br>90.22
2	8<br>3.56<br>36.36<br>13.33	14<br>6.22<br>63.64<br>8.48	22<br>9.78
Total	60<br>26.67	165<br>73.33	225<br>100.00
Frequency Missing = 1088			

Frequency<br>Percent<br>Row Pct<br>Col Pct

Table of Gender by VAR108			
	VAR108(Rescue Cath +/- PCI)		
Gender(Gender)	1	2	Total
1	60<br>82.19<br>93.75<br>88.24	4<br>5.48<br>6.25<br>80.00	64<br>87.67
2	8<br>10.96<br>88.89<br>11.76	1<br>1.37<br>11.11<br>20.00	9<br>12.33
Total	68<br>93.15	5<br>6.85	73<br>100.00
Frequency Missing = 1240			

The FREQ Procedure

Frequency<br>Percent<br>Row Pct<br>Col Pct

Table of Gender by Thrombectomy_device_used_1			
	Thrombectomy_device_used_1(Thrombectomy device used_1)		
Gender(Gender)	1	2	Total
1	10<br>5.24<br>5.85<br>100.00	161<br>84.29<br>94.15<br>88.95	171<br>89.53
2	0<br>0.00<br>0.00<br>0.00	20<br>10.47<br>100.00<br>11.05	20<br>10.47
Total	10<br>5.24	181<br>94.76	191<br>100.00
Frequency Missing = 1122			

Frequency<br>Percent<br>Row Pct<br>Col Pct

Table of Gender by Thrombectomy_device_used			
	Thrombectomy_device_used(Thrombectomy device used)		
Gender(Gender)	1	2	Total
1	109<br>17.99<br>19.85<br>94.78	440<br>72.61<br>80.15<br>89.61	549<br>90.59
2	6<br>0.99<br>10.53<br>5.22	51<br>8.42<br>89.47<br>10.39	57<br>9.41
Total	115<br>18.98	491<br>81.02	606<br>100.00
Frequency Missing = 707			

Frequency<br>Percent<br>Row Pct<br>Col Pct

Table of Gender by Clinical_Signs_of_Reperfusion			
	Clinical_Signs_of_Reperfusion(Clinical Signs of Reperfusion)		
Gender(Gender)	1	2	Total
1	73<br>55.73<br>61.86<br>92.41	45<br>34.35<br>38.14<br>86.54	118<br>90.08
2	6<br>4.58<br>46.15<br>7.59	7<br>5.34<br>53.85<br>13.46	13<br>9.92
Total	79<br>60.31	52<br>39.69	131<br>100.00
Frequency Missing = 1182			

The FREQ Procedure

Frequency<br>Percent<br>Row Pct<br>Col Pct

Table of Gender by Did_the_Pt__present_to_your_hosp			
	Did_the_Pt__present_to_your_hosp(Did the Pt. present to your hospital with clinical signs of reperfusion)		
Gender(Gender)	1	2	Total
1	109<br>57.67<br>64.12<br>91.60	61<br>32.28<br>35.88<br>87.14	170<br>89.95
2	10<br>5.29<br>52.63<br>8.40	9<br>4.76<br>47.37<br>12.86	19<br>10.05
Total	119<br>62.96	70<br>37.04	189<br>100.00
Frequency Missing = 1124			

Frequency<br>Percent<br>Row Pct<br>Col Pct

Table of Gender by Directly_transferred_to_Cath_lab			
	Directly_transferred_to_Cath_lab(Directly transferred to Cath-lab hospital (Drip & ship pathway))		
Gender(Gender)	1	2	Total
1	238<br>64.50<br>74.84<br>85.00	80<br>21.68<br>25.16<br>89.89	318<br>86.18
2	42<br>11.38<br>82.35<br>15.00	9<br>2.44<br>17.65<br>10.11	51<br>13.82
Total	280<br>75.88	89<br>24.12	369<br>100.00
Frequency Missing = 944			

Frequency<br>Percent<br>Row Pct<br>Col Pct

Table of Gender by Primary_PCI_done			
	Primary_PCI_done(Primary PCI done)		
Gender(Gender)	1	2	Total
1	490<br>79.42<br>88.45<br>90.91	64<br>10.37<br>11.55<br>82.05	554<br>89.79
2	49<br>7.94<br>77.78<br>9.09	14<br>2.27<br>22.22<br>17.95	63<br>10.21
Total	539<br>87.36	78<br>12.64	617<br>100.00
Frequency Missing = 696			

The FREQ Procedure

Frequency<br>Percent<br>Row Pct<br>Col Pct

Table of Gender by Transferred_for_primary_PCI

Gender(Gender)	Transferred_for_primary_PCI(Transferred for primary PCI) 1	2	Total
1	73<br>64.04<br>69.52<br>91.25	32<br>28.07<br>30.48<br>94.12	105<br>92.11
2	7<br>6.14<br>77.78<br>8.75	2<br>1.75<br>22.22<br>5.88	9<br>7.89
Total	80<br>70.18	34<br>29.82	114<br>100.00

Frequency Missing = 1199

Frequency<br>Percent<br>Row Pct<br>Col Pct

Table of Gender by No_thrombolytic_therapy_or_Prima

Gender(Gender)	No_thrombolytic_therapy_or_Prima(No thrombolytic therapy or Primary PCI: Why not? Choose one) 1	2	4	Total
1	44<br>56.41<br>68.75<br>84.62	5<br>6.41<br>7.81<br>100.00	15<br>19.23<br>23.44<br>71.43	64<br>82.05
2	8<br>10.26<br>57.14<br>15.38	0<br>0.00<br>0.00<br>0.00	6<br>7.69<br>42.86<br>28.57	14<br>17.95
Total	52<br>66.67	5<br>6.41	21<br>26.92	78<br>100.00

Frequency Missing = 1235

Frequency<br>Percent<br>Row Pct<br>Col Pct

Table of Gender by No_thrombolytic_therapy_or_trans

Gender(Gender)	No_thrombolytic_therapy_or_trans(No thrombolytic therapy or transferred for Primary PCI: Why not? Choose one) 1	2	3	4	Total
1	24<br>70.59<br>75.00<br>96.00	1<br>2.94<br>3.13<br>50.00	6<br>17.65<br>18.75<br>100.00	1<br>2.94<br>3.13<br>100.00	32<br>94.12
2	1<br>2.94<br>50.00<br>4.00	1<br>2.94<br>50.00<br>50.00	0<br>0.00<br>0.00<br>0.00	0<br>0.00<br>0.00<br>0.00	2<br>5.88
Total	25<br>73.53	2<br>5.88	6<br>17.65	1<br>2.94	34<br>100.00

Frequency Missing = 1279

The FREQ Procedure

Frequency<br>Percent<br>Row Pct<br>Col Pct

Table of Gender by Elective_Cath_done__within_3_24			
	Elective_Cath_done__within_3_24(Elective Cath done (within 3-24 hrs. from reperfusion))		
Gender(Gender)	1	2	Total
1	15<br>83.33<br>88.24<br>93.75	2<br>11.11<br>11.76<br>100.00	17<br>94.44
2	1<br>5.56<br>100.00<br>6.25	0<br>0.00<br>0.00<br>0.00	1<br>5.56
Total	16<br>88.89	2<br>11.11	18<br>100.00
Frequency Missing = 1295			
