# Supplementary material for: The second survey of the Saudi Acute Myocardial Infarction Registry Program: Main results and temporal changes in care (STARS-2 program)
Source: PLoS One. 2025 Sep 2;20(9):e0331215. doi: 10.1371/journal.pone.0331215 (PMC12404464; doi:10.1371/journal.pone.0331215)
Supplement: S1 Data — (ZIP) [file pone.0331215.s011.zip › Raw data/Reperfusion overall and by gender.pdf]

stemi vs nstemi

The FREQ Procedure

Was the Pt. given a thrombolytic outside your hospital?				
Was_the_Pt__given_a_thrombolytic	Frequency	Percent	Cumulative Frequency	Cumulative Percent
1	204	15.40	204	15.40
2	1121	84.60	1325	100.00
Frequency Missing = 1366				

Was thrombolytic therapy given inside your hospital				
Was_thrombolytic_therapy_given_i	Frequency	Percent	Cumulative Frequency	Cumulative Percent
1	411	34.42	411	34.42
2	783	65.58	1194	100.00
Frequency Missing = 1497				

Thrombolytics given (outside of your hospital)				
Thrombolytics_given__outside_of	Frequency	Percent	Cumulative Frequency	Cumulative Percent
1	157	25.08	157	25.08
2	469	74.92	626	100.00
Frequency Missing = 2065				

Arterial access				
Arterial_access	Frequency	Percent	Cumulative Frequency	Cumulative Percent
1	282	24.12	282	24.12
2	886	75.79	1168	99.91
3	1	0.09	1169	100.00
Frequency Missing = 1522				

Arterial access_1				
Arterial_access_1	Frequency	Percent	Cumulative Frequency	Cumulative Percent
1	97	23.32	97	23.32
2	319	76.68	416	100.00
Frequency Missing = 2275				

The FREQ Procedure

Rescue Cath +/- PCI				
VAR108	Frequency	Percent	Cumulative Frequency	Cumulative Percent
1	68	93.15	68	93.15
2	5	6.85	73	100.00
Frequency Missing = 2618				

Thrombectomy device used_1				
Thrombectomy_device_used_1	Frequency	Percent	Cumulative Frequency	Cumulative Percent
1	16	4.58	16	4.58
2	333	95.42	349	100.00
Frequency Missing = 2342				

Thrombectomy device used				
Thrombectomy_device_used	Frequency	Percent	Cumulative Frequency	Cumulative Percent
1	115	18.98	115	18.98
2	491	81.02	606	100.00
Frequency Missing = 2085				

Clinical Signs of Reperfusion				
Clinical_Signs_of_Reperfusion	Frequency	Percent	Cumulative Frequency	Cumulative Percent
1	79	60.31	79	60.31
2	52	39.69	131	100.00
Frequency Missing = 2560				

Did the Pt. present to your hospital with clinical signs of reperfusion				
Did_the_Pt__present_to_your_hosp	Frequency	Percent	Cumulative Frequency	Cumulative Percent
1	119	62.96	119	62.96
2	70	37.04	189	100.00
Frequency Missing = 2502				

The FREQ Procedure

Directly transferred to Cath-lab hospital (Drip & ship pathway)				
Directly_transferred_to_Cath_lab	Frequency	Percent	Cumulative Frequency	Cumulative Percent
1	280	75.88	280	75.88
2	89	24.12	369	100.00
Frequency Missing = 2322				

Primary PCI done				
Primary_PCI_done	Frequency	Percent	Cumulative Frequency	Cumulative Percent
1	539	87.36	539	87.36
2	78	12.64	617	100.00
Frequency Missing = 2074				

Transferred for primary PCI				
Transferred_for_primary_PCI	Frequency	Percent	Cumulative Frequency	Cumulative Percent
1	80	70.18	80	70.18
2	34	29.82	114	100.00
Frequency Missing = 2577				

No thrombolytic therapy or Primary PCI: Why not? Choose one				
No_thrombolytic_therapy_or_Prima	Frequency	Percent	Cumulative Frequency	Cumulative Percent
1	52	66.67	52	66.67
2	5	6.41	57	73.08
4	21	26.92	78	100.00
Frequency Missing = 2613				

No thrombolytic therapy or transferred for Primary PCI: Why not? Choose one				
No_thrombolytic_therapy_or_trans	Frequency	Percent	Cumulative Frequency	Cumulative Percent
1	25	73.53	25	73.53
2	2	5.88	27	79.41
3	6	17.65	33	97.06
4	1	2.94	34	100.00
Frequency Missing = 2657				

The FREQ Procedure

Elective Cath done (within 3-24 hrs. from reperfusion)				
Elective_Cath_done__within_3_24	Frequency	Percent	Cumulative Frequency	Cumulative Percent
**1**	16	88.89	16	88.89
**2**	2	11.11	18	100.00
**Frequency Missing = 2673**				

The FREQ Procedure

Frequency<br>Percent<br>Row Pct<br>Col Pct

Table of Gender by Was_the_Pt__given_a_thrombolytic			
	Was_the_Pt__given_a_thrombolytic(Was the Pt. given a thrombolytic outside your hospital?)		
Gender(Gender)	1	2	Total
1	182<br>13.74<br>15.44<br>89.22	997<br>75.25<br>84.56<br>88.94	1179<br>88.98
2	22<br>1.66<br>15.07<br>10.78	124<br>9.36<br>84.93<br>11.06	146<br>11.02
Total	204<br>15.40	1121<br>84.60	1325<br>100.00
Frequency Missing = 1366			

Frequency<br>Percent<br>Row Pct<br>Col Pct

Table of Gender by Was_thrombolytic_therapy_given_i			
	Was_thrombolytic_therapy_given_i(Was thrombolytic therapy given inside your hospital)		
Gender(Gender)	1	2	Total
1	356<br>29.82<br>33.55<br>86.62	705<br>59.05<br>66.45<br>90.04	1061<br>88.86
2	55<br>4.61<br>41.35<br>13.38	78<br>6.53<br>58.65<br>9.96	133<br>11.14
Total	411<br>34.42	783<br>65.58	1194<br>100.00
Frequency Missing = 1497			

Frequency<br>Percent<br>Row Pct<br>Col Pct

Table of Gender by Thrombolytics_given__outside_of			
	Thrombolytics_given__outside_of(Thrombolytics given (outside of your hospital))		
Gender(Gender)	1	2	Total
1	142<br>22.68<br>27.57<br>90.45	373<br>59.58<br>72.43<br>79.53	515<br>82.27
2	15<br>2.40<br>13.51<br>9.55	96<br>15.34<br>86.49<br>20.47	111<br>17.73
Total	157<br>25.08	469<br>74.92	626<br>100.00
Frequency Missing = 2065			

The FREQ Procedure

Frequency<br>Percent<br>Row Pct<br>Col Pct

Table of Gender by Arterial_access				
	Arterial_access(Arterial access)			
Gender(Gender)	1	2	3	Total
1	225<br>19.25<br>22.80<br>79.79	761<br>65.10<br>77.10<br>85.89	1<br>0.09<br>0.10<br>100.00	987<br>84.43
2	57<br>4.88<br>31.32<br>20.21	125<br>10.69<br>68.68<br>14.11	0<br>0.00<br>0.00<br>0.00	182<br>15.57
Total	282<br>24.12	886<br>75.79	1<br>0.09	1169<br>100.00
Frequency Missing = 1522				

Frequency<br>Percent<br>Row Pct<br>Col Pct

Table of Gender by Arterial_access_1			
	Arterial_access_1(Arterial access_1)		
Gender(Gender)	1	2	Total
1	77<br>18.51<br>21.63<br>79.38	279<br>67.07<br>78.37<br>87.46	356<br>85.58
2	20<br>4.81<br>33.33<br>20.62	40<br>9.62<br>66.67<br>12.54	60<br>14.42
Total	97<br>23.32	319<br>76.68	416<br>100.00
Frequency Missing = 2275			

Frequency<br>Percent<br>Row Pct<br>Col Pct

Table of Gender by VAR108			
	VAR108(Rescue Cath +/- PCI)		
Gender(Gender)	1	2	Total
1	60<br>82.19<br>93.75<br>88.24	4<br>5.48<br>6.25<br>80.00	64<br>87.67
2	8<br>10.96<br>88.89<br>11.76	1<br>1.37<br>11.11<br>20.00	9<br>12.33
Total	68<br>93.15	5<br>6.85	73<br>100.00
Frequency Missing = 2618			

stemi vs nstemi

The FREQ Procedure

Frequency<br>Percent<br>Row Pct<br>Col Pct

Table of Gender by Thrombectomy_device_used_1			
	Thrombectomy_device_used_1(Thrombectomy device used_1)		
Gender(Gender)	1	2	Total
1	16<br>4.58<br>5.35<br>100.00	283<br>81.09<br>94.65<br>84.98	299<br>85.67
2	0<br>0.00<br>0.00<br>0.00	50<br>14.33<br>100.00<br>15.02	50<br>14.33
Total	16<br>4.58	333<br>95.42	349<br>100.00
Frequency Missing = 2342			

Frequency<br>Percent<br>Row Pct<br>Col Pct

Table of Gender by Thrombectomy_device_used			
	Thrombectomy_device_used(Thrombectomy device used)		
Gender(Gender)	1	2	Total
1	109<br>17.99<br>19.85<br>94.78	440<br>72.61<br>80.15<br>89.61	549<br>90.59
2	6<br>0.99<br>10.53<br>5.22	51<br>8.42<br>89.47<br>10.39	57<br>9.41
Total	115<br>18.98	491<br>81.02	606<br>100.00
Frequency Missing = 2085			

Frequency<br>Percent<br>Row Pct<br>Col Pct

Table of Gender by Clinical_Signs_of_Reperfusion			
	Clinical_Signs_of_Reperfusion(Clinical Signs of Reperfusion)		
Gender(Gender)	1	2	Total
1	73<br>55.73<br>61.86<br>92.41	45<br>34.35<br>38.14<br>86.54	118<br>90.08
2	6<br>4.58<br>46.15<br>7.59	7<br>5.34<br>53.85<br>13.46	13<br>9.92
Total	79<br>60.31	52<br>39.69	131<br>100.00
Frequency Missing = 2560			

The FREQ Procedure

Frequency
Percent
Row Pct
Col Pct

Table of Gender by Did_the_Pt__present_to_your_hosp

Gender(Gender)	Did_the_Pt__present_to_your_hosp(Did the Pt. present to your hospital with clinical signs of reperfusion) 1	2	Total
1	109<br>57.67<br>64.12<br>91.60	61<br>32.28<br>35.88<br>87.14	170<br>89.95
2	10<br>5.29<br>52.63<br>8.40	9<br>4.76<br>47.37<br>12.86	19<br>10.05
Total	119<br>62.96	70<br>37.04	189<br>100.00

Frequency Missing = 2502

Frequency
Percent
Row Pct
Col Pct

Table of Gender by Directly_transferred_to_Cath_lab

Gender(Gender)	Directly_transferred_to_Cath_lab(Directly transferred to Cath-lab hospital (Drip & ship pathway)) 1	2	Total
1	238<br>64.50<br>74.84<br>85.00	80<br>21.68<br>25.16<br>89.89	318<br>86.18
2	42<br>11.38<br>82.35<br>15.00	9<br>2.44<br>17.65<br>10.11	51<br>13.82
Total	280<br>75.88	89<br>24.12	369<br>100.00

Frequency Missing = 2322

Frequency
Percent
Row Pct
Col Pct

Table of Gender by Primary_PCI_done

Gender(Gender)	Primary_PCI_done(Primary PCI done) 1	2	Total
1	490<br>79.42<br>88.45<br>90.91	64<br>10.37<br>11.55<br>82.05	554<br>89.79
2	49<br>7.94<br>77.78<br>9.09	14<br>2.27<br>22.22<br>17.95	63<br>10.21
Total	539<br>87.36	78<br>12.64	617<br>100.00

Frequency Missing = 2074

The FREQ Procedure

Frequency<br>Percent<br>Row Pct<br>Col Pct

Table of Gender by Transferred_for_primary_PCI

Gender(Gender)	Transferred_for_primary_PCI(Transferred for primary PCI) 1	2	Total
1	73<br>64.04<br>69.52<br>91.25	32<br>28.07<br>30.48<br>94.12	105<br>92.11
2	7<br>6.14<br>77.78<br>8.75	2<br>1.75<br>22.22<br>5.88	9<br>7.89
Total	80<br>70.18	34<br>29.82	114<br>100.00

Frequency Missing = 2577

Frequency<br>Percent<br>Row Pct<br>Col Pct

Table of Gender by No_thrombolytic_therapy_or_Prima

Gender(Gender)	No_thrombolytic_therapy_or_Prima(No thrombolytic therapy or Primary PCI: Why not? Choose one) 1	2	4	Total
1	44<br>56.41<br>68.75<br>84.62	5<br>6.41<br>7.81<br>100.00	15<br>19.23<br>23.44<br>71.43	64<br>82.05
2	8<br>10.26<br>57.14<br>15.38	0<br>0.00<br>0.00<br>0.00	6<br>7.69<br>42.86<br>28.57	14<br>17.95
Total	52<br>66.67	5<br>6.41	21<br>26.92	78<br>100.00

Frequency Missing = 2613

Frequency<br>Percent<br>Row Pct<br>Col Pct

Table of Gender by No_thrombolytic_therapy_or_trans

Gender(Gender)	No_thrombolytic_therapy_or_trans(No thrombolytic therapy or transferred for Primary PCI: Why not? Choose one) 1	2	3	4	Total
1	24<br>70.59<br>75.00<br>96.00	1<br>2.94<br>3.13<br>50.00	6<br>17.65<br>18.75<br>100.00	1<br>2.94<br>3.13<br>100.00	32<br>94.12
2	1<br>2.94<br>50.00<br>4.00	1<br>2.94<br>50.00<br>50.00	0<br>0.00<br>0.00<br>0.00	0<br>0.00<br>0.00<br>0.00	2<br>5.88
Total	25<br>73.53	2<br>5.88	6<br>17.65	1<br>2.94	34<br>100.00

Frequency Missing = 2657

The FREQ Procedure

Frequency<br>Percent<br>Row Pct<br>Col Pct

Table of Gender by Elective_Cath_done__within_3_24			
	Elective_Cath_done__within_3_24(Elective Cath done (within 3-24 hrs. from reperfusion))		
Gender(Gender)	1	2	Total
1	15<br>83.33<br>88.24<br>93.75	2<br>11.11<br>11.76<br>100.00	17<br>94.44
2	1<br>5.56<br>100.00<br>6.25	0<br>0.00<br>0.00<br>0.00	1<br>5.56
Total	16<br>88.89	2<br>11.11	18<br>100.00
Frequency Missing = 2673			
