# Supplementary material for: The second survey of the Saudi Acute Myocardial Infarction Registry Program: Main results and temporal changes in care (STARS-2 program)
Source: PLoS One. 2025 Sep 2;20(9):e0331215. doi: 10.1371/journal.pone.0331215 (PMC12404464; doi:10.1371/journal.pone.0331215)
Supplement: S1 Data — (ZIP) [file pone.0331215.s011.zip › Raw data/STEMI and cath lab hospital numbers and figure 1.pdf]

STEMI and Cath labs

The FREQ Procedure

STEMI/NSTEMI				
**STEMI_NSTEMI**	**Frequency**	**Percent**	**Cumulative Frequency**	**Cumulative Percent**
**1**	1313	48.81	1313	48.81
**2**	1377	51.19	2690	100.00
**Frequency Missing = 1**				

Cath Lab Hospital				
**Cath_Lab_Hospital**	**Frequency**	**Percent**	**Cumulative Frequency**	**Cumulative Percent**
**1**	1740	64.68	1740	64.68
**2**	950	35.32	2690	100.00
**Frequency Missing = 1**				

The UNIVARIATE Procedure
Variable: symptom_to_ED_min__0 (symptom_to_ED_min_>0)

Moments			
N	1311	Sum Weights	1311
Mean	545.43402	Sum Observations	715064
Std Deviation	1444.03704	Variance	2085242.97
Skewness	14.5800927	Kurtosis	341.639042
Uncorrected SS	3121688524	Corrected SS	2731668292
Coeff Variation	264.750087	Std Error Mean	39.8820053

Basic Statistical Measures			
Location		Variability	
Mean	545.4340	Std Deviation	1444
Median	180.0000	Variance	2085243
Mode	120.0000	Range	37745
		Interquartile Range	409.00000

Note: The mode displayed is the smallest of 2 modes with a count of 27.

Tests for Location: Mu0=0				
Test	Statistic		p Value	
Student's t	t	13.67619	Pr > |t|	<.0001
Sign	M	655.5	Pr >= |M|	<.0001
Signed Rank	S	430008	Pr >= |S|	<.0001

Quantiles (Definition 5)	
Level	Quantile
100% Max	37746
99%	5143
95%	2349
90%	1260
75% Q3	473
50% Median	180
25% Q1	64
10%	19
5%	5
1%	1
0% Min	1

The UNIVARIATE Procedure
Variable: symptom_to_ED_min__0 (symptom_to_ED_min_>0)

Extreme Observations			
Lowest		Highest	
Value	Obs	Value	Obs
1	2468	7828	1254
1	2464	8660	1112
1	2122	10185	116
1	1659	10203	1354
1	1610	37746	2147

Missing Values			
		Percent Of	
Missing Value	Count	All Obs	Missing Obs
.	2	0.15	100.00

The UNIVARIATE Procedure
Variable: symptom_to_FMC_min__0 (symptom_to_FMC_min_>0)

Moments			
**N**	397	**Sum Weights**	397
**Mean**	381.642317	**Sum Observations**	151512
**Std Deviation**	954.375121	**Variance**	910831.872
**Skewness**	5.72132389	**Kurtosis**	41.4084914
**Uncorrected SS**	418512812	**Corrected SS**	360689421
**Coeff Variation**	250.070571	**Std Error Mean**	47.8987143

Basic Statistical Measures			
**Location**		**Variability**	
**Mean**	381.6423	**Std Deviation**	954.37512
**Median**	120.0000	**Variance**	910832
**Mode**	30.0000	**Range**	10059
		**Interquartile Range**	265.00000

Tests for Location: Mu0=0				
**Test**	**Statistic**		**p Value**	
**Student's t**	**t**	7.967694	**Pr > |t|**	<.0001
**Sign**	**M**	198.5	**Pr >= |M|**	<.0001
**Signed Rank**	**S**	39501.5	**Pr >= |S|**	<.0001

Quantiles (Definition 5)	
**Level**	**Quantile**
**100% Max**	10060
**99%**	4418
**95%**	1595
**90%**	840
**75% Q3**	300
**50% Median**	120
**25% Q1**	35
**10%**	15
**5%**	10
**1%**	3
**0% Min**	1

The UNIVARIATE Procedure
Variable: symptom_to_FMC_min__0 (symptom_to_FMC_min_>0)

Extreme Observations			
Lowest		Highest	
Value	Obs	Value	Obs
1	211	4385	760
2	2503	4418	261
2	240	6660	110
3	1525	7410	1112
3	889	10060	1354

Missing Values			
		Percent Of	
Missing Value	Count	All Obs	Missing Obs
.	916	69.76	100.00

The UNIVARIATE Procedure
Variable: FMC_to_ED_min__0 (FMC_to_ED_min_>0)

Moments			
**N**	397	**Sum Weights**	397
**Mean**	418.085642	**Sum Observations**	165980
**Std Deviation**	755.177561	**Variance**	570293.149
**Skewness**	3.70594854	**Kurtosis**	16.3822745
**Uncorrected SS**	295229942	**Corrected SS**	225836087
**Coeff Variation**	180.62748	**Std Error Mean**	37.9012753

Basic Statistical Measures			
Location		Variability	
**Mean**	418.0856	**Std Deviation**	755.17756
**Median**	135.0000	**Variance**	570293
**Mode**	6.0000	**Range**	5725
		**Interquartile Range**	376.00000

Note: The mode displayed is the smallest of 5 modes with a count of 5.

Tests for Location: Mu0=0				
Test	Statistic		p Value	
**Student's t**	**t**	11.03091	**Pr > |t|**	<.0001
**Sign**	**M**	198.5	**Pr >= |M|**	<.0001
**Signed Rank**	**S**	39501.5	**Pr >= |S|**	<.0001

Quantiles (Definition 5)	
Level	Quantile
**100% Max**	5726
**99%**	4395
**95%**	1740
**90%**	1065
**75% Q3**	428
**50% Median**	135
**25% Q1**	52
**10%**	14
**5%**	8
**1%**	5
**0% Min**	1

The UNIVARIATE Procedure
Variable: FMC_to_ED_min__0 (FMC_to_ED_min_>0)

Extreme Observations			
Lowest		Highest	
Value	Obs	Value	Obs
1	38	4080	219
1	514	4395	351
2	2165	4435	270
5	84	5008	670
5	736	5726	1254

Missing Values			
		Percent Of	
Missing Value	Count	All Obs	Missing Obs
.	916	69.76	100.00

The UNIVARIATE Procedure
Variable: ED_ECG_min__0 (ED_ECG_min_>0)

Moments			
**N**	1257	**Sum Weights**	1257
**Mean**	14.0684169	**Sum Observations**	17684
**Std Deviation**	55.419981	**Variance**	3071.3743
**Skewness**	18.7737137	**Kurtosis**	421.715872
**Uncorrected SS**	4106432	**Corrected SS**	3857646.12
**Coeff Variation**	393.931894	**Std Error Mean**	1.56314309

Basic Statistical Measures			
Location		Variability	
**Mean**	14.06842	**Std Deviation**	55.41998
**Median**	7.00000	**Variance**	3071
**Mode**	10.00000	**Range**	1446
		**Interquartile Range**	8.00000

Tests for Location: Mu0=0				
**Test**	Statistic		p Value	
**Student's t**	t	9.000083	Pr > |t|	<.0001
**Sign**	M	628.5	Pr >= |M|	<.0001
**Signed Rank**	S	395326.5	Pr >= |S|	<.0001

Quantiles (Definition 5)	
**Level**	**Quantile**
**100% Max**	1447
**99%**	117
**95%**	32
**90%**	21
**75% Q3**	12
**50% Median**	7
**25% Q1**	4
**10%**	2
**5%**	1
**1%**	1
**0% Min**	1

The UNIVARIATE Procedure
Variable: ED_ECG_min__0 (ED_ECG_min_>0)

Extreme Observations			
Lowest		Highest	
Value	Obs	Value	Obs
1	2677	302	959
1	2619	550	1244
1	2572	565	1165
1	2564	896	2664
1	2538	1447	1733

Missing Values			
		Percent Of	
Missing Value	Count	All Obs	Missing Obs
.	56	4.27	100.00

The UNIVARIATE Procedure
Variable: ECG_to_lytic_2_min__0 (ECG_to_lytic_2_min_>0)

Moments			
**N**	404	**Sum Weights**	404
**Mean**	14.0074257	**Sum Observations**	5659
**Std Deviation**	72.6258124	**Variance**	5274.50863
**Skewness**	19.1771651	**Kurtosis**	378.749656
**Uncorrected SS**	2204895	**Corrected SS**	2125626.98
**Coeff Variation**	518.480796	**Std Error Mean**	3.61326922

Basic Statistical Measures			
Location		Variability	
**Mean**	14.00743	**Std Deviation**	72.62581
**Median**	7.00000	**Variance**	5275
**Mode**	10.00000	**Range**	1446
		**Interquartile Range**	11.00000

Tests for Location: Mu0=0				
Test	Statistic		p Value	
**Student's t**	**t**	3.876663	**Pr > |t|**	0.0001
**Sign**	**M**	202	**Pr >= |M|**	<.0001
**Signed Rank**	**S**	40905	**Pr >= |S|**	<.0001

Quantiles (Definition 5)	
Level	Quantile
**100% Max**	1447
**99%**	80
**95%**	30
**90%**	20
**75% Q3**	14
**50% Median**	7
**25% Q1**	3
**10%**	2
**5%**	1
**1%**	1
**0% Min**	1

The UNIVARIATE Procedure
Variable: ECG_to_lytic_2_min__0 (ECG_to_lytic_2_min_>0)

Extreme Observations			
Lowest		Highest	
Value	Obs	Value	Obs
1	2677	80	1664
1	2619	90	1965
1	2445	112	1660
1	2341	121	2595
1	2249	1447	1733

Missing Values			
		Percent Of	
Missing Value	Count	All Obs	Missing Obs
.	909	69.23	100.00

The UNIVARIATE Procedure
Variable: ECG_to_baloon_min__0 (ECG_to_baloon_min_>0)

Moments			
N	515	Sum Weights	515
Mean	76.592233	Sum Observations	39445
Std Deviation	110.281638	Variance	12162.0396
Skewness	6.91209233	Kurtosis	65.5285023
Uncorrected SS	9272469	Corrected SS	6251288.37
Coeff Variation	143.985406	Std Error Mean	4.85958958

Basic Statistical Measures			
Location		Variability	
Mean	76.59223	Std Deviation	110.28164
Median	55.00000	Variance	12162
Mode	45.00000	Range	1475
		Interquartile Range	40.00000

Tests for Location: Mu0=0				
Test	Statistic		p Value	
Student's t	t	15.76105	Pr > |t|	<.0001
Sign	M	257	Pr >= |M|	<.0001
Signed Rank	S	66177.5	Pr >= |S|	<.0001

Quantiles (Definition 5)	
Level	Quantile
100% Max	1475
99%	575
95%	205
90%	130
75% Q3	77
50% Median	55
25% Q1	37
10%	18
5%	11
1%	4
0% Min	0

The UNIVARIATE Procedure
Variable: ECG_to_baloon_min__0 (ECG_to_baloon_min_>0)

Extreme Observations			
Lowest		Highest	
Value	Obs	Value	Obs
0	846	682	1336
2	1078	778	398
2	864	784	2560
4	515	914	51
4	411	1475	164

Missing Values			
		Percent Of	
Missing Value	Count	All Obs	Missing Obs
.	798	60.78	100.00
