# Supplementary material for: The second survey of the Saudi Acute Myocardial Infarction Registry Program: Main results and temporal changes in care (STARS-2 program)
Source: PLoS One. 2025 Sep 2;20(9):e0331215. doi: 10.1371/journal.pone.0331215 (PMC12404464; doi:10.1371/journal.pone.0331215)

table 1 - categorical

The FREQ Procedure

Frequency<br>Percent<br>Row Pct<br>Col Pct

Table of STEMI_NSTEMI by Gender			
	Gender(Gender)		
STEMI_NSTEMI(STEMI/NSTEMI)	1	2	Total
1	1170<br>43.49<br>89.11<br>52.97	143<br>5.32<br>10.89<br>29.73	1313<br>48.81
2	1039<br>38.62<br>75.45<br>47.03	338<br>12.57<br>24.55<br>70.27	1377<br>51.19
Total	2209<br>82.12	481<br>17.88	2690<br>100.00
Frequency Missing = 1			

Statistics for Table of STEMI_NSTEMI by Gender

Statistic	DF	Value	Prob
Chi-Square	1	85.3484	<.0001
Likelihood Ratio Chi-Square	1	87.6263	<.0001
Continuity Adj. Chi-Square	1	84.4210	<.0001
Mantel-Haenszel Chi-Square	1	85.3166	<.0001
Phi Coefficient		0.1781	
Contingency Coefficient		0.1754	
Cramer's V		0.1781	

Fisher's Exact Test	
Cell (1,1) Frequency (F)	1170
Left-sided Pr <= F	1.0000
Right-sided Pr >= F	<.0001
Table Probability (P)	<.0001
Two-sided Pr <= P	<.0001

Sample Size = 2690
Frequency Missing = 1

The FREQ Procedure

Frequency<br>Percent<br>Row Pct<br>Col Pct

Table of STEMI_NSTEMI by Nationality			
	Nationality(Nationality)		
STEMI_NSTEMI(STEMI/NSTEMI)	1	2	Total
1	791<br>29.41<br>60.24<br>42.48	522<br>19.41<br>39.76<br>63.04	1313<br>48.81
2	1071<br>39.81<br>77.78<br>57.52	306<br>11.38<br>22.22<br>36.96	1377<br>51.19
Total	1862<br>69.22	828<br>30.78	2690<br>100.00
Frequency Missing = 1			

Statistics for Table of STEMI_NSTEMI by Nationality

Statistic	DF	Value	Prob
Chi-Square	1	96.9853	<.0001
Likelihood Ratio Chi-Square	1	97.7476	<.0001
Continuity Adj. Chi-Square	1	96.1641	<.0001
Mantel-Haenszel Chi-Square	1	96.9493	<.0001
Phi Coefficient		-0.1899	
Contingency Coefficient		0.1865	
Cramer's V		-0.1899	

Fisher's Exact Test	
Cell (1,1) Frequency (F)	791
Left-sided Pr <= F	<.0001
Right-sided Pr >= F	1.0000
Table Probability (P)	<.0001
Two-sided Pr <= P	<.0001

Sample Size = 2690
Frequency Missing = 1

The FREQ Procedure

Frequency<br>Percent<br>Row Pct<br>Col Pct

Table of STEMI_NSTEMI by Ethnicity

	Ethnicity(Ethnicity)			
STEMI_NSTEMI(STEMI/NSTEMI)	1	2	3	Total
1	926<br>34.42<br>70.53<br>44.12	340<br>12.64<br>25.89<br>67.33	47<br>1.75<br>3.58<br>54.65	1313<br>48.81
2	1173<br>43.61<br>85.19<br>55.88	165<br>6.13<br>11.98<br>32.67	39<br>1.45<br>2.83<br>45.35	1377<br>51.19
Total	2099<br>78.03	505<br>18.77	86<br>3.20	2690<br>100.00

Frequency Missing = 1

Statistics for Table of STEMI_NSTEMI by Ethnicity

Statistic	DF	Value	Prob
Chi-Square	2	88.9812	<.0001
Likelihood Ratio Chi-Square	2	90.2753	<.0001
Mantel-Haenszel Chi-Square	1	63.2192	<.0001
Phi Coefficient		0.1819	
Contingency Coefficient		0.1789	
Cramer's V		0.1819	

Sample Size = 2690
Frequency Missing = 1

Frequency<br>Percent<br>Row Pct<br>Col Pct

Table of STEMI_NSTEMI by Type_of_STEMI

	Type_of_STEMI(Type of STEMI)				
STEMI_NSTEMI(STEMI/NSTEMI)	1	2	3	999	Total
1	690<br>25.65<br>52.55<br>100.00	545<br>20.26<br>41.51<br>100.00	78<br>2.90<br>5.94<br>100.00	0<br>0.00<br>0.00<br>0.00	1313<br>48.81
2	0<br>0.00<br>0.00<br>0.00	0<br>0.00<br>0.00<br>0.00	0<br>0.00<br>0.00<br>0.00	1377<br>51.19<br>100.00<br>100.00	1377<br>51.19
Total	690<br>25.65	545<br>20.26	78<br>2.90	1377<br>51.19	2690<br>100.00

Frequency Missing = 1

The FREQ Procedure

Statistics for Table of STEMI_NSTEMI by Type_of_STEMI

Statistic	DF	Value	Prob
Chi-Square	3	2690.0000	<.0001
Likelihood Ratio Chi-Square	3	3727.6090	<.0001
Mantel-Haenszel Chi-Square	1	2688.9981	<.0001
Phi Coefficient		1.0000	
Contingency Coefficient		0.7071	
Cramer's V		1.0000	

Sample Size = 2690
Frequency Missing = 1

Frequency<br>Percent<br>Row Pct<br>Col Pct

Table of STEMI_NSTEMI by History_of_angina			
	History_of_angina(History of angina)		
STEMI_NSTEMI(STEMI/NSTEMI)	1	2	Total
1	252<br>9.37<br>19.19<br>34.05	1061<br>39.44<br>80.81<br>54.41	1313<br>48.81
2	488<br>18.14<br>35.44<br>65.95	889<br>33.05<br>64.56<br>45.59	1377<br>51.19
Total	740<br>27.51	1950<br>72.49	2690<br>100.00
Frequency Missing = 1			

The FREQ Procedure

Statistics for Table of STEMI_NSTEMI by History_of_angina

Statistic	DF	Value	Prob
Chi-Square	1	88.9638	<.0001
Likelihood Ratio Chi-Square	1	90.2638	<.0001
Continuity Adj. Chi-Square	1	88.1510	<.0001
Mantel-Haenszel Chi-Square	1	88.9308	<.0001
Phi Coefficient		-0.1819	
Contingency Coefficient		0.1789	
Cramer's V		-0.1819	

Fisher's Exact Test	
Cell (1,1) Frequency (F)	252
Left-sided Pr <= F	<.0001
Right-sided Pr >= F	1.0000
Table Probability (P)	<.0001
Two-sided Pr <= P	<.0001

Sample Size = 2690
Frequency Missing = 1

Frequency
Percent
Row Pct
Col Pct

Table of STEMI_NSTEMI by History_of_MI			
	History_of_MI(History of MI)		
STEMI_NSTEMI(STEMI/NSTEMI)	1	2	Total
1	112<br>4.16<br>8.53<br>27.79	1201<br>44.65<br>91.47<br>52.51	1313<br>48.81
2	291<br>10.82<br>21.13<br>72.21	1086<br>40.37<br>78.87<br>47.49	1377<br>51.19
Total	403<br>14.98	2287<br>85.02	2690<br>100.00
Frequency Missing = 1			

The FREQ Procedure

Statistics for Table of STEMI_NSTEMI by History_of_MI

Statistic	DF	Value	Prob
Chi-Square	1	83.8137	<.0001
Likelihood Ratio Chi-Square	1	86.6140	<.0001
Continuity Adj. Chi-Square	1	82.8271	<.0001
Mantel-Haenszel Chi-Square	1	83.7825	<.0001
Phi Coefficient		-0.1765	
Contingency Coefficient		0.1738	
Cramer's V		-0.1765	

Fisher's Exact Test	
Cell (1,1) Frequency (F)	112
Left-sided Pr <= F	<.0001
Right-sided Pr >= F	1.0000
Table Probability (P)	<.0001
Two-sided Pr <= P	<.0001

Sample Size = 2690
Frequency Missing = 1

Frequency<br>Percent<br>Row Pct<br>Col Pct

Table of STEMI_NSTEMI by History_of_PCI			
	History_of_PCI(History of PCI)		
STEMI_NSTEMI(STEMI/NSTEMI)	1	2	Total
1	112<br>4.16<br>8.53<br>26.73	1201<br>44.65<br>91.47<br>52.88	1313<br>48.81
2	307<br>11.41<br>22.29<br>73.27	1070<br>39.78<br>77.71<br>47.12	1377<br>51.19
Total	419<br>15.58	2271<br>84.42	2690<br>100.00
Frequency Missing = 1			

The FREQ Procedure

Statistics for Table of STEMI_NSTEMI by History_of_PCI

Statistic	DF	Value	Prob
Chi-Square	1	96.8405	<.0001
Likelihood Ratio Chi-Square	1	100.3877	<.0001
Continuity Adj. Chi-Square	1	95.7966	<.0001
Mantel-Haenszel Chi-Square	1	96.8045	<.0001
Phi Coefficient		-0.1897	
Contingency Coefficient		0.1864	
Cramer's V		-0.1897	

Fisher's Exact Test	
Cell (1,1) Frequency (F)	112
Left-sided Pr <= F	<.0001
Right-sided Pr >= F	1.0000
Table Probability (P)	<.0001
Two-sided Pr <= P	<.0001

Sample Size = 2690
Frequency Missing = 1

Frequency
Percent
Row Pct
Col Pct

Table of STEMI_NSTEMI by History_of_CABG			
	History_of_CABG(History of CABG)		
STEMI_NSTEMI(STEMI/NSTEMI)	1	2	Total
1	15<br>0.56<br>1.14<br>21.13	1298<br>48.25<br>98.86<br>49.56	1313<br>48.81
2	56<br>2.08<br>4.07<br>78.87	1321<br>49.11<br>95.93<br>50.44	1377<br>51.19
Total	71<br>2.64	2619<br>97.36	2690<br>100.00
Frequency Missing = 1			

The FREQ Procedure

Statistics for Table of STEMI_NSTEMI by History_of_CABG

Statistic	DF	Value	Prob
Chi-Square	1	22.3680	<.0001
Likelihood Ratio Chi-Square	1	23.8864	<.0001
Continuity Adj. Chi-Square	1	21.2445	<.0001
Mantel-Haenszel Chi-Square	1	22.3597	<.0001
Phi Coefficient		-0.0912	
Contingency Coefficient		0.0908	
Cramer's V		-0.0912	

Fisher's Exact Test	
Cell (1,1) Frequency (F)	15
Left-sided Pr <= F	<.0001
Right-sided Pr >= F	1.0000
Table Probability (P)	<.0001
Two-sided Pr <= P	<.0001

Sample Size = 2690
Frequency Missing = 1

Frequency<br>Percent<br>Row Pct<br>Col Pct

Table of STEMI_NSTEMI by History_of_heart_failure			
	History_of_heart_failure(History of heart failure)		
STEMI_NSTEMI(STEMI/NSTEMI)	1	2	Total
1	31<br>1.15<br>2.36<br>19.02	1282<br>47.66<br>97.64<br>50.73	1313<br>48.81
2	132<br>4.91<br>9.59<br>80.98	1245<br>46.28<br>90.41<br>49.27	1377<br>51.19
Total	163<br>6.06	2527<br>93.94	2690<br>100.00
Frequency Missing = 1			

The FREQ Procedure

Statistics for Table of STEMI_NSTEMI by History_of_heart_failure

Statistic	DF	Value	Prob
Chi-Square	1	61.6368	<.0001
Likelihood Ratio Chi-Square	1	66.3893	<.0001
Continuity Adj. Chi-Square	1	60.3741	<.0001
Mantel-Haenszel Chi-Square	1	61.6139	<.0001
Phi Coefficient		-0.1514	
Contingency Coefficient		0.1497	
Cramer's V		-0.1514	

Fisher's Exact Test	
Cell (1,1) Frequency (F)	31
Left-sided Pr <= F	<.0001
Right-sided Pr >= F	1.0000
Table Probability (P)	<.0001
Two-sided Pr <= P	<.0001

Sample Size = 2690
Frequency Missing = 1

Frequency
Percent
Row Pct
Col Pct

Table of STEMI_NSTEMI by History_of_stroke			
	History_of_stroke(History of stroke)		
STEMI_NSTEMI(STEMI/NSTEMI)	1	2	Total
1	35<br>1.30<br>2.67<br>28.46	1278<br>47.51<br>97.33<br>49.79	1313<br>48.81
2	88<br>3.27<br>6.39<br>71.54	1289<br>47.92<br>93.61<br>50.21	1377<br>51.19
Total	123<br>4.57	2567<br>95.43	2690<br>100.00
Frequency Missing = 1			

The FREQ Procedure

Statistics for Table of STEMI_NSTEMI by History_of_stroke

Statistic	DF	Value	Prob
Chi-Square	1	21.3740	<.0001
Likelihood Ratio Chi-Square	1	22.1268	<.0001
Continuity Adj. Chi-Square	1	20.5288	<.0001
Mantel-Haenszel Chi-Square	1	21.3660	<.0001
Phi Coefficient		-0.0891	
Contingency Coefficient		0.0888	
Cramer's V		-0.0891	

Fisher's Exact Test	
Cell (1,1) Frequency (F)	35
Left-sided Pr <= F	<.0001
Right-sided Pr >= F	1.0000
Table Probability (P)	<.0001
Two-sided Pr <= P	<.0001

Sample Size = 2690
Frequency Missing = 1

Frequency<br>Percent<br>Row Pct<br>Col Pct

Table of STEMI_NSTEMI by History_of_chronic_renal_failure			
	History_of_chronic_renal_failure(History of chronic renal failure)		
STEMI_NSTEMI(STEMI/NSTEMI)	1	2	Total
1	29<br>1.08<br>2.21<br>14.50	1284<br>47.73<br>97.79<br>51.57	1313<br>48.81
2	171<br>6.36<br>12.42<br>85.50	1206<br>44.83<br>87.58<br>48.43	1377<br>51.19
Total	200<br>7.43	2490<br>92.57	2690<br>100.00
Frequency Missing = 1			

The FREQ Procedure

Statistics for Table of STEMI_NSTEMI by History_of_chronic_renal_failure

Statistic	DF	Value	Prob
Chi-Square	1	101.7983	<.0001
Likelihood Ratio Chi-Square	1	112.6050	<.0001
Continuity Adj. Chi-Square	1	100.3202	<.0001
Mantel-Haenszel Chi-Square	1	101.7605	<.0001
Phi Coefficient		-0.1945	
Contingency Coefficient		0.1910	
Cramer's V		-0.1945	

Fisher's Exact Test	
Cell (1,1) Frequency (F)	29
Left-sided Pr <= F	<.0001
Right-sided Pr >= F	1.0000
Table Probability (P)	<.0001
Two-sided Pr <= P	<.0001

Sample Size = 2690
Frequency Missing = 1

Frequency<br>Percent<br>Row Pct<br>Col Pct

Table of STEMI_NSTEMI by DM			
	DM(DM)		
STEMI_NSTEMI(STEMI/NSTEMI)	1	2	Total
1	685<br>25.46<br>52.17<br>43.94	628<br>23.35<br>47.83<br>55.53	1313<br>48.81
2	874<br>32.49<br>63.47<br>56.06	503<br>18.70<br>36.53<br>44.47	1377<br>51.19
Total	1559<br>57.96	1131<br>42.04	2690<br>100.00
Frequency Missing = 1			

The FREQ Procedure

Statistics for Table of STEMI_NSTEMI by DM

Statistic	DF	Value	Prob
Chi-Square	1	35.2252	<.0001
Likelihood Ratio Chi-Square	1	35.2899	<.0001
Continuity Adj. Chi-Square	1	34.7630	<.0001
Mantel-Haenszel Chi-Square	1	35.2121	<.0001
Phi Coefficient		-0.1144	
Contingency Coefficient		0.1137	
Cramer's V		-0.1144	

Fisher's Exact Test	
Cell (1,1) Frequency (F)	685
Left-sided Pr <= F	<.0001
Right-sided Pr >= F	1.0000
Table Probability (P)	<.0001
Two-sided Pr <= P	<.0001

Sample Size = 2690
Frequency Missing = 1

Frequency<br>Percent<br>Row Pct<br>Col Pct

Table of STEMI_NSTEMI by HTN			
	HTN(HTN)		
STEMI_NSTEMI(STEMI/NSTEMI)	1	2	Total
1	643<br>23.90<br>48.97<br>40.85	670<br>24.91<br>51.03<br>60.04	1313<br>48.81
2	931<br>34.61<br>67.61<br>59.15	446<br>16.58<br>32.39<br>39.96	1377<br>51.19
Total	1574<br>58.51	1116<br>41.49	2690<br>100.00
Frequency Missing = 1			

The FREQ Procedure

Statistics for Table of STEMI_NSTEMI by HTN

Statistic	DF	Value	Prob
Chi-Square	1	96.1887	<.0001
Likelihood Ratio Chi-Square	1	96.7390	<.0001
Continuity Adj. Chi-Square	1	95.4224	<.0001
Mantel-Haenszel Chi-Square	1	96.1529	<.0001
Phi Coefficient		-0.1891	
Contingency Coefficient		0.1858	
Cramer's V		-0.1891	

Fisher's Exact Test	
Cell (1,1) Frequency (F)	643
Left-sided Pr <= F	<.0001
Right-sided Pr >= F	1.0000
Table Probability (P)	<.0001
Two-sided Pr <= P	<.0001

Sample Size = 2690
Frequency Missing = 1

Frequency
Percent
Row Pct
Col Pct

Table of STEMI_NSTEMI by Hypercholestrolemia			
	Hypercholestrolemia(Hypercholestrolemia)		
STEMI_NSTEMI(STEMI/NSTEMI)	1	2	Total
1	444<br>16.51<br>33.82<br>42.33	869<br>32.30<br>66.18<br>52.96	1313<br>48.81
2	605<br>22.49<br>43.94<br>57.67	772<br>28.70<br>56.06<br>47.04	1377<br>51.19
Total	1049<br>39.00	1641<br>61.00	2690<br>100.00
Frequency Missing = 1			

The FREQ Procedure

Statistics for Table of STEMI_NSTEMI by Hypercholestrolemia

Statistic	DF	Value	Prob
Chi-Square	1	28.9376	<.0001
Likelihood Ratio Chi-Square	1	29.0224	<.0001
Continuity Adj. Chi-Square	1	28.5137	<.0001
Mantel-Haenszel Chi-Square	1	28.9268	<.0001
Phi Coefficient		-0.1037	
Contingency Coefficient		0.1032	
Cramer's V		-0.1037	

Fisher's Exact Test	
Cell (1,1) Frequency (F)	444
Left-sided Pr <= F	<.0001
Right-sided Pr >= F	1.0000
Table Probability (P)	<.0001
Two-sided Pr <= P	<.0001

Sample Size = 2690
Frequency Missing = 1

Frequency<br>Percent<br>Row Pct<br>Col Pct

Table of STEMI_NSTEMI by Current_or_ex_smoking			
	Current_or_ex_smoking(Current or ex-smoking)		
STEMI_NSTEMI(STEMI/NSTEMI)	1	2	Total
1	643<br>23.90<br>48.97<br>55.10	670<br>24.91<br>51.03<br>43.99	1313<br>48.81
2	524<br>19.48<br>38.05<br>44.90	853<br>31.71<br>61.95<br>56.01	1377<br>51.19
Total	1167<br>43.38	1523<br>56.62	2690<br>100.00
Frequency Missing = 1			

The FREQ Procedure

Statistics for Table of STEMI_NSTEMI by Current_or_ex_smoking

Statistic	DF	Value	Prob
Chi-Square	1	32.6192	<.0001
Likelihood Ratio Chi-Square	1	32.6749	<.0001
Continuity Adj. Chi-Square	1	32.1762	<.0001
Mantel-Haenszel Chi-Square	1	32.6070	<.0001
Phi Coefficient		0.1101	
Contingency Coefficient		0.1095	
Cramer's V		0.1101	

Fisher's Exact Test	
Cell (1,1) Frequency (F)	643
Left-sided Pr <= F	1.0000
Right-sided Pr >= F	<.0001
Table Probability (P)	<.0001
Two-sided Pr <= P	<.0001

Sample Size = 2690
Frequency Missing = 1

Frequency<br>Percent<br>Row Pct<br>Col Pct

Table of STEMI_NSTEMI by Chief_complaint						
	Chief_complaint(Chief complaint)					
STEMI_NSTEMI(STEMI/NSTEMI)	1	2	3	4	5	Total
1	1203<br>44.72<br>91.62<br>51.32	29<br>1.08<br>2.21<br>16.48	59<br>2.19<br>4.49<br>46.46	13<br>0.48<br>0.99<br>92.86	9<br>0.33<br>0.69<br>31.03	1313<br>48.81
2	1141<br>42.42<br>82.86<br>48.68	147<br>5.46<br>10.68<br>83.52	68<br>2.53<br>4.94<br>53.54	1<br>0.04<br>0.07<br>7.14	20<br>0.74<br>1.45<br>68.97	1377<br>51.19
Total	2344<br>87.14	176<br>6.54	127<br>4.72	14<br>0.52	29<br>1.08	2690<br>100.00
Frequency Missing = 1						

The FREQ Procedure

Statistics for Table of STEMI_NSTEMI by Chief_complaint

Statistic	DF	Value	Prob
Chi-Square	4	94.3802	<.0001
Likelihood Ratio Chi-Square	4	103.7053	<.0001
Mantel-Haenszel Chi-Square	1	14.7590	0.0001
Phi Coefficient		0.1873	
Contingency Coefficient		0.1841	
Cramer's V		0.1873	

Sample Size = 2690
Frequency Missing = 1

Frequency<br>Percent<br>Row Pct<br>Col Pct

Table of STEMI_NSTEMI by _1st_medical_contact						
	_1st_medical_contact(1st medical contact)					
STEMI_NSTEMI(STEMI/NSTEMI)	1	2	3	4	999	Total
1	73<br>2.71<br>5.56<br>54.89	244<br>9.07<br>18.58<br>67.40	1<br>0.04<br>0.08<br>100.00	79<br>2.94<br>6.02<br>60.77	916<br>34.05<br>69.76<br>44.38	1313<br>48.81
2	60<br>2.23<br>4.36<br>45.11	118<br>4.39<br>8.57<br>32.60	0<br>0.00<br>0.00<br>0.00	51<br>1.90<br>3.70<br>39.23	1148<br>42.68<br>83.37<br>55.62	1377<br>51.19
Total	133<br>4.94	362<br>13.46	1<br>0.04	130<br>4.83	2064<br>76.73	2690<br>100.00
Frequency Missing = 1						

Statistics for Table of STEMI_NSTEMI by _1st_medical_contact

Statistic	DF	Value	Prob
Chi-Square	4	76.7561	<.0001
Likelihood Ratio Chi-Square	4	78.1350	<.0001
Mantel-Haenszel Chi-Square	1	69.6472	<.0001
Phi Coefficient		0.1689	
Contingency Coefficient		0.1666	
Cramer's V		0.1689	

Sample Size = 2690
Frequency Missing = 1

The FREQ Procedure

Frequency<br>Percent<br>Row Pct<br>Col Pct

Table of STEMI_NSTEMI by Transferred_by_EMS_e_g__Red_Cres				
	Transferred_by_EMS_e_g__Red_Cres(Transferred by EMS e.g. Red Crescent or Red Cross?)			
**STEMI_NSTEMI(STEMI/NSTEMI)**	**1**	**2**	**999**	**Total**
**1**	156<br>5.80<br>11.88<br>68.12	226<br>8.40<br>17.21<br>62.95	931<br>34.61<br>70.91<br>44.29	1313<br>48.81
**2**	73<br>2.71<br>5.30<br>31.88	133<br>4.94<br>9.66<br>37.05	1171<br>43.53<br>85.04<br>55.71	1377<br>51.19
**Total**	229<br>8.51	359<br>13.35	2102<br>78.14	2690<br>100.00
**Frequency Missing = 1**				

Statistics for Table of STEMI_NSTEMI by Transferred_by_EMS_e_g__Red_Cres

Statistic	DF	Value	Prob
Chi-Square	2	80.1000	<.0001
Likelihood Ratio Chi-Square	2	81.0873	<.0001
Mantel-Haenszel Chi-Square	1	78.5873	<.0001
Phi Coefficient		0.1726	
Contingency Coefficient		0.1700	
Cramer's V		0.1726	

Sample Size = 2690
Frequency Missing = 1

Frequency<br>Percent<br>Row Pct<br>Col Pct

Table of STEMI_NSTEMI by Cardiac_arrest			
	Cardiac_arrest(Cardiac arrest)		
**STEMI_NSTEMI(STEMI/NSTEMI)**	**1**	**2**	**Total**
**1**	54<br>2.01<br>4.11<br>72.97	1259<br>46.80<br>95.89<br>48.13	1313<br>48.81
**2**	20<br>0.74<br>1.45<br>27.03	1357<br>50.45<br>98.55<br>51.87	1377<br>51.19
**Total**	74<br>2.75	2616<br>97.25	2690<br>100.00
**Frequency Missing = 1**			

The FREQ Procedure

Statistics for Table of STEMI_NSTEMI by Cardiac_arrest

Statistic	DF	Value	Prob
Chi-Square	1	17.7803	<.0001
Likelihood Ratio Chi-Square	1	18.3730	<.0001
Continuity Adj. Chi-Square	1	16.7998	<.0001
Mantel-Haenszel Chi-Square	1	17.7737	<.0001
Phi Coefficient		0.0813	
Contingency Coefficient		0.0810	
Cramer's V		0.0813	

Fisher's Exact Test	
Cell (1,1) Frequency (F)	54
Left-sided Pr <= F	1.0000
Right-sided Pr >= F	<.0001
Table Probability (P)	<.0001
Two-sided Pr <= P	<.0001

Sample Size = 2690
Frequency Missing = 1

Frequency<br>Percent<br>Row Pct<br>Col Pct

Table of STEMI_NSTEMI by CHF_Killip_Class					
	CHF_Killip_Class(CHF Killip Class)				
STEMI_NSTEMI(STEMI/NSTEMI)	1	2	3	4	Total
1	1166<br>43.35<br>88.80<br>49.45	81<br>3.01<br>6.17<br>41.54	37<br>1.38<br>2.82<br>38.54	29<br>1.08<br>2.21<br>70.73	1313<br>48.81
2	1192<br>44.31<br>86.56<br>50.55	114<br>4.24<br>8.28<br>58.46	59<br>2.19<br>4.28<br>61.46	12<br>0.45<br>0.87<br>29.27	1377<br>51.19
Total	2358<br>87.66	195<br>7.25	96<br>3.57	41<br>1.52	2690<br>100.00
Frequency Missing = 1					

The FREQ Procedure

Statistics for Table of STEMI_NSTEMI by CHF_Killip_Class

Statistic	DF	Value	Prob
Chi-Square	3	16.4484	0.0009
Likelihood Ratio Chi-Square	3	16.7283	0.0008
Mantel-Haenszel Chi-Square	1	0.2259	0.6346
Phi Coefficient		0.0782	
Contingency Coefficient		0.0780	
Cramer's V		0.0782	

Sample Size = 2690
Frequency Missing = 1

Frequency<br>Percent<br>Row Pct<br>Col Pct

Table of STEMI_NSTEMI by Echo_Options						
	Echo_Options(Echo-Options)					
STEMI_NSTEMI(STEMI/NSTEMI)	1	2	3	4	999	Total
1	337<br>12.53<br>25.67<br>35.59	393<br>14.61<br>29.93<br>51.11	261<br>9.70<br>19.88<br>56.74	113<br>4.20<br>8.61<br>54.07	209<br>7.77<br>15.92<br>68.52	1313<br>48.81
2	610<br>22.68<br>44.30<br>64.41	376<br>13.98<br>27.31<br>48.89	199<br>7.40<br>14.45<br>43.26	96<br>3.57<br>6.97<br>45.93	96<br>3.57<br>6.97<br>31.48	1377<br>51.19
Total	947<br>35.20	769<br>28.59	460<br>17.10	209<br>7.77	305<br>11.34	2690<br>100.00
Frequency Missing = 1						

Statistics for Table of STEMI_NSTEMI by Echo_Options

Statistic	DF	Value	Prob
Chi-Square	4	129.2313	<.0001
Likelihood Ratio Chi-Square	4	131.3276	<.0001
Mantel-Haenszel Chi-Square	1	53.8139	<.0001
Phi Coefficient		0.2192	
Contingency Coefficient		0.2141	
Cramer's V		0.2192	

Sample Size = 2690
Frequency Missing = 1

The FREQ Procedure

Frequency<br>Percent<br>Row Pct<br>Col Pct

Table of STEMI_NSTEMI by Elective_coronary_angiogram				
	Elective_coronary_angiogram(Elective coronary angiogram)			
STEMI_NSTEMI(STEMI/NSTEMI)	1	2	999	Total
1	225<br>8.36<br>17.14<br>54.09	594<br>22.08<br>45.24<br>44.86	494<br>18.36<br>37.62<br>52.00	1313<br>48.81
2	191<br>7.10<br>13.87<br>45.91	730<br>27.14<br>53.01<br>55.14	456<br>16.95<br>33.12<br>48.00	1377<br>51.19
Total	416<br>15.46	1324<br>49.22	950<br>35.32	2690<br>100.00
Frequency Missing = 1				

Statistics for Table of STEMI_NSTEMI by Elective_coronary_angiogram

Statistic	DF	Value	Prob
Chi-Square	2	16.7554	0.0002
Likelihood Ratio Chi-Square	2	16.7740	0.0002
Mantel-Haenszel Chi-Square	1	5.9662	0.0146
Phi Coefficient		0.0789	
Contingency Coefficient		0.0787	
Cramer's V		0.0789	

Sample Size = 2690
Frequency Missing = 1

Frequency<br>Percent<br>Row Pct<br>Col Pct

Table of STEMI_NSTEMI by Arterial_access					
	Arterial_access(Arterial access)				
STEMI_NSTEMI(STEMI/NSTEMI)	1	2	3	999	Total
1	132<br>4.91<br>10.06<br>46.81	473<br>17.59<br>36.05<br>53.39	1<br>0.04<br>0.08<br>100.00	706<br>26.26<br>53.81<br>46.45	1312<br>48.79
2	150<br>5.58<br>10.89<br>53.19	413<br>15.36<br>29.99<br>46.61	0<br>0.00<br>0.00<br>0.00	814<br>30.27<br>59.11<br>53.55	1377<br>51.21
Total	282<br>10.49	886<br>32.95	1<br>0.04	1520<br>56.53	2689<br>100.00
Frequency Missing = 2					

The FREQ Procedure

Statistics for Table of STEMI_NSTEMI by Arterial_access

Statistic	DF	Value	Prob
Chi-Square	3	12.3218	0.0064
Likelihood Ratio Chi-Square	3	12.7111	0.0053
Mantel-Haenszel Chi-Square	1	7.6794	0.0056
Phi Coefficient		0.0677	
Contingency Coefficient		0.0675	
Cramer's V		0.0677	
WARNING: 25% of the cells have expected counts less than 5. Chi-Square may not be a valid test.			

Sample Size = 2689
Frequency Missing = 2

Frequency<br>Percent<br>Row Pct<br>Col Pct

Table of STEMI_NSTEMI by Arterial_access_1				
	Arterial_access_1(Arterial access_1)			
STEMI_NSTEMI(STEMI/NSTEMI)	1	2	999	Total
1	60<br>2.23<br>4.57<br>61.86	165<br>6.13<br>12.57<br>51.72	1088<br>40.45<br>82.86<br>47.85	1313<br>48.81
2	37<br>1.38<br>2.69<br>38.14	154<br>5.72<br>11.18<br>48.28	1186<br>44.09<br>86.13<br>52.15	1377<br>51.19
Total	97<br>3.61	319<br>11.86	2274<br>84.54	2690<br>100.00
Frequency Missing = 1				

Statistics for Table of STEMI_NSTEMI by Arterial_access_1

Statistic	DF	Value	Prob
Chi-Square	2	8.5385	0.0140
Likelihood Ratio Chi-Square	2	8.5872	0.0137
Mantel-Haenszel Chi-Square	1	5.4844	0.0192
Phi Coefficient		0.0563	
Contingency Coefficient		0.0563	
Cramer's V		0.0563	

Sample Size = 2690
Frequency Missing = 1

The UNIVARIATE Procedure
Variable: Age (Age)

Moments			
N	2690	Sum Weights	2690
Mean	57.1624535	Sum Observations	153767
Std Deviation	12.4312016	Variance	154.534774
Skewness	0.23567342	Kurtosis	-0.0215841
Uncorrected SS	9205243	Corrected SS	415544.008
Coeff Variation	21.747145	Std Error Mean	0.23968286

Basic Statistical Measures			
Location		Variability	
Mean	57.16245	Std Deviation	12.43120
Median	57.00000	Variance	154.53477
Mode	53.00000	Range	84.00000
		Interquartile Range	16.00000

Tests for Location: Mu0=0				
Test	Statistic		p Value	
Student's t	t	238.492	Pr > |t|	<.0001
Sign	M	1345	Pr >= |M|	<.0001
Signed Rank	S	1809698	Pr >= |S|	<.0001

Quantiles (Definition 5)	
Level	Quantile
100% Max	103
99%	88
95%	79
90%	74
75% Q3	65
50% Median	57
25% Q1	49
10%	42
5%	38
1%	30
0% Min	19

The UNIVARIATE Procedure
Variable: Age (Age)

Extreme Observations			
Lowest		Highest	
Value	Obs	Value	Obs
19	2076	97	1593
19	819	100	1665
22	1775	101	2426
23	528	102	177
24	920	103	2089

Missing Values			
		Percent Of	
Missing Value	Count	All Obs	Missing Obs
.	1	0.04	100.00

The UNIVARIATE Procedure
Variable: BMI (BMI)

Moments			
N	2690	Sum Weights	2690
Mean	28.5318625	Sum Observations	76750.71
Std Deviation	4.77416365	Variance	22.7926385
Skewness	1.23451385	Kurtosis	5.49921412
Uncorrected SS	2251130.11	Corrected SS	61289.405
Coeff Variation	16.7327445	Std Error Mean	0.09204944

Basic Statistical Measures			
Location		Variability	
Mean	28.53186	Std Deviation	4.77416
Median	27.96500	Variance	22.79264
Mode	29.41000	Range	59.84000
		Interquartile Range	5.84000

Tests for Location: Mu0=0				
Test	Statistic		p Value	
Student's t	t	309.9624	Pr > |t|	<.0001
Sign	M	1345	Pr >= |M|	<.0001
Signed Rank	S	1809698	Pr >= |S|	<.0001

Quantiles (Definition 5)	
Level	Quantile
100% Max	75.000
99%	42.750
95%	36.710
90%	34.475
75% Q3	31.200
50% Median	27.965
25% Q1	25.360
10%	23.165
5%	21.940
1%	19.920
0% Min	15.160

The UNIVARIATE Procedure
Variable: BMI (BMI)

Extreme Observations			
Lowest		Highest	
Value	Obs	Value	Obs
15.16	439	56.64	955
17.10	1671	56.64	2307
17.18	1504	56.76	786
17.30	561	57.19	1994
17.30	525	75.00	1543

Missing Values			
		Percent Of	
Missing Value	Count	All Obs	Missing Obs
.	1	0.04	100.00

The UNIVARIATE Procedure
Variable: HR_bpm_ (HR(bpm))

Moments			
N	2689	Sum Weights	2689
Mean	84.1640015	Sum Observations	226317
Std Deviation	17.7514532	Variance	315.114091
Skewness	0.80677197	Kurtosis	4.06061322
Uncorrected SS	19894771	Corrected SS	847026.675
Coeff Variation	21.0915034	Std Error Mean	0.34232492

Basic Statistical Measures			
Location		Variability	
Mean	84.16400	Std Deviation	17.75145
Median	84.00000	Variance	315.11409
Mode	80.00000	Range	220.00000
		Interquartile Range	21.00000

Tests for Location: Mu0=0				
Test	Statistic		p Value	
Student's t	t	245.86	Pr > |t|	<.0001
Sign	M	1344.5	Pr >= |M|	<.0001
Signed Rank	S	1808353	Pr >= |S|	<.0001

Quantiles (Definition 5)	
Level	Quantile
100% Max	230
99%	136
95%	112
90%	105
75% Q3	94
50% Median	84
25% Q1	73
10%	64
5%	59
1%	47
0% Min	10

The UNIVARIATE Procedure
Variable: HR_bpm_ (HR(bpm))

Extreme Observations			
Lowest		Highest	
Value	Obs	Value	Obs
10	1347	170	88
10	1297	173	47
13	1002	190	1565
18	776	196	1810
35	3	230	1681

Missing Values			
		Percent Of	
Missing Value	Count	All Obs	Missing Obs
.	2	0.07	100.00

The UNIVARIATE Procedure
Variable: SBP_mmgH_ (SBP(mmgH))

Moments			
N	2689	Sum Weights	2689
Mean	134.87579	Sum Observations	362681
Std Deviation	25.4554825	Variance	647.98159
Skewness	0.31357686	Kurtosis	0.62734547
Uncorrected SS	50658661	Corrected SS	1741774.51
Coeff Variation	18.8732778	Std Error Mean	0.49089198

Basic Statistical Measures			
Location		Variability	
Mean	134.8758	Std Deviation	25.45548
Median	134.0000	Variance	647.98159
Mode	140.0000	Range	204.00000
		Interquartile Range	31.00000

Tests for Location: Mu0=0				
Test	Statistic		p Value	
Student's t	t	274.7566	Pr > |t|	<.0001
Sign	M	1344.5	Pr >= |M|	<.0001
Signed Rank	S	1808353	Pr >= |S|	<.0001

Quantiles (Definition 5)	
Level	Quantile
100% Max	244
99%	204
95%	180
90%	169
75% Q3	150
50% Median	134
25% Q1	119
10%	105
5%	98
1%	75
0% Min	40

The UNIVARIATE Procedure
Variable: SBP_mmgH_ (SBP(mmgH))

Extreme Observations			
Lowest		Highest	
Value	Obs	Value	Obs
40	1297	220	1785
42	2387	220	1823
55	2326	222	527
60	1602	230	1088
60	1357	244	826

Missing Values			
		Percent Of	
Missing Value	Count	All Obs	Missing Obs
.	2	0.07	100.00

table 1 - continious comparison

The TTEST Procedure

Variable: Age (Age)

STEMI_NSTEMI	Method	N	Mean	Std Dev	Std Err	Minimum	Maximum
1		1313	55.2856	11.9748	0.3305	19.0000	102.0
2		1377	58.9521	12.5977	0.3395	19.0000	103.0
Diff (1-2)	Pooled		-3.6665	12.2976	0.4743		
Diff (1-2)	Satterthwaite		-3.6665		0.4738		

STEMI_NSTEMI	Method	Mean	95% CL Mean		Std Dev	95% CL Std Dev	
1		55.2856	54.6373	55.9339	11.9748	11.5336	12.4512
2		58.9521	58.2861	59.6180	12.5977	12.1442	13.0867
Diff (1-2)	Pooled	-3.6665	-4.5966	-2.7363	12.2976	11.9775	12.6354
Diff (1-2)	Satterthwaite	-3.6665	-4.5955	-2.7375			

Method	Variances	DF	t Value	Pr > |t|
Pooled	Equal	2688	-7.73	<.0001
Satterthwaite	Unequal	2688	-7.74	<.0001

Equality of Variances				
Method	Num DF	Den DF	F Value	Pr > F
Folded F	1376	1312	1.11	0.0633

table 1 - continious comparison

The TTEST Procedure

Variable: Age (Age)

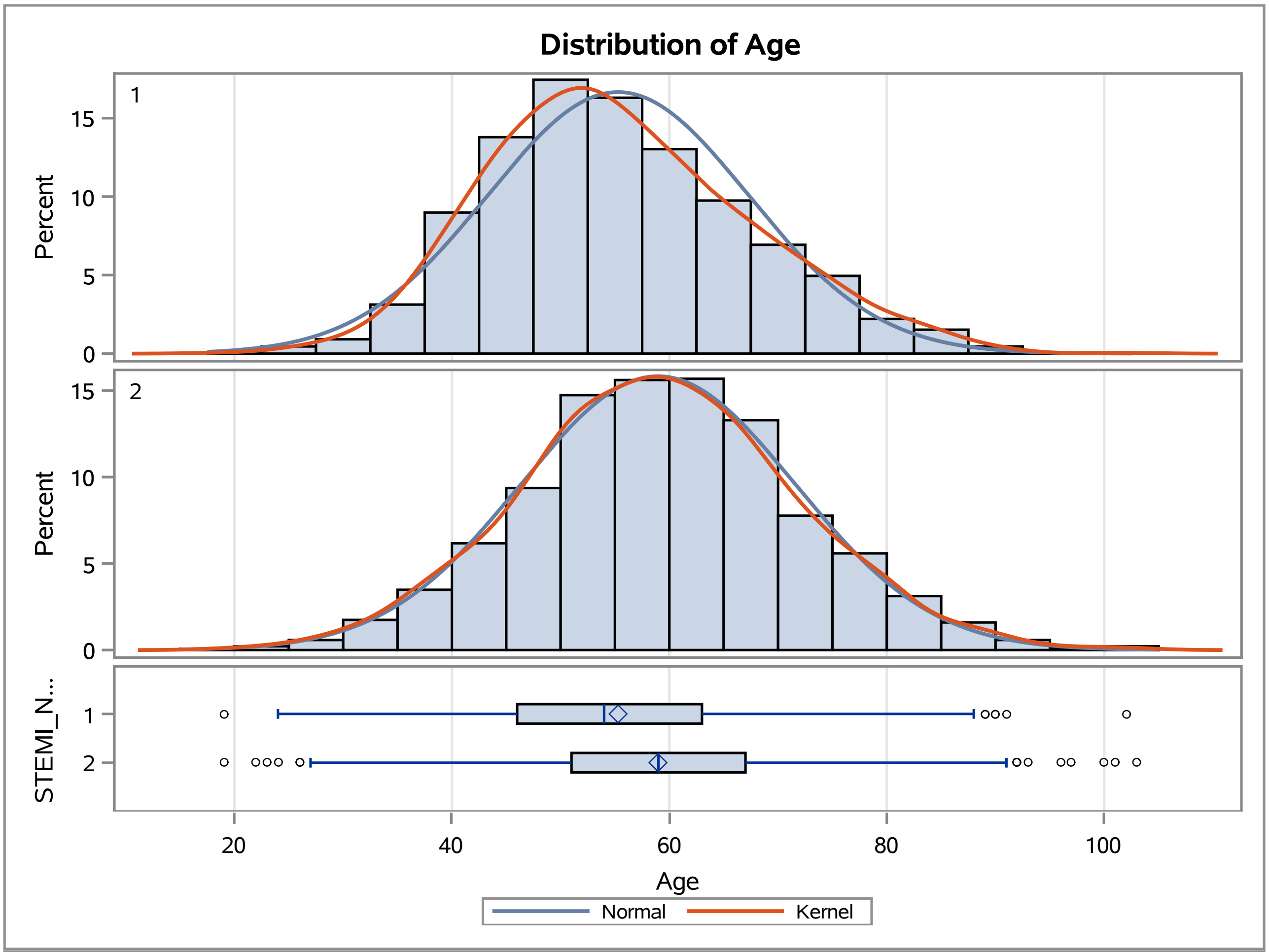

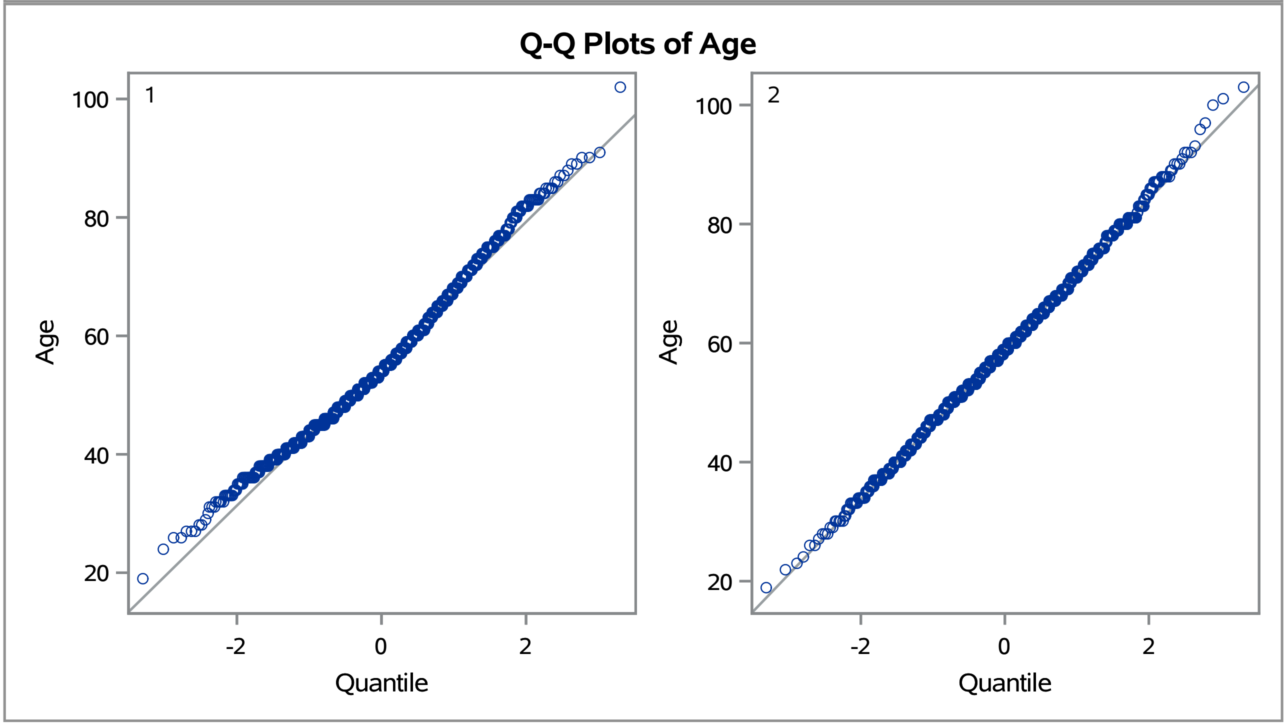

The TTEST Procedure

Variable: BMI (BMI)

Variable: BMI (BMI)

STEMI_NSTEMI	Method	N	Mean	Std Dev	Std Err	Minimum	Maximum
1		1313	28.0655	4.4057	0.1216	17.3000	75.0000
2		1377	28.9765	5.0625	0.1364	15.1600	57.1900
Diff (1-2)	Pooled		-0.9110	4.7533	0.1833		
Diff (1-2)	Satterthwaite		-0.9110		0.1827		

STEMI_NSTEMI	Method	Mean	95% CL Mean		Std Dev	95% CL Std Dev	
1		28.0655	27.8270	28.3040	4.4057	4.2434	4.5810
2		28.9765	28.7089	29.2442	5.0625	4.8802	5.2590
Diff (1-2)	Pooled	-0.9110	-1.2705	-0.5515	4.7533	4.6295	4.8838
Diff (1-2)	Satterthwaite	-0.9110	-1.2694	-0.5527			

Method	Variances	DF	t Value	Pr > |t|
Pooled	Equal	2688	-4.97	<.0001
Satterthwaite	Unequal	2666	-4.99	<.0001

Equality of Variances				
Method	Num DF	Den DF	F Value	Pr > F
Folded F	1376	1312	1.32	<.0001

The TTEST Procedure

Variable: BMI (BMI)

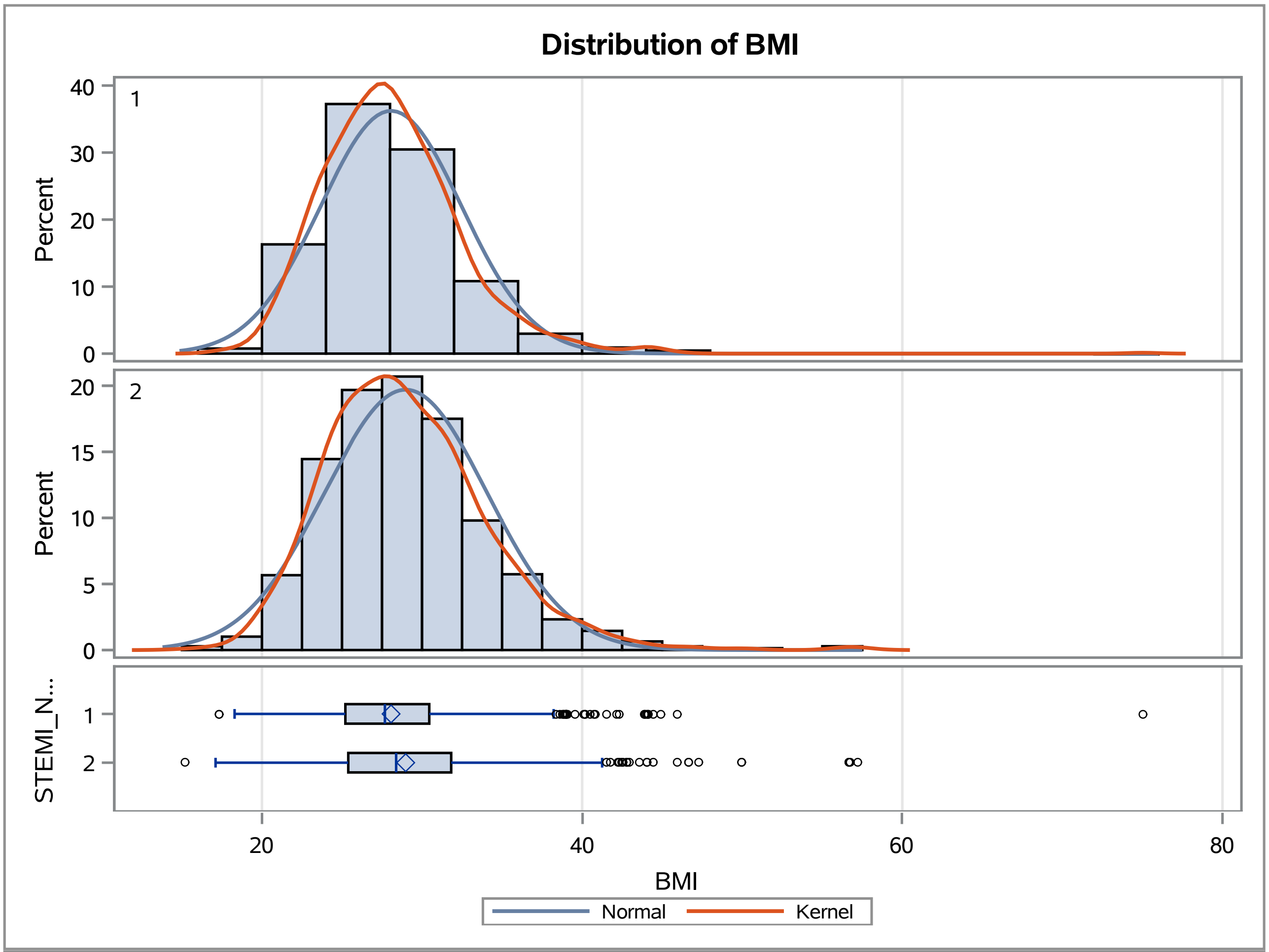

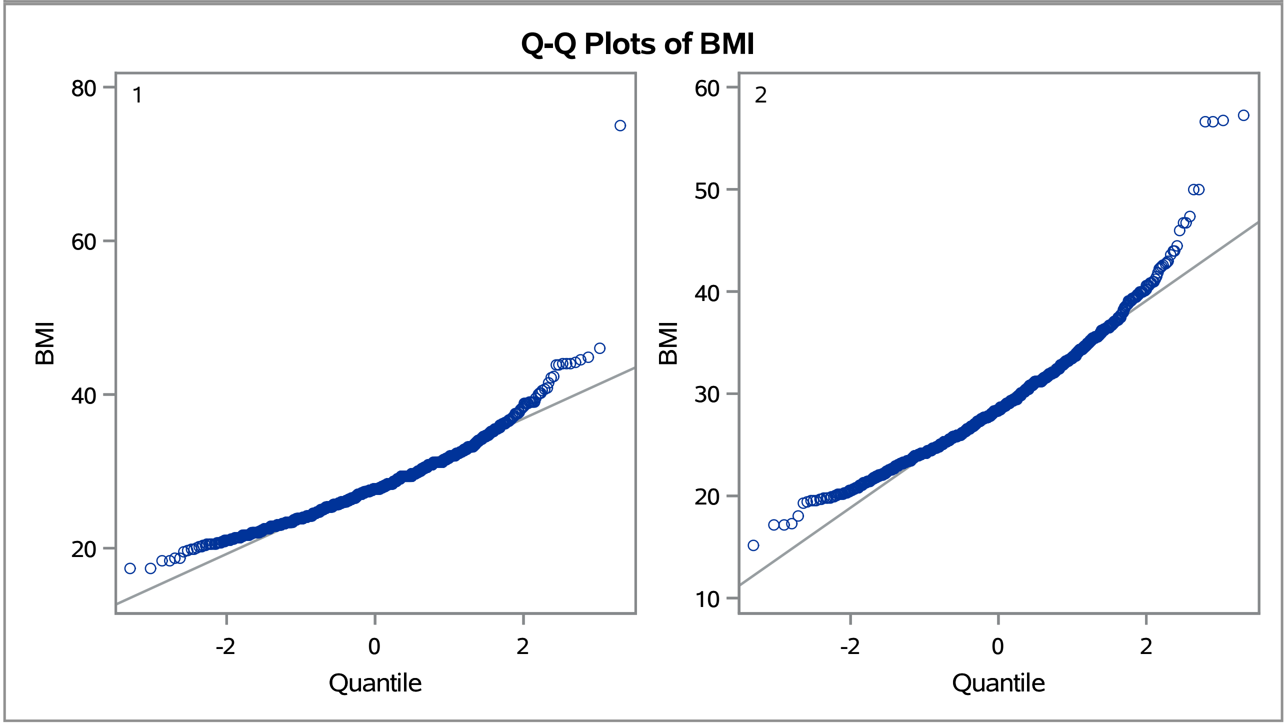

table 1 - continious comparison

The TTEST Procedure

Variable: HR_bpm_ (HR(bpm))

Variable: HR_bpm_ (HR(bpm))

STEMI_NSTEMI	Method	N	Mean	Std Dev	Std Err	Minimum	Maximum
1		1313	83.5834	18.9845	0.5239	10.0000	230.0
2		1376	84.7180	16.4770	0.4442	18.0000	170.0
Diff (1-2)	Pooled		-1.1346	17.7457	0.6846		
Diff (1-2)	Satterthwaite		-1.1346		0.6869		

STEMI_NSTEMI	Method	Mean	95% CL Mean		Std Dev	95% CL Std Dev	
1		83.5834	82.5556	84.6112	18.9845	18.2852	19.7399
2		84.7180	83.8467	85.5894	16.4770	15.8836	17.1168
Diff (1-2)	Pooled	-1.1346	-2.4771	0.2078	17.7457	17.2837	18.2332
Diff (1-2)	Satterthwaite	-1.1346	-2.4815	0.2123			

Method	Variances	DF	t Value	Pr > |t|
Pooled	Equal	2687	-1.66	0.0976
Satterthwaite	Unequal	2596.1	-1.65	0.0987

Equality of Variances				
Method	Num DF	Den DF	F Value	Pr > F
Folded F	1312	1375	1.33	<.0001

The TTEST Procedure

Variable: HR_bpm_ (HR(bpm))

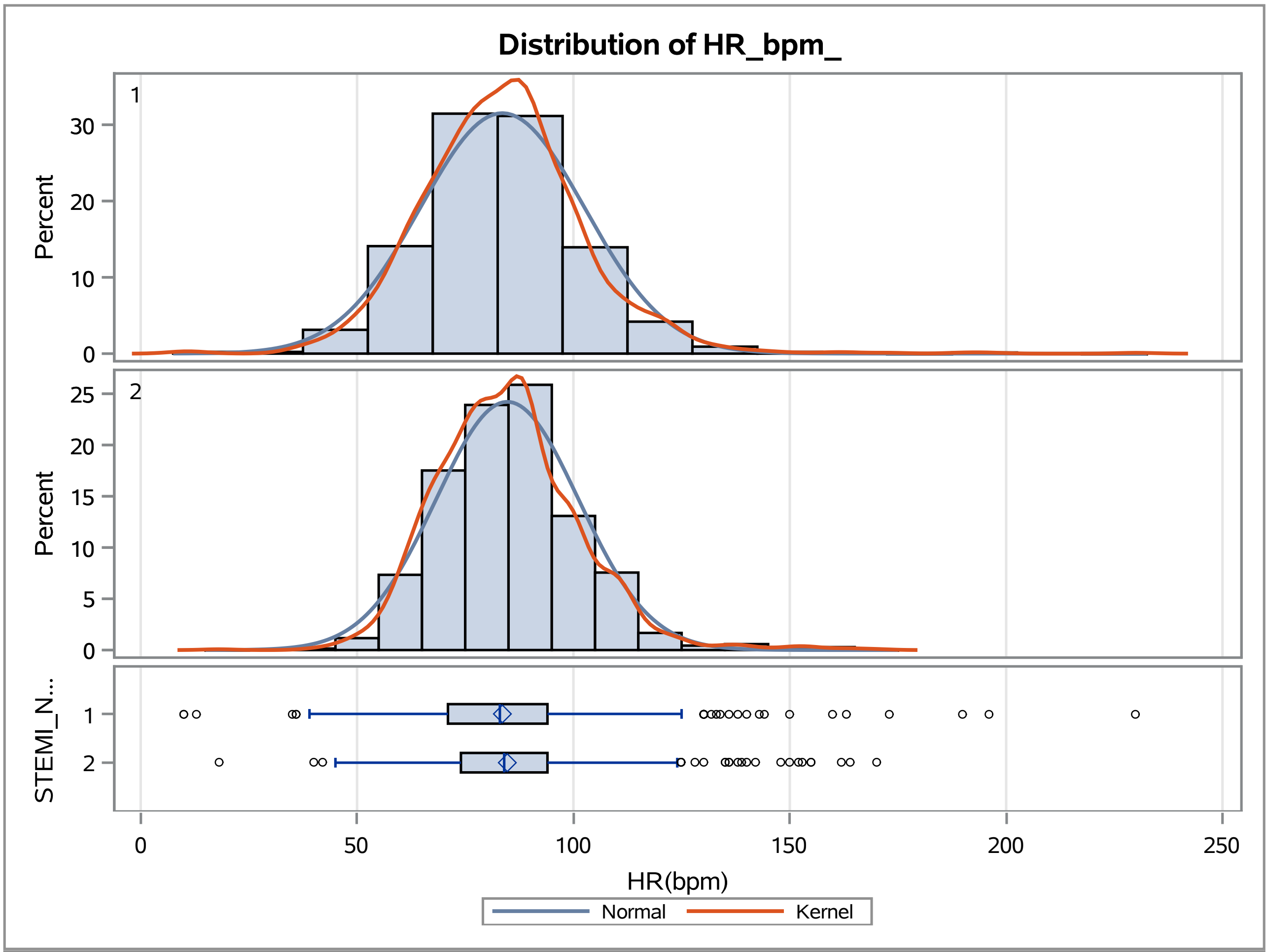

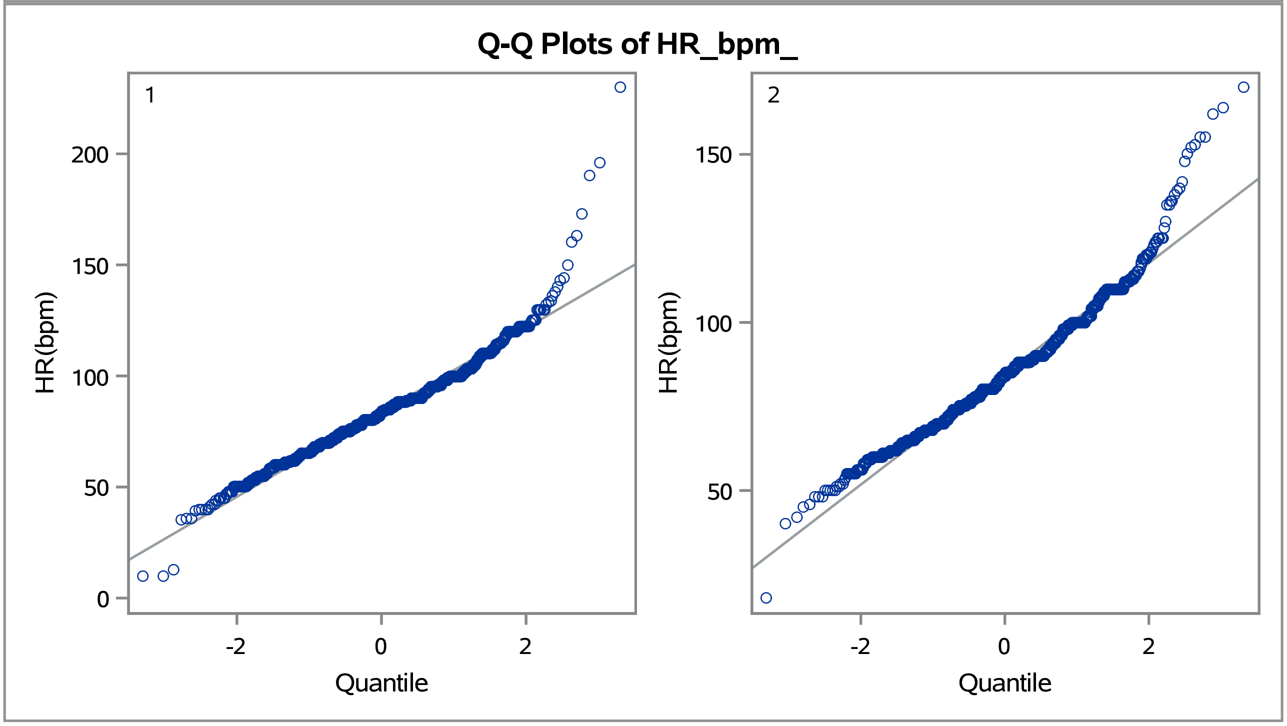

The TTEST Procedure

Variable: SBP_mmgH_ (SBP(mmgH))

Variable: SBP_mmgH_ (SBP(mmgH))

STEMI_NSTEMI	Method	N	Mean	Std Dev	Std Err	Minimum	Maximum
1		1313	132.2	26.6550	0.7356	40.0000	230.0
2		1376	137.4	23.9953	0.6469	60.0000	244.0
Diff (1-2)	Pooled		-5.1645	25.3289	0.9772		
Diff (1-2)	Satterthwaite		-5.1645		0.9796		

STEMI_NSTEMI	Method	Mean	95% CL Mean		Std Dev	95% CL Std Dev	
1		132.2	130.8	133.7	26.6550	25.6731	27.7156
2		137.4	136.1	138.7	23.9953	23.1311	24.9271
Diff (1-2)	Pooled	-5.1645	-7.0806	-3.2484	25.3289	24.6695	26.0248
Diff (1-2)	Satterthwaite	-5.1645	-7.0853	-3.2437			

Method	Variances	DF	t Value	Pr > |t|
Pooled	Equal	2687	-5.29	<.0001
Satterthwaite	Unequal	2626.8	-5.27	<.0001

Equality of Variances				
Method	Num DF	Den DF	F Value	Pr > F
Folded F	1312	1375	1.23	0.0001

The TTEST Procedure

Variable: SBP_mmgH_ (SBP(mmgH))

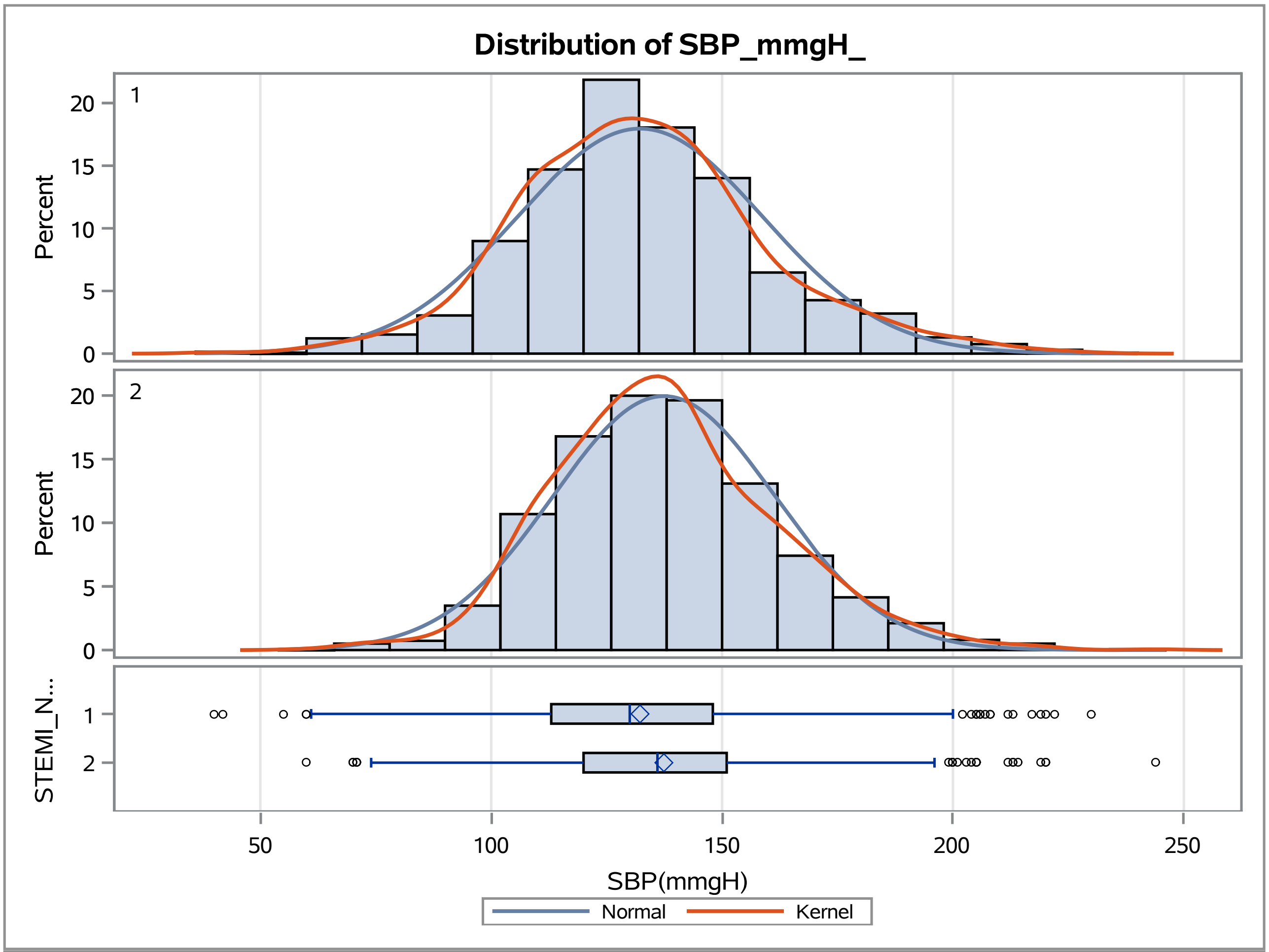

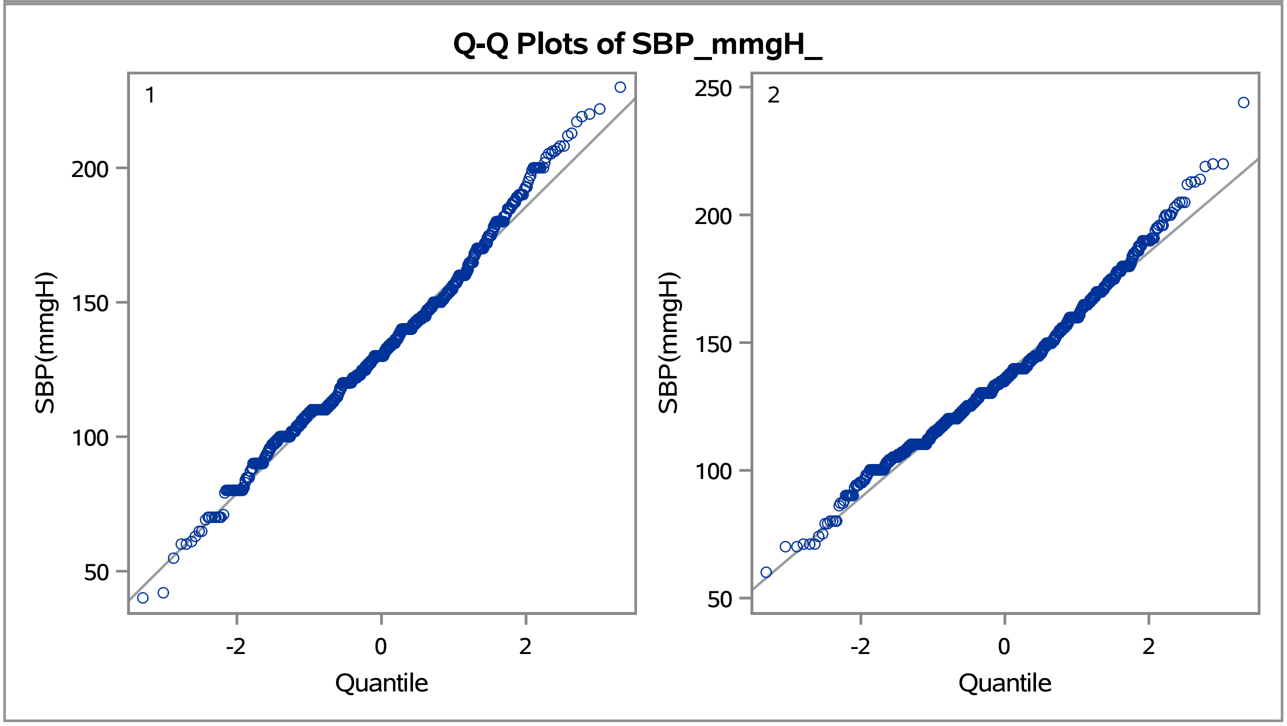

Supplement: S1 Data — (ZIP) [file pone.0331215.s011.zip › Raw data/table 1 results.pdf]
